# Cyclic Homo- and Heterohalogen Di-λ^3^-diarylhalonium Structures

**DOI:** 10.1021/jacs.3c02406

**Published:** 2023-06-13

**Authors:** Wei W. Chen, Margalida Artigues, Mercè Font-Bardia, Ana B. Cuenca, Alexandr Shafir

**Affiliations:** † BISi-Bonds Group, Institut de Química Avançada de Catalunya, IQAC-CSIC, c/Jordi Girona 20, 08034 Barcelona, Spain; ‡ BISi-Bonds/CRISOL Group, Department of Organic and Pharmaceutical Chemistry, Universitat Ramon Llull and Centro de Innovación en Química Avanzada (ORFEO-CINQA), Vía Augusta 390, 08017 Barcelona, Spain; § Department of Analytical and Applied Chemistry, Institut Químic de Sarrià, Universitat Ramon Llull, Vía Augusta 390, 08017 Barcelona, Spain; ∥ Unitat de Difracció de RX. Centres Científics i Tecnològics de la Universitat de Barcelona (CCiTUB), Universitat de Barcelona, c/Solé i Sabarís 1-3, 08028 Barcelona, Spain; ⊥ BISi-Bonds Group, Institut de Química Avançada de Catalunya, IQAC-CSIC, and Centro de Innovación en Química Avanzada (ORFEO-CINQA), 08034 Barcelona, Spain

## Abstract

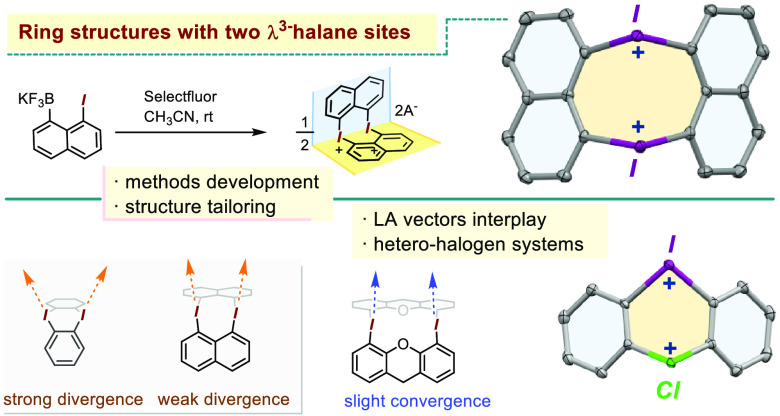

In the context of the ever-growing interest in the cyclic diaryliodonium salts, this work presents synthetic design principles for a new family of structures with two hypervalent halogens in the ring. The smallest bis-phenylene derivative, [(C_6_H_4_)_2_I_2_]^2+^, was prepared through oxidative dimerization of a precursor bearing the *ortho*-disposed iodine and trifluoroborate groups. We also report, for the first time, the formation of cycles containing two different halogen atoms. These present two phenylenes linked by hetero-(I/Br) or -(I/Cl) halogen pairs. This approach was also extended to the cyclic bis-naphthylene derivative [(C_10_H_6_)_2_I_2_]^2+^. The structures of these bis-halogen(III) rings were further assessed through X-ray analysis. The simplest cyclic phenylene bis-iodine(III) derivative features the interplanar angle of ∼120°, while a smaller angle of ∼103° was found for the analogous naphthylene-based salt. All dications form dimeric pairs through a combination of π–π and C–H/π interactions. As the largest member of the family, a bis-I(III)-macrocycle was also assembled using the quasi-planar xanthene backbone. Its geometry enables the two iodine(III) centers to be bridged intramolecularly by two bidentate triflate anions. In a preliminary manner, the interaction of the phenylene- and naphthalene-based bis-iodine(III) dications with a new family of rigid bidentate bis-pyridine ligands was studied in solution and the solid state, with an X-ray structure showing the chelating donor bonding to just one of the two iodine centers.

## Introduction

**Figure 1:**
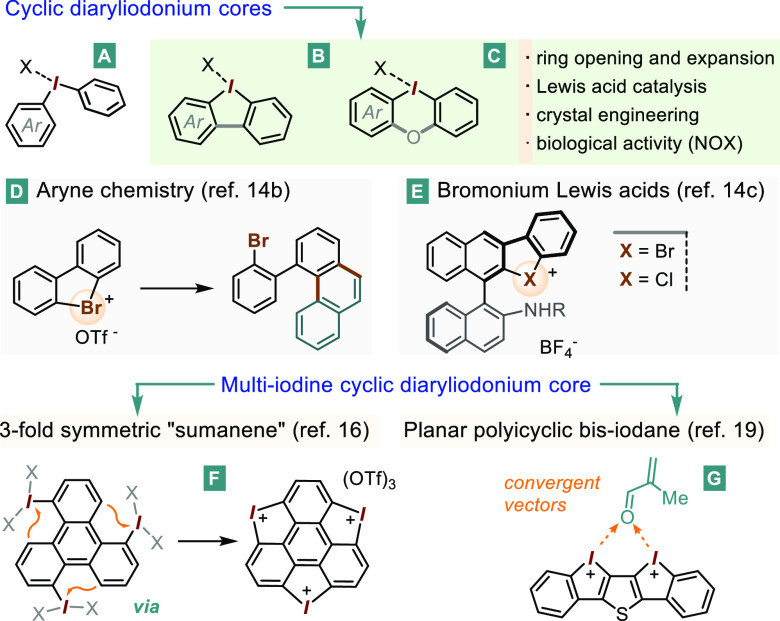
A sampling of cyclic diaryl-halogen(III) structures with some applications. **A**: canonical diaryliodonium motif; **B**, **C**: most common cyclic diaryliodonium structure types; **D**, **E**: recent application of bromonium and chloronium salts; **F**, **G**: examples of molecules presenting multiple diaryliodonium groups.

The term “diaryliodonium salt” refers to iodine(III) compounds in which the trivalent iodine center is bound to two aromatic rings and a third typically weakly coordinating anion (Figure 1). This structure class requires little introduction, given that the prototypical diaryliodonium motif (Figure 1, **A**)^1,2^ has been known for over 120 years,^3^ growing into an important class of aryl transfer agents. Diaryliodonium salts have been employed in a plethora of polar and radical processes, including metal-catalyzed and light-induced arylation reactions.^4^ In this context, the cyclic diaryliodonium derivatives containing mutually connected aryl groups have been of particular interest, with the iodine-containing ring structure imparting a series of new chemical and physical properties. For example, while the archetypal five-membered structure type **B** (Figure 1) is particularly stable toward conventional nucleophilic attack,^5^ this and the larger iodacycles (e*.*g., type **C**) undergo all sorts of ring-opening and ring-enlargement reactions under metal-catalyzed^6,7^ or single electron transfer (SET) conditions.^8^ Beyond this synthetic potential, cyclic diaryliodonium cations are also known to interfere with a variety of biological electron-transport systems. In fact, the parent diphenylene-iodonium (“DPI”) cation (archetype **B**) is widely used as a broad-spectrum go-to inhibitor of NADPH oxidases and other flavoenzymes,^9^ either as reporter/modulators of cellular activity^9b^ or as therapeutic candidates.^9c^ Recently, the ability of the Lewis acidic iodine(III) center to engage in highly directional intermolecular interactions, sometimes referred to as “halogen bonding”,^10^ has come under a spotlight in the fields of crystal engineering, molecular recognition, and Lewis acid organocatalysts.^11,12^ Another center of focus has been the revival (after decades of relatively little interest) of the hypervalent derivatives of the lighter two halogens: Br and Cl.^13,14^ Fresh examples include the bromonium-based cycloaddition reactions from the Wencel–Delord laboratory (Figure 1, **D**),^14a−14c^ as well as chiral Br- and Cl-centered onium salts as enantioselective halogen-bonding catalysts by Yoshida et al. (structure **E**).^14d^

In this context, our attention was drawn to the scarcely explored cyclic onium salts containing more than one iodine atom in a ring. A notable example of such compounds is the square-shaped macrocycle developed by Zhdankin and Stang, in which the near-90° C–I–C angles are used as corner pieces.^15^ Another example is the planar 3-fold symmetric iodine-doped sumanene derivative (Figure 1, **F**)^16^ obtained via an oxidation/E_A_S cyclization sequence. We note that routes such as this one are commonly used to prepare a variety of cyclic bis-diaryliodonium species,^17,18^ including the planar bis-diaryliodonium dication **G** (Figure 1). Interestingly, the latter exhibits exceptional Lewis acidity due to spatial convergence of the two iodonium C–I σ* vectors.^19^

**Figure 2:**
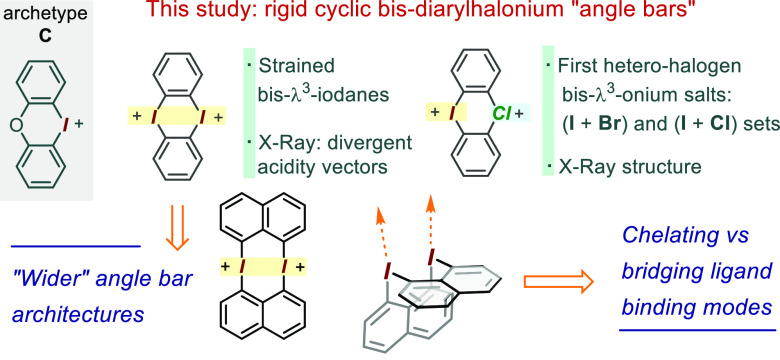
Principle bis-λ^3^-halonium angle bars developed in this study.

With these precedents, and prompted by our own recent work on heteroatom-containing iodonium salts,^20^ we wondered about the properties of a hitherto unknown cyclic structure related to the archetype **C** (Figures 1 and 2), but having not one, but two iodine(III) bridges. Beyond the sheer synthetic challenge of making this cycle, this structure class appears to be an interesting platform for new “angle bar” molecular geometries (provided a near-90° C–I–C angle), and for exploring other classes of interhalogen synergistic effects. As part of this new program in our lab, we now report the synthesis and X-ray structures of not only this bis-λ^3^-iodane architecture (I–I), but also the analogous heterohalogen six-membered cycle with the I–Br and I–Cl bridges. The latter two represent, to the best of our knowledge, the first examples of a bona fide hetero-λ^3^-organo-halogen derivative. We also report the synthesis and structure of larger (macro)cyclic bis-λ^3^-iodane angle bars, as well as their interaction modes—including chelating and bridging—with bidentate donor structures.

## Results and Discussion

### Bis-Halogen-Linked Bis-Phenyelene Cores

**Scheme 1:**
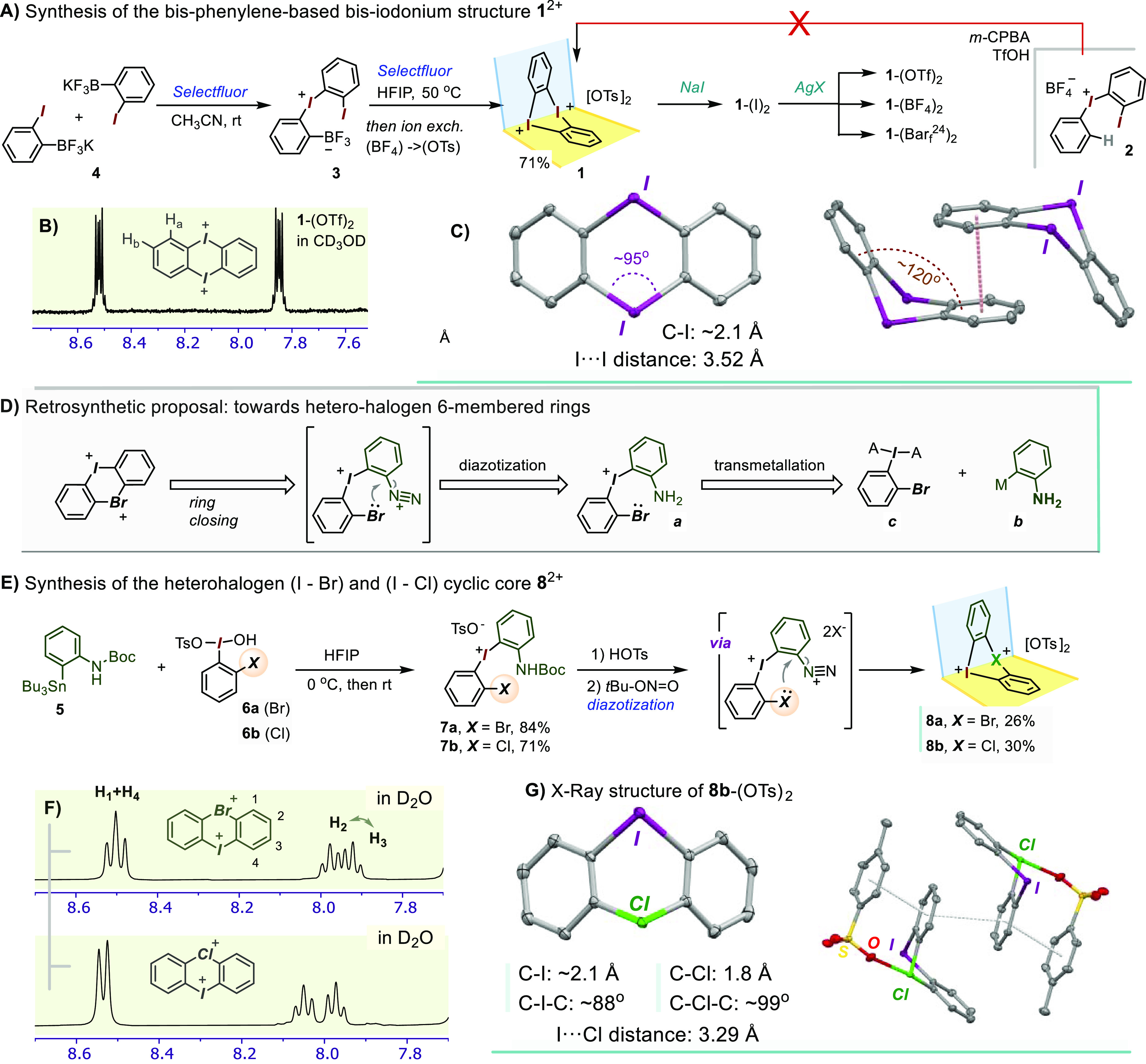
Synthesis and Characterization of the Cyclic Bis-diaryliodonium **1**^2+^ and the Hetero-Bis-diaryl-Halonium **8a**^2+^ and **8b**^2+^ A and B: Synthesis of **1**^2+^, along with a portion of its ^1^H NMR spectrum. C: Solid state structure of **1**^2+^ along the molecular *C*_2_ axis (left) and a view of the mutually fitting dication pairs (right) (thermal ellipsoids shown with a 50% probability, H and OTf omitted for clarity). D: retrosynthetic approach to **8**^2+^. E: synthesis of **8a**^2+^ and **8b**^2+^. F: Aromatic ^1^H NMR regions of **8a** (top) and **8b** (bottom). G: Solid state structure of **8b**^2+^; thermal ellipsoids shown with a 50% probability, H and OTs (fully or partially) omitted for clarity.

Our initial approach to the cyclic bis-iodonium **1**^**2+**^ was an electrophilic ring closing of the *ortho*-iodo-diphenyliodonium precursor **2**.^21^ However, attempts to cyclize **2** under oxidative conditions commonly used in the formation of cyclic iodonium salts were unsuccessful, likely due to the strongly electron-withdrawing effect exerted by the already-present iodine(III) group.^22^ Seeking to overcome this reluctance to C–H cyclization, we took notice of a recent report from the Legault laboratory on the oxidative dimerization of the *ortho*-iodo-phenyltrifluoroborate **4** to the interesting zwitterion **3**.^23^ Since the latter contains both an iodine and an *ortho* trifluoroborate group, we hoped that **4** might be directly dimerized to **1**^2+^ via two consecutive oxidation/transmetalation events. Our initial attempts to accomplish this transformation using Selectfluor as oxidant in CH_3_CN inevitably stopped at the intermediate **3**, mirroring the original report.^23^ In addition, attempts to use this solvent at higher temperatures led to significant amounts of the *ortho*-diiodobenzene side-product, ostensibly through the breakdown of **3** or of the putative cyclic target. Nevertheless, a breakthrough came thanks to the use of 1,1,1,3,3,3-hexafluoro-2-propanol (HFIP) as solvent. Hence, exposing **3** to excess Selectfluor at 40–50 °C in HFIP suppressed the formation of *ortho*-diiodobenzene, leading instead to a gradual appearance of a new species with only two ^1^H NMR resonances, found at 8.5 and 7.9 ppm (see Scheme 1, A and B), in a pattern consistent with the *C*_2*v*_-symmetric bis-diaryliodonium **1**^2+^. Further corroboration came from HR-MS(ESI+), which revealed a peak at *m*/*z* = 202.9358 for the target dication [(C_6_H_4_)_2_I_2_]^2+^ (calcd 202.9352 for *z* = 2). The product was initially formed as the tetrafluoroborate salt, i.e., **1**-(BF_4_)_2_. Nevertheless, given the crucial influence often exerted by the counterions in the chemistry and applications of a diaryliodonium fragment, the family was expanded with additional counterions. Hence, the BF_4_ salt could be converted to the virtually insoluble derivatives, either ditosylate **1**-(OTs)_2_ (71% based on **3**) or the di-iodide **1**-(I_2_). The −OTf, −BF_4_, and even the BAr_f_^24^ derivatives [BAr_f_^24^ = B(3,5-bis-trifluoromethyl-C_6_H_3_)_4_] were also synthesized through anion exchange with the corresponding silver salt. X-ray-quality crystals of **1**-(OTf)_2_ were grown through vapor diffusion of Et_2_O into the CH_3_CN solution. The salt crystallized in space group *P*1 and the solid state structure featured the tricyclic dication **1**^2+^ having a local *C*_2*v*_-symmetry. The two phenyl-containing planes intersect (at the I···I line) at a 120° angle (Scheme 1, C); while the C–I–C angles were measured at ∼95°. The I···I distance of 3.52 Å is somewhat shorter than the sum of the iodine van der Waals radii (∼3.8–4.0 Å), suggesting a degree of steric crowding between the two halogens. The bis-iodonium fragments are arranged in pairs (see Scheme 1, C, right), which are held together through π-stacking on their inner (concave) sides. Not shown are the additional principal I···O interactions with neighboring triflate anions (see SI) completing the roughly square planar environment around each iodine(III) center.

Having succeeded in the formation of the six-membered bis-iodane **1**^**2+**^, we sought to expand our study to rings incorporating two different halogen atoms. Indeed, despite a renewed spotlight on the organo-chloro(III) and -bromo(III) derivatives,^14,24^ we could find no precedent of molecules having two distinct high-valent halogen atoms, let alone an example of a heterohalogen ring structure.^25^ Given that the synthetic route designed for **1**^2+^ was deemed unsuitable for hard-to-oxidize lighter halogens, an alternative retrosynthetic sequence was envisioned to access the six-membered bromine(III)–iodine(III) cycle. The route would rely on the *ortho*-NH_2_ diaryliodonium precursors ***a*** (Scheme 1, D), with ring closing achieved via diazotization followed by cyclizative N_2_ displacement. The precursor ***a***, in turn, could arise via transmetalation using a suitably *ortho*-metalated aniline ***b***. This route, however, presents a series of challenges. One is the question of whether the diazotization event is even possible on a diaryliodonium core. Another is the actual synthesis of the *ortho*-amino derivative ***a***, a species with scarce precedent in the literature, having been described as difficult to prepare and highly unstable.^26^ Fortunately, albeit after considerable effort, a route was identified in which a reaction between the *ortho*-stannyl *N-*Boc aniline **5** and the Koser-type *ortho*-bromo λ^3^-iodane **6a** took place in HFIP to give the diaryliodonium intermediate **7a** in 84% yield (Scheme 1-E). A one-pot *N-*deprotection of **7a** with HOTs, followed by the addition of the diazotizing reagent *t*Bu-ON=O^14a^ led to the precipitation of an off-white powder. To our delight, the ^1^H NMR spectrum of this product displayed an aromatic ABCD pattern (clustered into two groups of resonances), in line with the *C*_*s*_-symmetric mixed iodine(III)–bromine(III) cyclic bis-halonium target **8a**-(OTs)_2_ (Scheme 1, F, top). The structure was further corroborated via HR-MS(ESI+) analysis, which revealed the (**8a-**OTs)^**+**^ peak at *m*/*z* = 528.8974 (vs calcd 528.8964), including the expected Br isotope pattern (see SI).

Seeking to extend this approach to the analogous mixed iodine(III)-chlorine(III) derivative, the *o*-Cl Koser derivative **6b** was transformed into the diaryliodinium salt **7b** in 71% yield. As had been the case for **7a**, this salt underwent diazotizative cyclization to give, in this case, the mixed I(III)–Cl(III) cyclic salt **8b**-(OTs)_2_.^27^ The ^1^H NMR analysis confirmed, once again, a *C*_*s*_-symmetric dicationic portion, albeit with only minimal chemical shift separation between the two *ortho*-halo positions H_1_ and H_4_ (Scheme 1, F, bottom). The HR-MS(ESI+) peak for (**8b-**OTs)^+^ was recorded at *m*/*z* = 484.9462, in line with the calculated value of 484.9470.

Although **8a** and **8b** were only sparingly soluble in water, their ^1^H NMR spectra in D_2_O showed them to be surprising stable in aqueous solutions, remaining unchanged for several weeks at room temperature (see SI, Figures S3 and S5, respectively). Paradoxically, after just a few hours in dmso-*d*_6_, both compounds were observed to undergo clean hydrolytic ring opening to the phenolic derivatives **S4** and **S6**, as observed by NMR and confirmed by HR-MS (see SI).

This made it somewhat challenging to grow single crystals of **8a** and **8b**, with earlier attempts invariably leading to hydrolysis. Nevertheless, X-ray-quality crystals of both species were obtained by taking advantage of the low product solubility in the reaction mixture upon their synthesis. Hence, a solution containing precursor **7a** (or **7b**), HOTs, and *t*Bu-ON=O in MeNO_2_ was briefly heated to 60 °C and then left undisturbed at room temperature. After several days, small colorless plate-like crystals of both **8a-**(OTs)_2_ and **8b-**(OTs)_2_ were observed. Their X-ray analysis revealed the expected cyclic heterohalogen structures which, as in the case of **1**, form the π-stacked pairs of dications (Scheme 1, G, for **8b**-(OTs)_2_; for **8a-**(OTs)_2_ see SI and the CIF file); these pairs, in turn, are boxed in between two tosylate anions via additional π-stacking interaction with the counterion's tolyl group. For both **8a**^2+^ and **8b**^2+^ structures, the C–X–C angles for the lighter halogen bridges (93.5° for Br and 98.6° for Cl) are larger than those for the corresponding C–I–C fragments (90.0° and 87.9°, respectively). This trend is in line with the predominant halogen p-orbital contribution for iodine(III) bonding and a larger s-orbital component associated with lighter halogens, as discussed recently by Stuart and co-workers.^28^

### Toward the Bis-diaryliodonium Angle Bar Structure

**Scheme 2:**
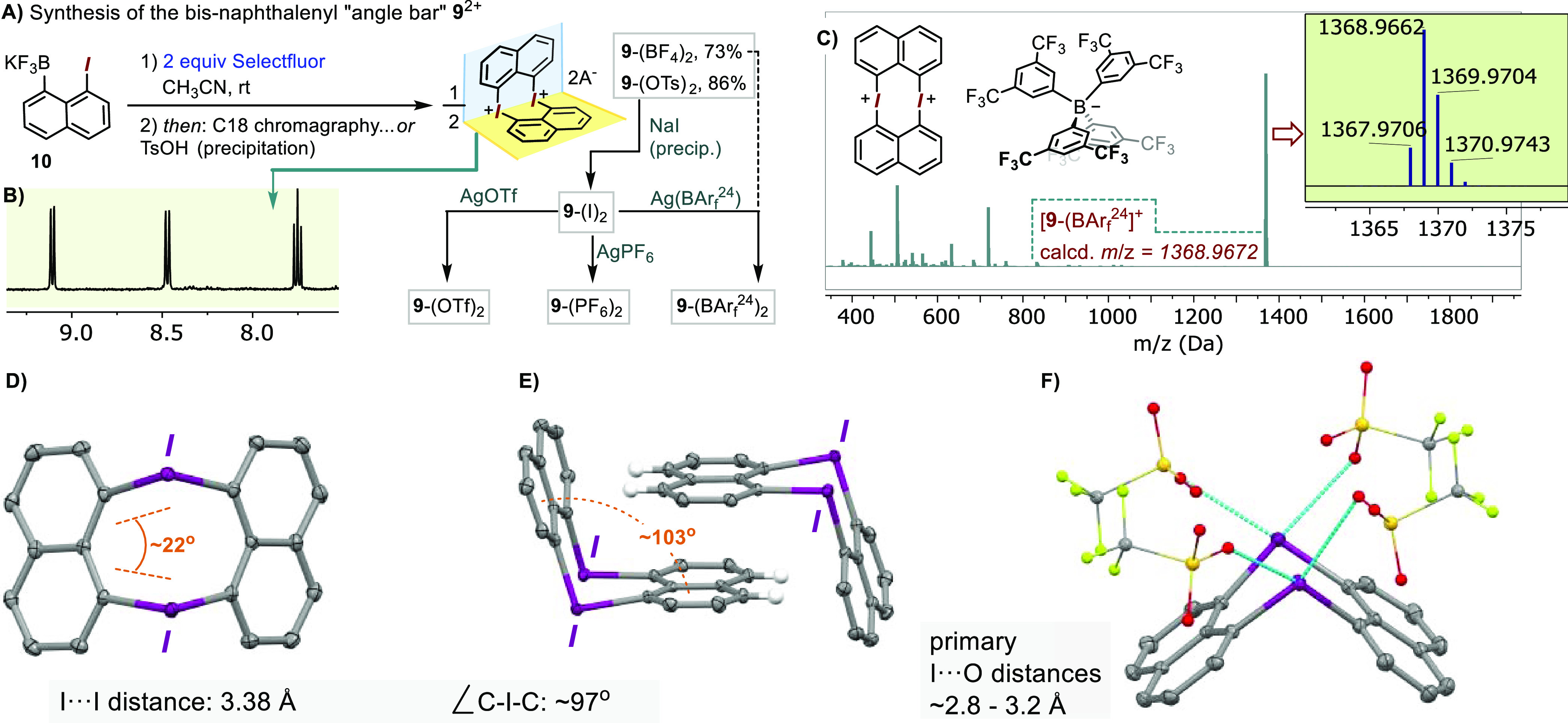
Synthesis and Characterization of the Naphthalenyl-Based Bis-λ^3^-diaryliodonium Dication of **9**^2+^ A and B: Synthetic route along with a portion of its ^1^H NMR spectrum. C: HR-MS(ESI+) analysis of **9**-(BAr_f_^24^)_2_. D, E, and F: partial X-ray ORTEP diagrams of **9**-(OTf)_2_ at 50% thermal ellipsoids; most H omitted for clarity.

As part of our broader interest in the cyclic bis-halonium cyclic structures, we wondered whether the use of the 1,8-disubstituted naphthalene backbone, or even a wider-spaced anthracene scaffold, could afford rigid structures that approach a 90° “angle bar” geometry. We envisage that in addition to serving as a blueprint to other rigid halogen-based molecular architectures, such structures could allow for the study of new types of interplay between halogen(III) Lewis acidity (or halogen bonding) vectors. Hence, aiming to apply an oxidative head-to-tail dimerization process, as seen with **1**, we began by synthesizing the *peri*-iodo-trifluoroborate precursor **10** from 1,8-diiodonaphthalene via a selective mono-magnesiation–borylation process (see SI). Gratifyingly, while the cyclization step in the synthesis of **1** required a stepwise approach and forcing conditions, simply treating **10** with excess Selectfluor in CH_3_CN at room temperature led, after 17 h, directly to the clean formation of the *C*_2*v*_-symmetric dication **9**^2+^ (Scheme 2, A).^29^ The resulting BF_4_ salt could be obtained in pure form in a 73% yield via reversed phase chromatography on C18 silica gel. Alternatively, treating the crude mixture with TsOH resulted in the precipitation of **9-**(OTs)_2_ as a clean pale-yellow solid in an 86% yield. The OTf, PF_6_, and BAr_f_^24^ derivatives were also obtained through a double anion exchange route via the insoluble **9-**(I)_2_ form, followed by treatment with the silver salt of the corresponding counterion. Alternatively, **9-**(BAr_f_^24^)_2_ could also be obtained directly from the initially formed tetrafluoroborate via salt metathesis with NaBAr_f_^24^. The *C*_2*v*_-symmetric dication **9**^2+^ presents three strongly deshielded ^1^H NMR resonances at ∼9.2, 8.5, and 7.9 ppm (see Scheme 2, B). The HR-MS(ESI+) analysis of the triflate salt (eluent spiked with formic acid) revealed a peak at *m*/*z* = 550.8991, in line with the value 550.8999 expected for [**9**]·(O_2_CH)^+^. Furthermore, the analysis of the BAr_f_^24^ derivative led to *m*/*z* = 1368.9662, matching well the theoretical value of 1368.9672 for [**9**]·(BAr_f_^24^)^+^ (Scheme 2, panel C). Crystals of **9**-(OTf)_2_ suitable for X-ray structure determination grew in space group *P*1 through vapor diffusion of Et_2_O into a CH_3_CN solution. As expected, the *peri* C–I vectors in each naphthalene unit diverged at an angle of ∼22° due to the steric repulsion between iodine atoms, a situation further confirmed by the relatively short I···I contact distance of 3.38 Å. The structure showed a 103° angle between the two naphthalene planes, a value closer to an idealized 90° angle than the ∼120° angle observed in **1** (see panels D and E in Scheme 2). Once again, the bis-cationic fragments are arranged in tightly fitting mutually complementing pairs, which appear to be held together by a combination of π-stacking and CH−π interactions (panel E). Each iodine atom also presents two main I···O interactions with the neighboring triflate anions to form an approximate square plane geometry (see panel F), along with a number of secondary I····O interactions.

**Scheme 3:**
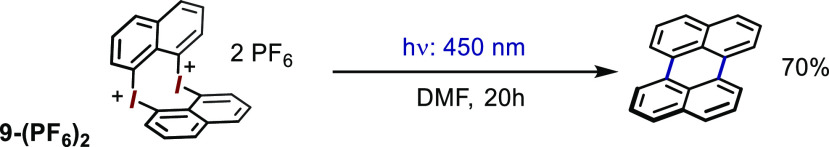
Photolytic Evolution of **9-**(PF_6_)_2_ to Perylene

The newly obtained **9**-(A)_2_ salts were found to degrade over time if not protected from light. Interestingly, perylene was identified as the main decomposition product. In fact, irradiation of **9-**(PF_6_)_2_ in a DMF solution at 450 nm led to a rather clean formation of perylene in ∼70% yield, likely through a sequence of homolytic C–I cleavage and C–C coupling steps (Scheme 3).

**Scheme 4:**
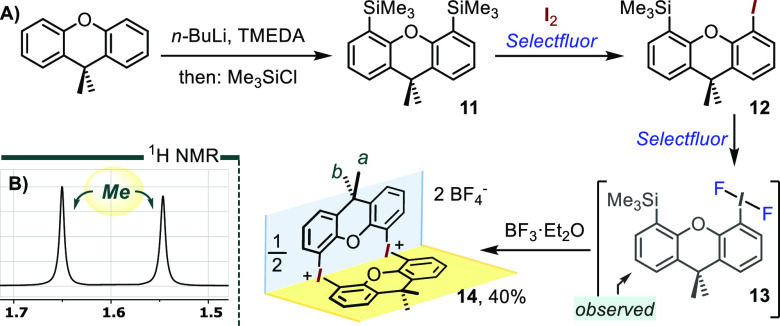
Cyclic Bis-iodonium Structure **14** Based on 9,9-Dimethylxanthene: (A) Synthesis Starting with 9,9-Dimethylxanthene; (B) Upfield Portion of the ^1^H NMR Spectrum

To our initial disappointment, attempts to produce a wider-spaced anthracene analogue of **9** were unsuccessful, likely due to the poor solubility of the anthracene precursor and the facile oxidative degradation of the anthracene core. Instead, we turned our attention to the geometrically similar 9,9-dimethylxanthene backbone. Hence, as shown in Scheme 4, A, the bifunctional precursor **12** was prepared from the 4,5-disilyl-9,9-dimethylxanthene **11** by a selective exchange of one of the −SiMe_3_ groups for I using the I_2_/Selectfluor combination. Next, exposing **12** to Selectfluor led to an initial formation of a new compound, **13**, tentatively identified by ^1^H and ^19^F NMR as bearing the hypervalent λ^3^-IF_2_ group.^30^ Heating this intermediate in the presence of BF_3_·Et_2_O helped induce the aryl transfer from silicon to iodine(III), leading to a gradual conversion of **13** to the more symmetric 12-membered macrocycle **14**^2+^. This result was supported by the observation of a simple three-resonance ^1^H NMR aromatic set for the new product (see SI). Furthermore, when measured in methanol-*d*_4_, the locked “angle bar” geometry leads to the splitting of the 9,9-di-Me signal into two Me singlets: one for the inner (***a***) and another for the outer (***b***) positions (Scheme 4, B). The **14**-(BF_4_)_2_ salt was isolated in 40% yield via reversed-phase chromatography, while the corresponding triflate salt was obtained through double anion metathesis via the sparingly insoluble **14**-(I)_2_ derivative. The HR-MS(ESI+) analysis produced a peak at *m*/*z* = 714.9847, consistent with 714.9837 calculated for [**14**]·(O_2_CH)^+^.

**Figure 3:**
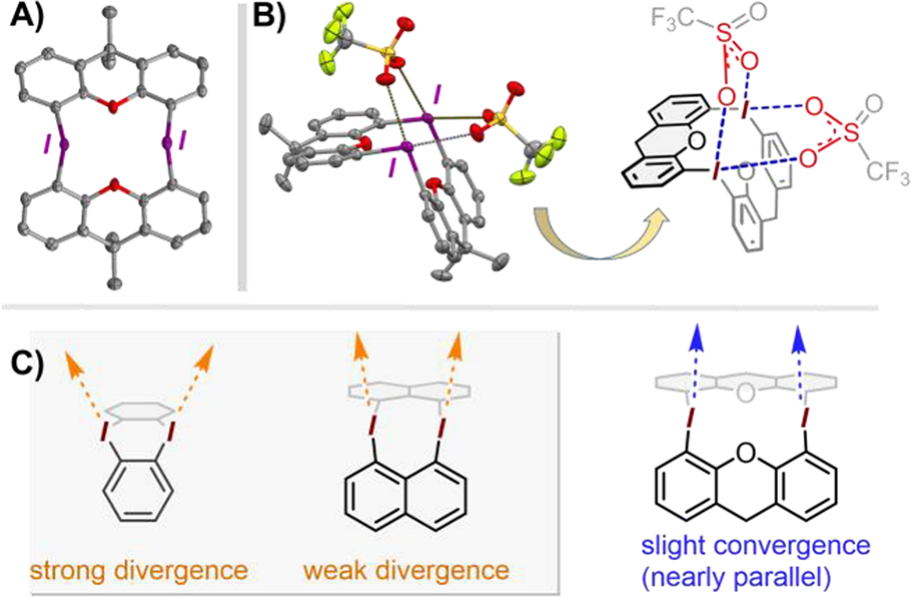
ORTEP-type diagram of the X-ray (single crystal) structure of **14**-(OTf)_2_. Only one of the two independent molecules shown, with protons omitted for clarity. (A) Top view of **14**^**2+**^ showing the 12-membered macrocycle. (B) Side view of the full molecule illustrating the bridging (I–O–S–O-I) η^2^-triflate ligands and the overall “paddle-wheel” geometry. (C) Comparison of divergence in the hypothetical C–I σ* vectors in cyclic bis-iodonium dications.

The structure of **14**-(OTf)_2_ was further assessed through single-crystal X-ray analysis (Figure 3). As expected, the xanthene backbone imparts a relatively large I··I spacing of ∼4.2 Å, a value similar to that expected in a hypothetical anthracene-based structure. At the same time, the xanthene moiety was found to display certain conformational flexibility around the central pyran ring.^31^ Interestingly, while the solid state structures of **1**^2+^ and **9**^2+^ show pairs of divergent Lewis acidity vectors stemming from the neighboring iodine(III) centers, the corresponding vector pairs in the xanthene-based **14**^2+^ show a small degree of convergence (see Figure 3, C). Thanks to this, the two triflate ligands in the structure of **14**^2+^ are each capable of connecting adjacent iodine centers via crystallographically symmetric I··S–O–S··I bridges, leading to a geometry broadly reminiscent of the “paddle-wheel” bimetallic complexes (see Figure 3, B).^32^

### Interaction with Rigid Bis-pyridine Ligands

Picking up on this last point, each halogen(III) center contributes with a pair of mutually perpendicular acidity directions. Therefore, all the bis-diarylhalonium dications shown thus far present four principal Lewis acidity vectors. In this scenario, a bidentate donor ligand could bind to such a dication in a number of modes. One possibility includes a ligand bridging between two adjacent iodine centers, as observed for the OTf ligands in **14**^2+^ (refer to Figure 3, B). Another is the chelating mode, i.e., two donor atoms binding to the same halogen site via its mutually perpendicular “vacant” sites.^33^ For the simple cyclic structure type **B** (see Figure 1), such scarcely precedented chelating mode was recently explored by the Huber laboratory employing bis-R(OR′)C=O: donor ligands based on a rigid bis-alkynyl-benzene backbone.^34^

**Scheme 5:**
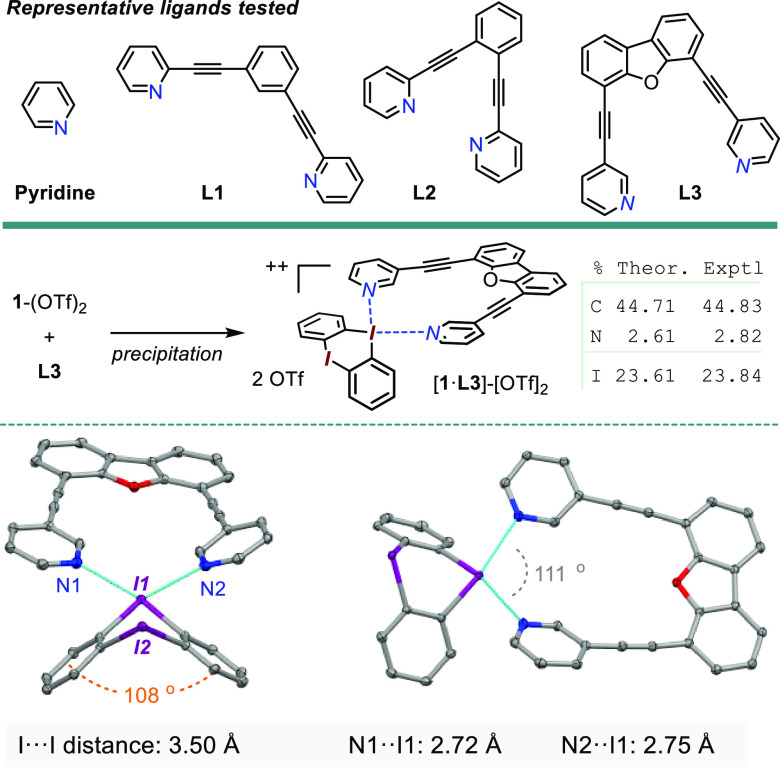
Selected Rigid Bis-pyridine Ligand Set, along with the Formation of a 1:1 Adduct between **1**^2+^ and **L3** Last panel (bottom) shows an X-ray ORTEP diagram (50% probability plots) of [**1·L3**]-(OTf)_2_; H and OTf omitted for clarity.

Combining this idea with the recent advances in pyridine-stabilized I^3+^, I^5+^, and, especially, I^1+^ structures,^35,36^ a small family of rigid bis-pyridine donors was synthesized varying the N–N distances and chelation angles (Scheme 5, top panel). These were then used, along with pyridine itself, in binding studies with the bis-diaryliodonium Lewis acceptors. In fact, pyridine was previously used as model donor to measure and benchmark the Lewis acidities of various diaryliodonum cations,^37^ as well as of their bromine(III) and chlorine(III) analogues.^28^ Hence, in a very preliminary assay, a portionwise addition of pyridine to a CD_2_Cl_2_ solution of **1**-(BAr_f_^24^)_2_ caused a gradual upfield shift of the **1**^2+^ resonances, which were then used to extract the binding constant. Assuming a 1:1 binding model, a value of *K*_a_ ∼ 609 M^–1^ was obtained, which would be 27 times higher than for the noncyclic salt **A** (Figure 1) and ∼4.7 times higher than the value of 130 M^–1^ that had been previously measured for the simple cyclic diaryliodonium prototype **B**.^28^ Even for the naphthalene-based **9**-(BAr_f_^24^)_2_, this titration led to a *K*_a_ ∼ 308 M^–1^, which although not as high as in **1**^2+^ is still ∼2.4 times higher than for **B**. We are cognizant, however, that the assumption of the 1:1 binding stoichiometry in this model may break down, especially at higher pyridine concentration, where the potential rise of the 1:2 adduct (one pyridine per each iodine center) may affect the accuracy of these preliminary binding constants. Next, the NMR titration of the model dications **1**^2+^ and **9**^2+^ with this ligand set revealed good levels of binding, achieving binding constants as high as *K* ∼ 10^4^ M^–1^ (see SI). We note, however, that in this preliminary module the exact binding modes could not be established unequivocally. Nevertheless, a combination of **1**-(OTf)_2_ with ligand **L3** did produce X-ray-quality single crystals with a one-to-one [**1**·**L3**]-(OTf)_2_ stoichiometry and the bis-pyridine chelating one of the two iodine atoms (Scheme 5). The adduct composition was further confirmed through elemental analysis of CHN, S, and I. Interestingly, the pyridine rings of the ligand appear to “push down” upon the phenylene groups, leading to an interplane angle of 108°, down from the 120° angle observed in the original **1**-(OTf)_2_.

## Conclusions and Outlook

In conclusion, this work amplifies the structure space of diaryliodonium salts to a new family of cyclic diaryliodonium structures containing two halogens in a ring. Noteworthy, despite over half a century of the history of six-membered cyclic iodonium salts, this is the first report describing the synthesis and X-ray structure of even the simplest ring structure **1**^**2+**^ having two iodine atoms bridging between two phenylene rings. This delay reflects the need to solve a synthetically difficult ring-closing step, now possible through a formal head-to-tail dimerization approach. Thanks to this methodology, cyclic bis-iodonium salts based on wider-spaced naphthalene and xanthene scaffolds were also synthesized, showing geometric features resembling a right-angle “angle bar” structure. Using a complementary stepwise approach, the structure class was further expanded to heterohalogen iodine(III)–bromine(III) and iodine(III)–chlorine(III) analogues **8**^2+^, which showed remarkable stability in water. The new bis-iodonium structures allow for the study of new types of interplay between chelating ligands and pairs of iodine(III) Lewis acid vectors, which includes both chelating and bridging binding modes. We envisage that this chemistry and the structure archetypes presented herein will serve as a blueprint for the development of a wider range of cyclic multihalogen structures that would be of interest in the realms such as synthetic methodology, molecular recognition, materials, organocatalysis, and self-assembly, to name a few.

## Supporting Information Available

Details on the synthetic procedures and product characterization, including NMR and MS data, and details on single-crystal X-ray structural characterization (PDF)
