# Supplementary material for: Cyclic Homo- and Heterohalogen Di-λ3-diarylhalonium Structures
Source: J Am Chem Soc. 2023 Jun 13;145(25):13796–804. doi: 10.1021/jacs.3c02406 (PMC10311461; doi:10.1021/jacs.3c02406)
Supplement: Supplementary file 1 — ja3c02406_si_001.pdf [file ja3c02406_si_001.pdf]

Electronic Supplementary Information

Cyclic homo- and hetero-halogen di-λ³-diarylhalonium structures

Wei W. Chen,[a,b] Margalida Artigues,[d] Mercè Font-Bardia,[e] Ana B. Cuenca,[b,c] Alexandr Shafir[a,c]**

[a] BISi-Bonds group, Institut de Química Avançada de Catalunya IQAC-CSIC, c/ Jordi Girona 20, 08034, Barcelona, Spain.
[b] BISi-Bonds/CRISOL group. Dept. of Organic and Pharma-ceutical Chemistry, Institut Químic de Sarrià, Universitat Ramon Llull, Vía Augusta 390, 08017 Barcelona, Spain.
[c] Centro de Innovación en Química Avanzada (ORFEO-CINQA), Spain
[d] Dept. of Analytical and Applied Chemistry, Institut Químic de Sarrià, Universitat Ramon Llull, Vía Augusta 390, 08017, Barcelona, Spain.
[e] Unitat de Difracció de RX. Centres Científics i Tecnològics de la Universitat de Barcelona (CCiTUB). Universitat de Barcelona, c/ Solé i Sabarís 1-3, 08028-Barcelona.

Table of content

1. General information: Material and Methods. S2
2. Synthesis and Characterization S4
3. ¹H-NMR titration experiments S35
4. References S44
5. NMR Spectras S46
6. X-Ray diffraction structural determination details. S102

1. General information: Material and Methods.

Reagents. All commercially acquired reagents were used as received unless indicated otherwise. Sodium tetrakis[3,5-*bis*(trifluoromethyl)phenyl]borate ($NaBAr_f^{24}$) was synthetized according to a reported protocol.[1]

Reaction conditions. Most reactions requiring inert atmosphere were conducted under argon atmosphere using standard Schlenk line techniques. When indicated, reactions were conducted in septum-sealed screw-top tubes (Kimble®), so that the Ar atmosphere could be created by applying evacuate/refill cycles *via* a needle coupled to Schlenk line. All other reactions were performed employing standard organic synthesis protocols.

Chromatography. Thin layer chromatography (TLC) was performed using Merck aluminium-backed plates of TLC Silica gel 60 F254; the plates were revealed using UV light at 254 nm or by staining using potassium permanganate. Standard Flash Column chromatography was accomplished using silica gel (60 Å pore size, 230-400 µm mesh size). GC-LRMS measurements were recorded on an Agilent 6890 chromatograph equipped with an Agilent 5973 Network MS detector

Liquid Chromatography coupled to a High-Resolution Mass Spectrometry**.** Analyses were carried out at the IQS-SCIEX DEMO LAB Facility, using a EXION LC (Sciex) CHROMATOGRAPH coupled to a QTOF X500B (Hybrid LC/MS/Ms quadrupole time-of-flight mass spectrometer). Direct injections were performed using as mobile phase A Milli-Q water (w/o 0.1% of Formic acid) and as mobile phase B CH_3CN (w/o 0.1% of Formic acid) (50:50).

Nuclear Magnetic Resonance. Spectroscopic experiments for the characterization of compounds were carried out at the Servicio de Resonancia Magnética Nuclear of the IQAC-CSIC and at the Structural Determination facility of the IQS, on a Bruker Avance NEO 400 MHz (9.3950 T) and Varian Mercury 400 MHz (9.3950 T) instrument, respectively (400 MHz for ^1H and 101 MHz for ^{13}C). The ^1H and ^{13}C chemical shifts (δ_H) are quoted in parts per million (ppm) and referenced to the appropriate NMR solvent peak(s), which for ^1H measurements would correspond to the residual *protio* component of the deuterated solvent. The ^{13}P chemical shifts are quoted with respect to H_3PO_4 (85% aq, 0.00 ppm), while the ^{19}F signals are reported relative to $CFCl_3$ (0.00 ppm). The ^{11}B chemical shift are referenced relative to the external $BF_3{\cdot}Et_2O$ resonance at 0.00 ppm. 2D-NMR experiments COSY, HSQC and HMBC were used where necessary in assigning NMR spectra. Spin-spin coupling constants (*J*) are reported in Hertz (Hz).

Other. Infrared spectra were recorded on an Avatar 360 FT-IR spectrophotometer equipped with Smart *i*TR window and are reported in cm^{-1}. Centrifugation were performed on a Compact

Centrifuge Z 206 A equipped with an angle rotor for 6 x 50 ml conical tubes. The samples were introduced into a Corning™ Falcon™ 50 mL Conical Centrifuge Tubes. CHNSI Elemental Analysis was performed at the Microanalysis Service of IQAC-CSIC.

2. Synthesis and Characterization

Synthesis of (2-iodophenyl)(phenyl)iodonium tetrafluoroborate salt 2:

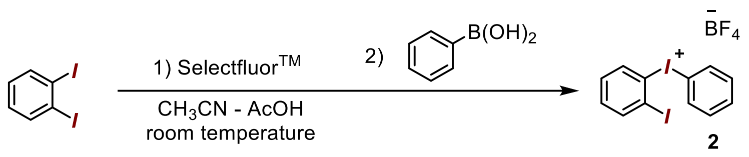

First step of the reaction performed following a modified protocol reported by the laboratory of Shreeve and et al.[2] In a round-bottom flask, 1,2-diiodobenzene(660 mg, 2.00 mmol) was dissolved in 50 mL of CH_3CN/AcOH (4:1) solvent mixture. Subsequently, Selectfluor™ (1.77 g, 5.00 mmol, 2.50 equiv.) was added in one portion and the resulting mixture was stirred for 5 hours at room temperature. After this time, phenyl boronic acid (268 mg, 1.10 mmol) was added to the flask and the mixture was stirred for an additional 2 hours at room temperature. Then, solvents were removed under reduced pressure by rota-evaporation and the remaining crude mixture was diluted with ~50 mL H_2O and extracted with CH_2Cl_2 (3x25 mL). The combined organic fraction was dried over $MgSO_4$, filtered and the solvent was removed by rota-evaporation. The oily residue was dissolved in a minimum amount of CH_2Cl_2 (~5.0 mL), followed by ~40 mL pentane. The mixture was stirred vigorously until total precipitation of a solid. The suspension was filtered, and the resulting solid residue was washed several times with pentane, followed by Et_2O to afford a brown solid, 476 g. Yield: 48%.

^{1}H NMR (400 MHz, DMSO-d$_6$) δ 8.53 (dd, J = 7.9, 1.4 Hz, 1H), 8.21 – 8.10 (m, 3H), 7.69 (t, J = 7.4 Hz, 1H), 7.61 – 7.49 (m, 3H), 7.41 (td, J = 7.6, 1.4 Hz, 1H). *Data consistent with reported compound.*[3]

Attempted cyclization of 2 under oxidative conditions

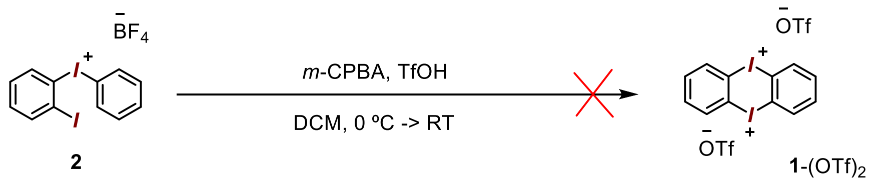

In an oven-dried screw-cap tube flask equipped with a magnetic stir bar was charged with **2** (10 mg, 0.02 mmol). The content was evacuated and backfilled with argon three times. The solid was dissolved with dry dichloromethane (1.0 mL), followed by the addition of *m*-CPBA (15 mg, 0.06 mmol, 3.00 equiv.) and the mixture was allowed to stir for 5 minutes. Then, the content was cooled to 0 °C in an ice/water bath. TfOH (18 mg, 11 μL, 0.12 mmol, 6 equiv.) was added *via* syringe into the reaction at 0 °C. Step in which the solution rapidly turned into a dark-brown mixture, and it was allowed to stir for 30 min at 0 °C and then, 1 hour at room temperature.

After that time, the crude mixture was concentrated under reduced pressure and the resulting residue was digested with Et_2O (2.0 mL), the precipitate was filtered and dried under high vacuum. The analysis by ^1H NMR in DMSO-d_6 did not indicate the formation of the cyclic product, but the oxidation of iodine did (see Figure S1)

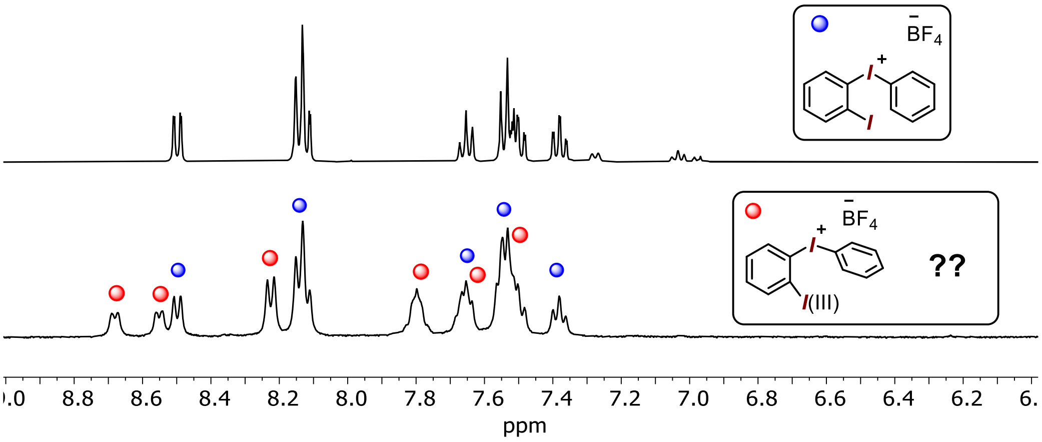

Figure S1: ^1H NMR spectra in DMSO-d_6 of **2** (upper spectra) and isolated solid residue (bottom spectra) from the attempted reaction of cyclization of **2** under oxidative conditions.

Synthesis of (2-iodophenyl)boronic acid:

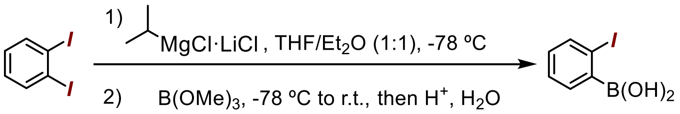

Reaction was conducted under Argon atmosphere. An oven-dried Schlenk flask equipped with a magnetic stir bar was charged with 1,2-diiodobenzene (3.3 g, 10.00 mmol) and dissolved with 100 mL of a mixture of dry THF-Et_2O (1:1). The flask content was cooled to -78 °C in a dry ice/acetone bath, once reached that temperature, a solution of Turbo Grignard 1.3M in THF (7.70 mL, 10.00 mmol) was added dropwise to the mixture and it was stirred 2h at -78 °C. After that time, $B(OMe)_3$ (3.4 mL, 30.0 mmol) was added in one portion to the content at -78 °C. Subsequently, the reaction mixture was allowed to reach room temperature and stirred overnight. Then, ~100 mL of an aqueous solution of HCl 10% was added and the mixture was stirred for 30 min. The resulting layers were separated, and the aqueous phase was extracted with Et_2O (3 x 75 mL). The combined organic phase was washed with brine, dried over $MgSO_4$ and filtered. After concentration in *vacuo*, the residue was purified by flash column chromatography on silica gel using as eluent a gradient mixture of hexanes:AcOEt (10:1 to 4:1), R_f = 0.21 in 4:1 hexanes/EtOAc. White solid, 1.83 g, yield: 74%.

The analysis by NMR displays a mixture of the boronic acid and boroxine in a ratio 1:1 in $CDCl_3$. Both species are reported below:

<u>*Boronic acid:*</u> **^{1}H NMR** (400 MHz, $CDCl_3$) δ 7.86 – 7.81 (m, 2H), 7.39 (td, *J* = 7.5, 1.1 Hz, 1H), 7.12 (ddd, *J* = 7.9, 7.4, 1.9 Hz, 1H), 5.13 (s, 2H, B(O**H**)$_2$).

<u>*Boroxine:*</u> **^{1}H NMR** (400 MHz, $CDCl_3$) δ 8.37 (dd, *J* = 7.5, 1.9 Hz, 3H), 8.06 – 7.98 (m, 3H), 7.48 (td, *J* = 7.4, 1.1 Hz, 3H), 7.20 (ddd, *J* = 7.9, 7.3, 1.9 Hz, 3H).

Synthesis of potassium 2-iodophenyltrifluoroborate 4.

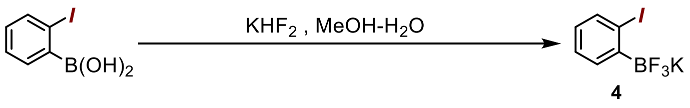

The synthesis was performed following an experimental procedure reported by Legault and coworkers.[4] In a round-bottom flask, (2-iodophenyl)boronic acid (124 mg, 0.50 mmol) was dissolved in 1.0 mL of methanol. In a vial, potassium bifluoride (129 mg, 1.65 mmol, 3.3 equiv) was dissolved in 0.5 mL of water and this solution was added dropwise to the round bottom flask under vigorous stirring. After an hour of stirring at room temperature, the solvents were evaporated. Next, the solid residue was further dried under high vacuum for 1 hour. The flask was washed with 10 mL of acetone (GC quality) and the solution was filtered through a Pasteur Pipette with a plug of cotton (The process was repeated 4 times). The resulting clear solution was concentrated to dryness. The solid thus obtained was washed with Et_2O (3 x 10 mL) and decanted. The resulting white solid was dried under high vacuum affording **4** as a white solid (Quantitative yield).

^{1}H NMR (400 MHz, Methanol-*d*$_4$) δ 7.70 (d, *J* = 7.8 Hz, 1H), 7.49 (dd, *J* = 7.6, 1.9 Hz, 1H), 7.16 (t, *J* = 7.3 Hz, 1H), 6.80 (td, *J* = 7.6, 1.9 Hz, 1H). *Data consistent with reported compound.*[4]

Synthesis of 2-[(2-iodophenyl)iodonio]phenyltrifluoroborate 3.

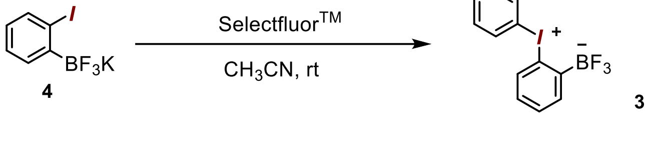

The synthesis was performed following an experimental procedure reported by Legault and coworkers.[4] In a round-bottom flask, potassium 2-iodophenyltrifluoroborate **4** (930 mg, 3.00 mmol) was dissolved in 30 mL of CH_3CN. Subsequently, Selectfluor™ (797 mg, 2.25 mmol) was added in one portion and the resulting mixture was stirred for 6 hours at room temperature. Then, solvent was removed under reduced pressure by rota-evaporation and the remaining

crude mixture was diluted with ~60 mL H_2O and extracted with CH_2Cl_2 (3x20 mL). The combined organic fraction was dried over $MgSO_4$, filtered and the solvent was removed by rota-evaporation. The resulting residue was dissolved in a minimum amount of CH_2Cl_2 (~3.0 mL), followed by ~30 mL pentane. The mixture was stirred vigorously until total precipitation of a solid. This was filtered and washed several times with pentane to afford a white solid, 524 mg. Yield: 71%.

^{1}H NMR (400 MHz, CD_3OD) δ 8.23 (dd, *J* = 7.7, 1.7 Hz, 1H), 8.14 (dd, *J* = 7.9, 1.6 Hz, 1H), 7.75 (dd, *J* = 7.3, 1.9 Hz, 1H), 7.57 – 7.43 (m, 3H), 7.33 – 7.28 (m, 1H), 7.24 (ddd, *J* = 8.5, 7.0, 1.9 Hz, 1H). *Data consistent with reported compound.*[4]

Synthesis of 1-(OTs)₂:

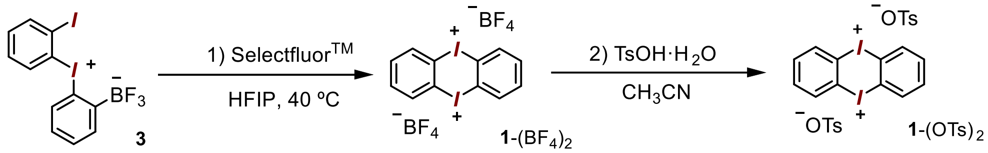

Reaction was conducted under Argon atmosphere and HFIP was degassed prior to use. In a Schlenk flask, trifluoro(2-((2-iodophenyl)iodonio)phenyl)borate **3** (474 mg, 1.00 mmol) was dissolved in 20 mL of dry HFIP. Followed by the addition of Selectfluor™ (1417 mg, 4.00 mmol) in one portion at room temperature, and the resulting mixture was stirred for 4 days at 40 °C. *(At this point, p-toluenaldehyde (29.5 µL, 0.25 mmol) was added as internal standard into the mixture. The analysis by ^{1}H-NMR in Acetone-d of an aliquot of the crude mixture showed a 55% yield of **1**-(BF_4)$_2$, see Figure S2B)*. Next, the reaction mixture was heated to 50 °C and allowed to react for additional 24 hours *(Another aliquot was analysed at this point, which indicated a yield of 68% of **1**-(BF_4)2 and total consumption of starting material **3**, see Figure 2C).*

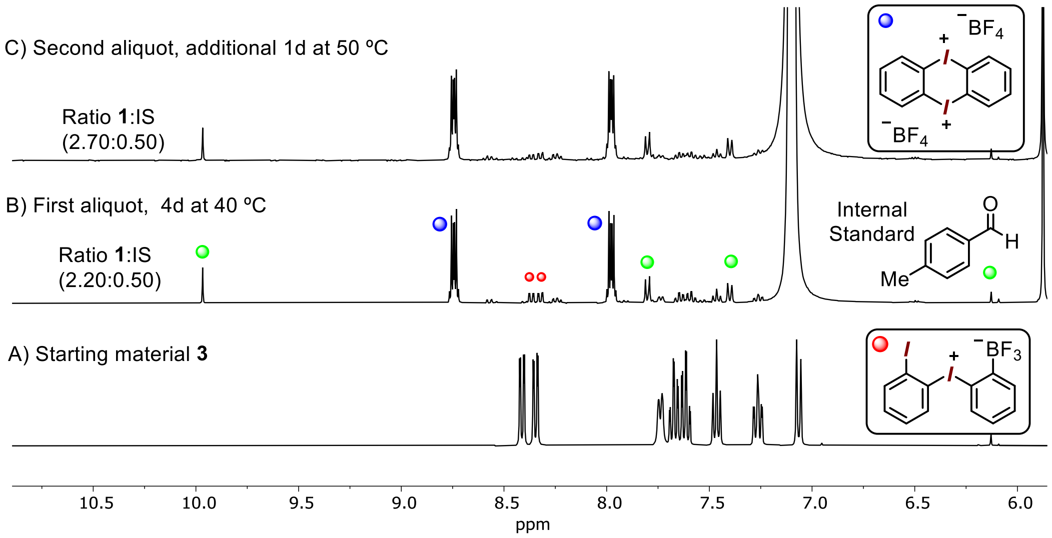

Figure S2: ^{1}H NMR spectra in Acetone-d of **3** (A), crude mixture after 4 days at 40 °C (B) and crude mixture after an additional 1 day at 50 °C (C).

Then, solvent was removed under reduced pressure by rota-evaporation. The resulting pale-yellow solid residue was dispersed in 30 mL CH_3CN. Then, TsOH·H_2O (791 mg, 4.00 mmol) was added to the mixture and stirred for 30 minutes leading to the precipitation of the target product as a white solid. The resulting suspension was introduced into a Falcon tube, subsequently centrifugated (6000 *rpm* for 60 seconds) and the supernatant liquid was extracted by Pasteur Pipette *(The solid residue was dispersed again in 20 mL CH_3CN, shaken manually, and centrifugated/decanted).* The resulting solid residue was dried under high vacuum to afford a white solid, 531 mg. Yield: 71%.

^{1}H NMR (400 MHz, DMSO) δ 8.53 – 8.39 (m, 4H, *meta C-**H***), 7.86 – 7.77 (m, 4H, *ortho C-**H***), 7.47 (d, *J* = 8.0 Hz, 4H, OTs), 7.11 (d, *J* = 7.9 Hz, 4H, OTs), 2.29 (s, 6H, OTs). **^{13}C NMR** (101 MHz, DMSO) δ 145.36 (OTs), 137.84 (OTs), 137.47 *(meta **C**-H)*, 133.65 *(ortho **C**-H)*, 128.13 (OTs), 125.49 (OTs), 124.23 (C-***I***), 20.79 (OTs). **HRMS** (ESI$^+$) m/z calcd for $C_{19}H_{15}I_2O_3S^+$ [M – OTs]$^+$ 576.8826, found 576.8821.

Synthesis of 1-(I)$_2$:

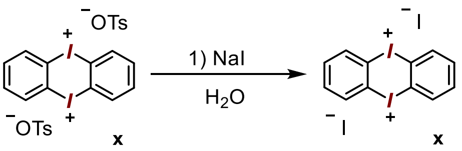

In a Falcon tube, AB-Ph-OTs **x** (38 mg, 0.05 mmol) was dissolved in 5.0 mL of H_2O. Then, NaI (75 mg, 0.50 mmol, 10.0 equiv.) was introduced into the mixture leading to the precipitation of the target product in iodide form **x** as a pale-yellow solid and the resulting mixture was stirred for 30 minutes at rt. The suspension was centrifugated (6000 *rpm* for 1 minute) and the supernatant liquid was extracted by Pasteur Pipette. The resulting residue was dispersed again in 5.0 mL of H_2O, manually shaken for 1 min, centrifugated (6000 *rpm* for 1 minute) and the resulting clear liquid was decanted (*Process repeated 2 times*). The solid was dried under high vacuum to afford a pale-yellow solid. Yield: quantitative

^{1}H NMR (400 MHz, DMSO-d$_6$) δ 8.48 – 8.40 (m, 4H), 7.81 – 7.72 (m, 4H).

Synthesis of 1-(BF$_4$)$_2$:

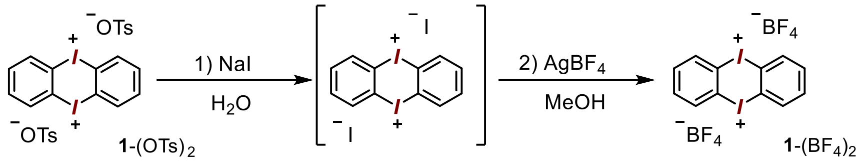

In a 50 mL Falcon tube, **1**-(OTs)$_2$ (187 mg, 0.25 mmol) was dispersed in 10 mL of H_2O. Then, NaI (285 mg, 1.50 mmol, 6.0 equiv.) was added and stirred for 10 min leading to the precipitation of the target product in iodide form as a pale-yellow solid. The suspension was centrifugated (6000 *rpm* for 1 minute) and the supernatant liquid was extracted by Pasteur Pipette (*The residue was digested again with 10 mL of H_2O, centrifugated and decanted*). The resulting residue was dispersed in 10 mL of MeOH, followed by the addition of $AgBF_4$ (107 mg, 0.55 mmol, 2.2 equiv.) and vigorously stirred for 10 min. After that time, the suspension was filtered through a nylon of 22 µm and additional MeOH (3 x 5 mL) was used to rinse/wash the flask, as well as the filter. The filtrate was concentrated under reduced pressure affording a white residue that was washed with dichloromethane (3 x 5 mL). The solid was dried under high vacuum to afford a white solid. Yield: quantitative

^{1}H NMR (400 MHz, CD_3OD) δ 8.58 – 8.51 (m, 4H), 7.90 – 7.84 (m, 4H). **^{13}C NMR** (101 MHz, CD_3OD) δ 138.75, 135.72, 123.44 (C-***I***). **^{19}F NMR** (376 MHz, CD_3OD) δ -153.86 ($^-BF_4$). **^{11}B NMR** (128 MHz, CD_3OD) δ -1.13. **HRMS** (ESI$^+$) m/z calcd for $C_{12}H_8I_2^{2+}$ $[M – 2BF_4]^{2+}$ 202.9352, found 202.9353.

Synthesis of 1-(OTf)$_2$:

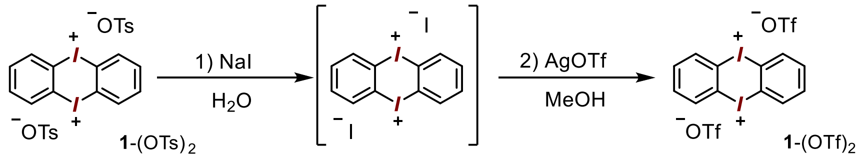

In a Falcon tube, **1**-(OTs)$_2$ (75 mg, 0.10 mmol) was dissolved in 5.0 mL of H_2O. Then, NaI (90 mg, 0.60 mmol, 6.0 equiv.) was introduced into the mixture leading to the precipitation of the target product in iodide form as a pale-yellow solid. The suspension was centrifugated (6000 *rpm* for 1 minute) and the supernatant liquid was extracted by Pasteur Pipette. The resulting residue was dispersed in 5.0 mL of MeOH, followed by the addition of AgOTf (62 mg, 0.24 mmol, 2.4 equiv.) and stirred 10 min. After that time, the suspension was filtered through a nylon of 22 µm and additional MeOH (2 x 5 mL) was used to rinse/wash the flask, as well as the filter. The filtrate was concentrated under reduced pressure affording a white residue that was washed with dichloromethane (2 x 5 mL). The solid was dried under high vacuum to afford a white solid. Yield: quantitative

^{1}**H NMR** (400 MHz, CD_3OD) δ 8.55 (ddd, *J* = 6.1, 3.5, 0.5 Hz, 1H, *ortho C-**H***), 7.89 (ddd, *J* = 6.1, 3.5, 0.5 Hz, 1H, *meta C-**H***). ^{13}**C NMR** (101 MHz, CD_3OD) δ 138.75 (*ortho* **C**H*)*, 135.79 (*meta* **C**H*)*, 123.47(C-***I***). ^{19}**F NMR** (376 MHz, CD_3OD) δ -80.11 ($^{-}$OTf). **HRMS** (ESI^+) m/z calcd for $C_{13}H_8F_3I_2O_3S^+$ $[M - OTf]^+$ 554.8230, found 554.8245.

Synthesis of 1-(BAr$_f$^{24})$_2$:

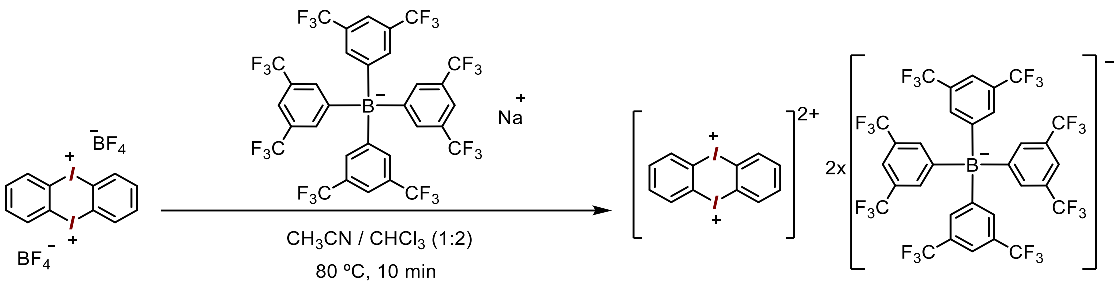

In a screw-cap tube was charged with **1**-(BF_4)$_2$ (58 mg, 0.10 mmol) and sodium tetrakis[3,5-*bis*(trifluoromethyl)phenyl]borate ($NaBAr_F$, 178 mg, 0.20 mmol), followed by the addition of 2.0 mL of CH_3CN. The pale-yellow mixture was stirred for 5 minutes at room temperature and 4 mL $CHCl_3$ was added. The tube was sealed and stirred at 80 °C for 10 min. After that time, the pale-brown mixture was allowed to cool down to room temperature and concentrated to dryness under reduced pressure. Then, DCM (10 mL) was added to the resulting residue, manually shaken, and filtered through a nylon filter of 22µm. The filtrate was concentrated under reduced pressure affording a brown oil, that was digested with dry $CHCl_3$ (0.5 mL) under vigorous stirring for 5 min. The mixture was allowed to settle in which a brown oil was deposited on the bottom of the flask and the clear supernatant was discarded. *(This washing process was repeated 3 times)* The brown oil was dried under reduced pressure, observing a bubbling from the oil and the subsequent solidification of the oily residue to afford a pale-yellow solid. This solid was further dried under high vacuum affording 129 mg. Yield: 61%.

^{1}**H NMR** (400 MHz, MeOD) δ 8.57 – 8.45 (m, 4H), 7.90 – 7.82 (m, 4H), 7.70 – 7.48 (m, 24H). ^{13}**C NMR** (101 MHz, MeOD) δ 162.85 (q, $J_{C\text{-}B}$ = 49.9 Hz), 138.66, 135.77 (*contain two overlapped peaks*), 130.42 (qq, $J_{C\text{-}F}$ = 31.7, $J_{C\text{-}B}$ = 2.8 Hz), 125.74 (q, $J_{C\text{-}F}$ = 271.6 Hz), 123.44, 118.67 – 118.27 (m). ^{19}**F NMR** (376 MHz, MeOD) δ -64.22. ^{11}**B NMR** (128 MHz, MeOD) δ -6.75. **HRMS** (ESI^+) m/z calcd for $C_{12}H_8I_2^{2+}$ $[M - 2BAr_F]^{2+}$ 202.9352, found 202.9351; and (ESI^-) m/z calcd for $C_{32}H_{12}BF_{24}^-$ $[BAr_F]^-$ 863.0654, found 863.0636.

Synthesis of *tert*-butyl (2-(tributylstannyl)phenyl)carbamate 5:

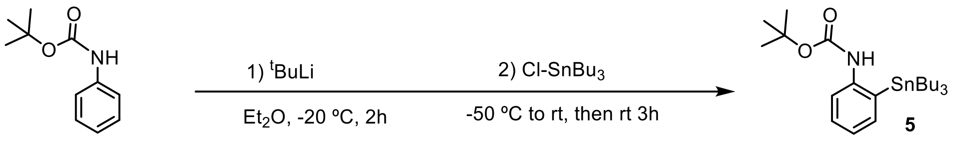

Reaction was conducted under Argon atmosphere. A flame-dried Schlenk flask equipped with a magnetic stir bar was charged with *tert*-butyl phenylcarbamate (966 mg, 5.00 mmol) and dissolved with 25 mL of dry Et_2O. The flask content was cooled to -20 °C in a dry ice/acetone bath (the dry ice was added periodically to keep that temperature with the help of a thermometer), once reached that temperature, a solution of *tert*-Butyl lithium 1.7M in pentane (7.35 mL, 12.50 mmol) was added dropwise to the flask and it was stirred 2h at -20 °C. After that time, the mixture was cooled to -50 °C and tributyltin chloride (3.4 mL, 12.50 mmol) was added dropwise. The cooling bath was removed, and the mixture was allowed to reach the room temperature. Once at that temperature, it was stirred for 3h. ~50 mL of a saturated solution was added slowly and stirred for 15 minutes. The organic layer was isolated, and the aqueous layer was extracted with Et_2O (2 x 25 mL). The combined organic phase was dried over $MgSO_4$, filtered, and concentrated under reduced pressure. The resulting oily residue was purified by flash column chromatography on silica gel using as eluent a gradient mixture of hexanes:AcOEt (1:0 to 20:1), R_f = 0.22 in 50:1 hexanes/EtOAc. Colorless oil, 2.26 g, yield: 94%.

^{1}H NMR (400 MHz, $CDCl_3$) δ 7.70 (d, J = 7.9 Hz, 1H), 7.36 – 7.27 (m, 2H), 7.07 (td, J = 7.3, 1.0 Hz, 1H), 6.28 (s, 1H), 1.55 – 1.50 (m, 6H), 1.51 (s, 9H), 1.40 – 1.28 (m, 6H), 1.15 – 1.07 (m, 6H), 0.89 (t, J = 7.3 Hz, 9H). **^{13}C NMR** (101 MHz, $CDCl_3$) δ 153.36, 143.48, 136.83, 129.19, 124.06, 121.89, 80.15, 29.05, 28.38, 27.35, 13.63, 10.03.

Synthesis of (2-bromophenyl)(hydroxy)-λ^3-iodaneyl 4-methylbenzenesulfonate 6a:

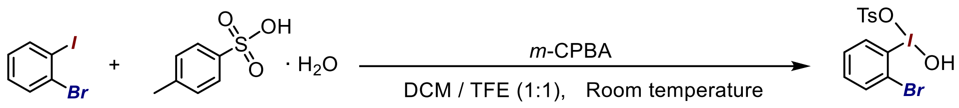

A round-bottom flask equipped with a magnetic stirbar was charged with the 1-bromo-2-iodobenzene (1415 mg, 5.00 mmol) and dissolved in 25.0 mL of the solvent mixture of dichloromethane / TFE (1:1). *m*-CPBA (1121 mg, 5.00 mmol, <77 %) was added into the flask under stirring, followed by the addition of *p*-toluenesulfonic acid monohydrate (951 mg, 5.00 mmol). After an hour of stirring at room temperature, the solvents were evaporated. The resulting yellow residue was digested with ~30 mL of a solvent mixture of $CHCl_3$ / Et_2O (1:1) under vigorous stirring for 30 min. The solid was filtrate and washed with a solvent mixture of

$CHCl_3$ / Et_2O (1:1, 2x15 mL). The white solid was further dried under high vacuum affording 2218 mg. Yield: 94%.

^{1}H NMR (400 MHz, DMSO) δ 9.99 (br s, 1H, OH), 8.49 (d, *J* = 7.7 Hz, 1H), 8.05 (d, *J* = 7.8 Hz, 1H), 7.66 (t, *J* = 7.4 Hz, 1H), 7.57 (t, *J* = 7.5 Hz, 1H), 7.47 (d, *J* = 7.8 Hz, 2H), 7.11 (d, *J* = 7.7 Hz, 2H), 2.29 (s, 3H). **^{13}C NMR** (101 MHz, DMSO) δ 145.49, 139.31, 137.75, 135.17, 133.02, 130.15, 129.80, 128.10, 126.84, 125.50, 20.80.

Synthesis of (2-chlorophenyl)(hydroxy)-λ^3-iodaneyl 4-methylbenzenesulfonate 6b:

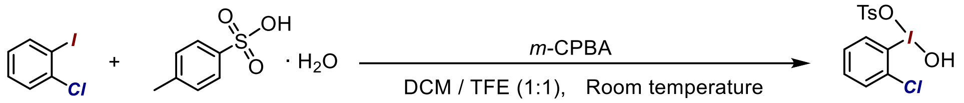

A round-bottom flask equipped with a magnetic stirbar was charged with the 1-chloro-2-iodobenzene (1192 mg, 5.00 mmol) and dissolved in 25.0 mL of the solvent mixture of dichloromethane / TFE (1:1). *m*-CPBA (1121 mg, 5.00 mmol, <77 %) was added into the flask under stirring, followed by the addition of *p*-toluenesulfonic acid monohydrate (951 mg, 5.00 mmol). After an hour of stirring at room temperature, the solvents were evaporated. The resulting yellow residue was digested with ~30 mL of a solvent mixture of $CHCl_3$ / Et_2O (1:1) under vigorous stirring for 30 min. The solid was filtrate and washed with a solvent mixture of $CHCl_3$ / Et_2O (1:1, 2x15 mL). The white solid was further dried under high vacuum affording 1998 mg. Yield: 94%.

^{1}H NMR (400 MHz, DMSO) δ 10.16 – 9.83 (br s, 1H, OH), 8.50 (d, *J* = 7.3 Hz, 1H), 7.92 (d, *J* = 7.6 Hz, 1H), 7.76 (t, *J* = 7.1 Hz, 1H), 7.54 (t, *J* = 7.2 Hz, 1H), 7.47 (d, *J* = 7.4 Hz, 2H), 7.11 (d, *J* = 7.2 Hz, 2H), 2.29 (s, 3H). **^{13}C NMR** (101 MHz, DMSO) δ 145.63, 138.98, 137.65, 135.76, 135.28, 129.75, 129.73, 128.06, 126.76, 125.49, 20.78.

Synthesis of 7a:

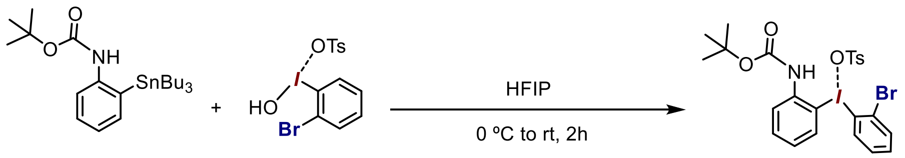

A round-bottom flask equipped with a magnetic stirbar was charged with the (2-bromophenyl)(hydroxy)-λ^3-iodaneyl 4-methylbenzenesulfonate (471 mg, 1.00 mmol) and dissolved in 10.0 mL of 1,1,1,3,3,3-Hexafluoro-2-propanol (HFIP). The content was cooled to 0 °C in an ice/water bath, followed by dropwise addition of tert-butyl (2-(tributylstannyl)phenyl)carbamate (579 mg, 1.20 mmol). The reaction was allowed to reach

room temperature, then stirred 2h at that temperature. Afterwards, the resulting mixture was concentrated under reduced pressure. ~20 mL of Et_2O / pentane (1:3) solvent mixture was added to the resulting orange oily residue and digested through vigorously stirring during 15 min. The solid was filtered and washed with a 1:3 Et_2O / pentane (3 x 15 mL) solvent mixture. The resulting white solid was dried under high vacuum affording 543 mg. Yield: 84%.

^{1}H NMR (400 MHz, CD_3OD) δ 8.39 (dd, *J* = 7.9, 1.6 Hz, 1H), 7.94 (dd, *J* = 8.0, 1.5 Hz, 1H), 7.72 – 7.68 (m, 2H), 7.68 – 7.52 (m, 4H), 7.38 (dd, *J* = 8.0, 1.6 Hz, 1H), 7.24 – 7.20 (m, 1H), 7.18 (ddd, *J* = 8.2, 7.3, 1.6 Hz, 1H), 2.36 (s, 3H), 1.60 (s, 9H). **^{13}C NMR** (101 MHz, CD_3OD) δ 158.89 (C=O), 143.56, 141.64, 140.85, 140.28, 136.22, 135.42, 135.07, 134.19, 131.73, 129.80, 129.39, 129.05, 126.96, 126.00, 123.04, 111.57 (**C**-*I*), 84.59, 28.55, 21.31. **HRMS** (ESI^+) m/z calcd for $C_{17}H_{18}BrINO_2^+$ $[M – OTs]^+$ 473.9560, found 473.9558.

Synthesis of 7b:

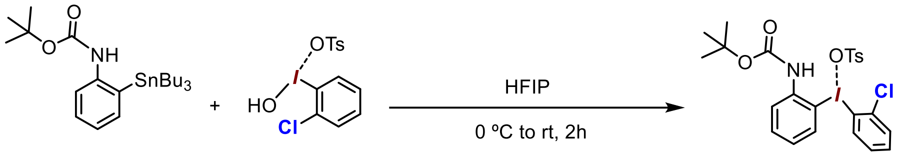

A round-bottom flask equipped with a magnetic stirbar was charged with the (2-chlorophenyl)(hydroxy)-λ^3-iodaneyl 4-methylbenzenesulfonate (427 mg, 1.00 mmol) and dissolved in 10.0 mL of 1,1,1,3,3,3-Hexafluoro-2-propanol (HFIP). The content was cooled to 0 °C in an ice/water bath, followed by dropwise addition of tert-butyl (2-(tributylstannyl)phenyl)carbamate (579 mg, 1.20 mmol). The reaction was allowed to reach room temperature, then stirred 2h at that temperature. Afterwards, the resulting mixture was concentrated under reduced pressure. ~20 mL of Et_2O / pentane (1:3) solvent mixture was added to the resulting orange oily residue and digested through vigorously stirring during 15 min. The solid was filtered and washed with a 1:3 Et_2O / pentane (3 x 15 mL) solvent mixture. The resulting white solid was dried under high vacuum affording 428 mg. Yield: 71%.

^{1}H NMR (400 MHz, DMSO-d_6) δ 9.95 (s, 1H, NH), 8.41 (d, *J* = 7.6 Hz, 1H), 7.98 (d, *J* = 7.9 Hz, 1H), 7.83 (d, *J* = 7.3 Hz, 1H), 7.72 (t, *J* = 7.6 Hz, 1H), 7.61 (t, *J* = 7.6 Hz, 1H), 7.53 – 7.44 (m, 4H), 7.25 (t, *J* = 6.9 Hz, 1H), 7.11 (d, *J* = 7.8 Hz, 2H), 2.28 (s, 3H), 1.51 (s, 9H). **^{13}C NMR** (100 MHz, DMSO-d_6) δ 154.89 (C=O), 145.73, 138.98, 138.77, 137.59, 136.03, 135.81, 134.78, 133.04, 130.48, 130.13, 128.08, 128.04, 126.61, 125.49, 119.17, 113.37 (**C**-*I*), 81.33, 27.99, 20.78. **HRMS** (ESI^+) m/z calcd for $C_{17}H_{18}BClINO_2^+$ $[M – OTs]^+$ 430.0065, found 430.0063.

Synthesis of 8a-(OTs)$_2$:

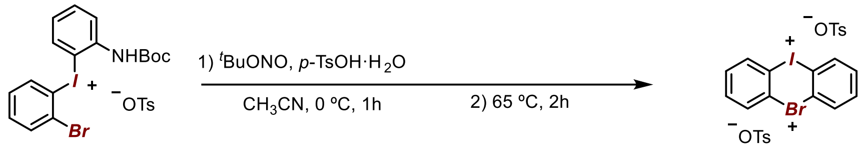

7a **8a**-(OTs)$_2$

Reaction was conducted under Argon atmosphere. In a screw-cap tube equipped with a magnetic stirbar was charged with the diaryliodonium **7a** (65 mg, 0.10 mmol) and *p*-toluenesulfonic acid monohydrate (57 mg, 0.30 mmol). The content was purged with a flux of argon during 30s. Then, 1.0 mL of dry CH_3CN was added and cooled to 0 °C in an ice/water bath. Once at 0 °C, ^{t}BuONO (24 µL, 21 mg, 0.20 mmol) was added dropwise at that temperature. The resulting mixture was stirred at 0 °C for 1h. After that time, the content was heated to 65 °C for 2h, step in which can be seen the formation of a white suspension. The tube was allowed to reach room temperature and diluted with 2.0 mL of CH_3CN. The resulting suspension was centrifugated and the supernatant liquid was decanted using a Pasteur Pipette *(This process was repeated two more times).* The resulting solid residue was dried under high vacuum to afford a white solid, 18 mg. Yield: 26%.

Characterization data:

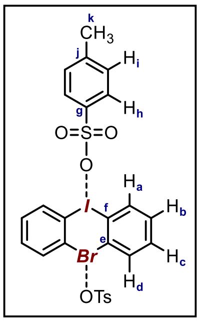

^{1}H NMR (400 MHz, D_2O) δ 8.50 (d, J = 9.2 Hz, 2H$_a$), 8.48 (d, J = 9.2 Hz, 2H$_d$), 7.96 (t, J = 7.5 Hz, 2H$_c$), 7.91 (t, J = 7.5 Hz, 2H$_b$), 7.67 (d, J = 8.1 Hz, 4H$_h$), 7.35 (d, J = 8.0 Hz, 4H$_i$), 2.38 (s, 6H$_k$). **^{13}C NMR** (101 MHz, D_2O) δ 143.10 (C$_j$), 140.12 (C$_g$), 138.58 (C$_a$), 136.14 (C$_c$), 135.83 (C$_b$), 134.73 (C$_e$-*Br*), 133.05 (C$_d$), 130.05 (C$_i$), 125.96 (C$_h$), 116.13 (C$_f$-***I***), 21.08 (C$_k$). **HRMS** (ESI$^+$) m/z calcd for $C_{19}H_{15}BrIO_3S^+$ [M – OTs]$^+$ 528.8964 / 530.8952, found 528.8974 / 528.8952.

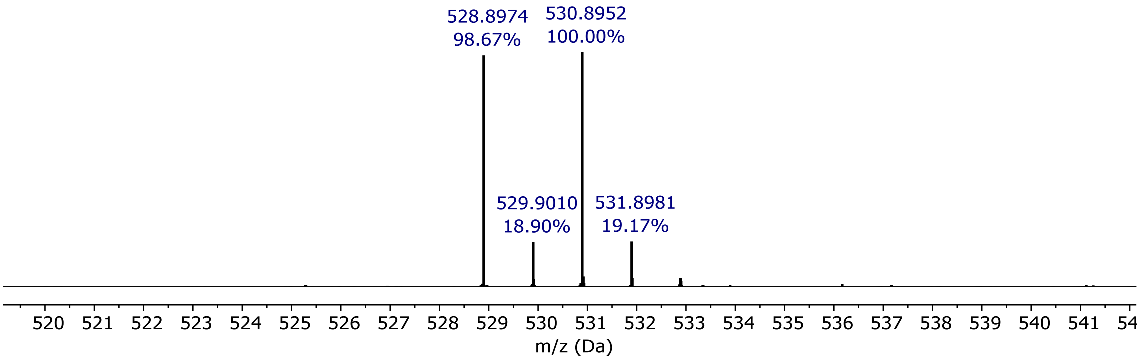

Stability of 8a-(OTs)$_2$ in D_2O:

In an NMR tube was charged with 1 mg of **8b**-(OTs)$_2$, followed by 0.6 mL of D_2O. The tube was sealed, manually shaken with 3 inversions, and directly submitted for ^{1}H-NMR analysis (Considered as t = 0h). Then, ^{1}H-NMR spectra were recorded at 1h, 3h, 4h, 2d and 20d (Figure S3).

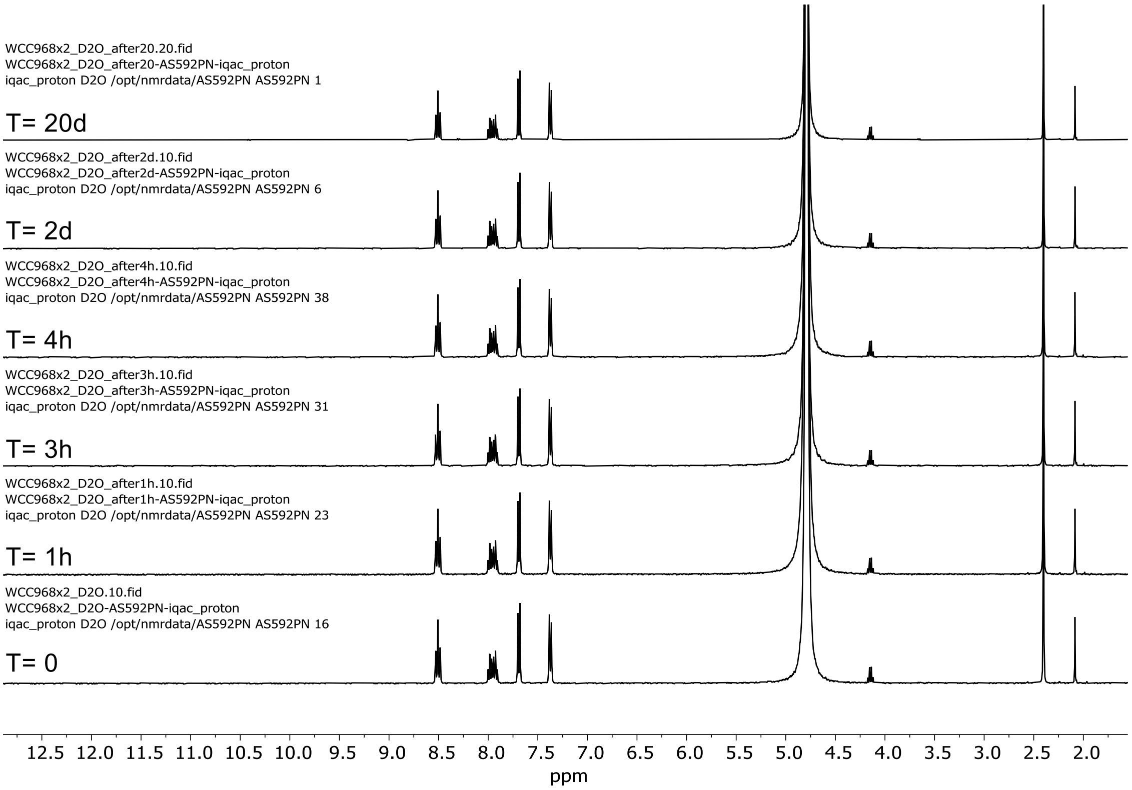

Figure S3: Stability monitorization of **8a**-(OTs)$_2$ in D_2O.

Hydrolytic ring-opening of 8a-(OTs)$_2$ in DMSO-d^6:

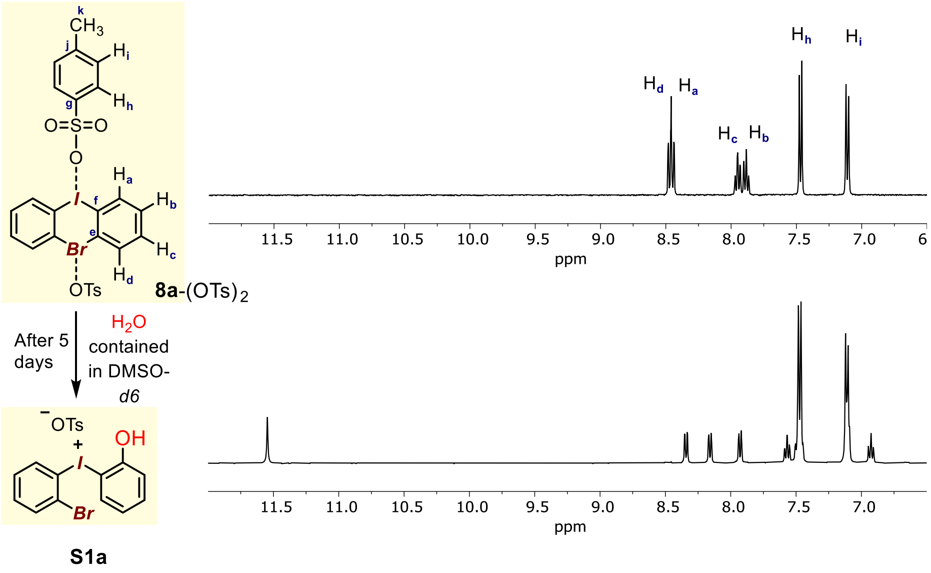

Figure S4: ^{1}H NMR spectra of **8a**-(OTs)$_2$ (upper spectra) and decomposed phenolic diaryliodonium salt **S1a** (bottom spectra) after approximately 5 days *via* the H_2O contained in the DMSO-d^6 solvent.

Characterization data of **S1a**:

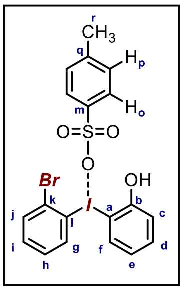

^{1}H NMR (400 MHz, DMSO-d_6) δ 11.55 (s, 1H, OH), 8.34 (dd, J = 7.9, 1.6 Hz, 1H), 8.16 (dd, J = 8.1, 1.5 Hz, 1H), 7.93 (dd, J = 8.0, 1.5 Hz, 1H), 7.57 (ddd, J = 8.0, 7.4, 1.6 Hz, 1H), 7.52 – 7.40 (m, 2H), 7.47 (d, J = 8.1 Hz, 2H), 7.11 (d, J = 7.8 Hz, 2H), 7.10 (dd, J = 8.3, 1.4 Hz, 1H), 6.93 (ddd, J = 8.1, 7.2, 1.4 Hz, 1H), 2.29 (s, 3H). ^{13}C NMR (126 MHz, DMSO-d_6) δ 156.12 (C_b), 145.73 (C_m), 139.23 (C_g), 137.62 (C_q), 137.10 (C_f), 134.82 (C_d), 134.15 (C_i), 133.51 (C_j), 130.23 (C_h), 128.07 (C_p), 126.96 (C_k-*Br*), 125.50 (C_o), 121.71 (C_e), 121.29 (C_l), 116.35 (C_c), 105.55 (C_a-***I***), 20.79 (C_r). **HRMS** (ESI$^+$) m/z calcd for $C_{12}H_9BrIO^+$ [M – OTs]$^+$ 374.8876, found 374.8874.

Synthesis of 8b-(OTs)$_2$:

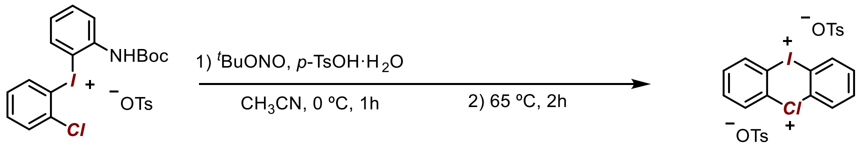

7b **8b**-(OTs)$_2$

Reaction was conducted under Argon atmosphere. In a screw-cap tube equipped with a magnetic stirbar was charged with the diaryliodonium **7b** (30 mg, 0.10 mmol) and *p*-toluenesulfonic acid monohydrate (29 mg, 0.15 mmol). The content was purged with a flux of argon during 30s. Then, 0.5 mL of dry CH_3CN was added and cooled to 0 °C in an ice/water bath. Once at 0 °C, ^{t}BuONO (13 µL, 10 mg, 0.10 mmol) was added dropwise at that temperature. The resulting mixture was stirred at 0 °C for 1h. After that time, the content was heated to 65 °C for 2h, step in which can be seen the formation of a white suspension. The tube was allowed to reach room temperature and diluted with 2.0 mL of CH_3CN. The resulting suspension was centrifugated and the supernatant liquid was decanted using a Pasteur Pipette *(This process was repeated two more times).* The resulting solid residue was dried under high vacuum to afford a white solid, 10 mg. Yield: 30%.

Characterization data:

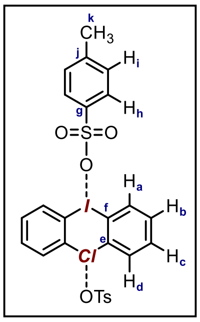

^{1}H NMR (400 MHz, D_2O) δ 8.52 (d, J = 8.2 Hz, 4H), 8.04 (t, J = 7.7 Hz, 2H), 7.96 (t, J = 7.7 Hz, 2H), 7.66 (d, J = 8.0 Hz, 4H), 7.35 (d, J = 7.9 Hz, 4H), 2.38 (s, 6H). ^{13}C NMR (101 MHz, D_2O) δ 143.10 (C_j), 139.98 (C_g), 138.60 (C_e-*Br*), 137.82 (C_a), 136.49 (C_b), 136.38 (C_c), 131.11 (C_d), 130.05 (C_i), 125.96 (C_h), 114.15 (C_f-***I***), 21.08 (C_k). **HRMS** (ESI$^+$) m/z calcd for $C_{19}H_{15}ClIO_3S^+$ [M – OTs]$^+$ 484.9470 / 486.9440, found 484.9462 / 486.9444.

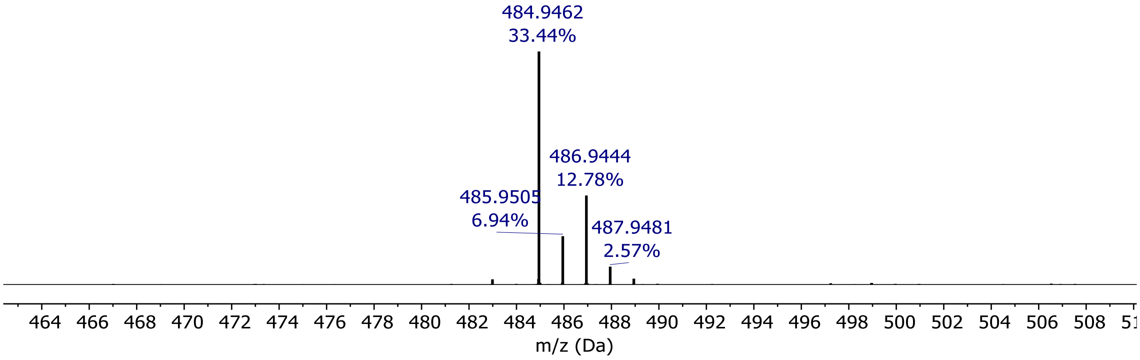

Stability of 8b-(OTs)$_2$ in D_2O:

In an NMR tube was charged with 1 mg of **8b**-(OTs)$_2$, followed by 0.6 mL of D_2O. The tube was sealed, manually shaken with 3 inversions, and directly submitted for ^{1}H-NMR analysis (Considered as t = 0h). Then, ^{1}H-NMR spectra were recorded at 1h, 17h. 2d and 20d (Figure S5).

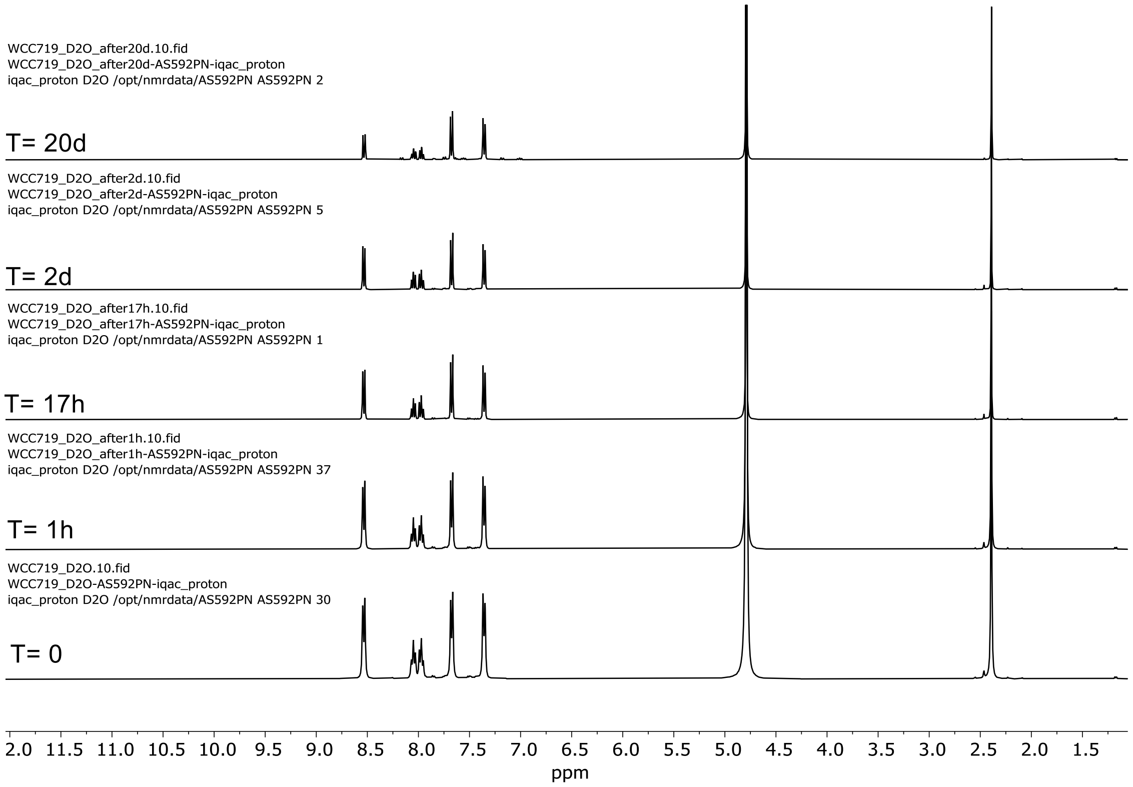

Figure S5: Stability monitorization of **8b**-$(OTs)_2$ in D_2O.

Hydrolytic ring-opening of 8b-$(OTs)_2$ in DMSO-d^6:

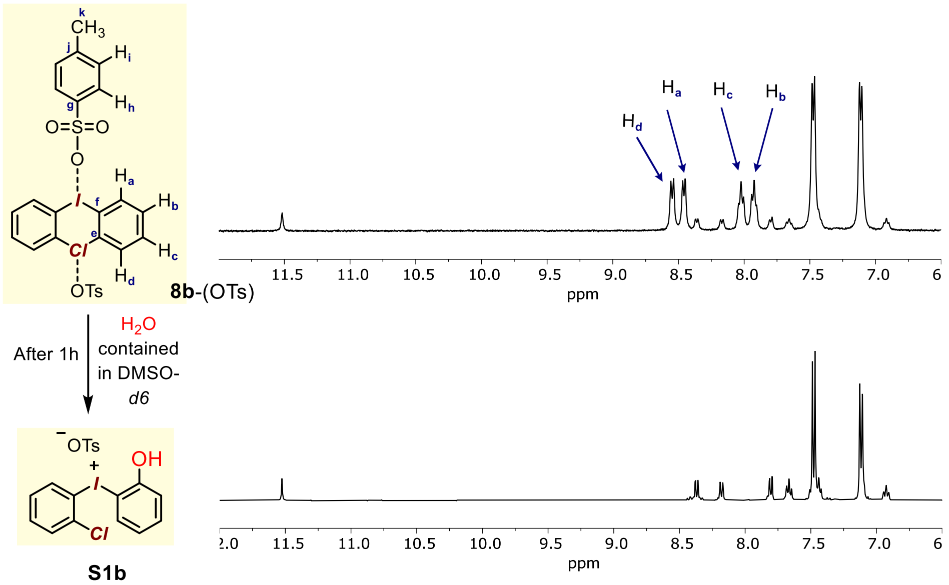

S1b

Figure S6: ^{1}H NMR spectra of **8b**-$(OTs)_2$ (upper spectra, partially decomposed to the ring-opened product **S1b**) and decomposed phenolic diaryliodonium salt **S1b** (bottom spectra) after approximately 1h *via* the H_2O contained in the DMSO-d^6 solvent.

Characterization data **S1b**:

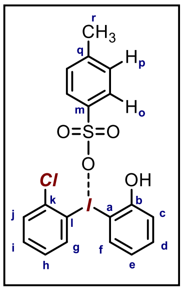

^{1}H NMR (400 MHz, DMSO-d^6) δ 11.54 (s, 1H, OH), 8.36 (d, *J* = 7.9 Hz, 1H), 8.17 (d, *J* = 7.5 Hz, 1H), 7.80 (d, *J* = 7.6 Hz, 1H), 7.66 (t, *J* = 7.1 Hz, 1H), 7.53 – 7.38 (m, 4H), 7.11 (pseudo d, *J* = 7.2 Hz, 3H), 6.92 (t, *J* = 7.7 Hz, 1H), 2.28 (s, 3H). **HRMS** (ESI$^+$) m/z calcd for $C_{12}H_9ClIO^+$ [M – OTs]$^+$ 330.9381 / 332.9352, found 330.9371 / 332.9347.

Synthesis of 1,8-diiodonaphthalene. *Following a protocol reported by Michael W. Göbel and et al.*[5]

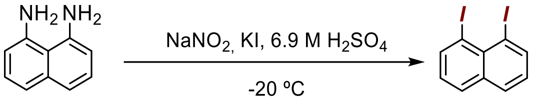

In a round bottom flask, 1,8-diaminonaphthalene (3.3 g, 21.0 mmol) was dissolved in 40 mL 6.9 M H_2SO_4 solution with vigorous stirring and cooled to -20 °C. Then, $NaNO_2$ (4.3 g, 63.0 mmol) dissolved in 17 mL H_2O, was subsequently added dropwise at -15 °C to -20 °C to the reaction mixture, followed by the addition of KI (20.9 g, 126.0 mmol) dissolved in 17 mL H_2O, at -15 °C to -20 °C. Afterwards, it was quickly heated to 80 °C for 10 minutes and allowed to reach room temperature. Then, NaOH pellets was added in small portions until neutralization of the reaction mixture. The resulting mixture was added in a separation funnel, followed by the addition of Et_2O (500 mL). The extract was successively washed with 10 % HCl solution (3x100 mL), saturated $Na_2S_2O_3$ solution (3x100 mL) and 1 M NaOH solution (3x100 mL). The combined organic phase was dried over $MgSO_4$, filtered and the solvent was removed by rota-evaporation. The resulting brown-dark residue was purified by flash column chromatography on silica gel using as eluent hexanes:AcOEt (40:1) affording a pale yellow solid (4.72 g, 60%)

^{1}H NMR (400 MHz, $CDCl_3$) δ 8.42 (dd, *J* = 7.3, 1.3 Hz, 2H), 7.84 (dd, *J* = 8.2, 1.3 Hz, 2H), 7.07 (t, *J* = 7.7 Hz, 2H).**^{13}C NMR** (101 MHz, $CDCl_3$) δ 144.18, 135.93, 132.27, 131.16, 127.08, 96.14 (C-***I***). *Data consistent with reported compound.*[5]

Synthesis of (8-iodonaphthalen-1-yl)boronic acid.

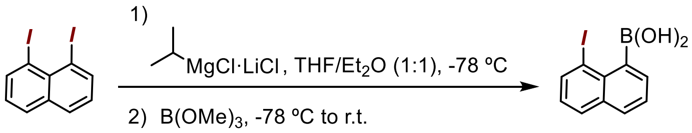

Reaction was conducted under Argon atmosphere. An oven-dried Schlenk flask equipped with a magnetic stir bar was charged with 1,8-diiodonaphthalene (1.9 g, 5.00 mmol) and dissolved with

100 mL of a mixture of dry THF-Et_2O (1:1). The flask content was cooled to -78 °C in a dry ice/acetone bath, once reached that temperature, a solution of Turbo Grignard 1.3M in THF (4.23 mL, 5.50 mmol) was added dropwise to the mixture and it was stirred 2h at -78 °C. After that time, $B(OMe)_3$ (1.7 mL, 15.0 mmol) was added in one portion to the content at -78 °C. Subsequently, the reaction mixture was allowed to reach room temperature and stirred overnight. Then, ~50 mL of an aqueous solution of HCl 10% was added and the mixture was stirred for 30 min. The resulting layers were separated, and the aqueous phase was extracted with Et_2O (3 x 50 mL). The combined organic phase was washed with brine, dried over $MgSO_4$ and filtered. After concentration in *vacuo*, the residue was purified by flash column chromatography on silica gel using as eluent a gradient mixture of hexanes:AcOEt (4:1 to 1:1), R_f = 0.13 in 4:1 hexanes/EtOAc. Pale yellow solid, 1.14 g, yield: 77%.

^{1}H NMR (400 MHz, Methanol-d_4) δ 8.17 (dd, *J* = 7.3, 1.3 Hz, 1H), 7.90 (dd, *J* = 8.2, 1.2 Hz, 1H), 7.82 (dd, *J* = 8.2, 1.4 Hz, 1H), 7.62 (dd, *J* = 6.8, 1.4 Hz, 1H), 7.48 (dd, *J* = 8.1, 6.8 Hz, 1H), 7.17 (dd, *J* = 8.1, 7.3 Hz, 1H). **^{13}C NMR** (101 MHz, Methanol-d_4) δ 140.03, 133.19, 130.78, 130.58, 127.55, 126.70, 100.24 (**C**-*I*).

Synthesis of trifluoro(8-iodonaphthalen-1-yl)-λ^4-borane, potassium salt 10:

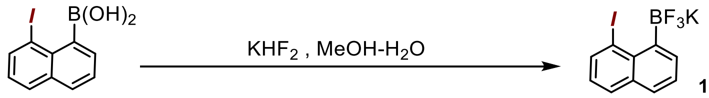

In a round-bottom flask, (8-iodonaphthalen-1-yl)boronic acid (1.0 g, 3.36 mmol) was dissolved in 7.5 mL of methanol. In a vial, potassium bifluoride (1.05 g, 13.43 mmol, 4.0 equiv) was dissolved in 6.0 mL of water and this solution was added dropwise to the round bottom flask under vigorous stirring. After two hours of stirring at room temperature, the solvents were evaporated under reduced pressure. Next, the solid residue was further dried under high vacuum for 1 hour. The flask was washed with 10 mL of acetone (GC quality) and the solution was filtered through a Pasteur Pipette with a plug of cotton (The process was repeated 4 times). The resulting solution was evaporated. The solid thus obtained was washed with Et_2O (3 x 10 mL) and decanted. The resulting solid was dried under high vacuum affording a pale-yellow solid (Quantitative yield).

^{1}H NMR (400 MHz, DMSO-d_6) δ 8.20 (dd, *J* = 7.2, 1.5 Hz, 1H), 7.91 (dd, *J* = 7.0, 1.6 Hz, 1H), 7.78 (ddd, *J* = 7.9, 1.4, 0.6 Hz, 1H), 7.59 (ddd, *J* = 7.9, 1.6, 0.5 Hz, 1H), 7.29 (ddd, *J* = 7.7, 7.0, 0.6 Hz, 1H), 6.95 (dd, *J* = 8.0, 7.2 Hz, 1H). **^{13}C NMR** (101 MHz, DMSO-d_6) δ 140.77, 138.95, 135.13, 133.57 (q, *J* = 5.7 Hz), 129.60, 127.44, 125.03, 124.72, 96.51. **^{19}F NMR** (376 MHz, DMSO-d_6) δ -122.67. **HRMS** (ESI$^+$) *m/z* calcd for $C_{12}H_{12}BINaO_2^+$ [M – 3F – K + 2CH$_3$O + Na]$^+$ 348.9867, found 348.9875.

Synthesis of 9-$(BF_4)_2$:

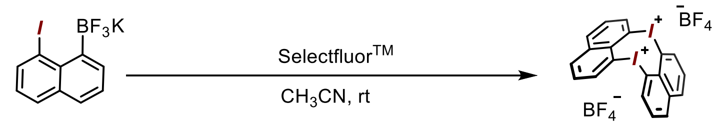

In a round-bottom flask, potassium trifluoro(8-iodonaphthalen-1-yl)borate **10** (108 mg, 0.30 mmol) was dissolved in 3.0 mL of CH_3CN. Followed by the addition of Selectfluor™ (212 mg, 0.60 mmol) in one portion at room temperature, and the resulting mixture was stirred at room temperature until fully conversion to the desired product. The progression of the reaction was done following the next protocol. *An aliquot (approximately 100 µL via syringe) of the crude mixture was concentrated under reduced pressure. The resulting solid residue was analyzed by ^{1}H-NMR in CD_3OD.* After 17 hours, the reaction shows fully consumption of the starting material and intermediates (Figure S7).

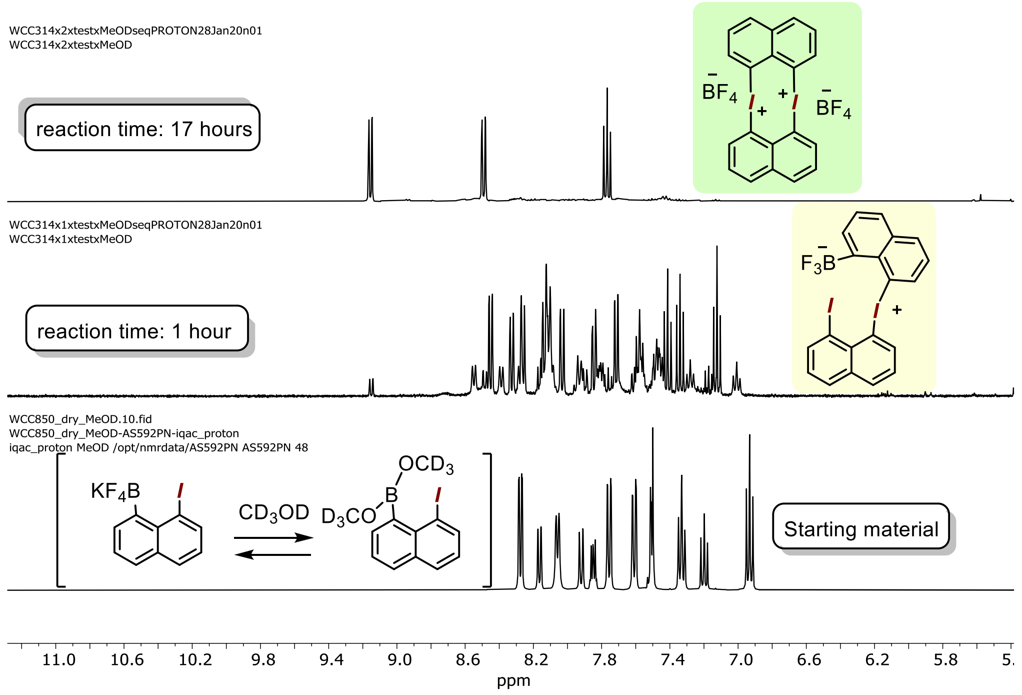

Figure S7. ^{1}H-NMR spectra in CD_3OD of the starting material **10** and crude mixture at different times. After 1 hour of reaction, and after 17 hours of reaction.

Work-up: Purification by column chromatography on reverse phase

Solvent was removed under reduced pressure by rota-evaporation. Then, 10 mL DCM was added to the solid residue and stirred vigorously for 10 minutes. The resulting suspension was introduced into an empty solid load cartridge that contained a layer of sand. Once the solid residue was deposited over the sand, an additional layer of sand was added and washed with

an additional 10 mL DCM. The clear pale-yellow filtrate (contain reduced side-products and mono-diaryliodonium salts) was discarded and the cartridge was flushed with air to dry the content. The resulting cake was purified by *CombiFlash EZ Prep,* using a Biotage C18 column of 13g. Gradient elution of H_2O / CH_3CN (0 to 30% CH_3CN, Flow Rate: 15 ml/min). The fractions containing the desired product were gathered and concentrated under reduced pressure affording a pale-yellow solid, 74 mg. Yield 73%.

^{1}H NMR (400 MHz, MeOD) δ 9.14 (dd, *J* = 7.5, 1.0 Hz, 4H, *para-H*), 8.48 (dd, *J* = 8.3, 0.9 Hz, 4H, *ortho-H*), 7.76 (t, *J* = 7.9 Hz, 4H, *meta-H*). ^{13}C NMR (101 MHz, MeOD) δ 145.36 (*para*-**C**H), 138.10 (*ortho*-**C**H*)*, 137.55, 130.59 (*meta*-**C**H), 126.58, 117.91 (**C**-*I*). **^{19}F NMR** (376 MHz, MeOD) δ -154.22. **^{11}B NMR** (128 MHz, MeOD) δ -1.11. **HRMS** (ESI$^+$) m/z calcd for $C_{21}H_{13}I_2O_2^+$ [M – 2BF$_4$ + HCOO]$^+$ 550.8999, found 550.8981.

Synthesis of 9-(OTs)$_2$:

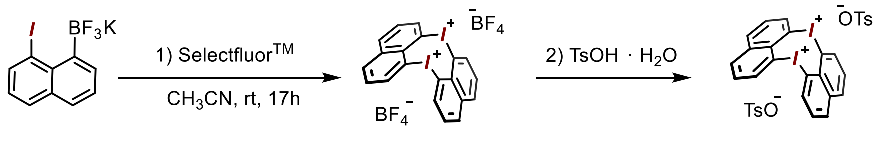

In a round-bottom flask, potassium trifluoro(8-iodonaphthalen-1-yl)borate **10** (108 mg, 0.30 mmol) was dissolved in 10 mL of CH_3CN. Followed by the addition of Selectfluor™ (212 mg, 0.60 mmol) in one portion at room temperature, and the resulting mixture was stirred at room temperature until fully conversion to the **9**-(BF$_4$)$_2$. Then, TsOH·H$_2$O (171 mg, 0.90 mmol) was added to the mixture and stirred for 10 minutes leading to the precipitation of the target product as a pale-yellow solid. The resulting suspension was introduced into a Falcon tube, subsequently centrifugated (6000 *rpm* for 60 seconds) and the supernatant liquid was extracted by Pasteur Pipette *(This process was repeated using 10 mL of CH$_3$CN).* The resulting solid residue was dried under high vacuum to afford a pale-yellow solid, 109 mg. Yield: 86%.

^{1}H NMR (400 MHz, DMSO) δ 9.15 (d, *J* = 7.4 Hz, 4H, *para-H*), 8.50 (d, *J* = 8.0 Hz, 4H, *ortho-H*), 7.78 (t, *J* = 7.8 Hz, 4H, *meta-H*), 7.47 (d, *J* = 7.8 Hz, 4H, OTs), 7.10 (d, *J* = 7.8 Hz, 4H, OTs), 2.28 (s, 6H, OTs). **^{13}C NMR** (101 MHz, DMSO) δ 145.59 (OTs), 143.47, 137.68 (OTs), 136.09, 134.47 (*meta* **C**=C*)*, 129.23 (*meta*-**C**H*)*, 128.06, 125.50, 124.86 (*ortho* **C**=C*)*, 118.27 (**C**-*I*), 20.78 (OTs). **HRMS** (ESI$^+$) m/z calcd for $C_{27}H_{19}I_2O_3S^+$ [M – OTs]$^+$ 676.9139, found 676.9139.

Synthesis of 9-(OTf)$_2$:

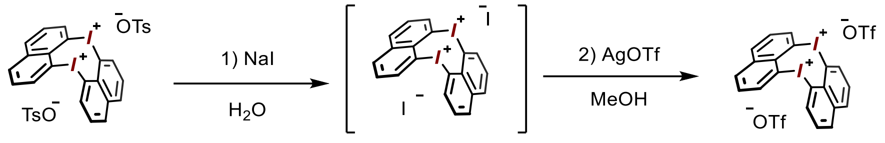

9-(OTs)$_2$ **9**-(OTf)$_2$

In a Falcon tube, **9**-(OTs)$_2$ (85 mg, 0.10 mmol) was dispersed in 5.0 mL of H_2O. Then, NaI (90 mg, 0.60 mmol, 6.0 equiv.) was introduced into the mixture leading to the precipitation of the target product in iodide form as a pale-yellow solid. The suspension was centrifugated (6000 *rpm* for 1 minute) and the supernatant liquid was extracted by Pasteur Pipette. The resulting residue was dispersed in 5.0 mL of MeOH, followed by the addition of AgOTf (62 mg, 0.24 mmol, 2.4 equiv.) and stirred 10 min. After that time, the suspension was filtered through a nylon filter of 22 µm and additional MeOH (2 x 5 mL) was used to rinse/wash the flask, as well as the filter. The filtrate was concentrated under reduced pressure affording a pale-yellow residue that was washed with dichloromethane (2 x 5 mL). The solid was dried under high vacuum to afford a pale-yellow solid. Yield: quantitative.

^{1}H NMR (400 MHz, DMSO-*d*$_6$) δ 9.15 (dd, *J* = 7.5, 1.2 Hz, 4H, *ortho-H*), 8.51 (dd, *J* = 8.2, 1.2 Hz, 4H, *para-H*), 7.79 (t, *J* = 7.8 Hz, 4H, *meta-H*). **^{13}C NMR** (101 MHz, DMSO-*d*$_6$) δ 143.47 (*ortho* **C**H*)*, 136.15 (*para* **C**H), 135.30(*meta* **C=**C*)*, 129.27 (*meta* **C**H), 124.82(*ortho* **C**=C*)*, 118.20 (**C**-*I*). **^{19}F NMR** (376 MHz, DMSO-*d*$_6$) δ -77.77 (OTf). **HRMS** (ESI$^+$) m/z calcd for $C_{21}H_{12}F_3I_2O_3S^+$ [M – OTf]$^+$ 654.8543, found 654.8549.

Synthesis of 9-(PF$_6$)$_2$:

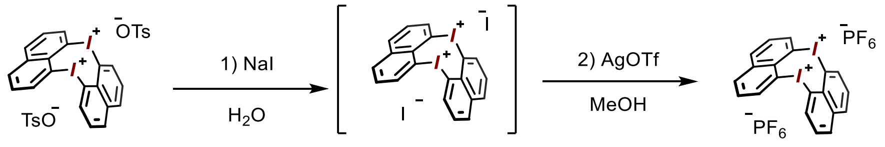

9-(OTs)$_2$ **9**-(PF$_6$)$_2$

In a Falcon tube, **9**-(OTs)$_2$ (85 mg, 0.10 mmol) was dispersed in 5.0 mL of H_2O. Then, NaI (90 mg, 0.60 mmol, 6.0 equiv.) was introduced into the mixture leading to the precipitation of the target product in iodide form as a pale-yellow solid. The suspension was centrifugated (6000 *rpm* for 1 minute) and the supernatant liquid was extracted by Pasteur Pipette. The resulting residue was dispersed in 5.0 mL of MeOH, followed by the addition of $AgPF_6$ (61 mg, 0.24 mmol, 2.4 equiv.) and stirred 10 min. After that time, the suspension was filtered through a nylon of 22 µm and additional MeOH (2 x 5 mL) was used to rinse/wash the flask, as well as the filter. The filtrate was concentrated under reduced pressure affording a pale-yellow residue that was washed with

dichloromethane (2 x 5 mL). The solid was dried under high vacuum to afford a pale-yellow solid. Yield: quantitative

^{1}H NMR (400 MHz, MeOD) δ 9.15 (dd, *J* = 7.6, 0.9 Hz, 4H), 8.48 (dd, *J* = 8.3, 1.2 Hz, 4H), 7.76 (t, *J* = 7.9 Hz, 4H). **^{13}C NMR** (101 MHz, MeOD) δ 145.44, 138.15, 137.56, 130.61, 126.55, 117.67. **^{19}F NMR** (376 MHz, MeOD) δ -74.57 (d, $J_{F\text{-}P}$ = 707.8 Hz). **^{31}P NMR** (162 MHz, MeOD) δ -144.54 (hept, $J_{P\text{-}F}$ = 707.7 Hz). **HRMS** (ESI^{+}) m/z calcd for $C_{20}H_{12}I_2^{2+}$ [M – 2PF$_6$]$^{2+}$ 252.9509, found 252.9512.

Synthesis of 9-(I)$_2$:

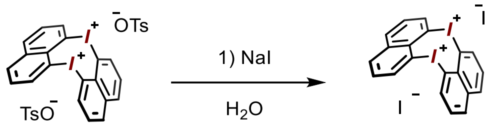

x x

In a Falcon tube, **9**-(OTs)$_2$ (42 mg, 0.05 mmol) was dissolved in 5.0 mL of H_2O. Then, NaI (75 mg, 0.50 mmol, 10.0 equiv.) was introduced into the mixture leading to the precipitation of the target product in iodide form as a pale-yellow solid and the resulting mixture was stirred in the dark for 1 h at rt. The suspension was centrifugated (6000 *rpm* for 1 minute) and the supernatant liquid was extracted by Pasteur Pipette. The resulting residue was dispersed again in 5.0 mL of H_2O, manually shaken for 1 min, centrifugated (6000 *rpm* for 1 minute) and the resulting clear liquid was decanted (*Process repeated 2 times*). The solid was dried under high vacuum to afford a pale-yellow solid. Yield: quantitative

^{1}H NMR (400 MHz, DMSO-*d*$_6$) δ 9.09 (d, *J* = 7.5 Hz, 4H), 8.44 (d, *J* = 7.5 Hz, 4H), 7.73 (t, *J* = 7.8 Hz, 4H).

Synthesis of 9-(BAr$_f$^{24})$_2$:

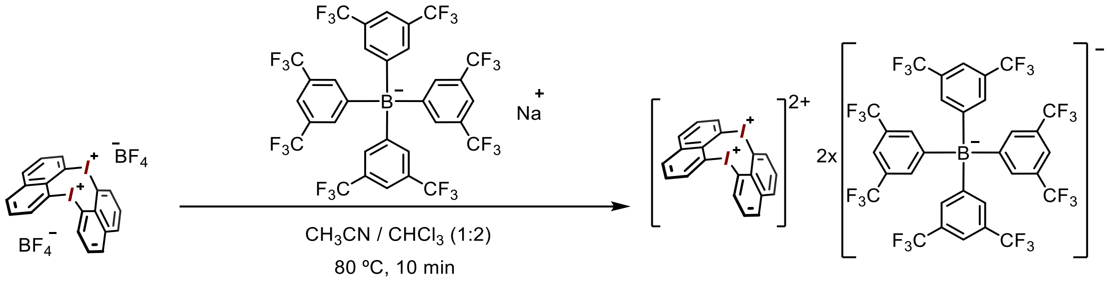

9-(BF$_4$)$_2$ **9**-(BArf^{24})$_2$

In a screw-cap tube was charged with **9**-(BF$_4$)$_2$ (68 mg, 0.10 mmol) and sodium tetrakis[3,5-bis(trifluoromethyl)phenyl]borate (NaBAr$_F$, 178 mg, 0.20 mmol), followed by the addition of 2.0 mL of CH_3CN. The pale-yellow mixture was stirred for 5 minutes at room temperature and 4 mL $CHCl_3$ was added. The tube was sealed and stirred at 80 °C for 10 min. After that time, the pale-

brown mixture was allowed to cool down to room temperature and concentrated to dryness under reduced pressure. Then, DCM (10 mL) was added to the resulting residue, manually shaken, and filtered through a nylon filter of 22µm. The filtrate was concentrated under reduced pressure affording a brown oil, that was digested with dry $CHCl_3$ (0.5 mL) under vigorous stirring for 5 min. The mixture was allowed to settle in which a brown oil was deposited on the bottom of the flask and the clear supernatant was discarded. *(This washing process was repeated 3 times)* The brown oil was dried under reduced pressure, observing a bubbling from the oil and the subsequent solidification of the oily residue to afford a pale-brown solid. This solid was further dried under high vacuum affording 150 mg. Yield: 67%.

^{1}H NMR (400 MHz, MeOD) δ 9.13 (dd, *J* = 7.6, 0.9 Hz, 4H), 8.46 (dd, *J* = 8.3, 0.9 Hz, 4H), 7.75 (t, *J* = 7.9 Hz, 4H), 7.61 – 7.59 (m, 16H), 7.57 (s, 8H). **^{13}C NMR** (101 MHz, MeOD) δ 162.85 (q, $J_{C\text{-}B}$ = 50.0 Hz), 145.34, 138.19, 137.58, 135.79 (m), 130.57, 130.41 (qq, $J_{C\text{-}F}$ = 31.8 Hz, $J_{C\text{-}B}$ = 2.9 Hz), 126.59, 125.74 (q, $J_{C\text{-}F}$ = 271.8Hz), 118.58 – 118.30 (m), 117.68. **^{19}F NMR** (376 MHz, MeOD) δ -64.24. **^{11}B NMR** (128 MHz, MeOD) δ -6.75. **HRMS** (ESI^+) m/z calcd for $C_{52}H_{24}BF_{24}I_2^+$ [M – BAr_F]$^+$ 1368.9672, found 1368.9662; and (ESI^-) m/z calcd for $C_{32}H_{12}BF_{24}^-$ [BAr_F]$^-$ 863.0654, found 863.0636.

Synthesis of perylene:

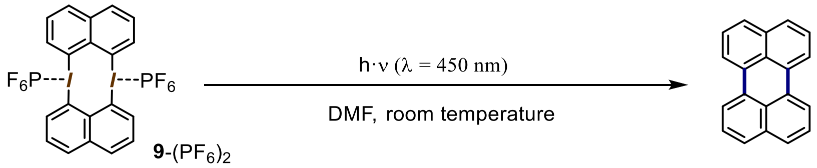

9-$(PF_6)_2$

In an oven-dried screw-cap tube was charged with the **9**-$(PF_6)_2$ (80 mg, 0.10 mmol). The content was evacuated under high vacuum and backfilled with argon (x3 cycles). Dry and degassed DMF (5.0 mL, 0.02M) was added under a flow of argon. The cap where is located the septum was covered with additional parafilm, and the resulting mixture was irradiated at λ=450 nm (50% intensity) and at room temperature for 20 hours (using the *Penn Photoreactor*). After this time, 1,3,5-trimethoxybenzene (8.4 mg, 0.05 mmols) was added as internal standard. The content was shaken manually ensuring a homogenous mixing. Then, an aliquot of the crude mixture was analyzed by ^{1}H-NMR in $CDCl_3$*: the spectrum indicated a yield of ~65%.* The crude mixture was diluted with 80 mL of Et_2O and washed with H_2O (4 x 20 mL). The organic layer was dried over Na_2SO_4 and filtered. After concentration in *vacuo*, the residue was purified by flash column chromatography on silica gel using as eluent a mixture of hexanes:AcOEt (30:1), R_f = 0.33 in 30:1 hexanes/EtOAc. Yellow solid, 23 mg *(The compound was isolated with the 1,3,5-*

trimethoxybenzene as an inseparable mixture, ratio perylene:1,3,5-trimethoxybenzene (1.00:0.50), which gives a theorical yield of 69%).

Even though, the compound of interest is contaminated with 1,3,5-trimethoxybenzene, the peaks of perylene are enough separated from the contamination and could be identified without problems.

^{1}H NMR (400 MHz, $CDCl_3$) δ 8.19 (d, J = 7.5 Hz, 4H), 7.68 (d, J = 8.0 Hz, 4H), 7.48 (t, J = 7.8 Hz, 4H). *Data consistent with reported compound.*[6]

Synthesis of (9,9-dimethyl-9H-xanthene-4,5-diyl)bis(trimethylsilane) 11:

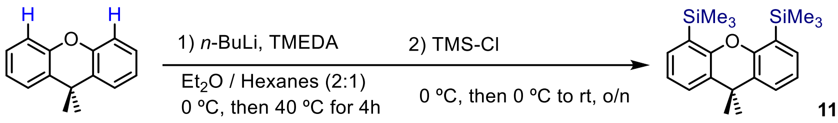

The synthesis was performed following a procedure outlined by Saito and coworkers.[7] A 100 mL oven-dried Schlenk tube was allowed to cool under Ar atmosphere. The tube was briefly opened to introduce the solid 9,9-dimethylxanthene (1.68 g, 8.00 mmol), and the inert atmosphere was re-established by applying Ar via 3 vacuum/refill cycles. Et_2O (anhydrous, 24 mL) was added causing the solid to dissolve. Next, hexane (anhydrous, 12 mL) was added, followed by TMEDA (1.86 g, 16.0, 2.4 mL) and the mixture was cooled to 0 °C. At this point, *n*-BuLi (2.5 M in Hexane, 7.7 mL, 19.2 mmol) was allowed dropwise over a ~5 min timespan leading to the formation of an orange solution. Cold bath was removed, the mixture was allowed to reach room temperature and then heated to 40 °C for 4h which led to the formation of a white precipitate. The mixture was once again cooled to 0 °C and TMSCl (2.44 mL, 2.086 g, 19.2 mmol) was added dropwise. The mixture was left in the same ice bath without refilling, thus slowly reaching room and then stirring at room temperature overnight. At this stage, the GC-MS analysis shows the target bis-trimethylsilane derivative as the main product (*m/z* = 354). Water (40 mL) was added, the mixture was stirred for 15 min and then extracted with Et_2O (3 x 30 mL). The organic fractions were combined and dried with $MgSO_4$ (anhyd). Solvent removal initially led to a colorless oil which gradually solidified. Product was purified by column chromatography on silica with neat hexanes (R_f = 0.51). The product was dried on rotary evaporator at ~20 mbar (which is max vacuum with our pump) @65 °C for 4h. Product was obtained as colorless oil, which overtime solidified into colorless crystalline solid. Yield: 2.682 g, 95%.

^{1}H NMR (400 MHz, $CDCl_3$) δ 7.47 (dd, *J* = 7.6, 1.8 Hz, 2H), 7.38 (dd, *J* = 7.2, 1.8 Hz, 2H), 7.09 (t, *J* = 7.4 Hz, 2H), 1.62 (s, 6H, CMe_2), 0.42 (s, 18H, $SiMe_3$). The analytical data are consistent with the reported literature.[7]

Synthesis of (5-iodo-9,9-dimethyl-9H-xanthen-4-yl)trimethylsilane 12:

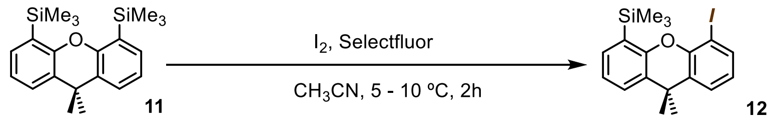

Reaction was conducted under Argon atmosphere. An oven-dried Schlenk flask equipped with a magnetic stir bar was charged with (9,9-dimethyl-9H-xanthene-4,5-diyl)*bis*(trimethylsilane) **11** (709 mg, 2.00 mmol). The flask was evacuated under high vacuum and backfilled with argon (x3 times). Then, 25 ml of dry CH_3CN was added and stirred approximately 10 minutes until complete dissolution of the starting material. Followed, by the addition of I_2 (254 mg, 1.00 mmol) in one portion and stirred for an additional 20 minutes at room temperature. After that time, the mixture was cooled between 5 to 10 °C in an ice-water bath using a thermometer as an indicator. Once a that temperature, Selectfluor™ (531 mg, 1.50 mmol) was added into the mixture and stirred for 2 hours at that temperature. Then, the mixture was allowed to reach room temperature and stirred overnight. A spoon of silica gel was added and concentrated under reduced pressure to dryness. The resulting cake was purified by flash column chromatography on silica gel using hexanes as eluent, R_f = 0.50 in hexanes. White solid, 666 mg *(contaminated with 10% of (9,9-dimethyl-9H-xanthen-4-yl)trimethylsilane)*, yield: 77%.

^{1}H NMR (400 MHz, $CDCl_3$) δ 7.72 (dd, *J* = 7.7, 1.3 Hz, 1H), 7.45 (dd, *J* = 7.8, 1.5 Hz, 1H), 7.40 (dd, *J* = 7.8, 1.3 Hz, 1H), 7.36 (dd, *J* = 7.1, 1.5 Hz, 1H), 7.12 (t, *J* = 7.4 Hz, 1H), 6.82 (t, *J* = 7.8 Hz, 1H), 1.64 (s, 6H), 0.54 (s, 9H). **^{13}C NMR** (101 MHz, $CDCl_3$) δ 154.68, 150.18, 138.35, 133.73, 131.39, 128.84, 127.85, 127.28, 126.64, 124.62, 123.52, 83.76 (C-***I***), 34.82, 33.13, 0.09 (TMS).

Synthesis of 14-(BF_4)$_2$:

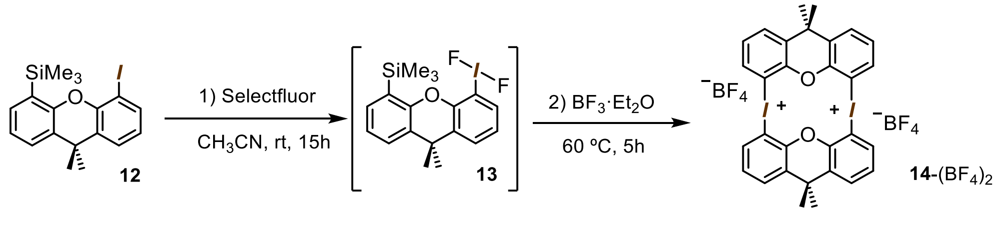

In an oven-dried Schlenk flask was charged with (5-iodo-9,9-dimethyl-9H-xanthen-4-yl)trimethylsilane **12** (61 mg, 0.15 mmol) and Selectfluor™ (106 mg, 0.30 mmol). The content was evacuated under high vacuum and backfilled with argon (x3 times). Then, 1.5 ml of dry CH_3CN was added and stirred for 15 hours at room temperature. After that time, an aliquot of the crude mixture was analyzed by ^{1}H-NMR in DMSO-d$_6$, which led to the formation of the intermediate **13** (Check below Figure S8, *NMR spectrum B*). Upon formation of the new

intermediate, $BF_3 \cdot Et_2O$ (19 µL, 0.20 mmol) was added *via* syringe under a flow of argon. The content was heated at 60 °C and stirred for 6 hours (Approximately time required to fully promote the formation of the xanthene angle bar based **14**-$(BF_4)_2$ (check figure S8, *NMR spectrum C and D*)).

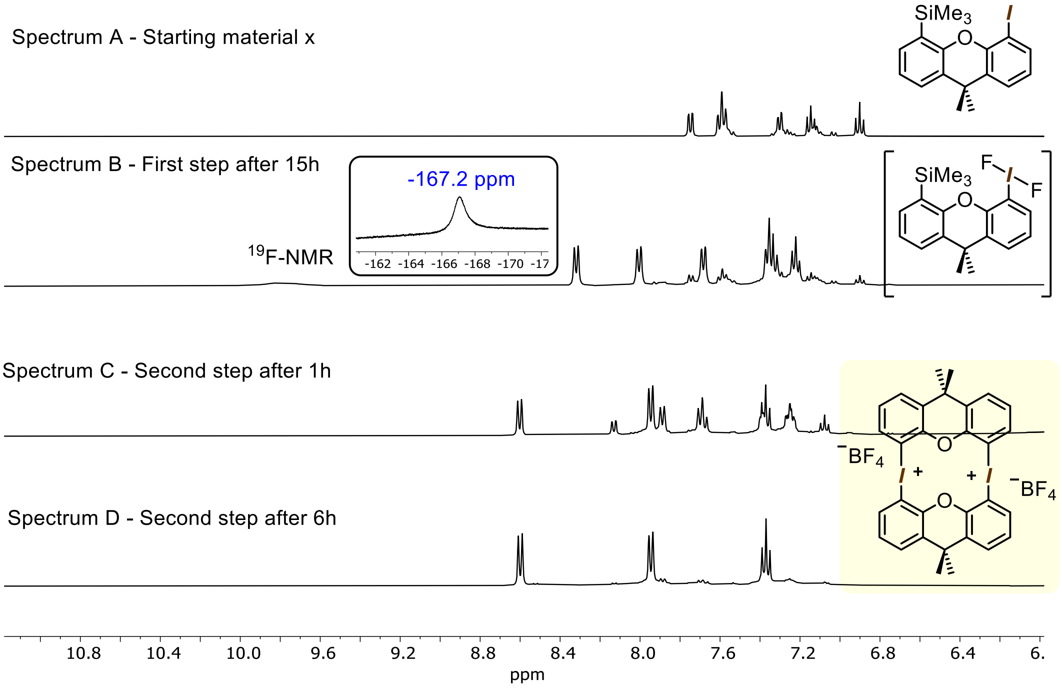

Figure S8. Comparison ^{1}H-NMR spectra at different stages of the reaction analyzed in DMSO-d$_6$: *Spectrum A*, starting material **12**; *Spectrum B*, Crude mixture at first step of the reaction after 15 hours; *Spectrum C and D:* crude mixture at second step of the reaction after 1 and 6 hours, respectively.

The reaction mixture was allowed to reach room temperature, transferred into a round-bottom flask, followed by the addition of a spoon of reversed phase silica gel C18 (100Å) and concentrated to dryness under reduced pressure. The resulting cake was purified by *CombiFlash EZ Prep,* using a Biotage C18 column of 13g. Gradient elution of H_2O / CH_3CN (0 to 30% CH_3CN, Flow Rate: 15 ml/min). The fractions containing the desired product were gathered, concentrated under reduced pressure, and dried under high vacuum affording a pale-yellow solid, 25 mg. Yield 40%.

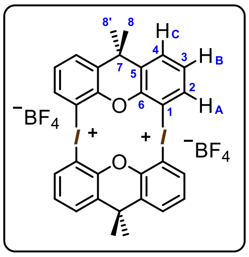

^{1}H NMR (400 MHz, Methanol-d_4) δ 8.46 (dd, J = 8.0, 1.4 Hz, 4H, H_A), 7.92 (dd, J = 7.9, 1.4 Hz, 4H, H_C), 7.37 (t, J = 7.9 Hz, 4H, H_B), 1.67 (s, 6H, *diasterotopic* CH_3), 1.66 (s, 6H, *diasterotopic* CH_3). **^{13}C NMR** (101 MHz, Methanol-d_4) δ 148.40 (C_6), 137.28 (C_2), 134.19 (C_4), 133.76 (C_5), 128.95 (C_3), 105.65 (C_1-***I***), 37.28 (C_7), 34.32 (*diasterotopic* CH_3), 31.65 (*diasterotopic* CH_3). **^{19}F NMR** (376 MHz, Methanol-d_4) δ -154.33. **HRMS** (ESI$^+$) m/z calcd for $C_{31}H_{25}I_2O_4^+$ [M – 2BF$_4$ + HCOO]$^+$ 714.9837, found 714.9847.

Synthesis of 14-(OTf)$_2$:

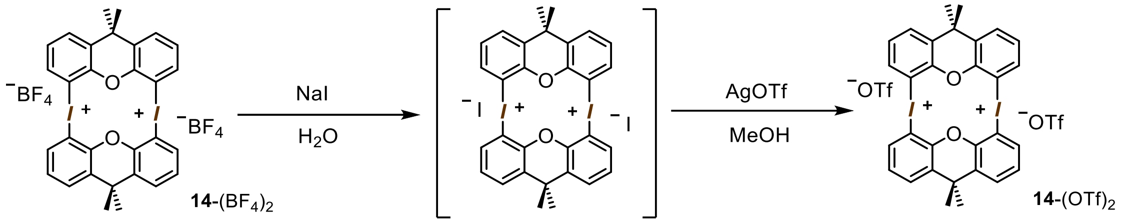

In a Falcon tube, **14**-(BF$_4$)$_2$ (42 mg, 0.05 mmol) was dispersed in 5.0 mL of H_2O. Then, NaI (60 mg, 0.40 mmol, 8.0 equiv.) was introduced into the mixture and stirred for 30 min leading to the precipitation of the target product in iodide form as a pale-yellow solid. The suspension was centrifugated (6000 *rpm* for 1 minute) and the supernatant liquid was extracted by Pasteur Pipette. The resulting residue was dispersed in 5.0 mL of MeOH, followed by the addition of AgOTf (28 mg, 0.11 mmol, 2.2 equiv.) and stirred 30 min. After that time, the suspension was filtered through a nylon filter of 22 μm and additional MeOH (2 x 5 mL) was used to rinse/wash the flask, as well as the filter. The filtrate was concentrated under reduced pressure affording a pale-yellow residue that was washed with $CHCl_3$ / Et_2O (1:1, 2 x 5 mL). The solid was dried under high vacuum to afford a pale-yellow solid. Yield: quantitative.

^{1}H NMR (400 MHz, MeOD) δ 8.46 (dd, J = 8.0, 1.3 Hz, 4H), 7.92 (dd, J = 7.9, 1.2 Hz, 4H), 7.37 (t, J = 7.9 Hz, 4H), 1.67 (s, 6H), 1.66 (s, 6H). **^{13}C NMR** (101 MHz, MeOD) δ 148.40, 137.27, 134.22, 133.78, 128.96, 105.64 (C-***I***), 37.29, 34.37 (*diasterotopic* CH_3), 31.62 (*diasterotopic* CH_3). **^{19}F NMR** (376 MHz, MeOD) δ -80.07 (OTf).

Synthesis of 1,2-bis(pyridin-2-ylethynyl)benzene L1:

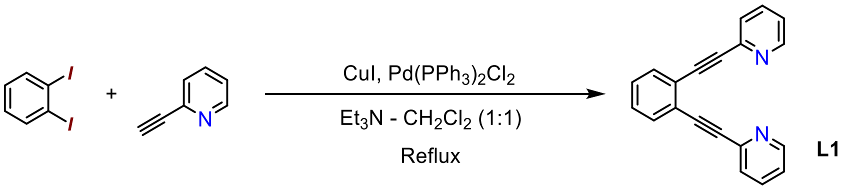

The synthesis was performed following a modified protocol reported by Madsen and coworkers.[9] *Reaction was conducted under Argon atmosphere*. A flame-dried Schlenk flask equipped with a magnetic stir bar was charged with CuI (17 mg, 0.04 mmol) and $Pd(PPh_3)_2Cl_2$ (63 mg, 0.04 mmol). The content was evacuated under high vacuum and backfilled with argon (x3). Then, 1,2-diiodobenzene (295 µL, 743 mg, 2.25 mmol) was added *via* syringe under a flow of argon, followed by the addition of 18 mL DCM (dry and degassed) and the mixture was stirred 5 min. Next, 2-ethynylpyridine (500 µL, 510 mg, 4.95 mmol) was added *via* syringe, followed by 18 mL of Et_3N (degassed). The flask was sealed and stirred at reflux (~70 °C) for16 hours. The content was allowed to reach the room temperature and concentrated to dryness under reduced pressure. The resulting solid residue was dissolved in 30 mL EtOAc and 30 mL H_2O, the organic layer was separated, and the aqueous layer was extracted with EtOAc (2 x 30 mL). Combined organic phase was dried over $MgSO_4$, filtered, and concentrated under reduced pressure. The resulting residue was purified by flash column chromatography on silica gel using as eluent a mixture of Hexanes / EtOAc (1:1), R_f = 0.20 in Hexanes / EtOAc (1:1). Brown crystalline solid, 582 mg, yield: 92%.

^{1}H NMR (400 MHz, CD_2Cl_2) δ 8.61 (ddd, *J* = 4.8, 1.7, 0.9 Hz, 1H), 7.84 – 7.79 (m, 1H), 7.71 (td, *J* = 7.7, 1.8 Hz, 1H), 7.62 (dd, *J* = 7.8, 1.6 Hz, 1H), 7.55 (dt, *J* = 7.8, 1.1 Hz, 1H), 7.42 (t, *J* = 7.8 Hz, 0H), 7.28 (ddd, *J* = 7.6, 4.9, 1.2 Hz, 1H). The analytical data are consistent with the reported literature.[8]

Synthesis of 1,3-bis(pyridin-2-ylethynyl)benzene L2:

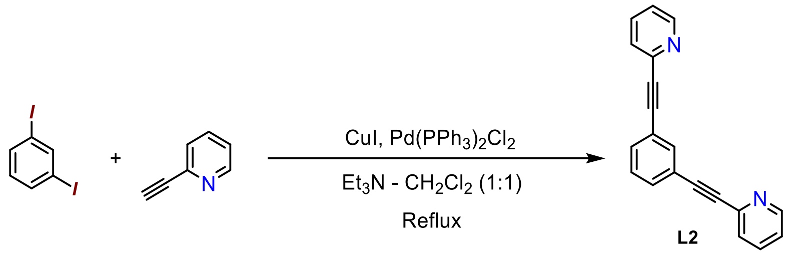

The synthesis was performed following a protocol reported by Madsen and coworkers.[9] *Reaction was conducted under Argon atmosphere*. A flame-dried Schlenk flask equipped with a magnetic stir bar was charged with CuI (17 mg, 0.04 mmol) and $Pd(PPh_3)_2Cl_2$ (63 mg, 0.04 mmol). The content was evacuated under high vacuum and backfilled with argon (x3). Then, 1,3-diiodobenzene (743 mg, 2.25 mmol) was added, followed by the addition of 18 mL DCM (dry and degassed) and the mixture was stirred 5 min. Next, 2-ethynylpyridine (500 µL, 510 mg, 4.95 mmol) was added *via* syringe, followed by 18 mL of Et_3N (degassed). The flask was sealed and stirred at reflux (~70 °C) for16 hours. The content was allowed to reach the room temperature

and concentrated to dryness under reduced pressure. The resulting solid residue was dissolved in 30 mL EtOAc and 30 mL H_2O, the organic layer was separated, and the aqueous layer was extracted with EtOAc (2 x 30 mL). Combined organic phase was dried over $MgSO_4$, filtered, and concentrated under reduced pressure. The resulting residue was purified by flash column chromatography on silica gel using as eluent a mixture of Hexanes / EtOAc (1:1), R_f = 0.20 in Hexanes / EtOAc (1:1). Yellow crystalline solid, 597 mg, yield: 95%.

^1H NMR (400 MHz, $CDCl_3$) δ 8.65 (d, *J* = 4.7 Hz, 2H), 7.72 – 7.63 (m, 6H), 7.37 (dd, *J* = 5.8, 3.3 Hz, 2H), 7.25 (dt, *J* = 6.9, 2.7 Hz, 2H). The analytical data are consistent with the reported literature.[9]

Synthesis of 4,6-diiododibenzo[*b,d*]furan:

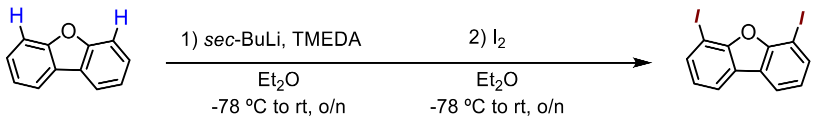

The synthesis was performed following a protocol reported by Zhang and Diver.[10] A 100 mL oven-dried Schlenk tube was allowed to cool under Ar atmosphere. The tube was charged with the solid dibenzo[*b,d*]furan (505 mg, 3.00 mmol) and TMEDA (1.05 g, 9.00 mmol, 1.35 mL). Et_2O (anhydrous, 30 mL) was added, and the mixture was cooled to -78 °C. At this point, *sec*-BuLi (1.4 M in cyclohexane, 8.6 mL, 12 mmol) was added dropwise over a ~5 min timespan. The flask was sealed and stirred overnight at room temperature. Next, the resulting yellow suspension was cooled again to -78 °C and I_2 (2.7 g, 10.50 mmol) was added, followed by the addition of additional Et_2O (anhydrous, 15 mL). The mixture was left in the same ice bath without refilling, thus slowly reaching room and then stirring at room temperature overnight. The reaction was slowly quenched with a saturated solution of $NaHSO_3$ (ca. 20 mL). Then, the organic layer was separated, and the aqueous layer was extracted with Et_2O (3 x 20 mL). The combined organic layer was dried over $MgSO_4$, filtered, and concentrated under reduced pressure. The resulting solid residue was recrystallized from a mixture of Hexanes / EtOAc (20 %, 20 mL) affording a colorless crystalline solid. Yield: 756 mg, 65%.

^1H NMR (400 MHz, $CDCl_3$) δ 7.88 (dd, *J* = 7.7, 1.1 Hz, 2H), 7.85 (dd, *J* = 7.8, 1.1 Hz, 2H), 7.13 (t, *J* = 7.7 Hz, 2H). The analytical data are consistent with the reported literature.[10]

Synthesis of 4,6-bis(pyridin-3-ylethynyl)dibenzo[*b,d*]furan L3:

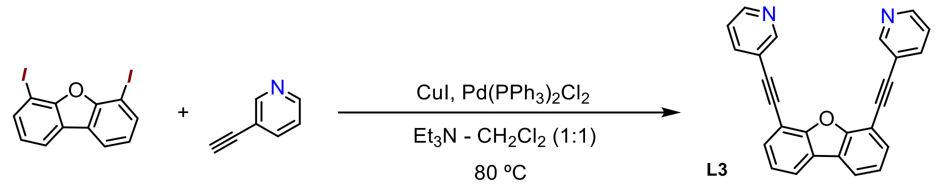

Reaction was conducted under Argon atmosphere. A flame-dried Schlenk flask equipped with a magnetic stir bar was charged with CuI (8 mg, 0.04 mmol), $Pd(PPh_3)_2Cl_2$ (28 mg, 0.04 mmol) and 4,6-diiododibenzo[*b,d*]furan (420 mg, 1.00 mmol). The content was evacuated under high vacuum and backfilled with argon (x3). Then, 3-ethynylpyridine (226 mg, 2.20 mmol) was added under argon atmosphere, followed by 8 mL of DCM (dry and degassed) and 8 mL of Et_3N (degassed). The flask was sealed and stirred at 80 °C for16 hours. The content was allowed to reach the room temperature and concentrated to dryness under reduced pressure. The resulting solid residue was dissolved in 50 mL EtOAc and 50 mL of a saturated solution of NH_4Cl (aq), then it was stirred vigorously for 5 minutes. The organic layer was separated, and the aqueous layer was extracted with EtOAc (2 x 50 mL). Combined organic phase was dried over $MgSO_4$, filtered, and concentrated under reduced pressure. The resulting residue was purified by flash column chromatography on silica gel using as eluent a mixture of DCM / MeOH (20:1), R_f = 0.30 in DCM / MeOH (20:1). Yellow solid, 352 mg, yield: 95%.

^{1}H NMR (400 MHz, Acetone) δ 8.87 (broad s, 1H), 8.63 (broad d, *J* = 4.0 Hz, 1H), 8.22 (dd, *J* = 7.8, 1.1 Hz, 1H), 8.03 (dt, *J* = 7.9, 1.9 Hz, 1H), 7.77 (dd, *J* = 7.6, 1.1 Hz, 1H), 7.51 (t, *J* = 7.7 Hz, 1H), 7.48 (dd, *J* = 7.7, 4.7 Hz, 1H). ^{13}C NMR (101 MHz, Acetone) δ 156.68, 152.80, 150.18, 139.25, 131.82, 125.14, 124.60, 124.39, 123.13, 120.74, 108.18, 92.17, 87.19. **HRMS** (ESI^+) m/z calcd for $C_{26}H_{15}N_2O^+$ $[M + H]^+$ 371.1179, found 371.1168.

Synthesis of 1·L3-(OTf)₂:

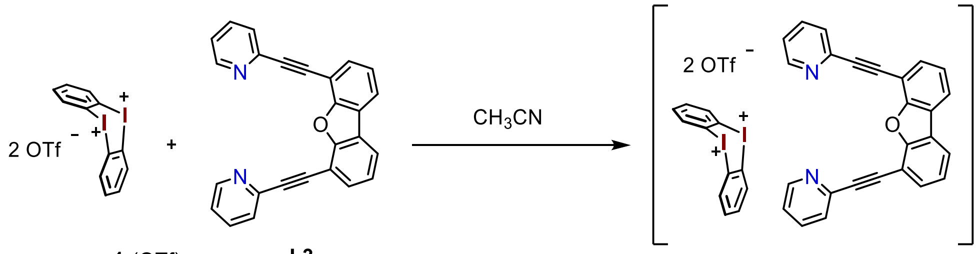

1-(OTf)₂ **L3** **1·L3**-(OTf)₂

A vial was charged with the **1**-$(OTf)_2$ (17 mg, 0.025 mmol) and **L3** (18 mg, 0.05 mmol). CH_3CN (6 mL) was added, and the resulting suspension was gently heated with a gun-heat until complete dissolution of solids. At this point, while the solution still warm (~40 to 50 °C) it was filtered into a screw-cap tube using a nylon filter of 22 µm. The tube was sealed and placed in a

dark place at room temperature. After one day, colourless crystalline crystals appeared sticked in the walls of the glass. *The crystals obtained were suitable for X-Ray diffraction.* The supernatant was decanted, and crystals were rinsed with 2 mL CH_3CN. The remaining crystals were dried under high vacuum. Colourless crystals, 20 mg. Yield: 74%.

^{1}H NMR (400 MHz, DMSO) δ 8.86 (s, 2H), 8.65 (d, *J* = 4.2 Hz, 2H), 8.47 (dd, *J* = 5.9, 3.5 Hz, 4H), 8.32 (d, *J* = 7.7 Hz, 2H), 8.08 (d, *J* = 7.8 Hz, 2H), 7.89 – 7.75 (m, 6H), 7.58 – 7.46 (m, 4H). **^{13}C NMR** (101 MHz, DMSO) δ 155.11, 151.61, 149.48, 138.67, 137.48, 133.81, 131.11, 124.07, 124.01, 123.83, 123.79, 122.91, 119.08, 106.46, 91.43, 86.43. **^{19}F NMR** (376 MHz, DMSO) δ -77.74. **CHNS [%]:** calc C 44.71, H 2.06, N 2.61, S 5.97, I 23.62; found C 44.83, H 2.02, N 2.72, S 5.92, I 23.84.

Crystal growth of 1-$(OTf)_2$, 9-$(OTf)_2$ and 14-$(OTf)_2$.

Crystals from these compounds were obtained by slow diffusion. A small vial was charged with 5 mg of the corresponding "Angle Bar" and dissolved in 1.0 mL of CH_3CN. The solution was filtered into a 1.5 mL screw-cap vial using a nylon filter of 22 µm. Then, 1.5 mL screw-cap vial was placed in a 15 mL vial that contained 3 mL Et_2O. The outer vial was sealed, placed in a dark and stored until suitable crystals were formed.

Crystal growth of 8a-$(OTs)_2$ and 8b-$(OTs)_2$.

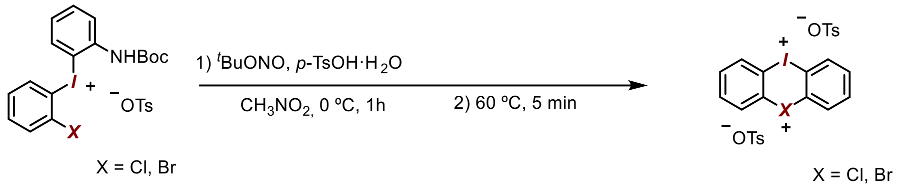

Crystals from these compounds were obtained by growing the crystals in the same reaction media. In a screw-cap tube equipped with a magnetic stirbar was charged with the diaryliodonium **x** (8 mg, 0.0125 mmol) and *p*-toluenesulfonic acid monohydrate (7 mg, 0.038 mmol). The content was purged with a flux of argon during 30s. Then, 2.0 mL of CH_3NO_2 was added and cooled to 0 °C in an ice/water bath. Once at 0 °C, ^{t}BuONO (3.3 µL, 3 mg, 0.025 mmol) was added dropwise at that temperature. The resulting mixture was stirred at 0 °C for 1h. After that time, the content was heated to 60 °C for 5 minutes *(at this point the solution still clear transparent)*. Then, the screw-cap tube was placed in a dark place and allowed to reach room temperature. The content was stored until suitable crystals were formed.

3. ^{1}H-NMR titration experiments

All experiments were conducted at ambient temperature and for pipetting Hamilton syringes were used. *Stock solution of host were prepared from 5.0 µmol of the corresponding Angle Bar derivative and dissolved in 400 µL of* CD_2Cl_2*, in addition 5.0 µmol of* $SiEt_4$ *was added as internal standard. Stock solutions of the guest were prepared as 125 mM solutions in* CD_2Cl_2*.* An NMR tube was charged with 100 µL of the corresponding host solution and 500 µL of CD_2Cl_2. For each measurement point a certain amount of guest solution was added, after the addition the tube was sealed and inverted 5 times. Each ^{1}H-NMR was measured with 8 scans and referenced to the deuterated solvent. The peak at 0.53 ppm of $SiEt_4$ (corresponding to the -CH_2-) was used as internal standard to check by integration the amount of Host and Guest after each measurement and corrected if necessary. For the determination of the binding constants (K in M^{-1}), the data collected was fitted using the Bindfit program[11,12]. The shift of the proton *ortho* to the I(III) center was selected and measured in shifts in Δppm vs guest equivalents. A 1:1 stoichiometry was assumed.

Results:

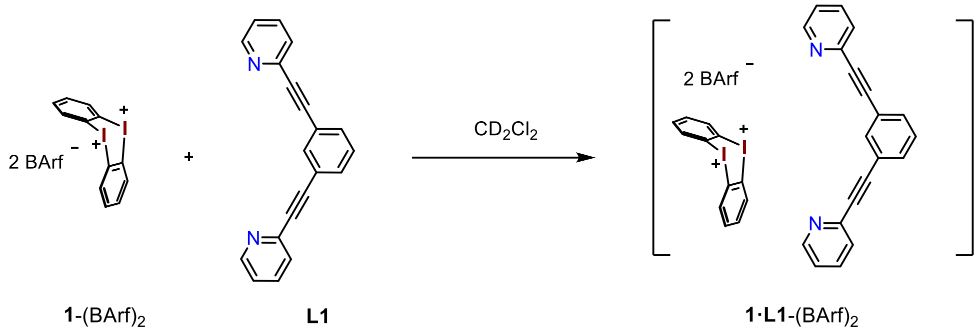

1-(BArf)$_2$ **L1** **1**·**L1**-(BArf)$_2$

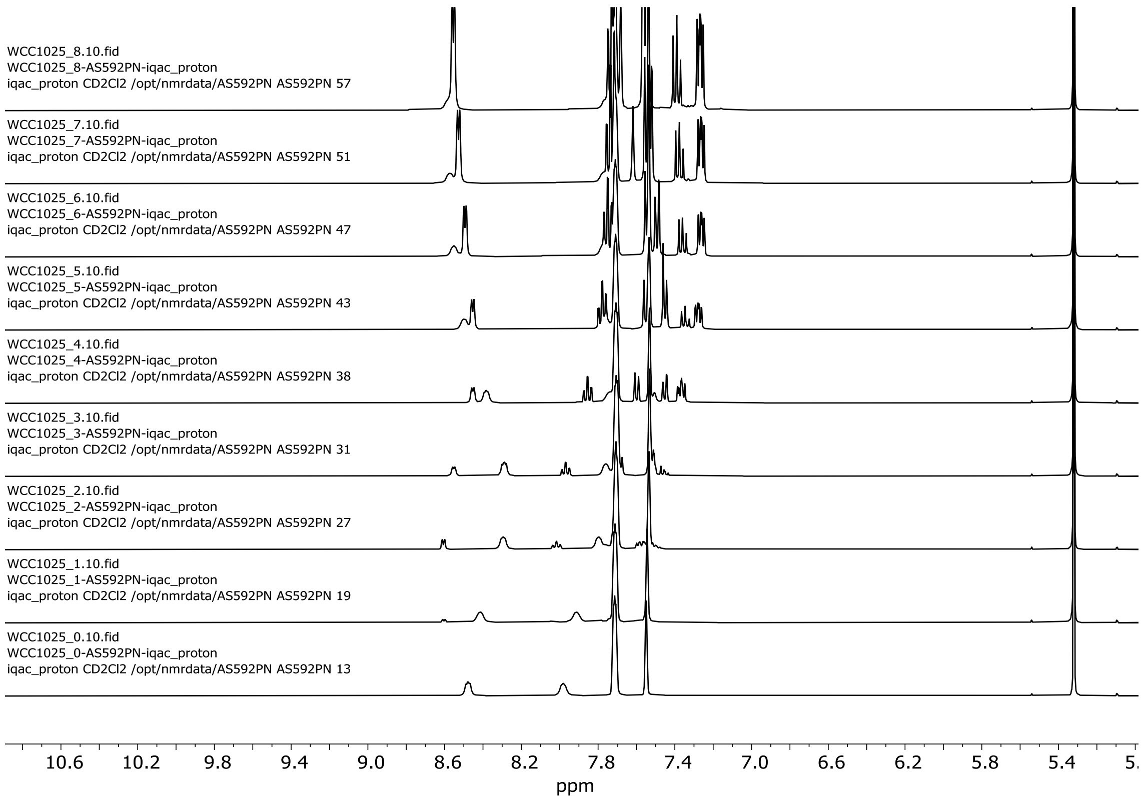

Figure S9. 400 MHz ^{1}H-NMR spectra for the titration between **1**-(BArf^{24})$_2$ with **L1** in CD_2Cl_2.

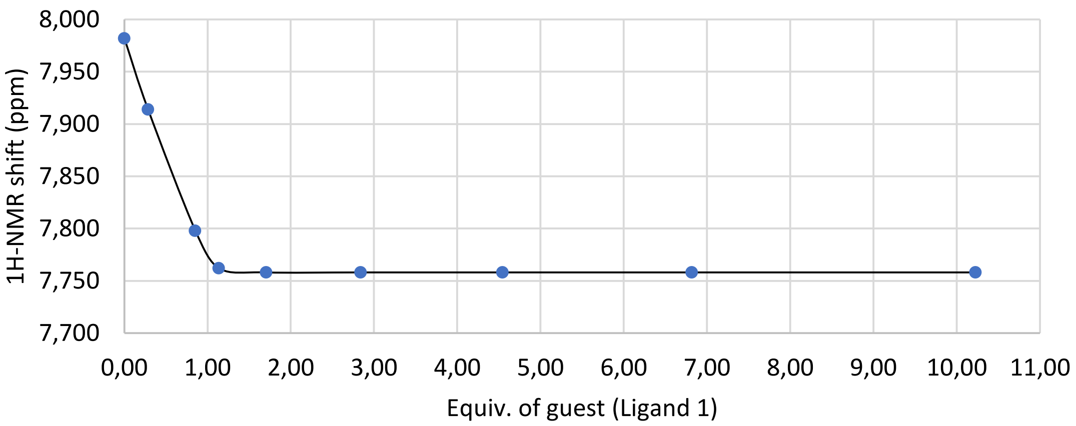

Figure S10. Titration of **1**-(BArf^{24})$_2$ with **L1** in CD_2Cl_2, $K = 1.19 \cdot 10^5$ M^{-1}.

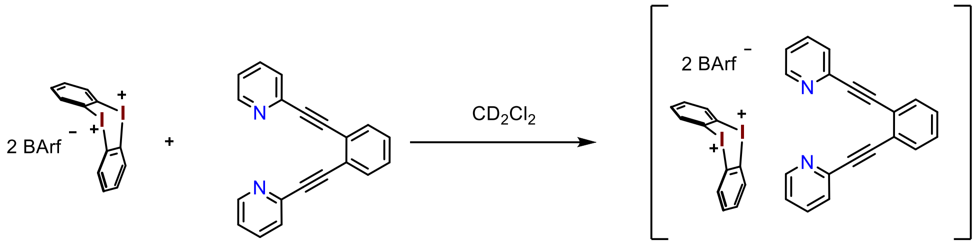

1-(BArf)$_2$ **L2** **1·L2**-(BArf)$_2$

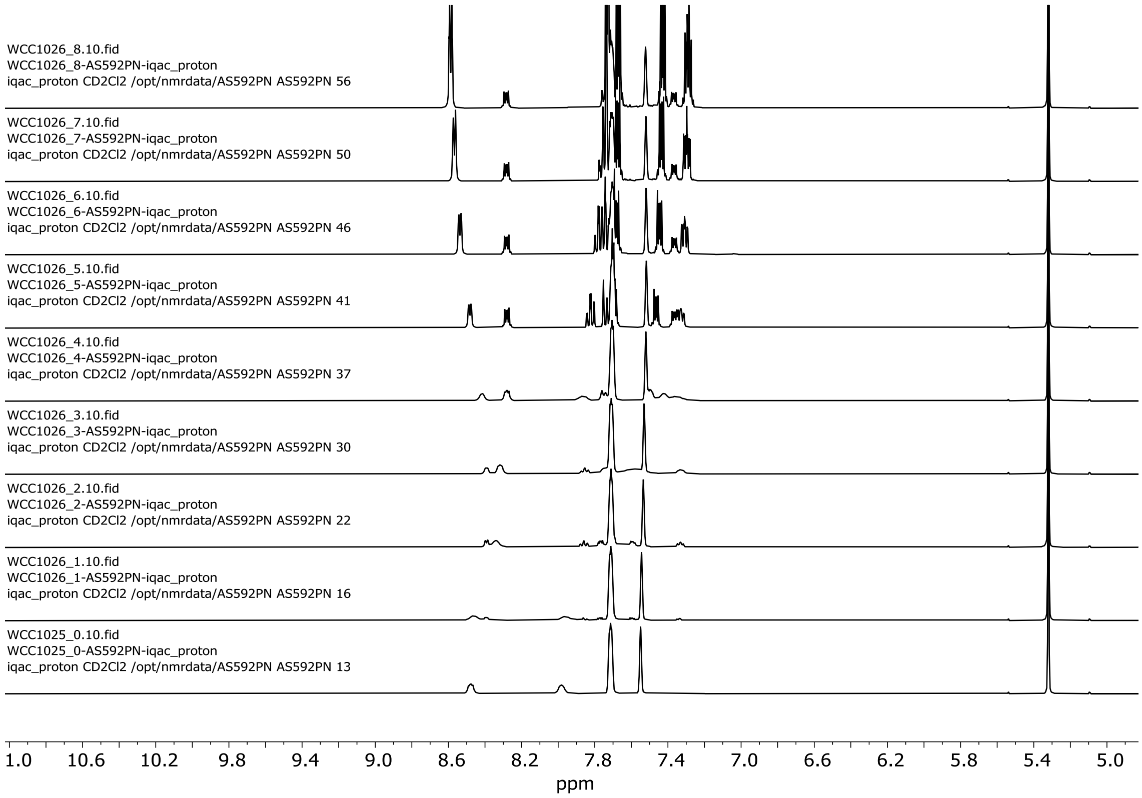

Figure S11. 400 MHz ^{1}H-NMR spectra for the titration between **1**-(BArf^{24})$_2$ with **L2** in CD_2Cl_2.

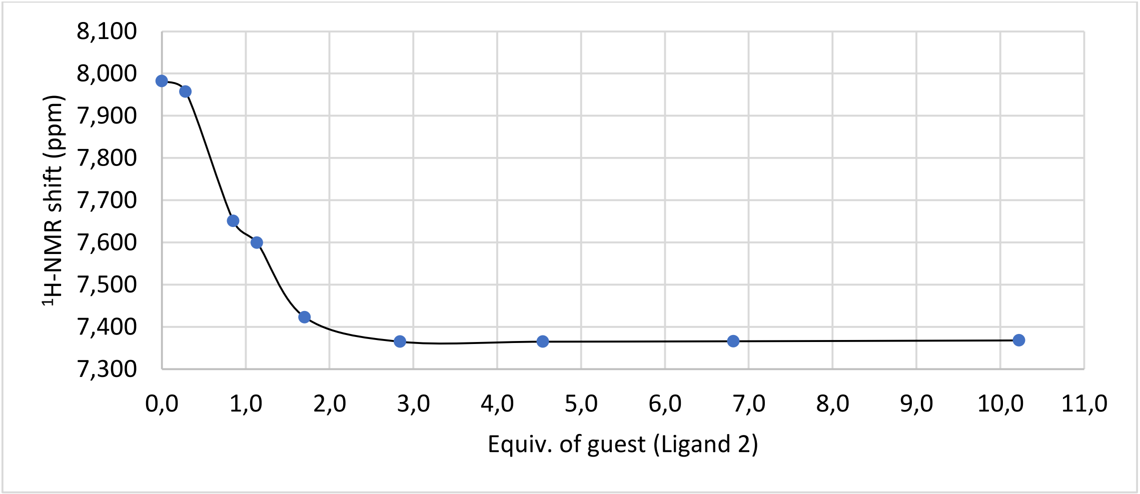

Figure S12. Titration of **1**-(BArf^{24})$_2$ with **L2** in CD_2Cl_2, $K = 1.79 \cdot 10^3$ M^{-1}.

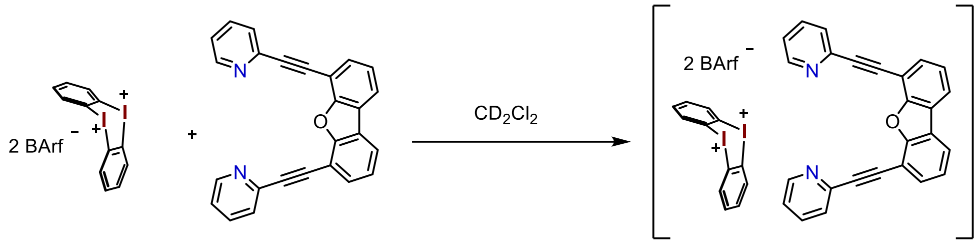

1-(BArf)$_2$ **L3** **1**·**L3**-(BArf)$_2$

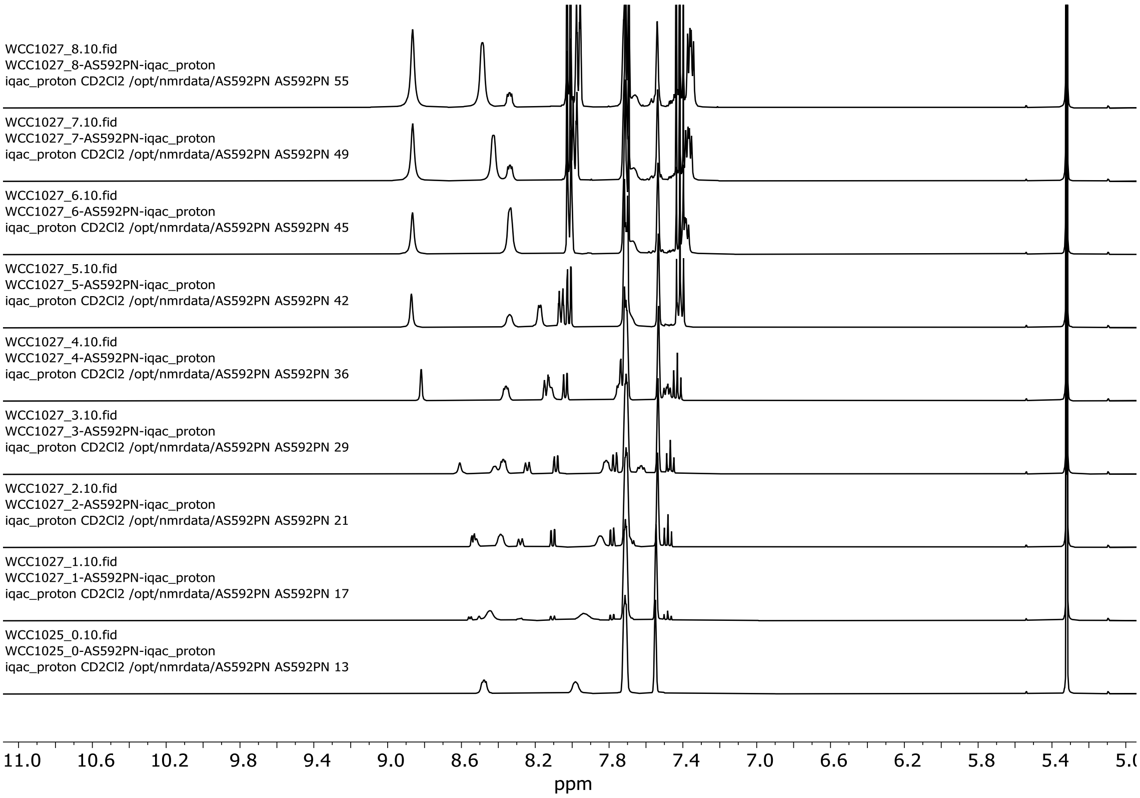

Figure S13. 400 MHz ^{1}H-NMR spectra for the titration between **1**-(BArf^{24})$_2$ with **L3** in CD_2Cl_2.

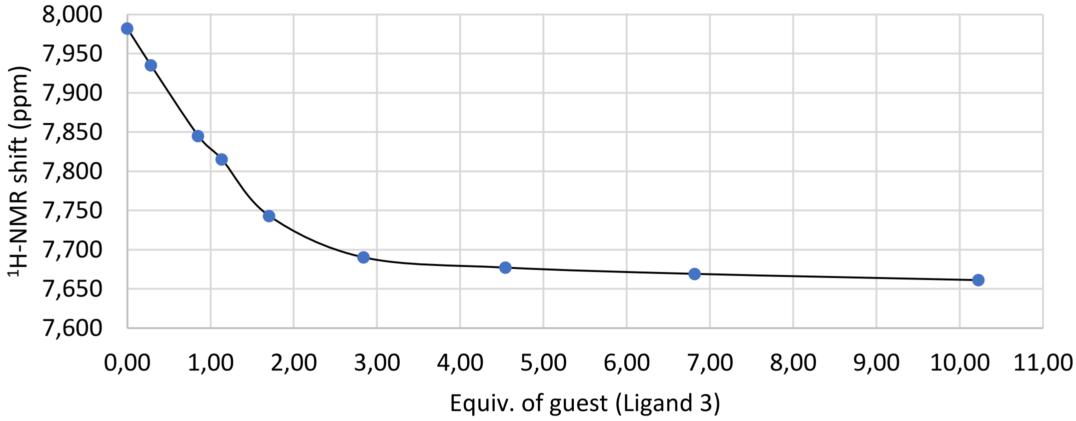

Figure S14. Titration of **1**-(BArf^{24})$_2$ with **L3** in CD_2Cl_2, $K = 9.36 \cdot 10^2$ M^{-1}.

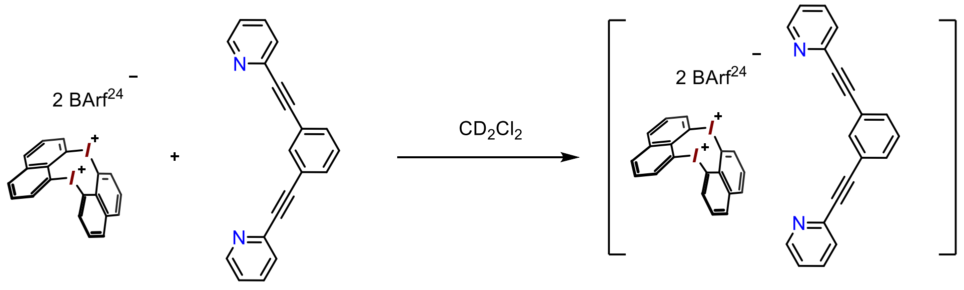

9-(BArf^{24})$_2$ **L1** 9·**L1**-(BArf^{24})$_2$

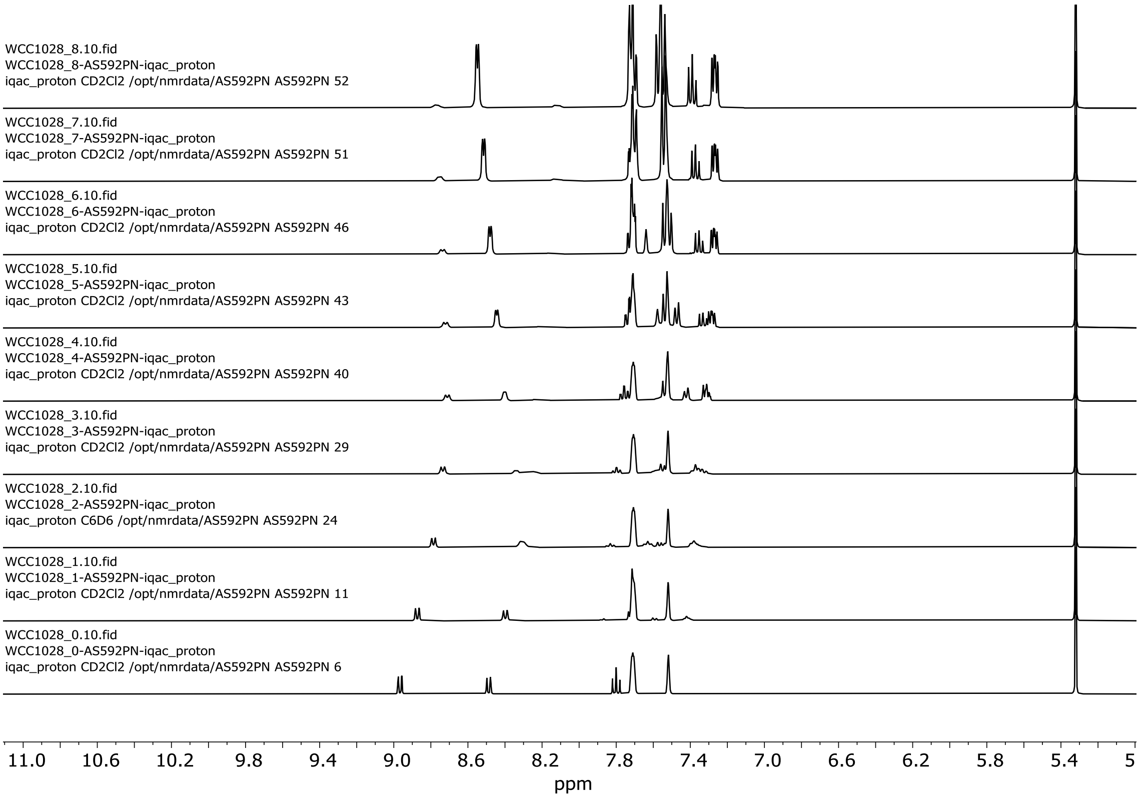

Figure S15. 400 MHz ^{1}H-NMR spectra for the titration between **9**-(BArf^{24})$_2$ with **L1** in CD_2Cl_2.

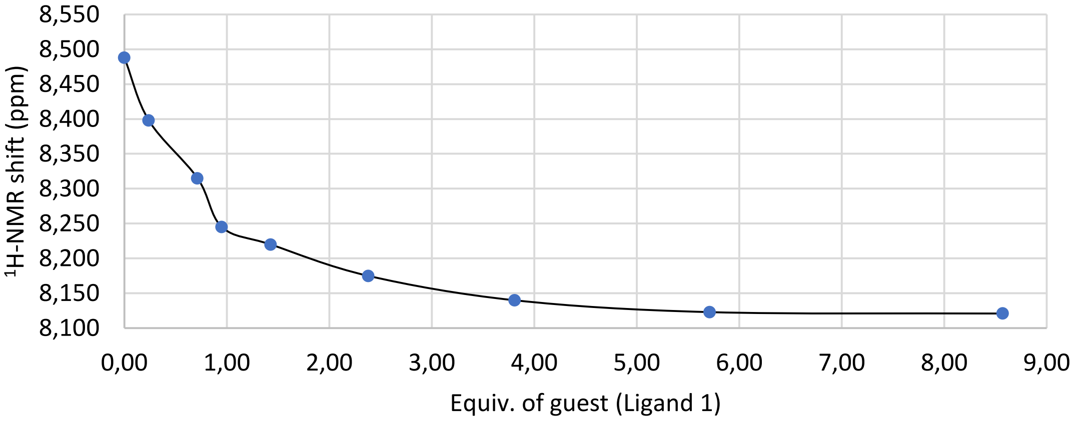

Figure S16. Titration of **9**-(BArf^{24})$_2$ with **L1** in CD_2Cl_2, K = 1.83·10^{3} M^{-1}.

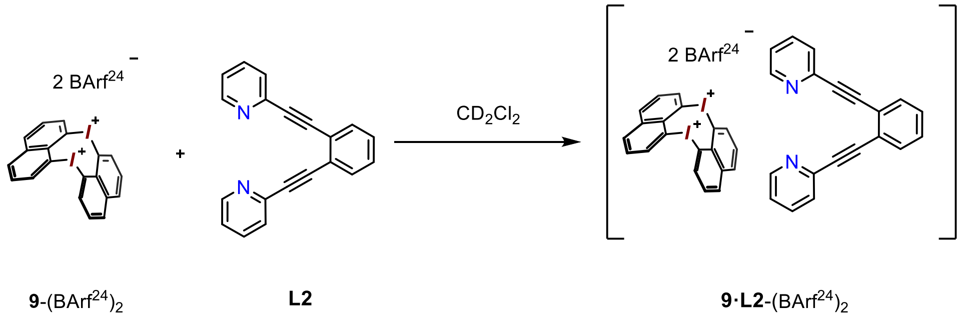

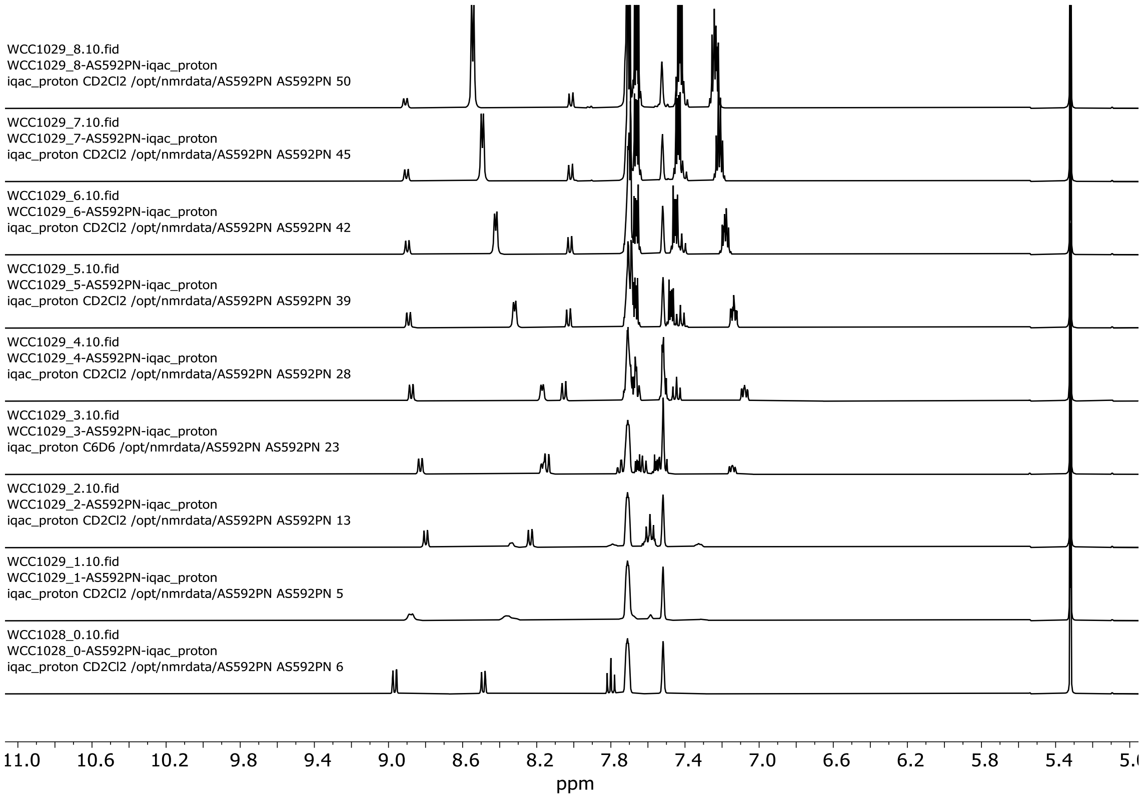

Figure S17. 400 MHz ^{1}H-NMR spectra for the titration between **9**-(BArf^{24})$_2$ with **L2** in CD_2Cl_2.

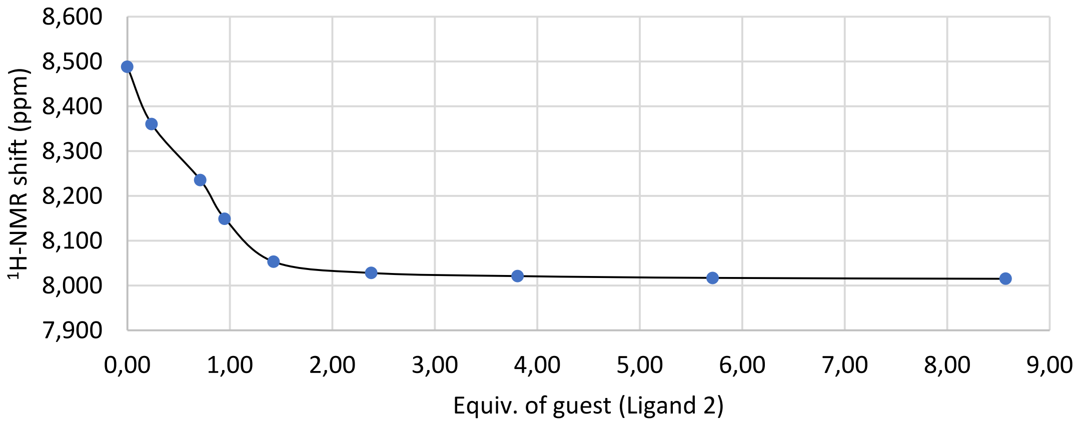

Figure S18. Titration of **9**-(BArf^{24})$_2$ with **L2** in CD_2Cl_2, K = 4.63·10^{3} M^{-1}.

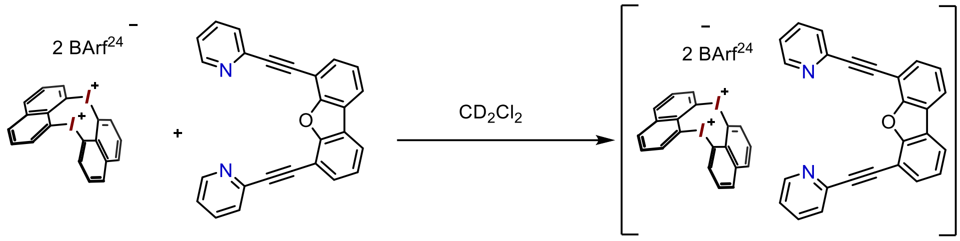

9-(BArf^{24})$_2$ **L3** **9·L3**-(BArf^{24})$_2$

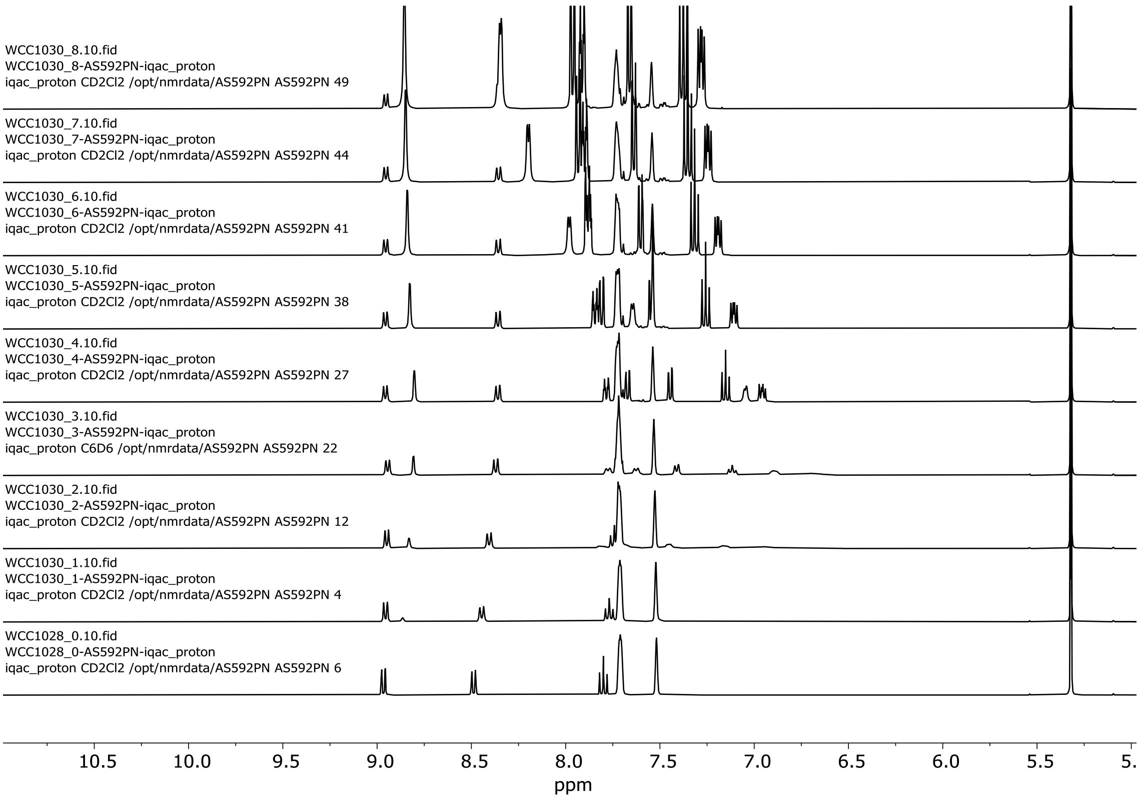

Figure S19. 400 MHz ^{1}H-NMR spectra for the titration between **9**-(BArf^{24})$_2$ with **L3** in CD_2Cl_2.

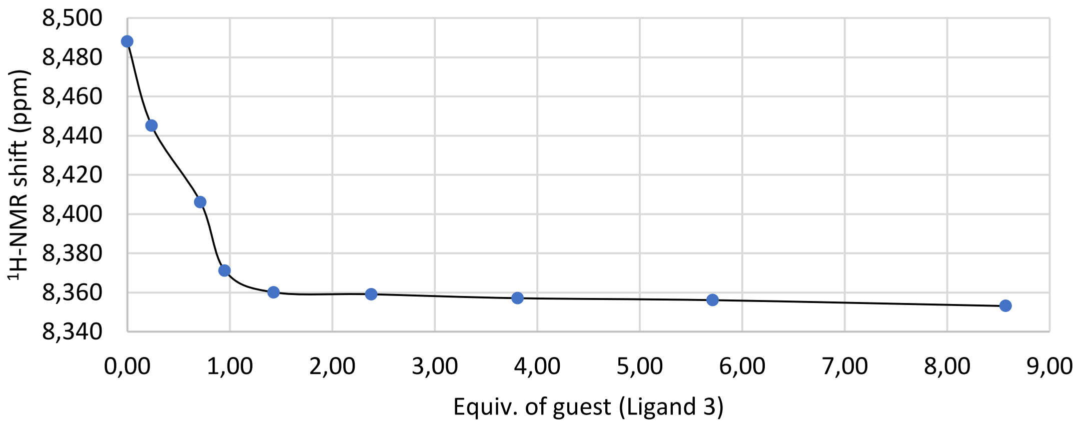

Figure S20. Titration of **9**-(BArf^{24})$_2$ with **L2** in CD_2Cl_2, $K = 2.68 \cdot 10^4$ M^{-1}.

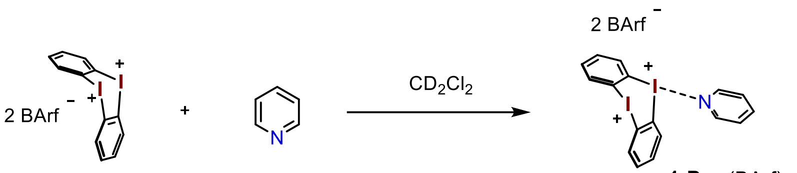

1-(BArf)$_2$ **1·Pyr**-(BArf)$_2$

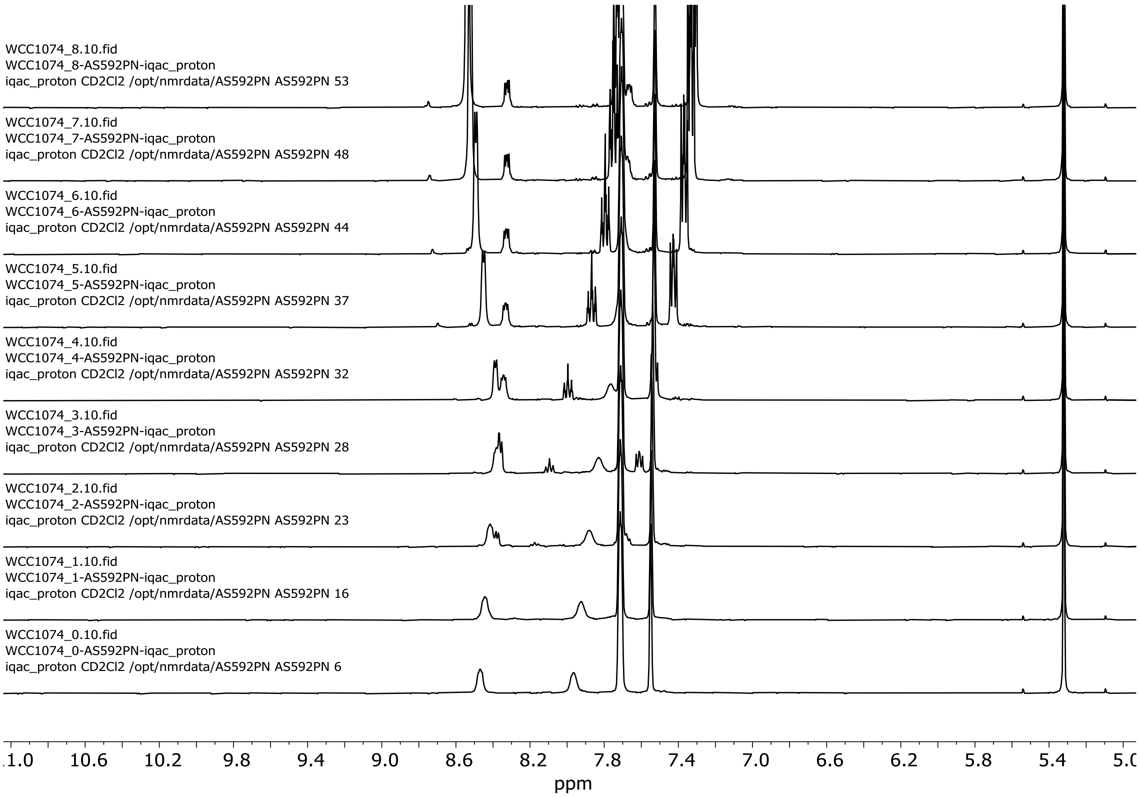

Figure S21. 400 MHz ^{1}H-NMR titration between **1**-(BArf^{24})$_2$ with pyridine in CD_2Cl_2.

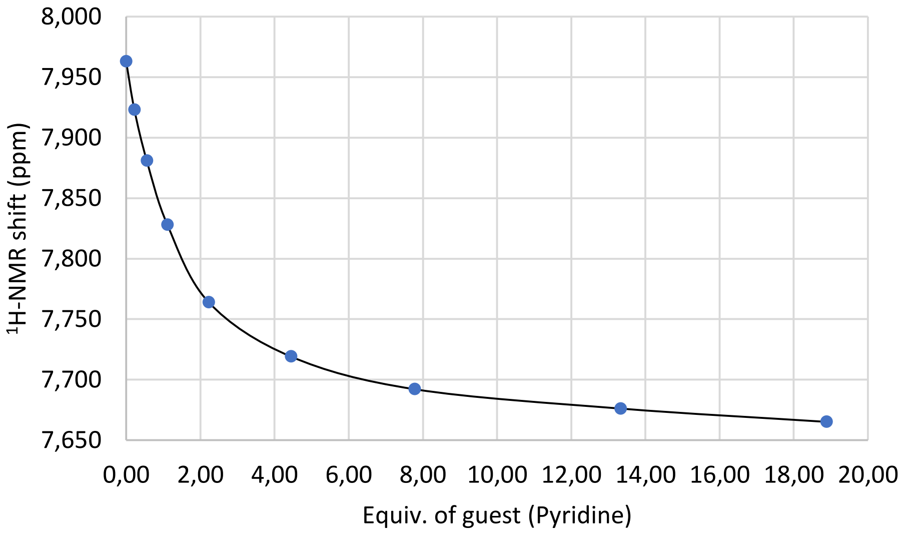

Figure S22. Titration of **1**-(BArf^{24})$_2$ with pyridine in CD_2Cl_2, K = 609 M^{-1}.

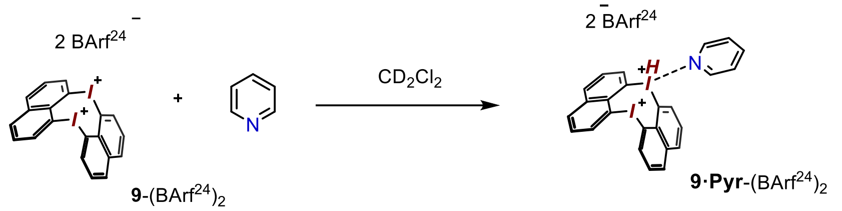

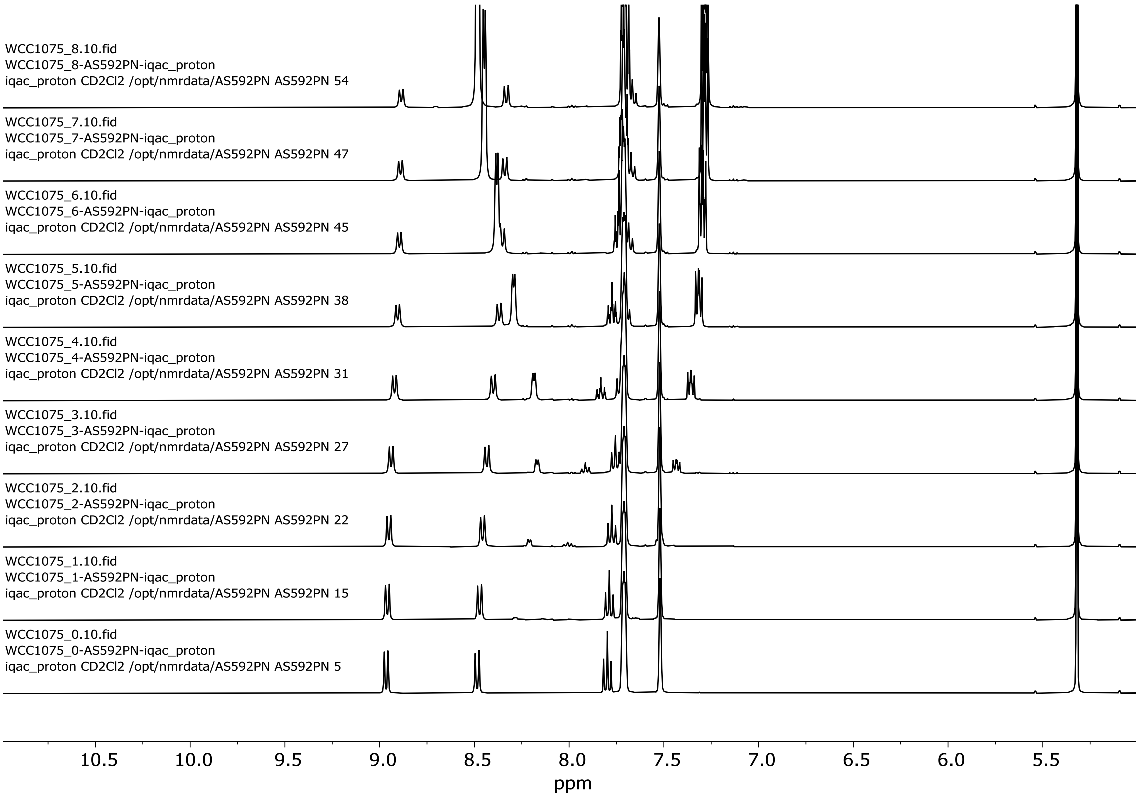

Figure S23. 400 MHz ^{1}H-NMR titration between **9**-(BArf^{24})$_2$ with pyridine in CD_2Cl_2.

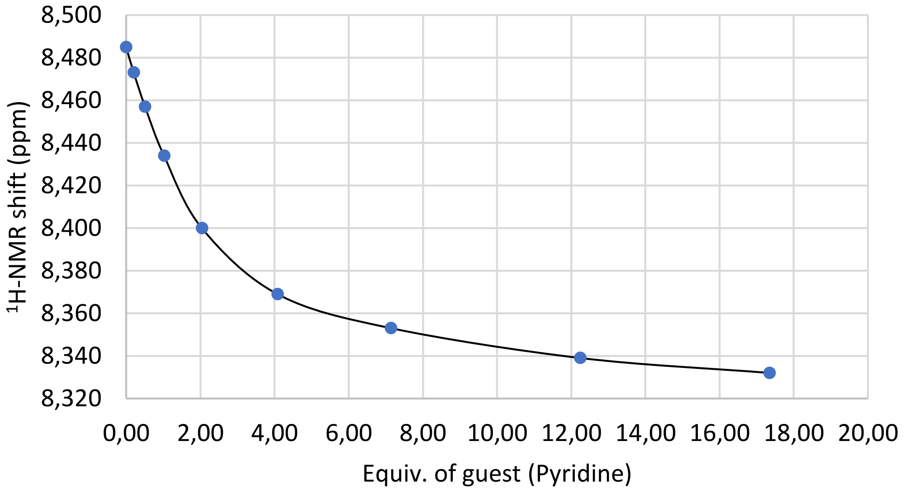

Figure S24. Titration of **1**-(BArf^{24})$_2$ with pyridine in CD_2Cl_2, K = 308 M^{-1}.

4. References

1. Reger, D. L.; Wright, T. D.; Little, C. A.; Lamba, J. J. S.; Smith, M. D. Control of the Stereochemical Impact of the Lone Pair in Lead(II) Tris(pyrazolyl)methane Complexes. Improved Preparation of Na{B[3,5-$(CF_3)_2C_6H_3]_4$}. *Inorg. Chem.* **2001**, *40*(15), 3810-3814.

2. Ye, C.; Twamley, B.; Shreeve, J. N. M. Straightforward Syntheses of Hypervalent Iodine(III) Reagents Mediated by Selectfluor. *Org. Lett.* **2005**, *7*, 3961−3964.

3. Kervefors, G.; Becker, A.; Dey, C.; Olofsson, B. Metal-free formal synthesis of phenoxazine. *Beilstein J. Org. Chem.* **2018**, *14*, 1491–1497.

4. Robidas, R.; Guérin, V.; Provençal, L.; Echeverria, M.; Legault, C. Y. Investigation of Iodonium Trifluoroborate Zwitterions as Bifunctional Arene Reagents. *Org. Lett.* **2017**, *19*, 6420−6423.

5. Weimar, M.; Dürner, G.; Bats, J. W. and Göbel M. W. Enantioselective Synthesis of (+)-Estrone Exploiting a Hydrogen Bond-Promoted Diels−Alder Reaction. *J. Org. Chem.* **2010**, *75*(8) 2718–2721.

6. Dore, A.; Gladiali, S.; Cossu, S.; De Lucchi, O. Binaphthothiophene: source of 1,1'-binaphthalene, perylene, 2,2'-diiodo- and 2,2'-dimethyl-1,1'-binaphthalene. Synlett, **1992**, *10*, 807-808.

7. Makino, T.; Yamasaki, R.; Saito, S. Concise Synthesis of Diborylxanthenes, *Synthesis* **2008**, *6*, 859-864.

8. Carlsson, A.-C. C.; Grafenstein, J.; Budnjo, A.; Laurila, J. L.;̈ Bergquist, J.; Karim, A.; Kleinmaier, R.; Brath, U.; Erdelyi, M. Symmetric Halogen Bonding Is Preferred in Solution. *J. Am. Chem. Soc.* **2012**, *134*, 5706.

9. Kaae, B. H.; Harpsoe, K.; Kvist, T.; Mathiesen, J. M.; Molck, C.; Gloriam, D.; Jimenez, H. N.; Uberti, M. A.; Nielsen, S. M.; Nielsen, B.; Brauner-Osborne, H.; Sauerberg, P.; Clausen, R. P.; and Madsen, U. Structure-activity relationships for negative allosteric mGluR5 modulators. *ChemMedChem,* **2012**, *7*, 440−451.

10. Zhang, Y.; Diver, S. T. A Macrocyclic Ruthenium Carbene for Size-Selective Alkene Metathesis. *J. Am. Chem. Soc.* **2020**, *142*, 3371−3374.

11. Brynn Hibbert, D.; Thordarson, P. The death of the Job plot, transparency, open science and online tools, uncertainty estimation methods and other developments in supramolecular chemistry data analysis. *Chem. Commun*. **2016**, *52*, 12792–12805.

12. Thordarson, P. Determining association constants from titration experiments in supramolecular chemistry. *Chem. Soc. Rev.* **2011**, *40*, 1305–1323.

5. NMR Spectras

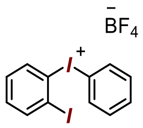

(**2**): ^1H NMR (400 MHz, DMSO-d_6)

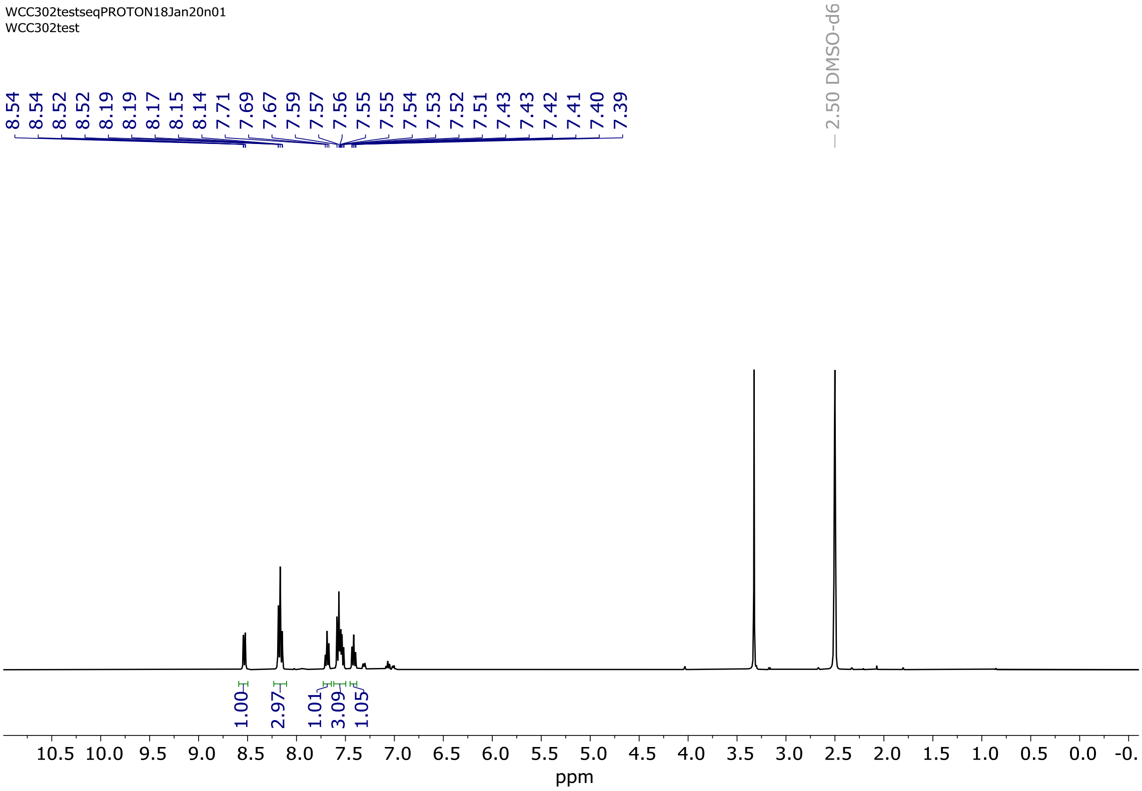

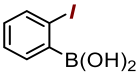

(2-iodophenyl)boronic acid: ^{1}H NMR (400 MHz, $CDCl_3$)

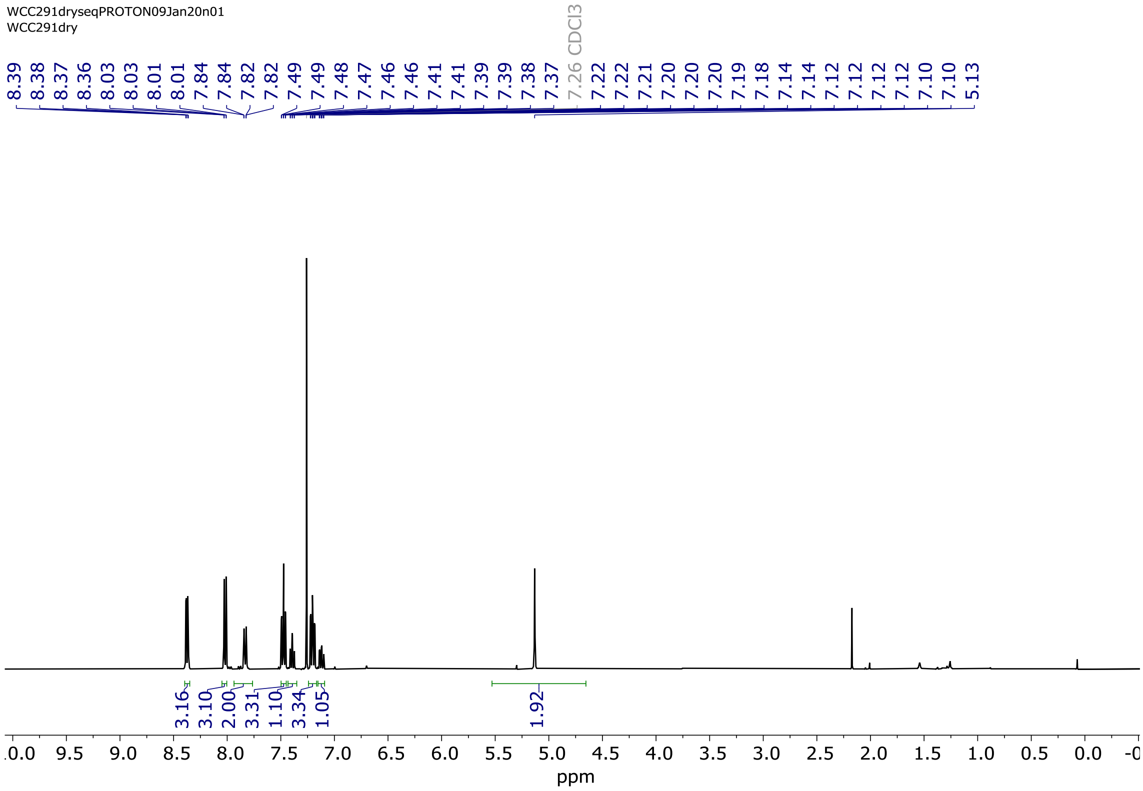

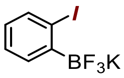 (**4**): ^{1}H NMR (400 MHz, CD_3OD)

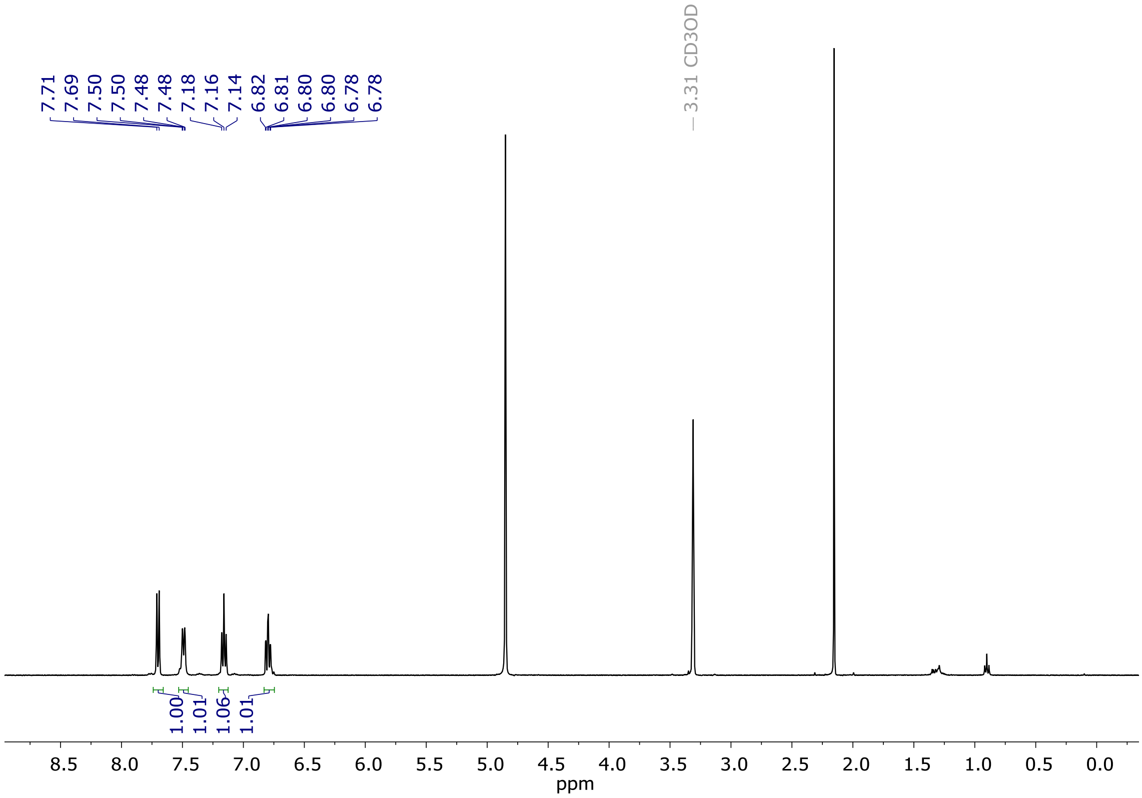

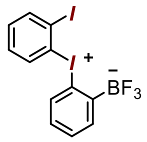

(**5**): ^{1}H NMR (400 MHz, CD_3OD)

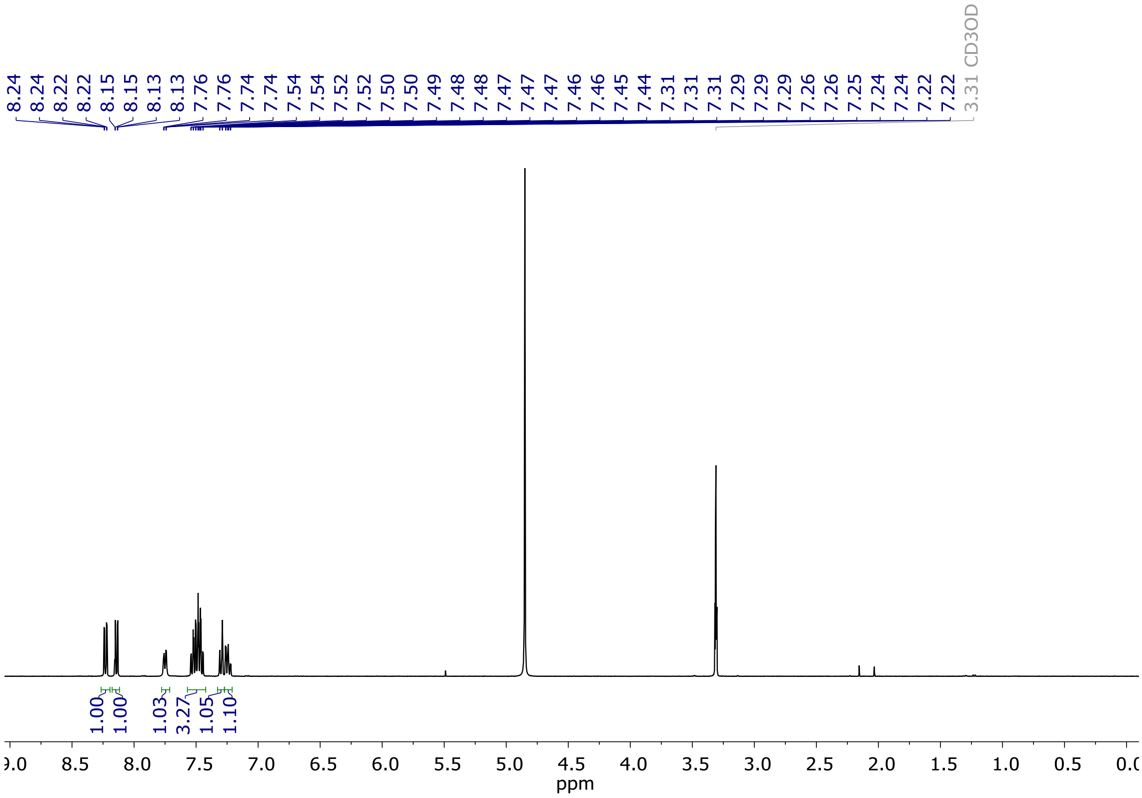

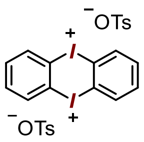

1-$(OTs)_2$: ^{1}H NMR (400 MHz, DMSO-d_6)

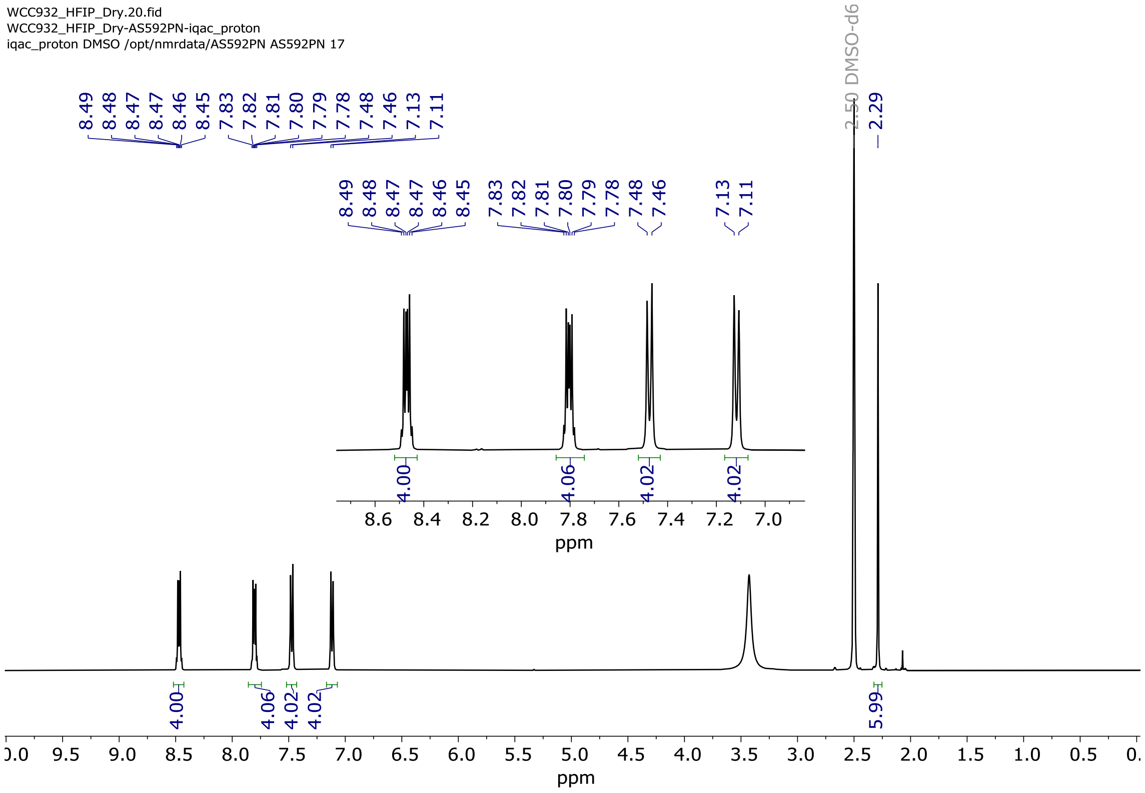

^{13}C NMR (101 MHz, DMSO-d_6)

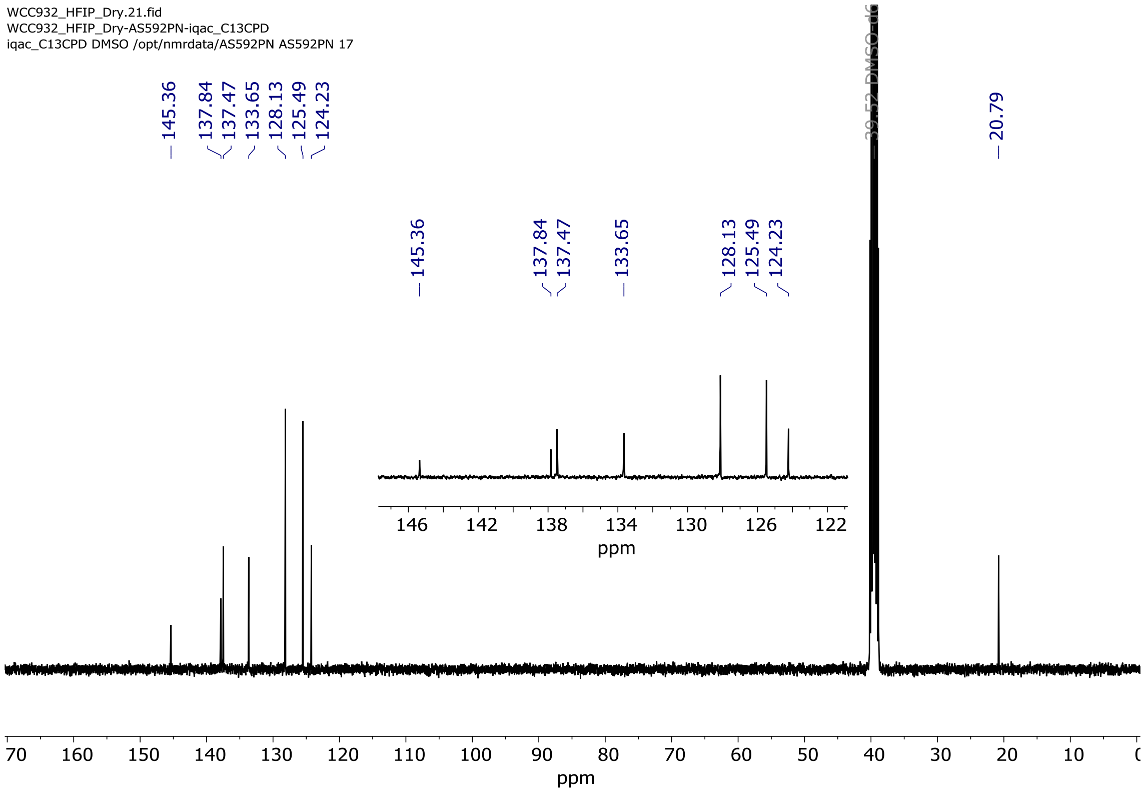

^{1}H-^{13}C HSQCEDETGPSISP experiment in DMSO-d_6

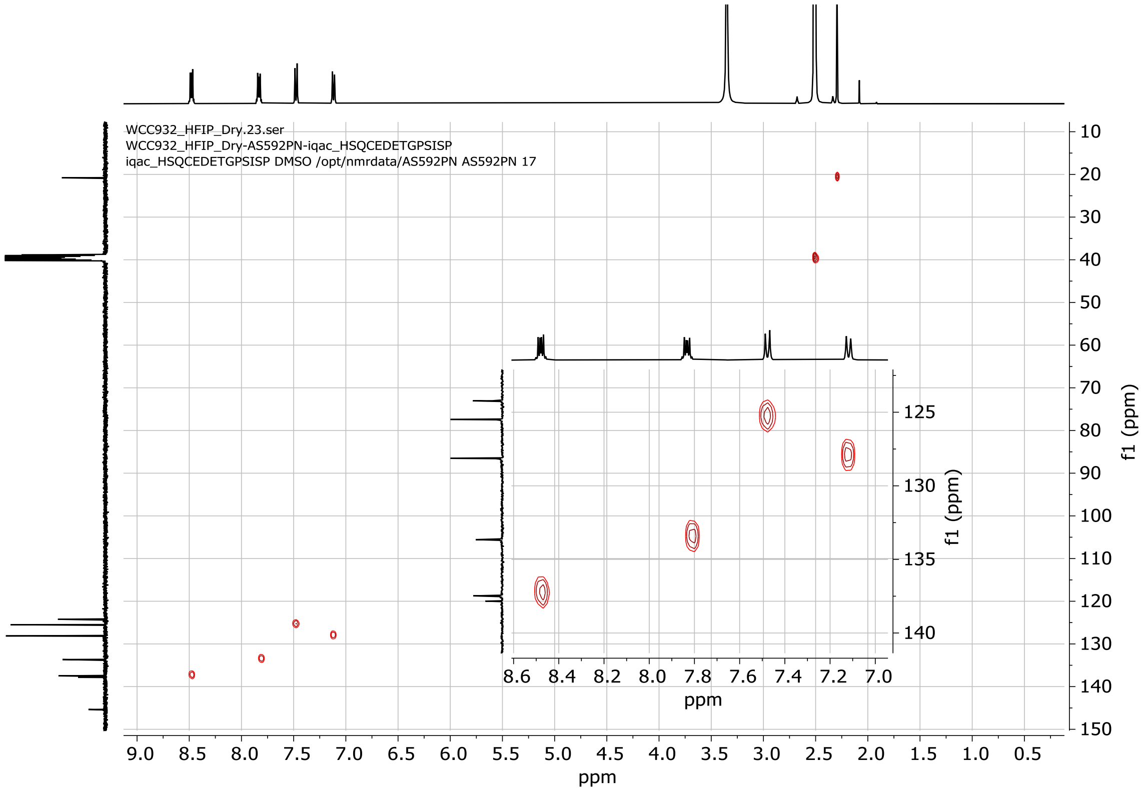

^{1}H-^{13}C HMBCGP experiment in DMSO-d_6

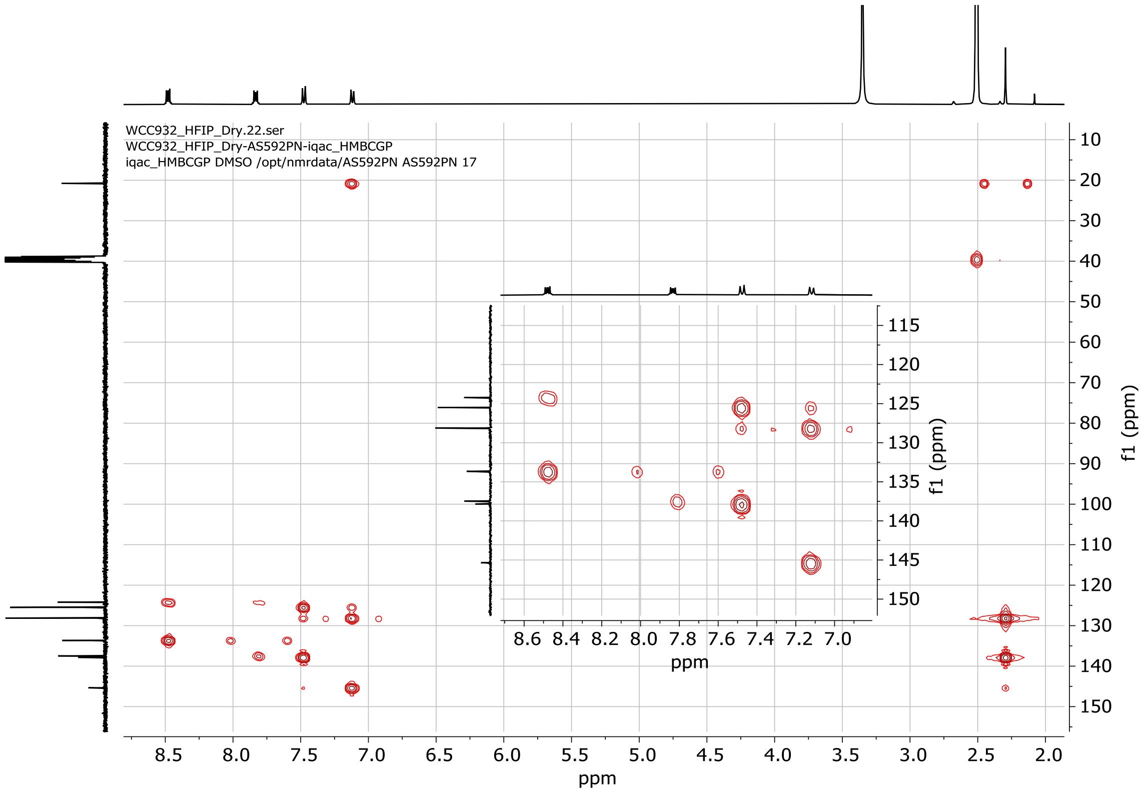

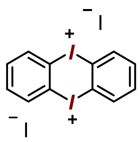

1-(I)$_2$: ^{1}H NMR (400 MHz, DMSO-d_6)

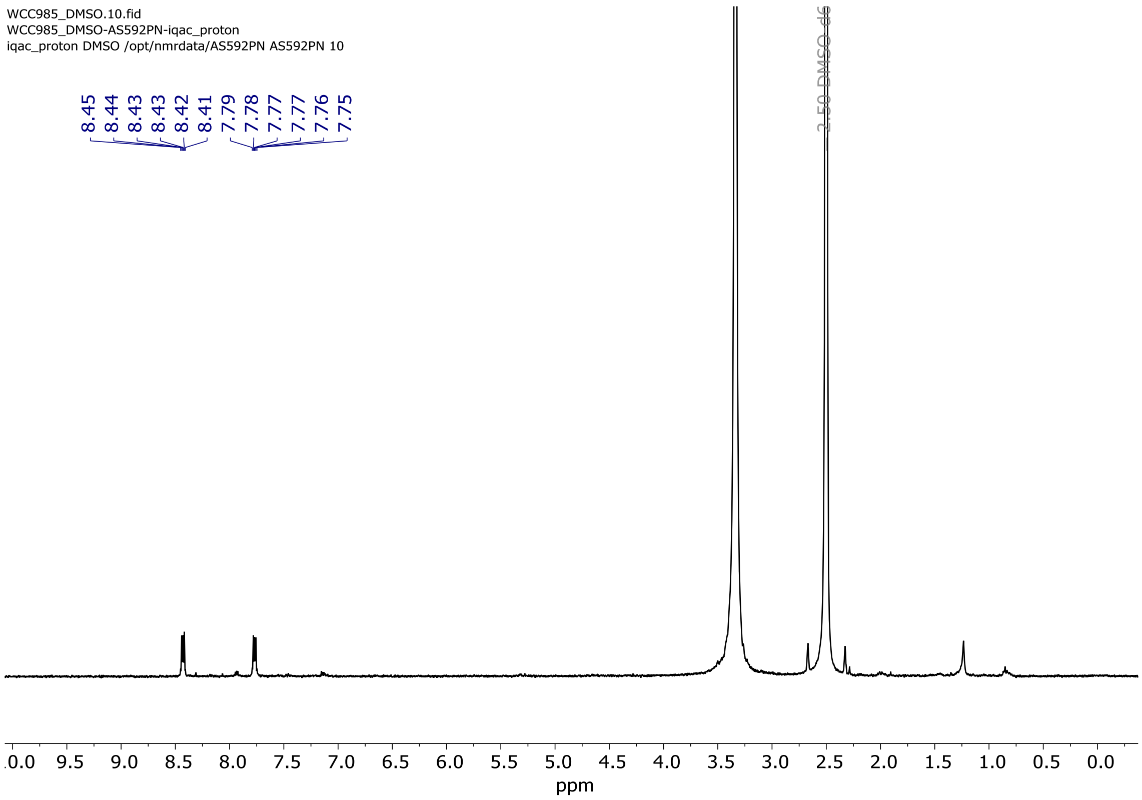

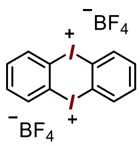

1-(BF_4)$_2$: ^1H NMR (400 MHz, CD_3OD)

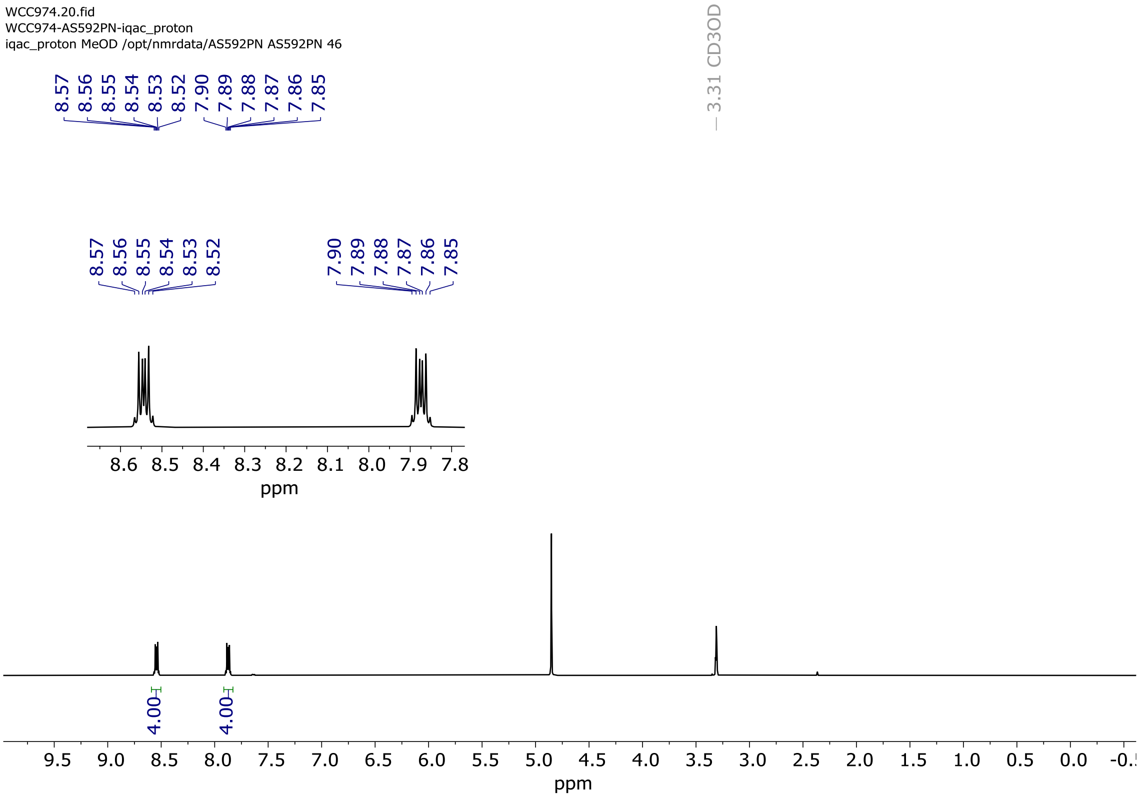

^{13}C NMR (101 MHz, CD_3OD)

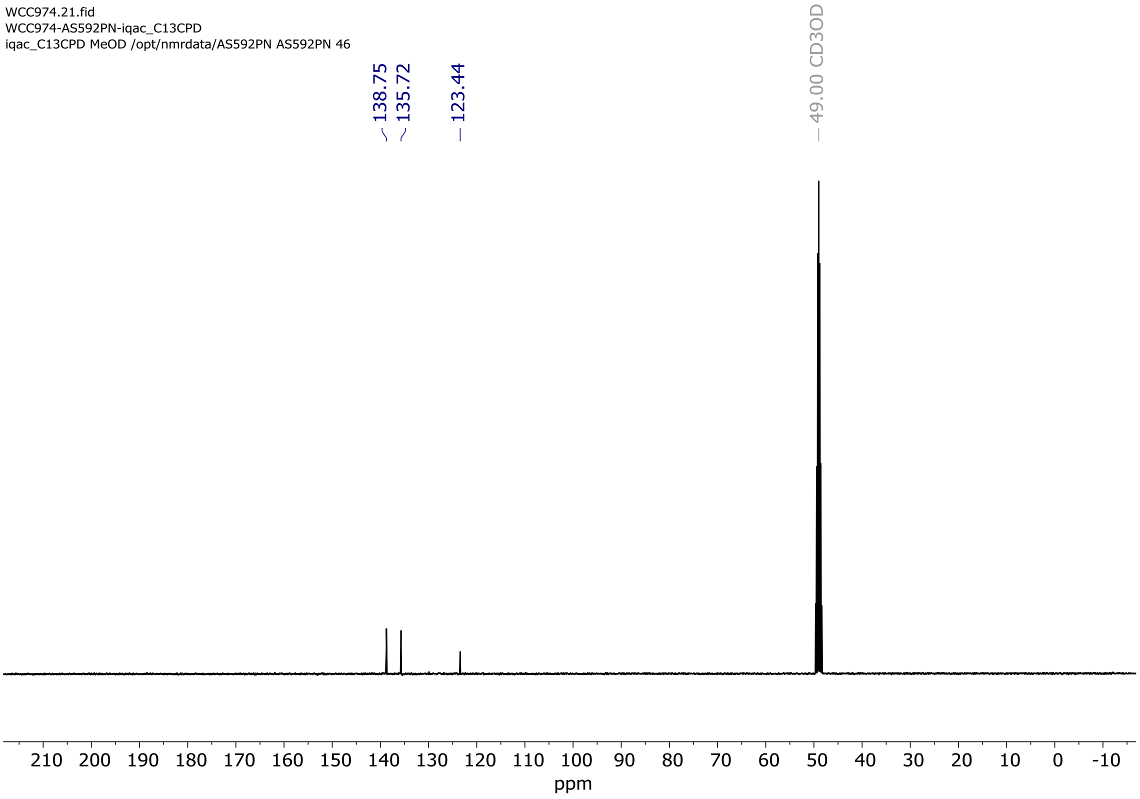

^{1}H-^{13}C HSQCEDETGPSISP experiment in CD_3OD

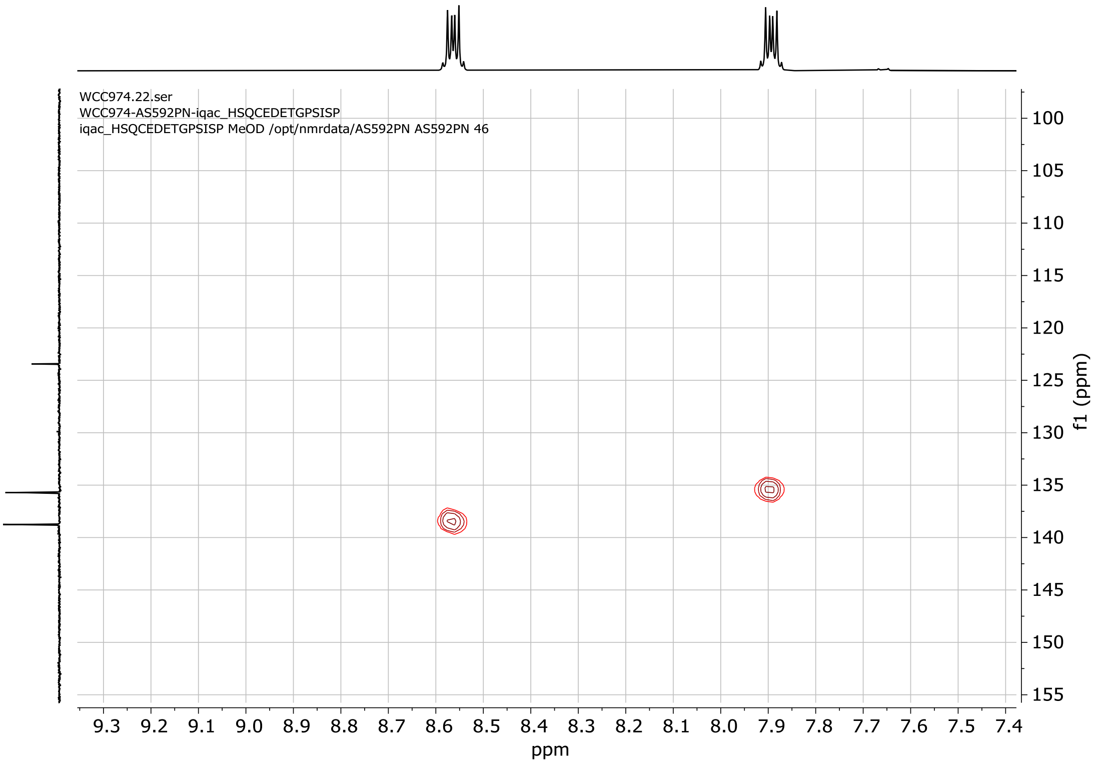

^{19}F NMR (376 MHz, CD_3OD)

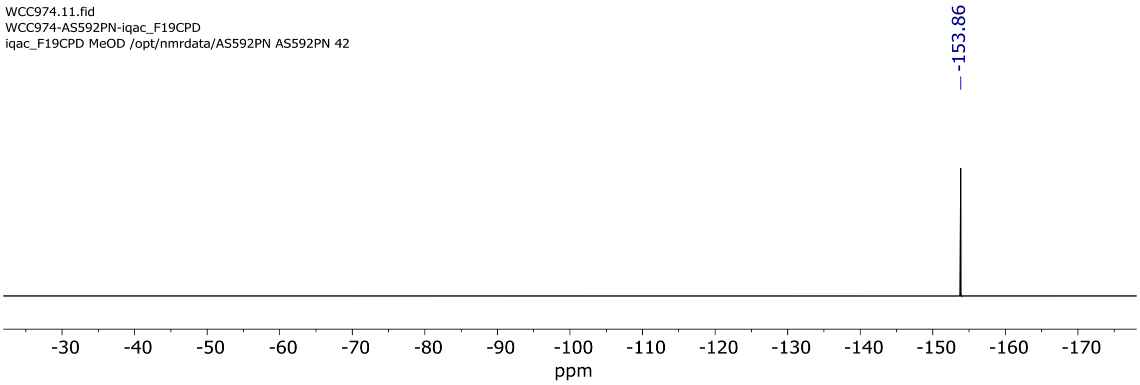

^{11}B NMR (128 MHz, CD_3OD)

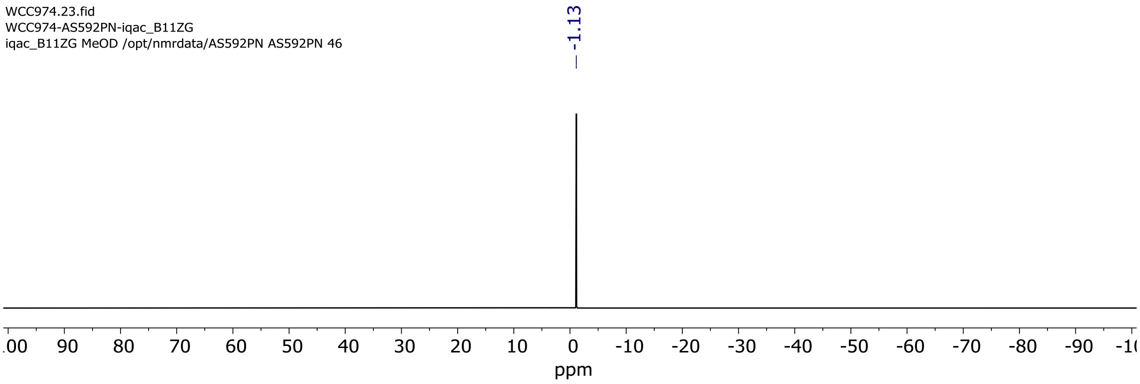

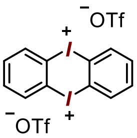

1-(OTf)$_2$: ^{1}H NMR (400 MHz, CD_3OD)

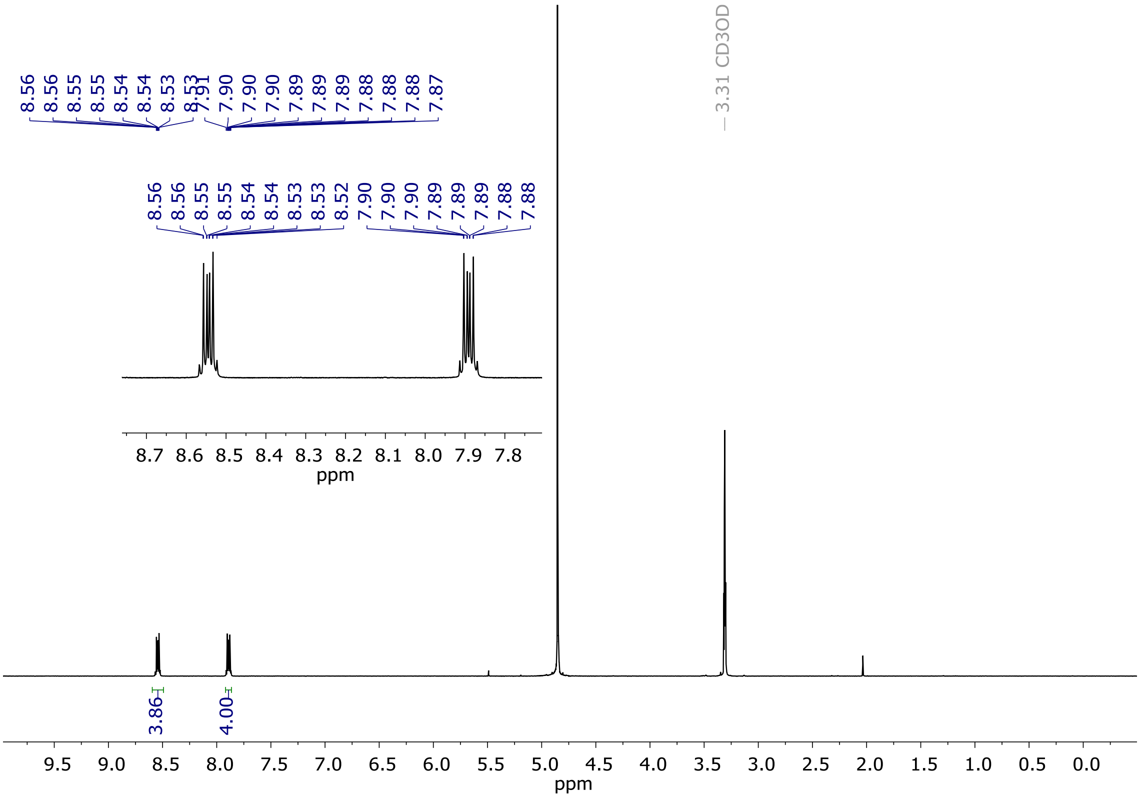

^{13}C NMR (101 MHz, CD_3OD)

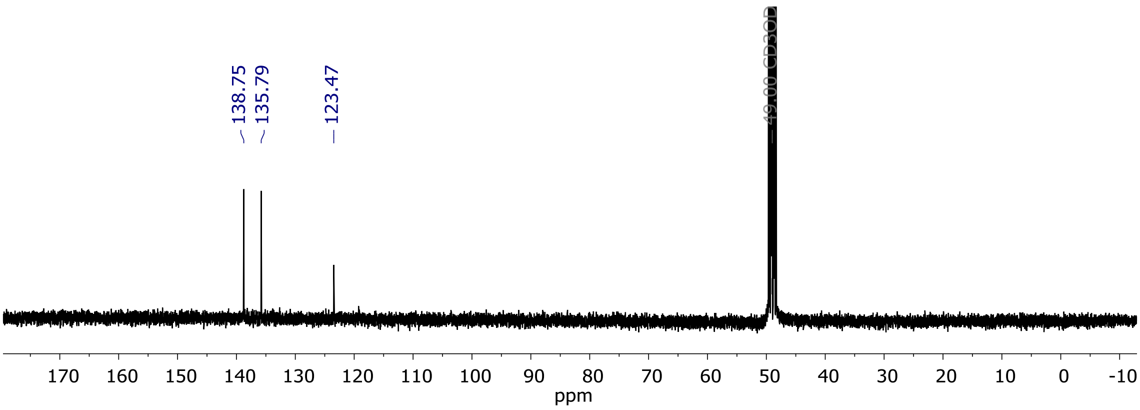

^{19}F NMR (376 MHz, CD_3OD)

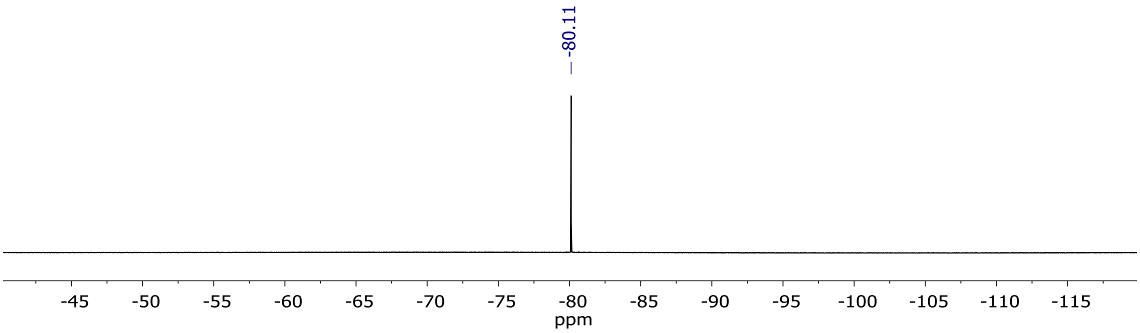

COSY ^{1}H-^{1}H experiment in CD_3OD

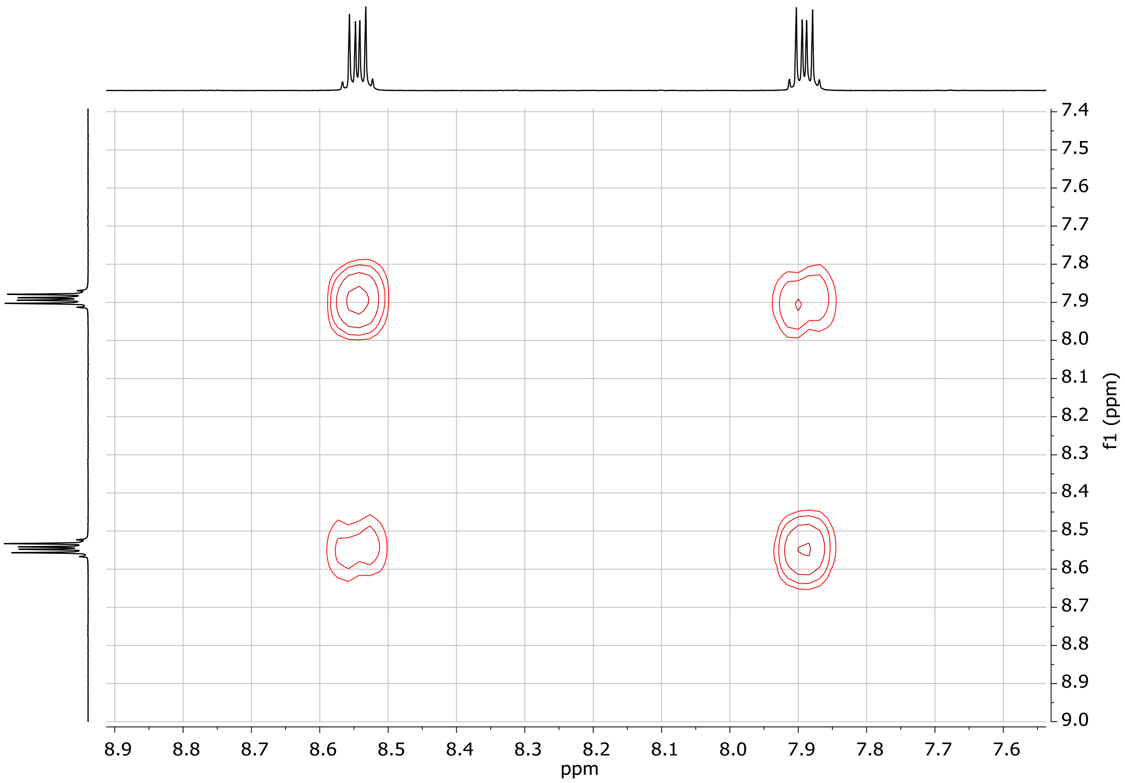

HSQCAD experiment in CD_3OD

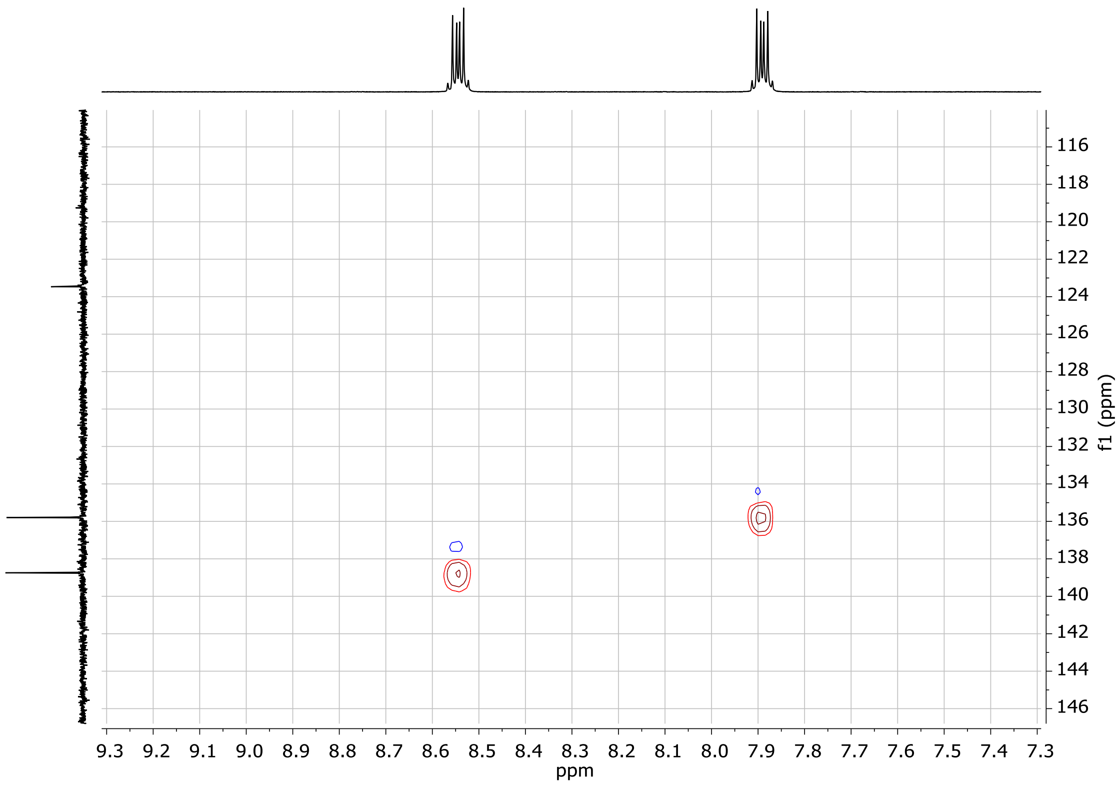

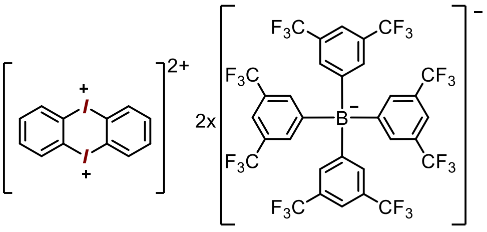

1-(BArf[24])$_2$: ^{1}H NMR (400 MHz, CD_3OD)

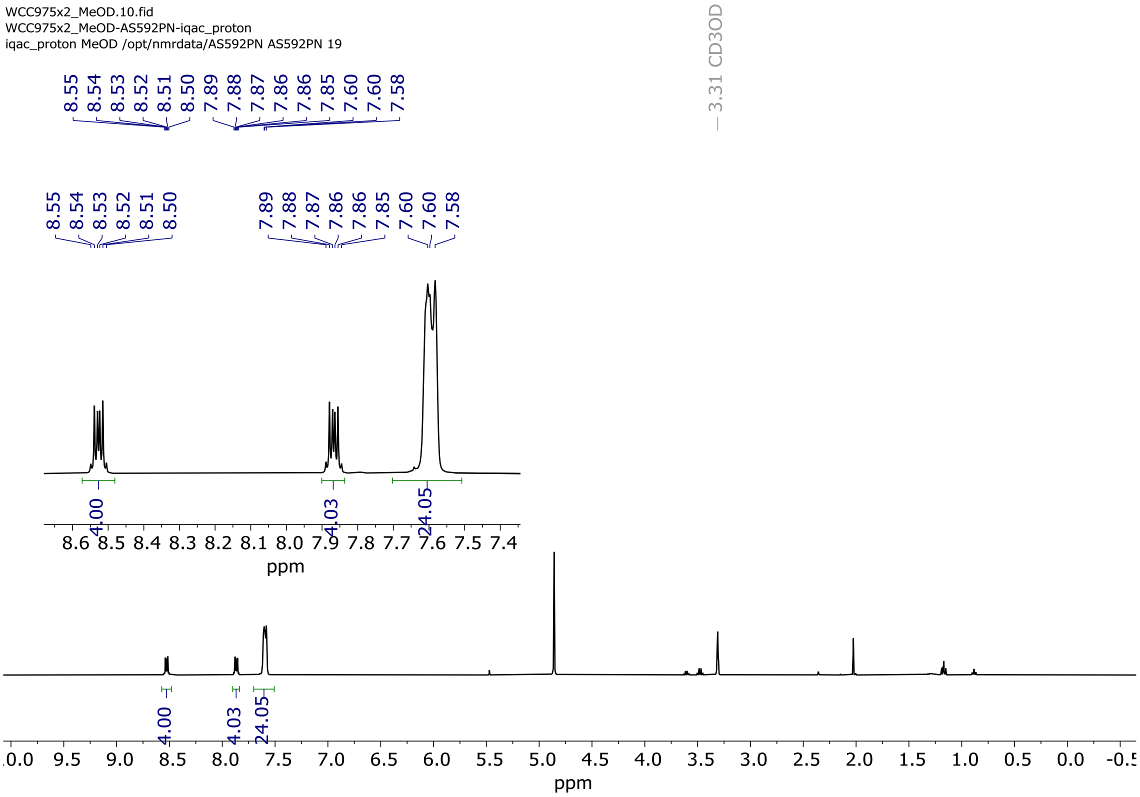

^{13}C NMR (101 MHz, CD_3OD)

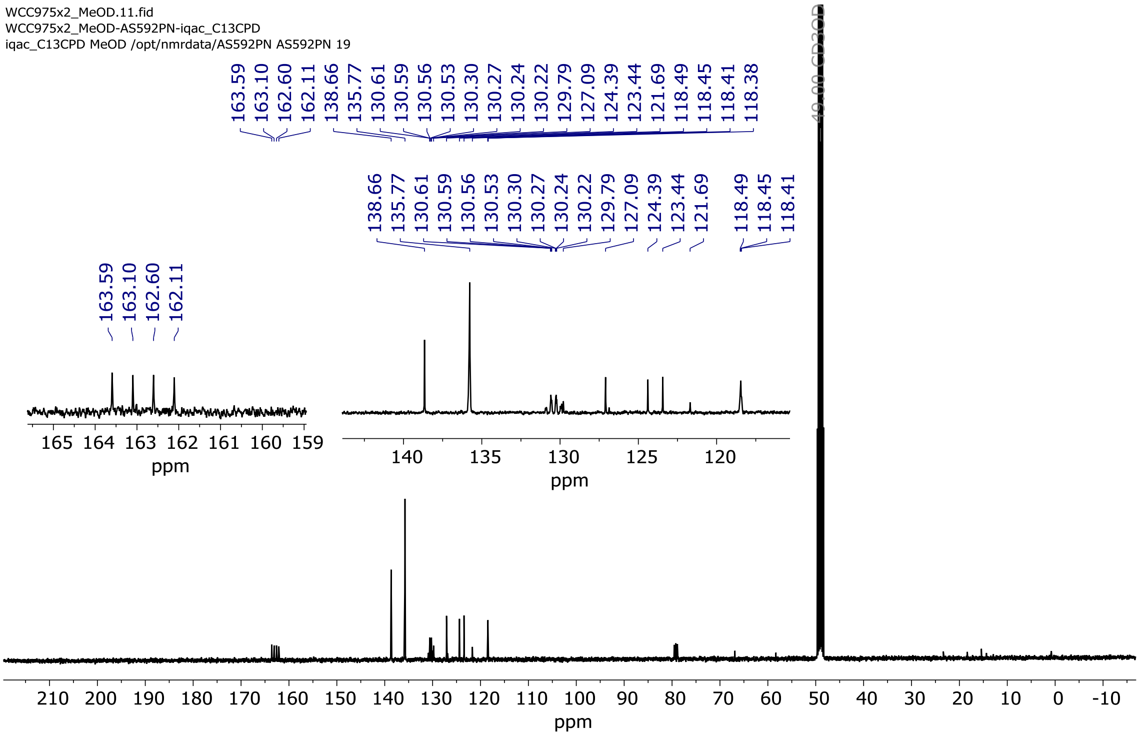

^{19}F NMR (376 MHz, CD_3OD)

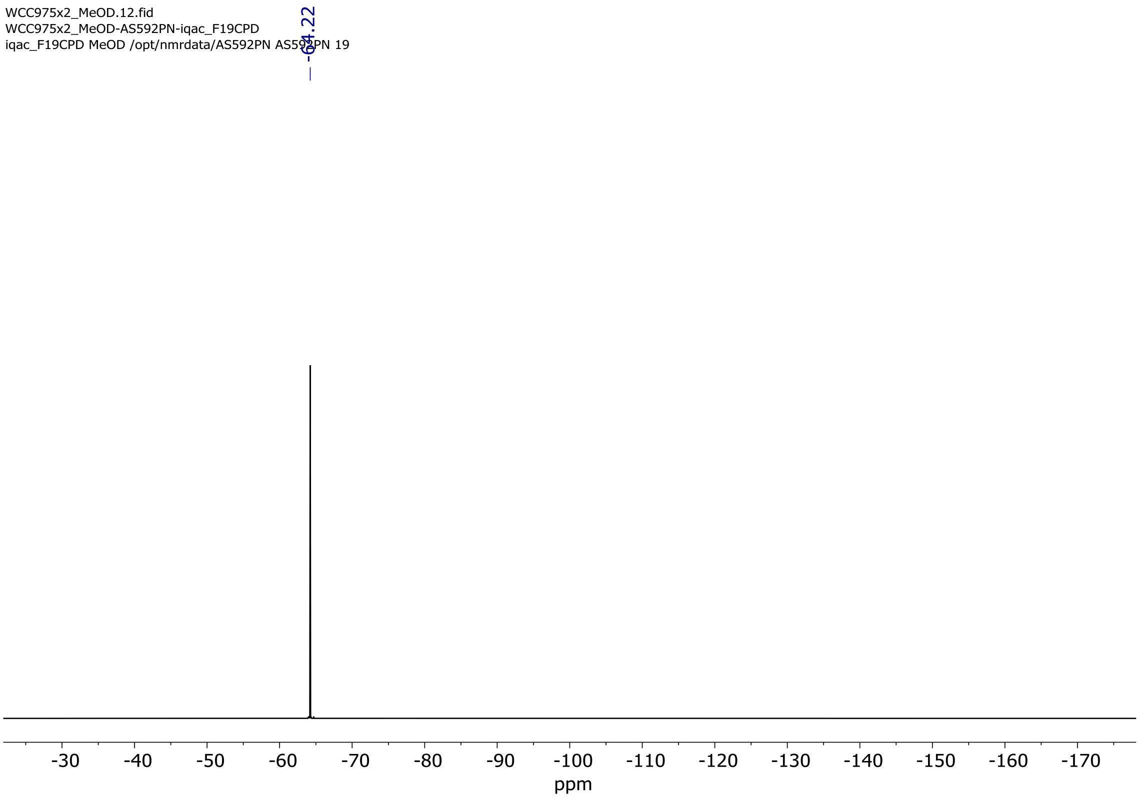

^{11}B NMR (128 MHz, CD_3OD)

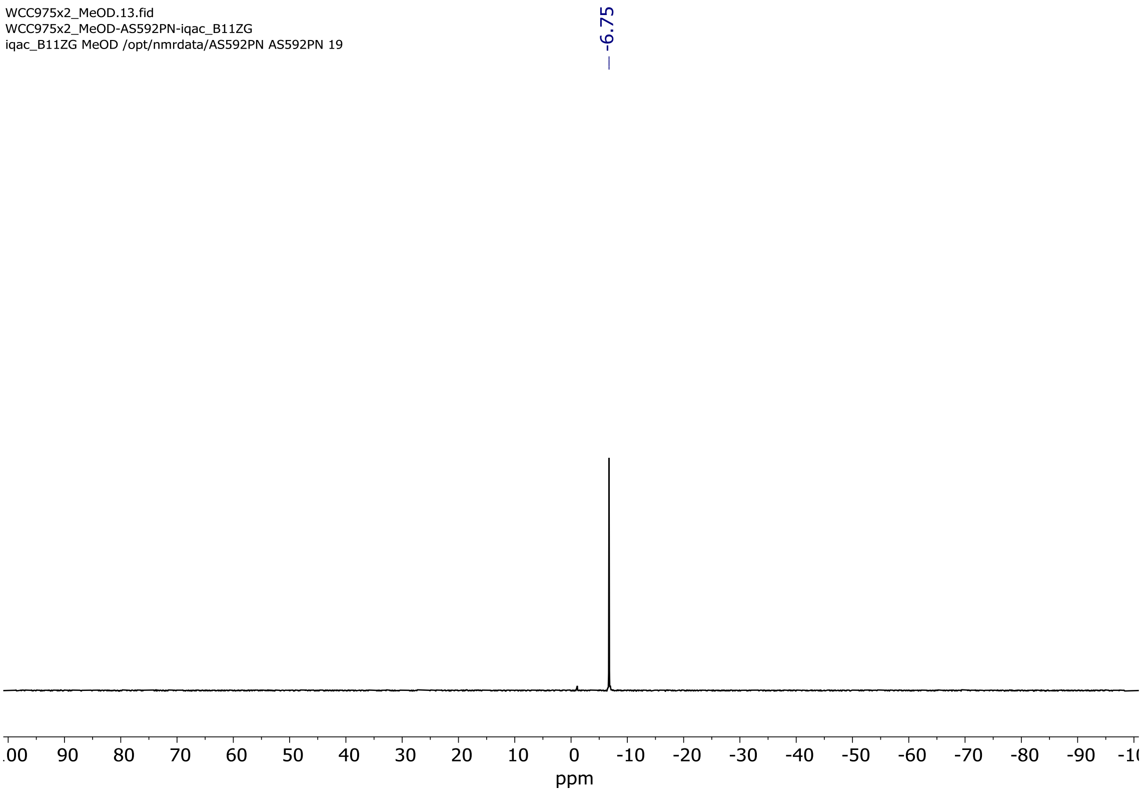

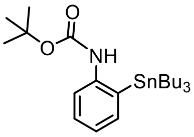

(**5**): ^{1}H NMR (400 MHz, $CDCl_3$)

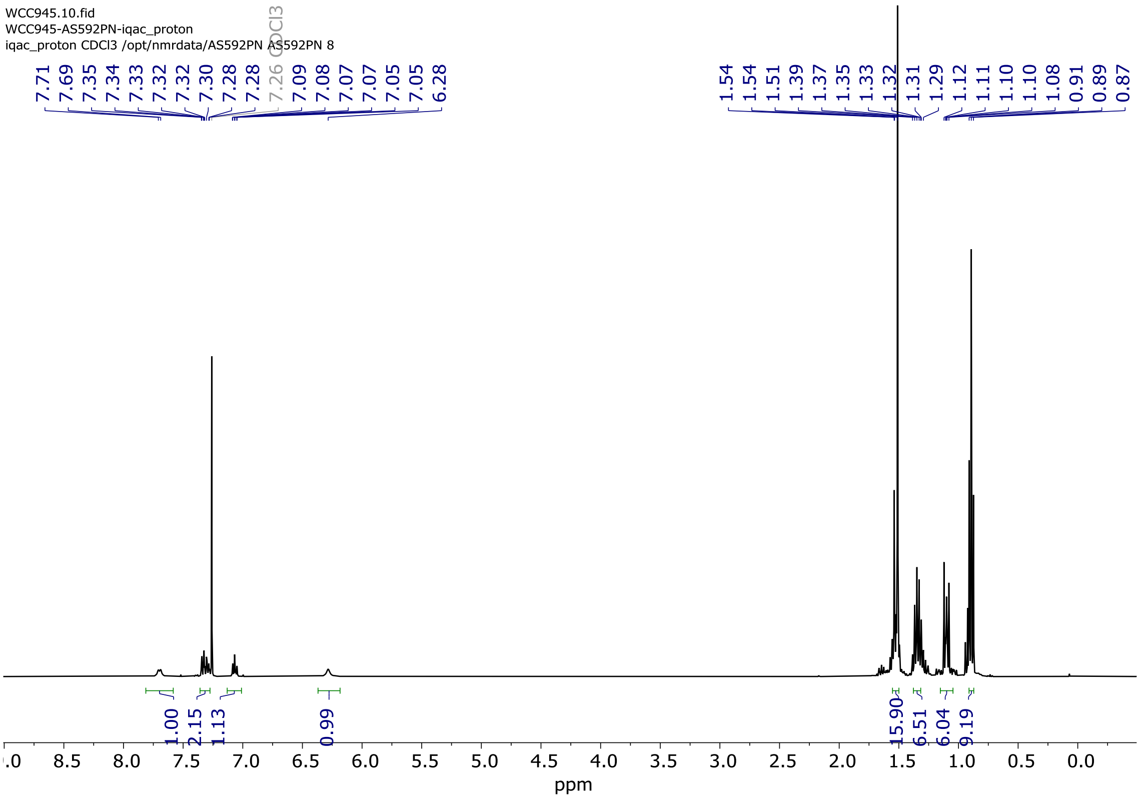

^{13}C NMR (101 MHz, $CDCl_3$)

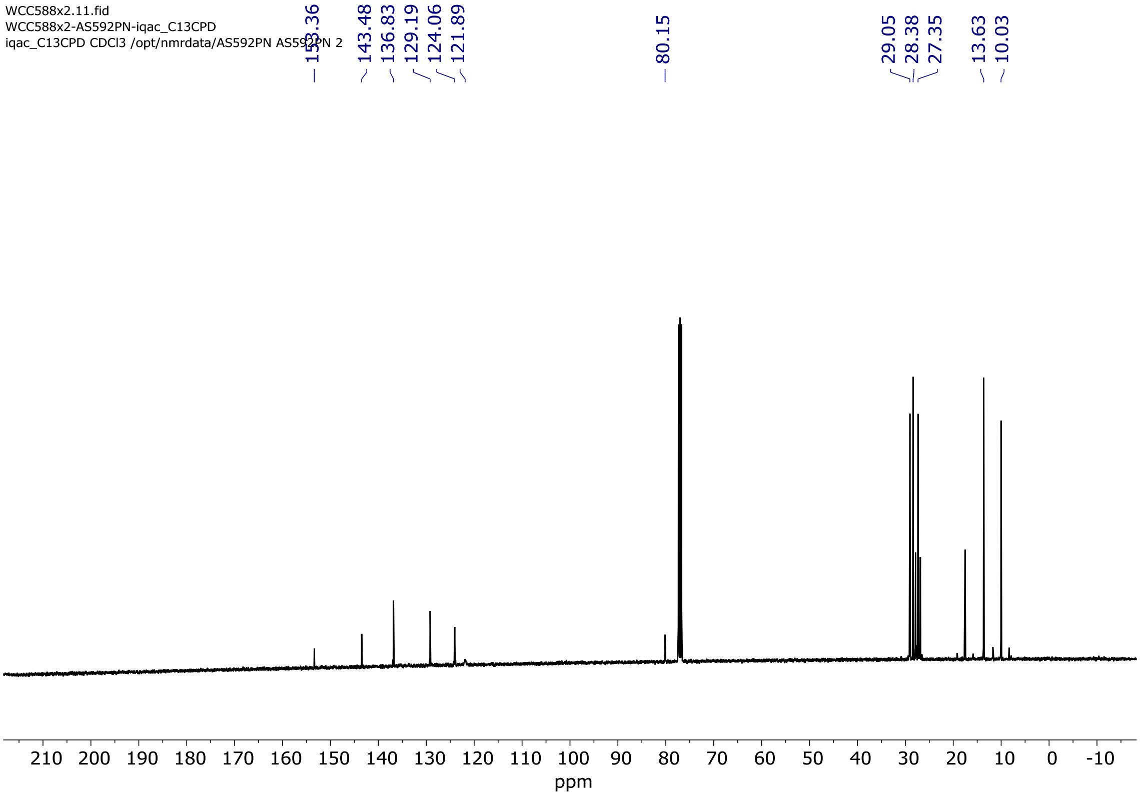

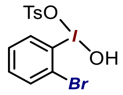

(**6a**): ^{1}H NMR (400 MHz, DMSO-d_6)

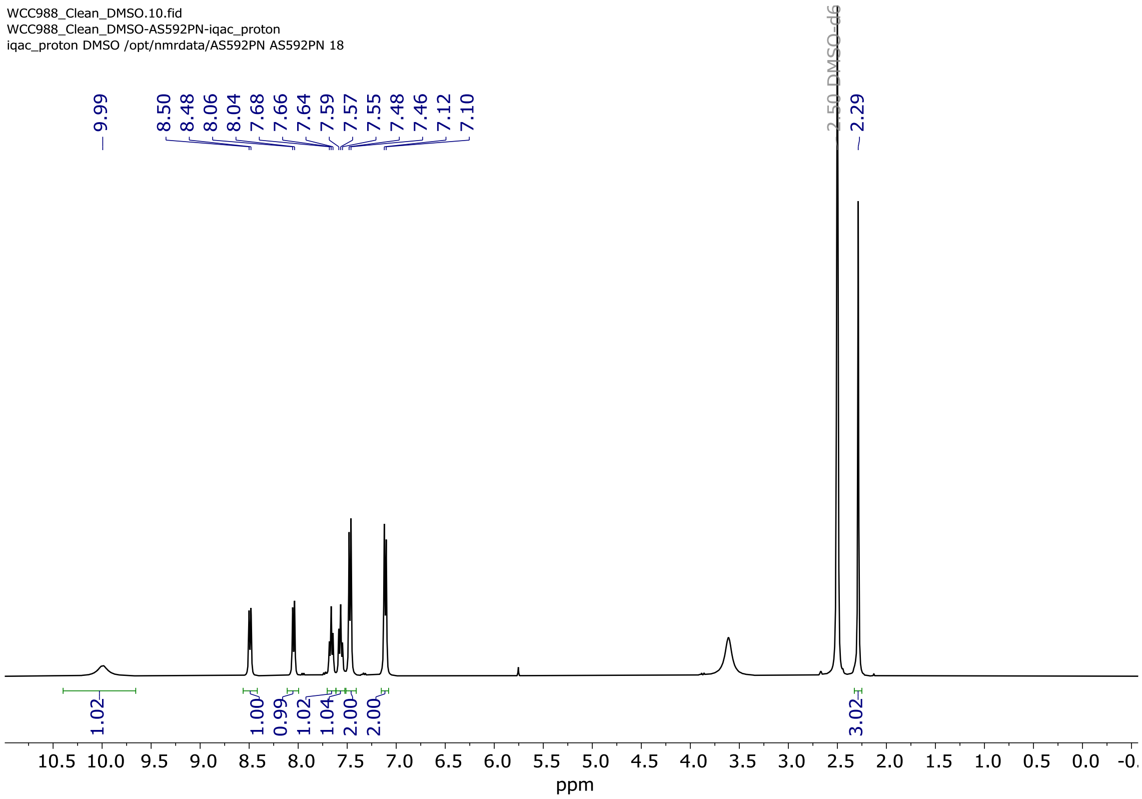

^{13}C NMR (101 MHz, DMSO-d_6)

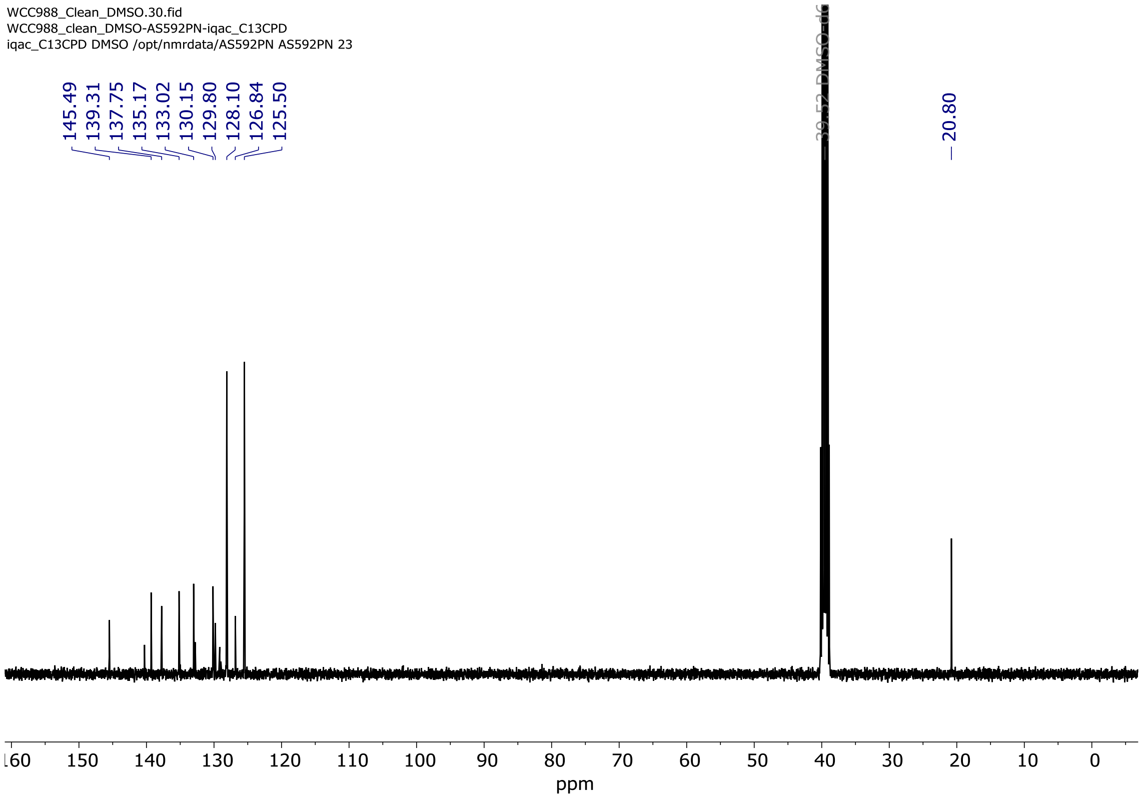

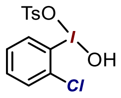

(**6b**): ^{1}H NMR (400 MHz, DMSO-d_6)

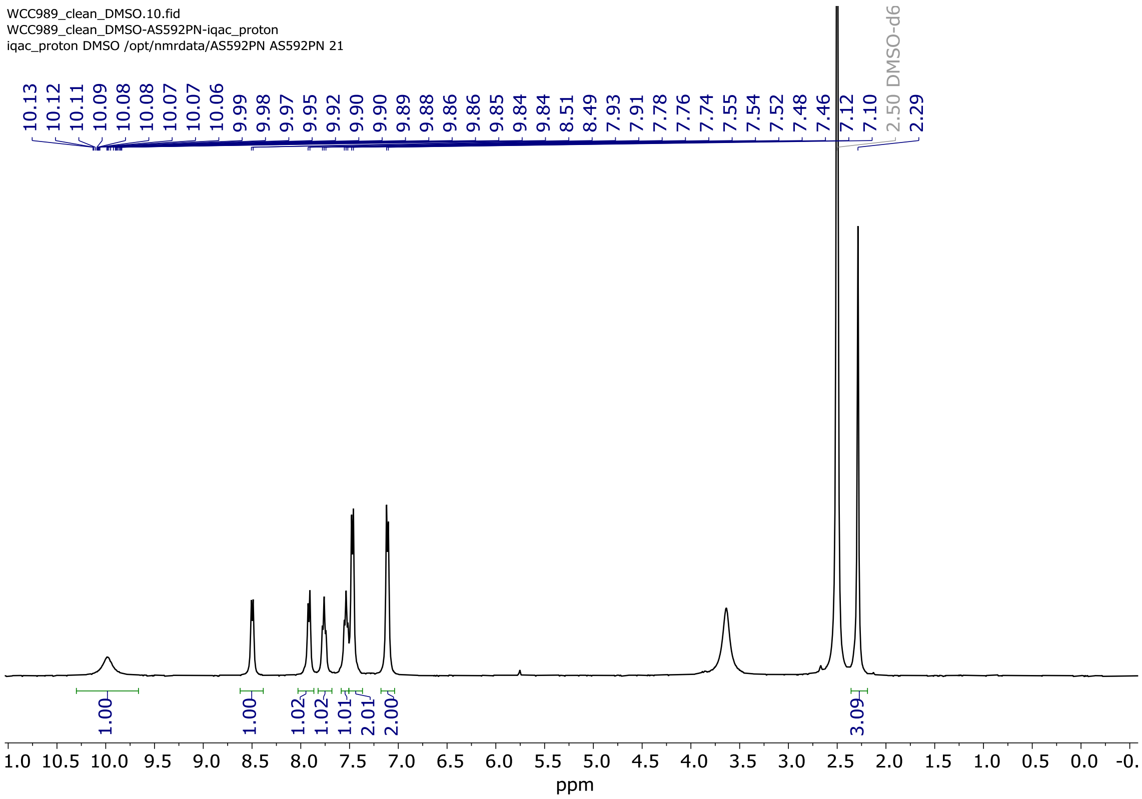

^{13}C NMR (101 MHz, DMSO-d_6)

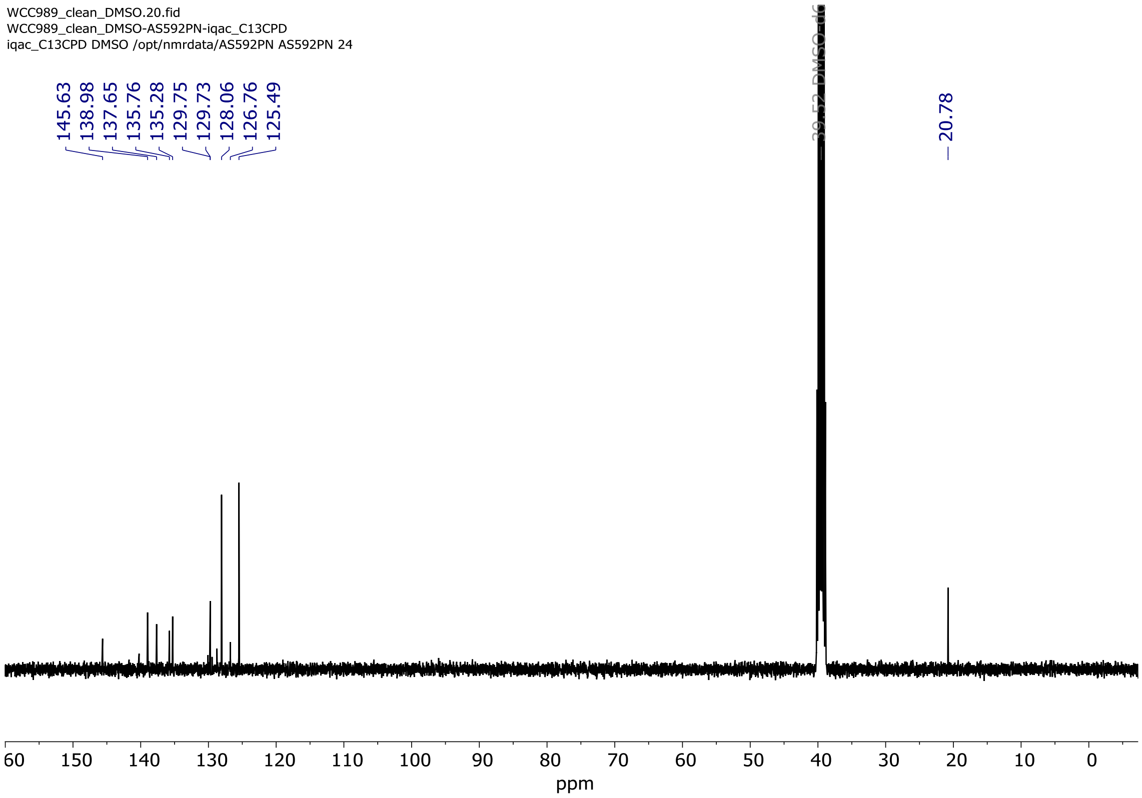

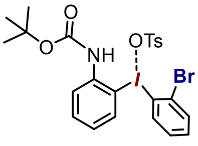

(**7a**): ^{1}H NMR (400 MHz, $CDCl_3$)

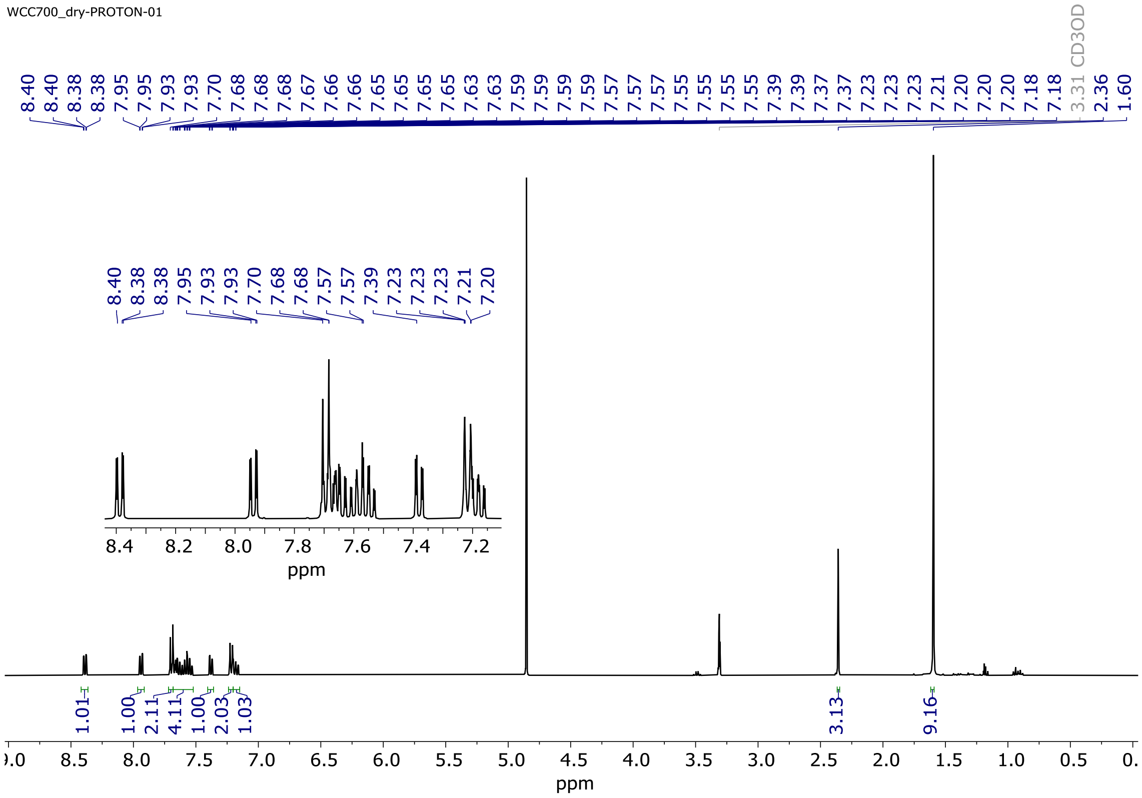

^{13}C NMR (101 MHz, $CDCl_3$)

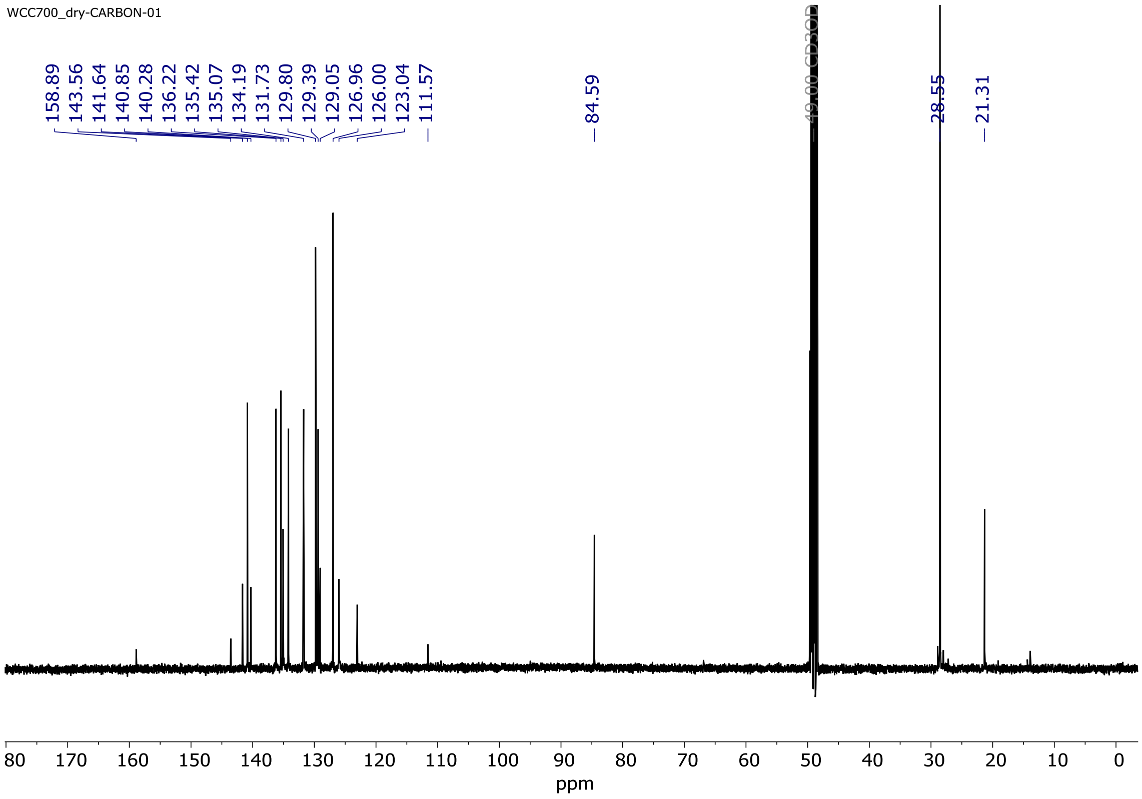

HSQCAD experiment in CD_3OD

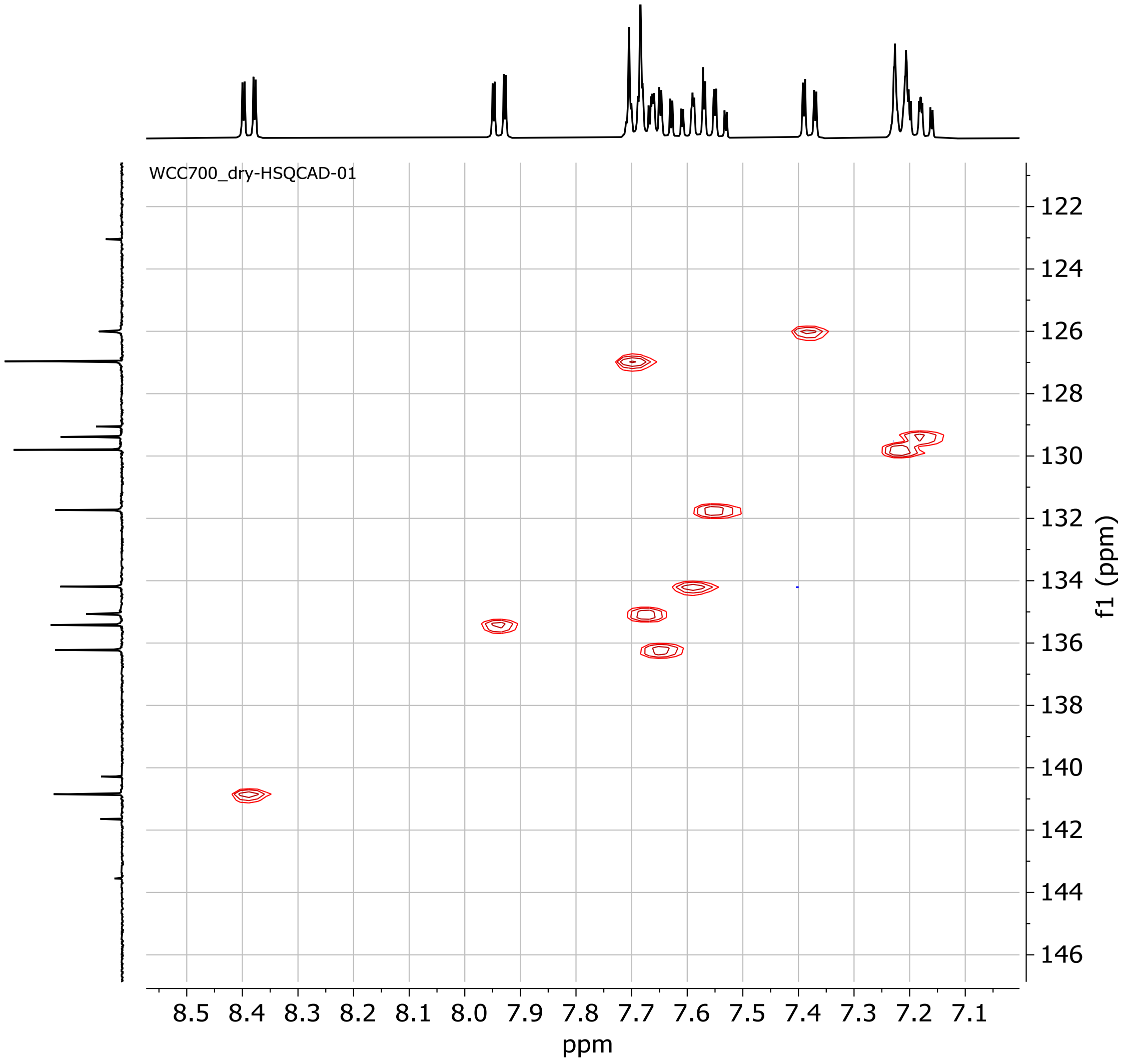

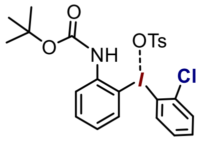

(**7b**): ^{1}H NMR (400 MHz, DMSO_d^{6})

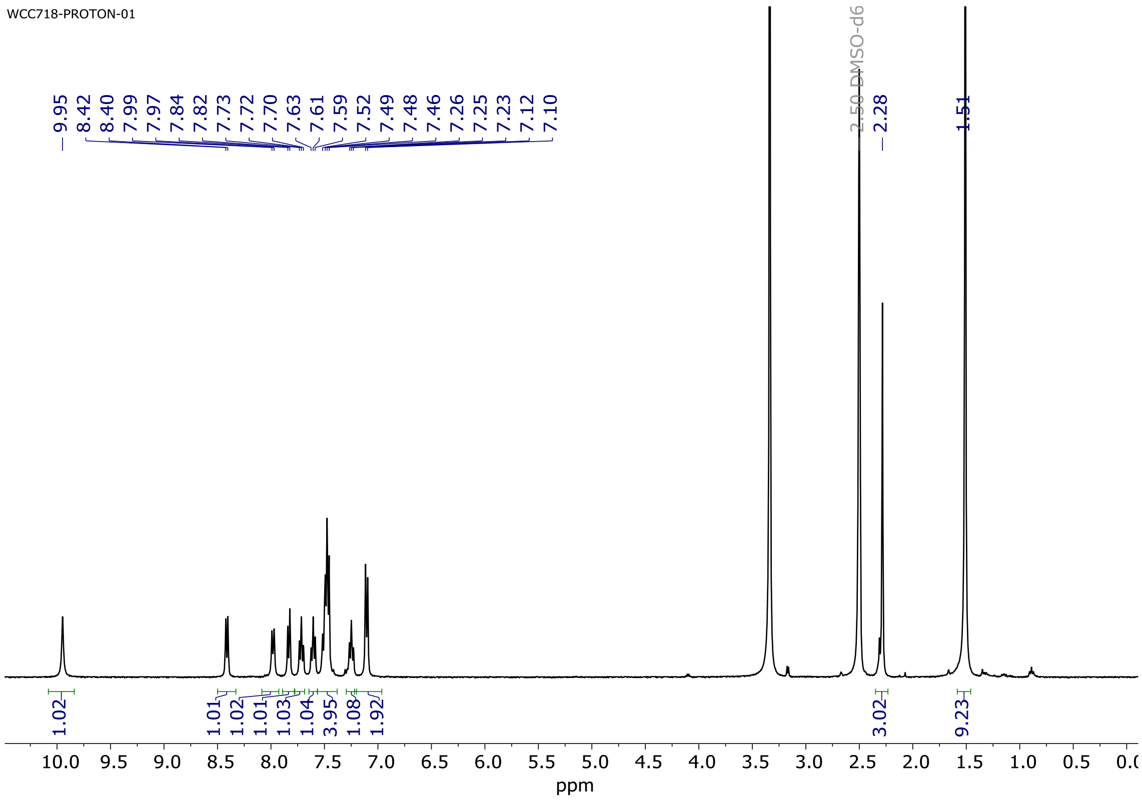

^{13}C NMR (101 MHz, DMSO_d^{6})

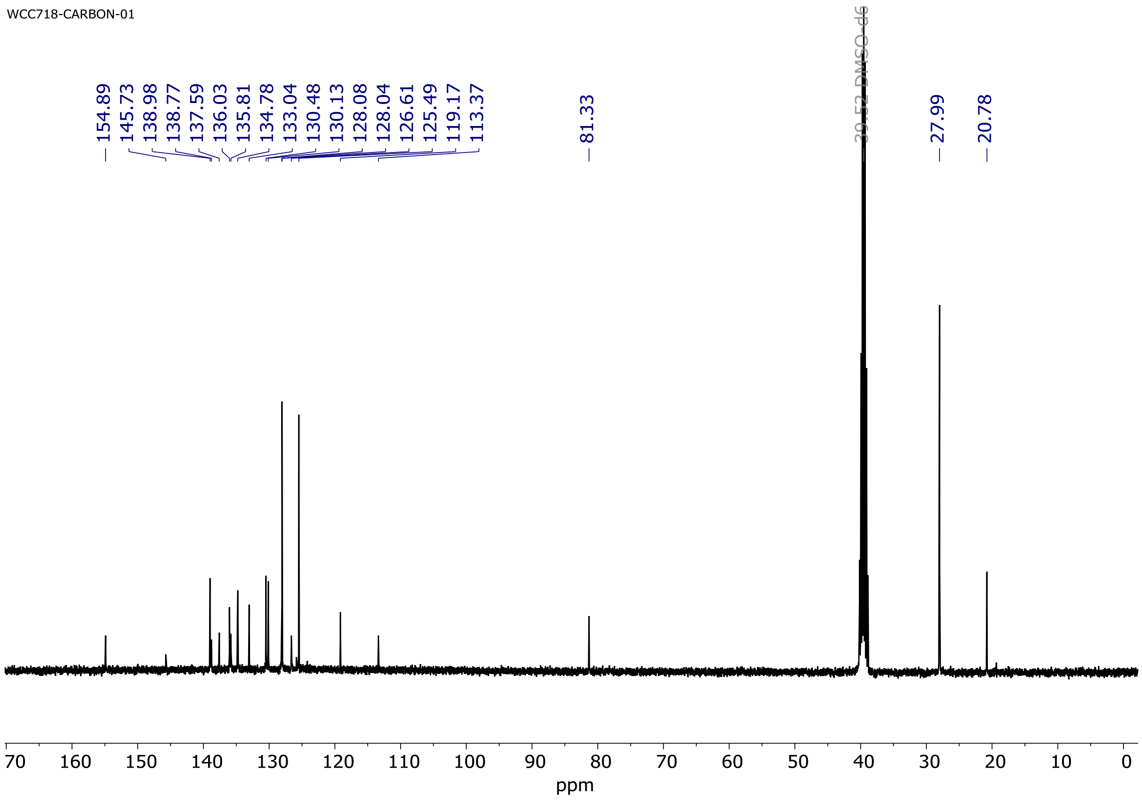

HSQCAD experiment in DMSO_d[6]

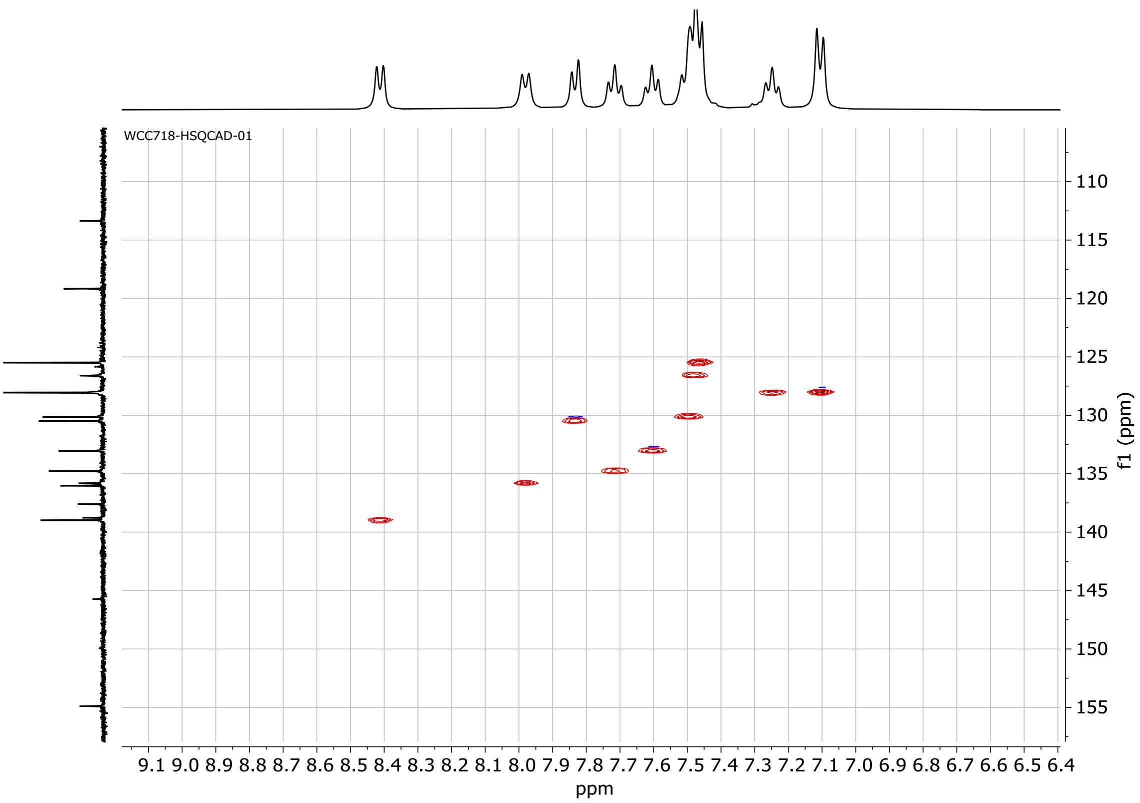

Gradient HMBCAD experiment in DMSO-d_6

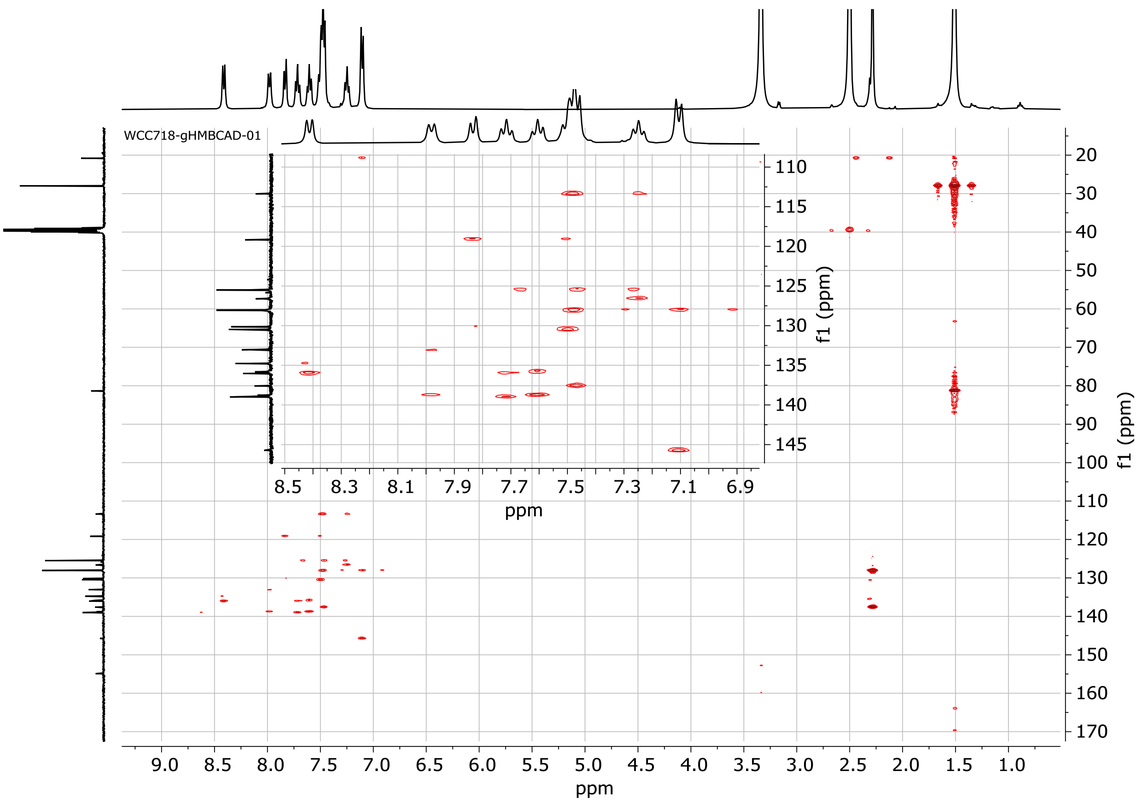

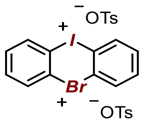

8a-$(OTs)_2$: ^1H NMR (400 MHz, D_2O)

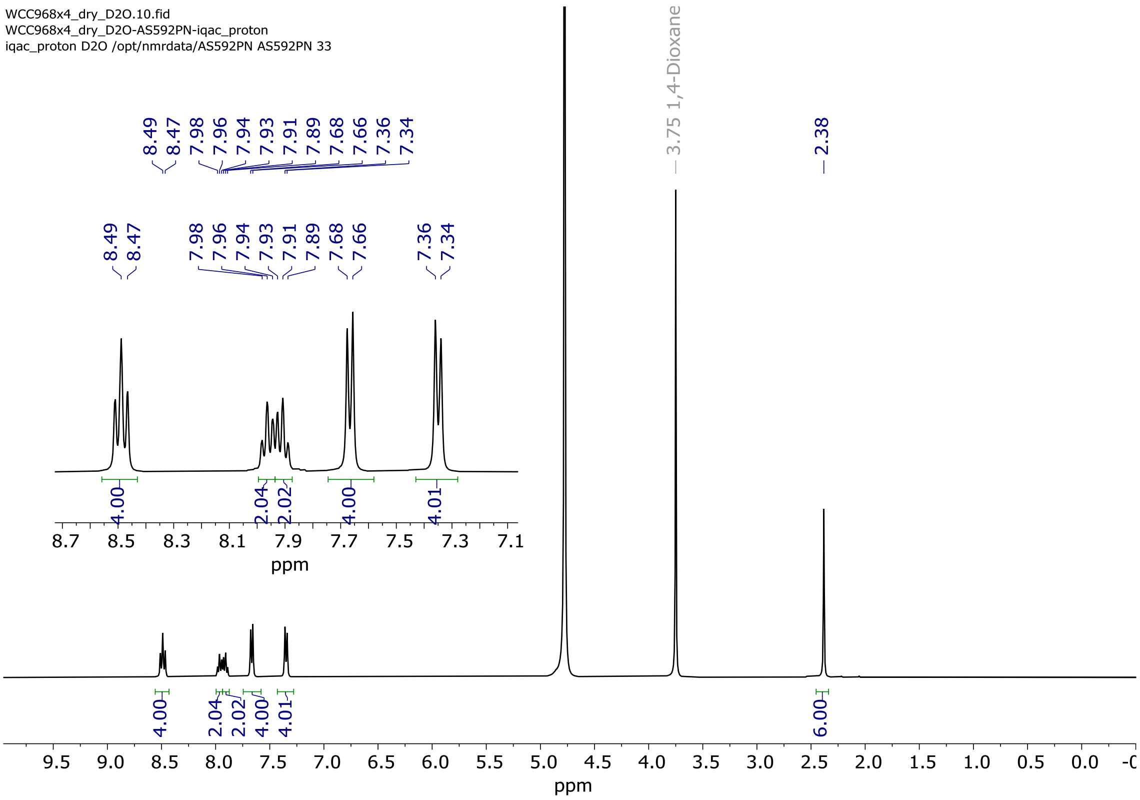

^{13}C NMR (101 MHz, D_2O)

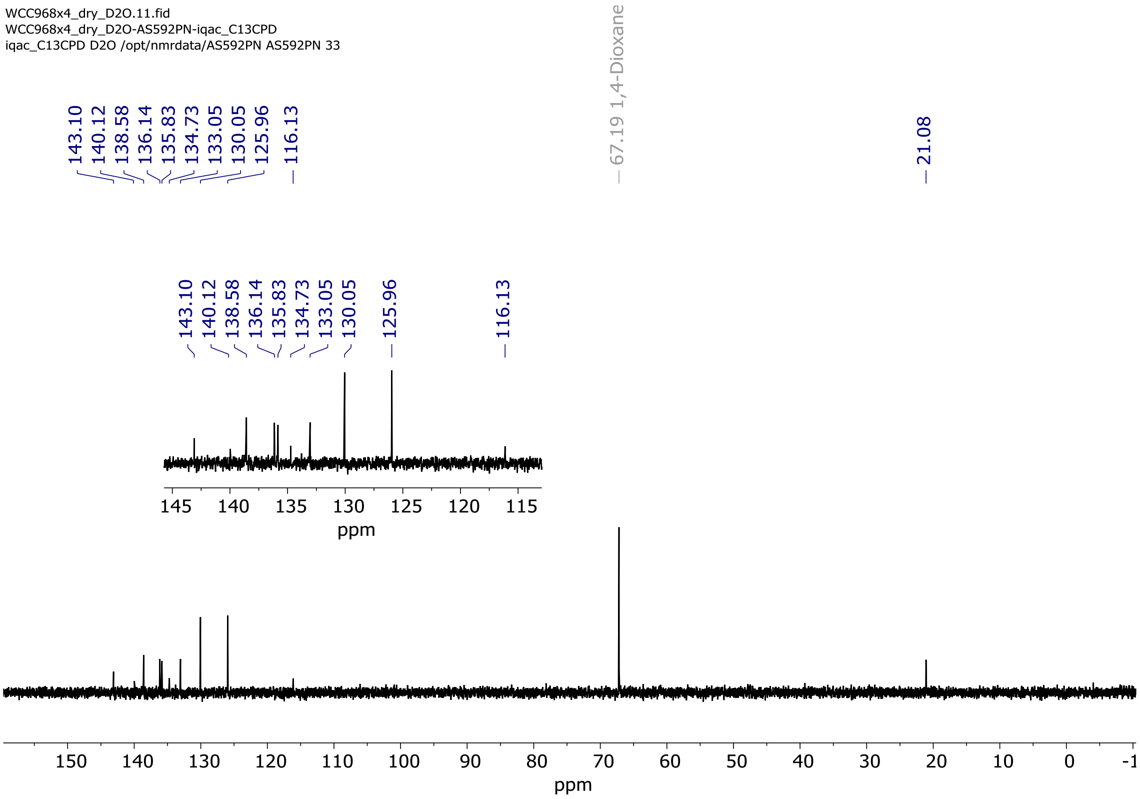

^{1}H-^{13}C HSQCEDETGP experiment in D_2O

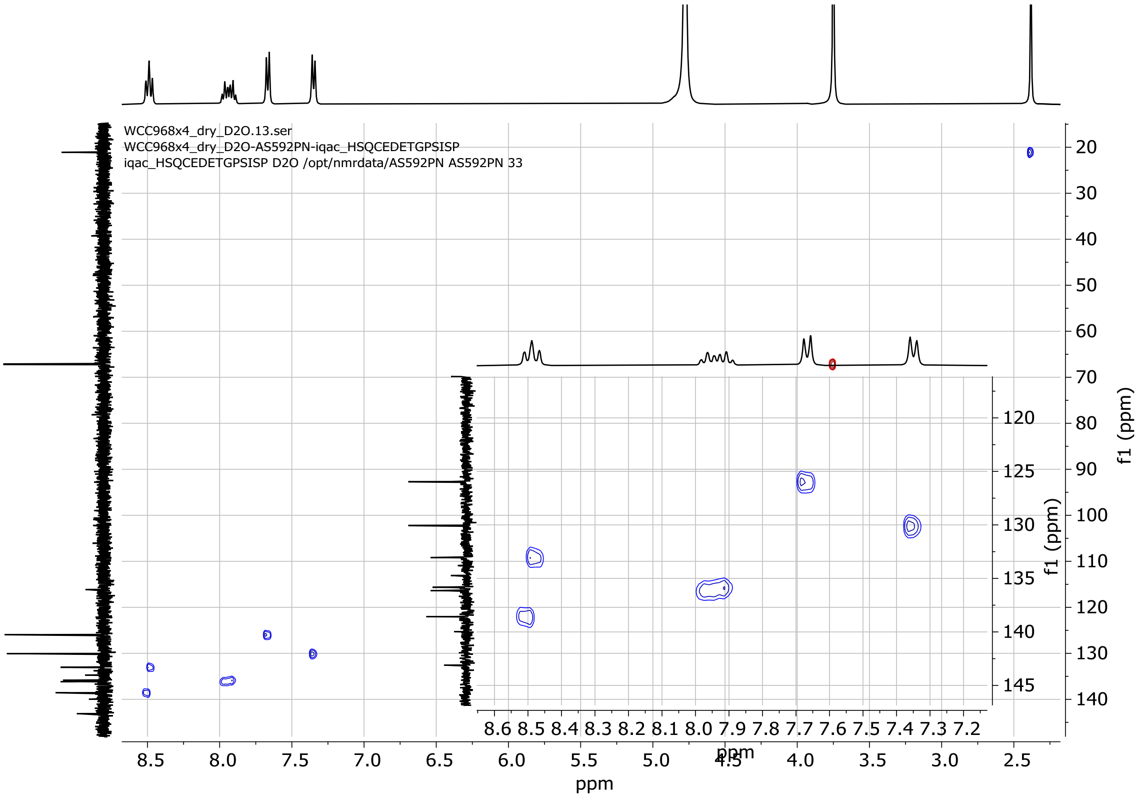

^{1}H-^{13}C HMBCGP experiment in D_2O

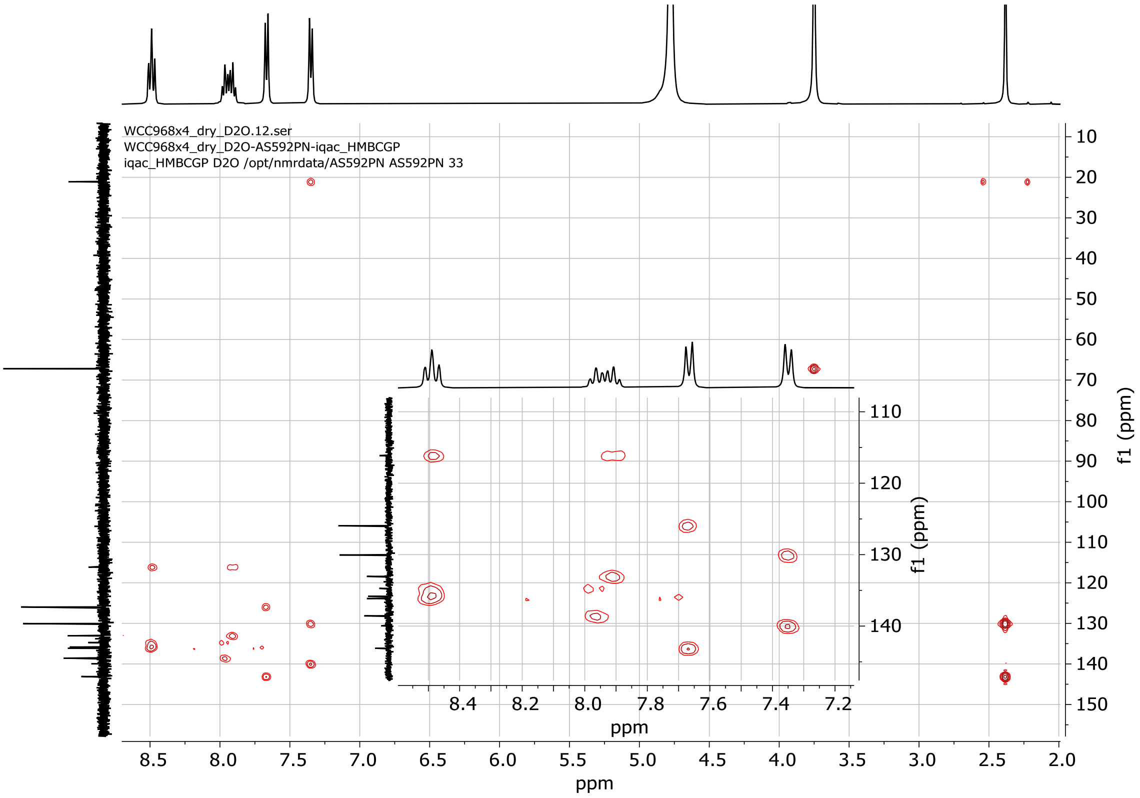

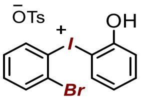

(**S1a**): ^{1}H NMR (400 MHz, DMSO-d^{6})

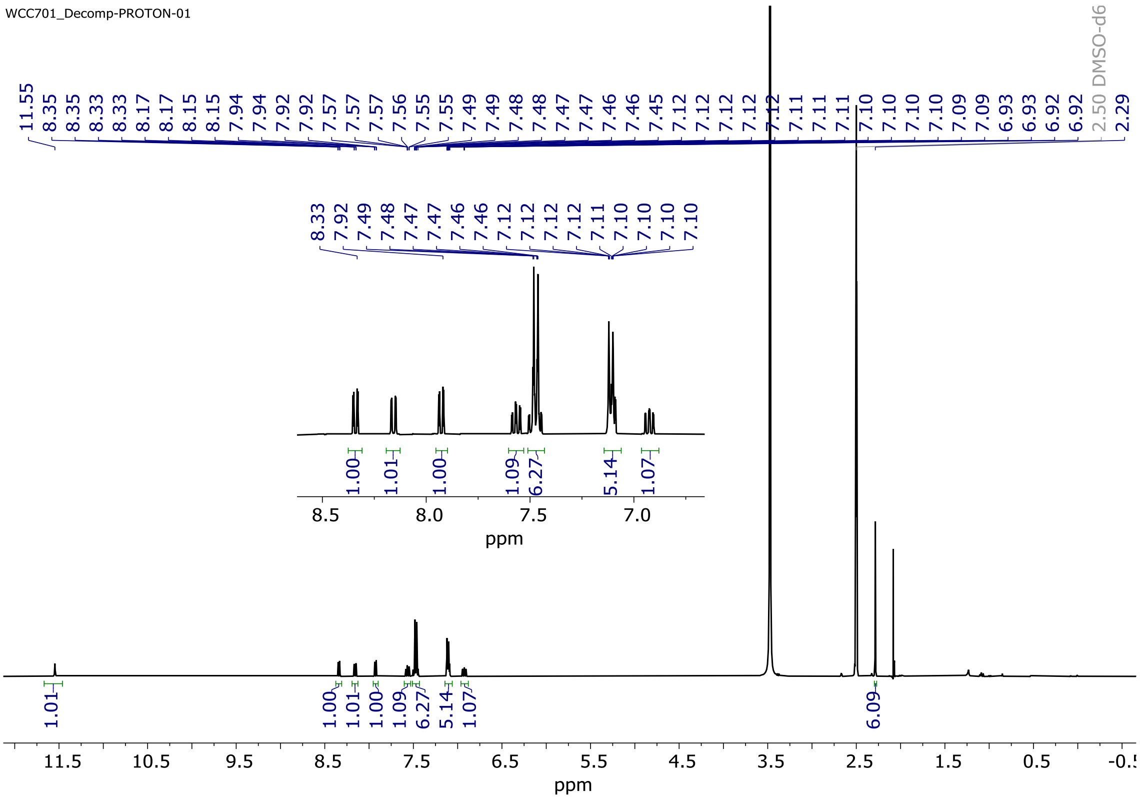

^{13}C NMR (101 MHz, DMSO-d^{6})

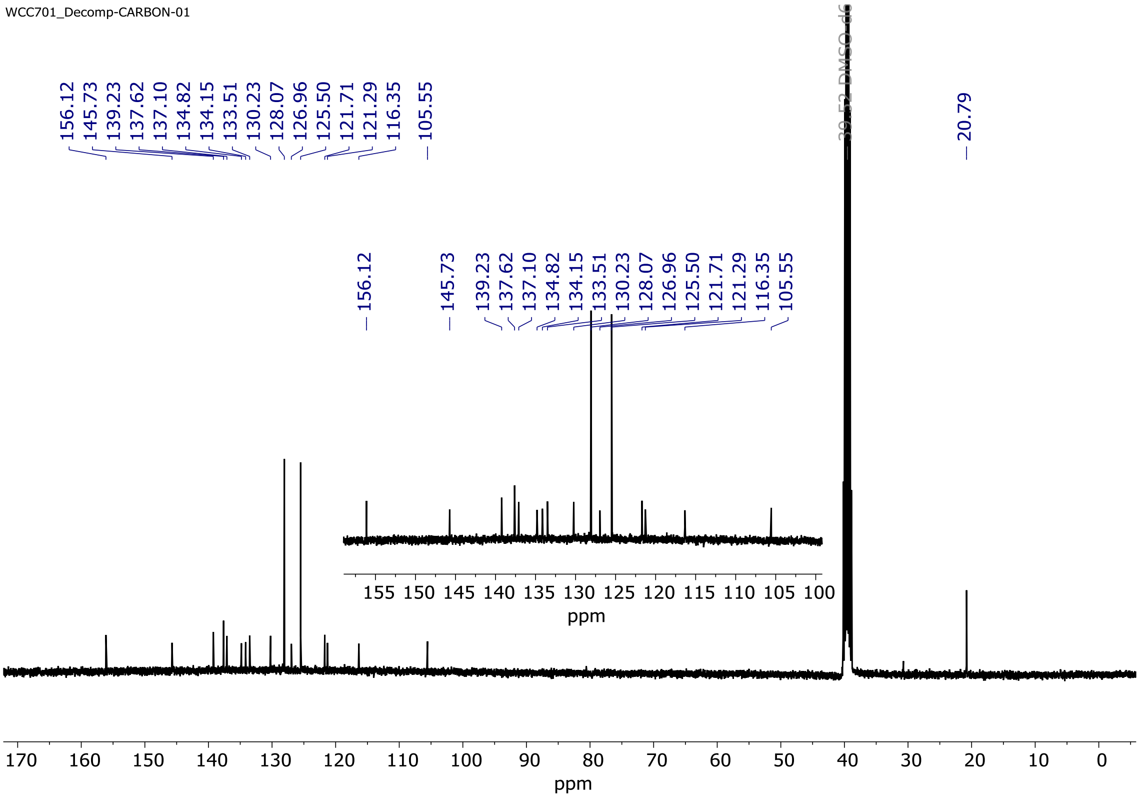

^{1}H-^{13}C HSQCEDETGP experiment in DMSO-d^6

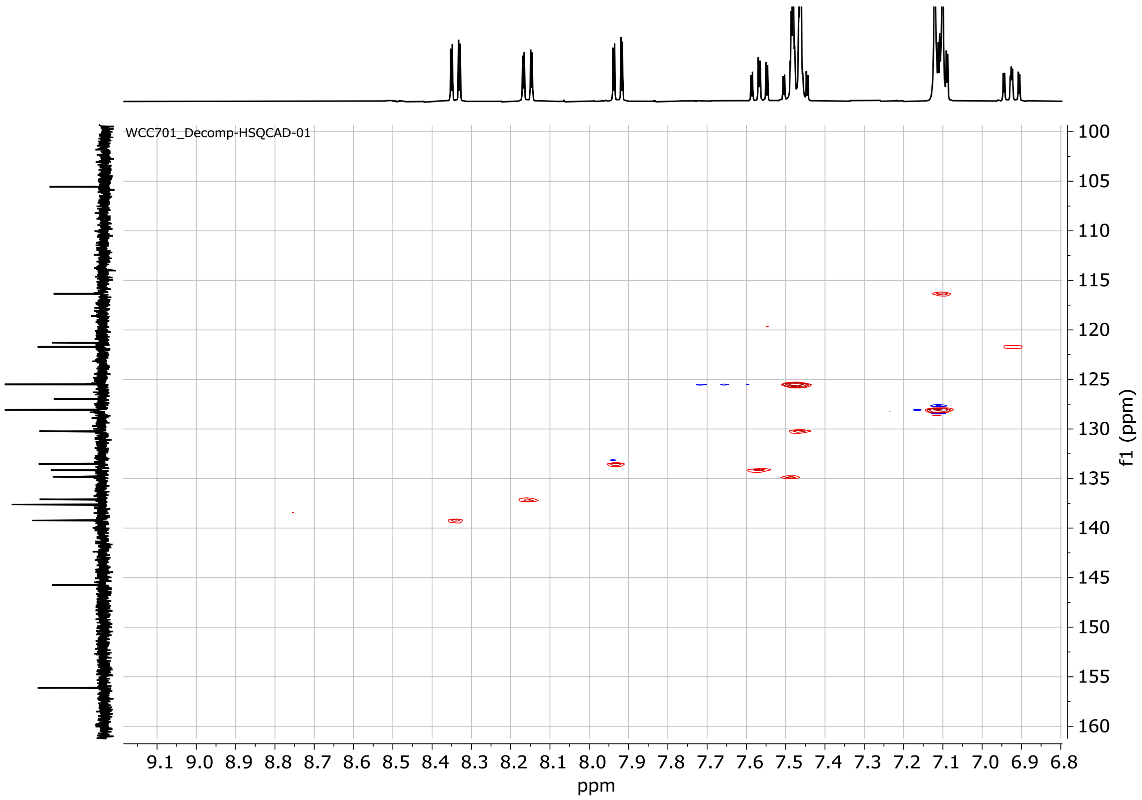

^{1}H-^{13}C HMBCGP experiment in DMSO-d^6

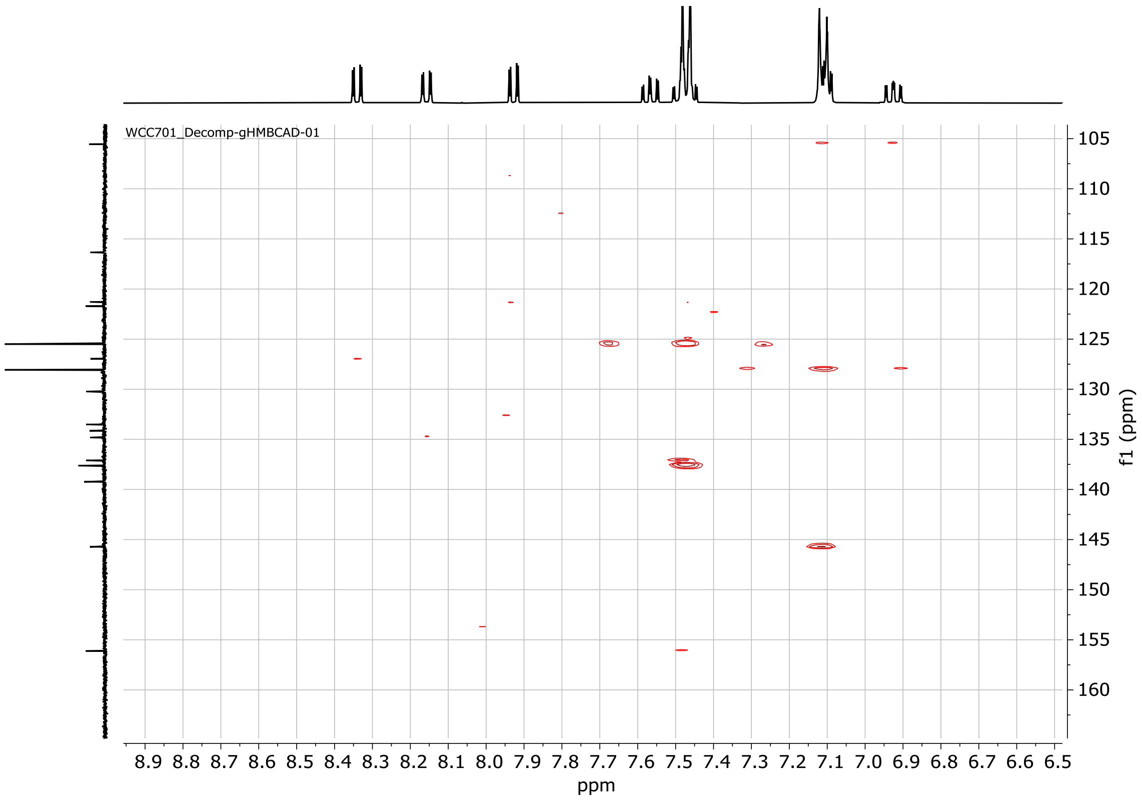

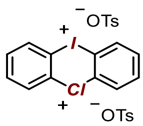

8b-$(OTs)_2$: ^1H NMR (400 MHz, D_2O)

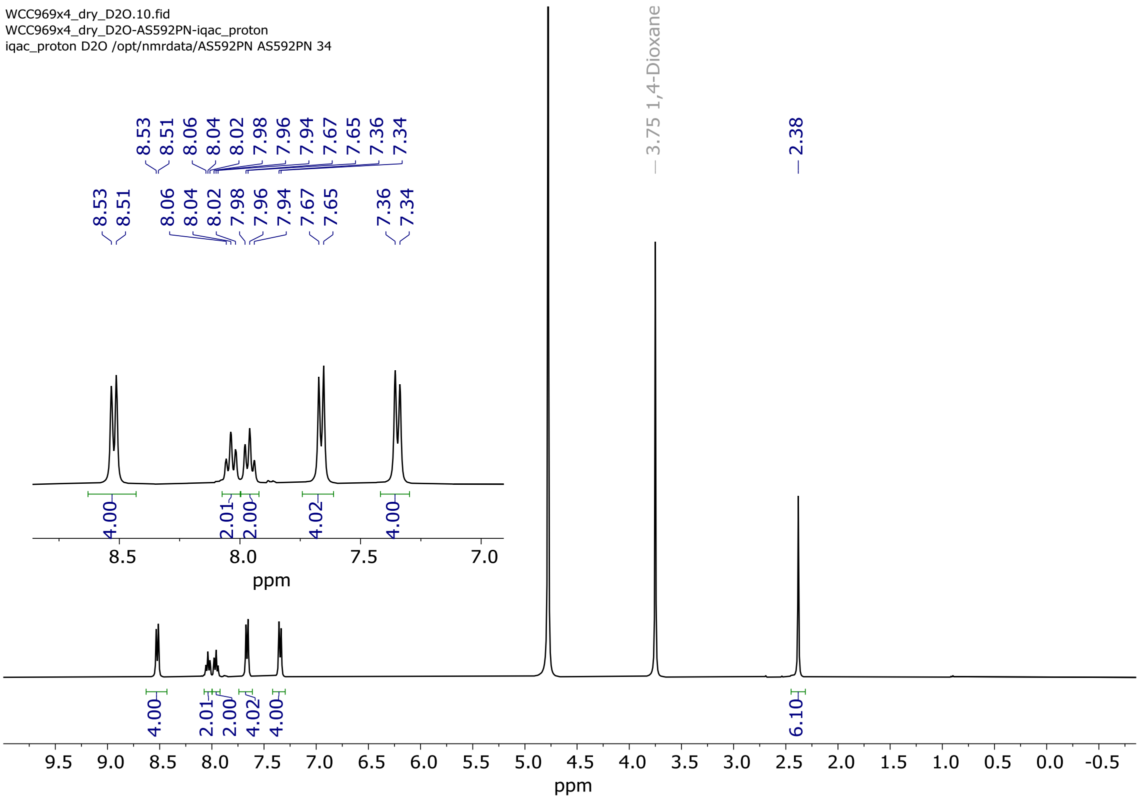

^{13}C NMR (101 MHz, D_2O)

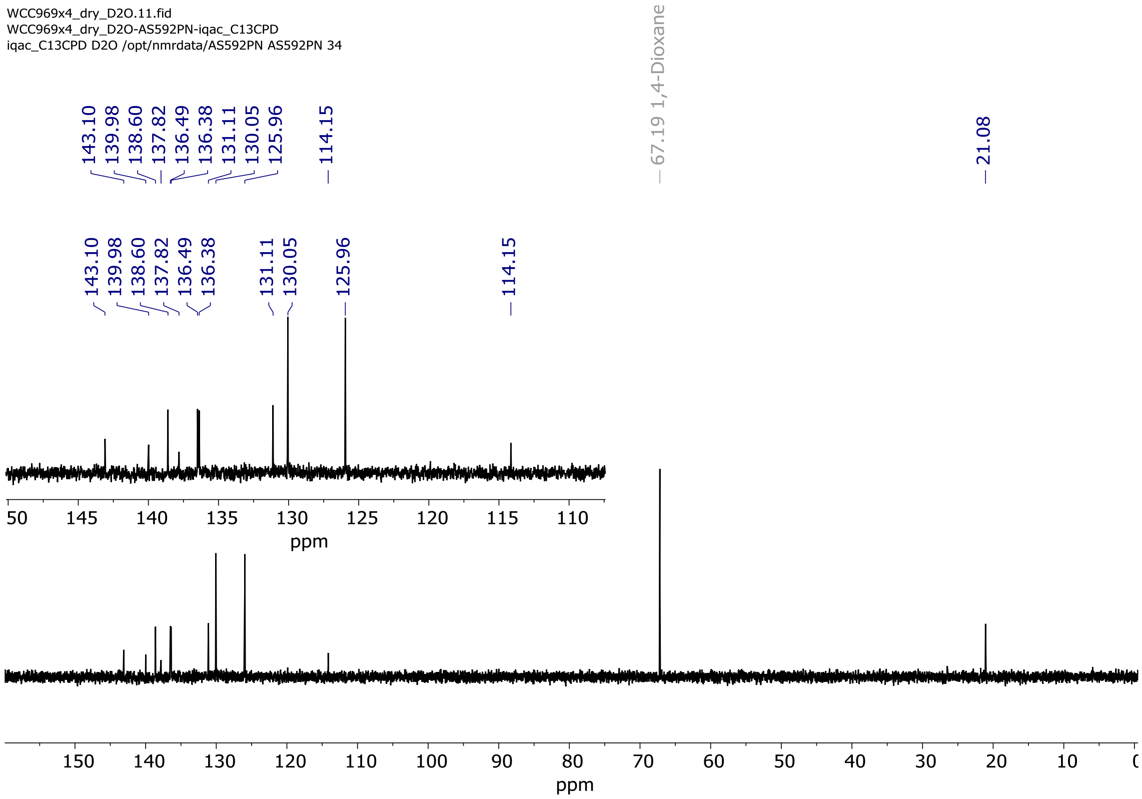

^{1}H-^{13}C HSQCEDETGP experiment in D_2O

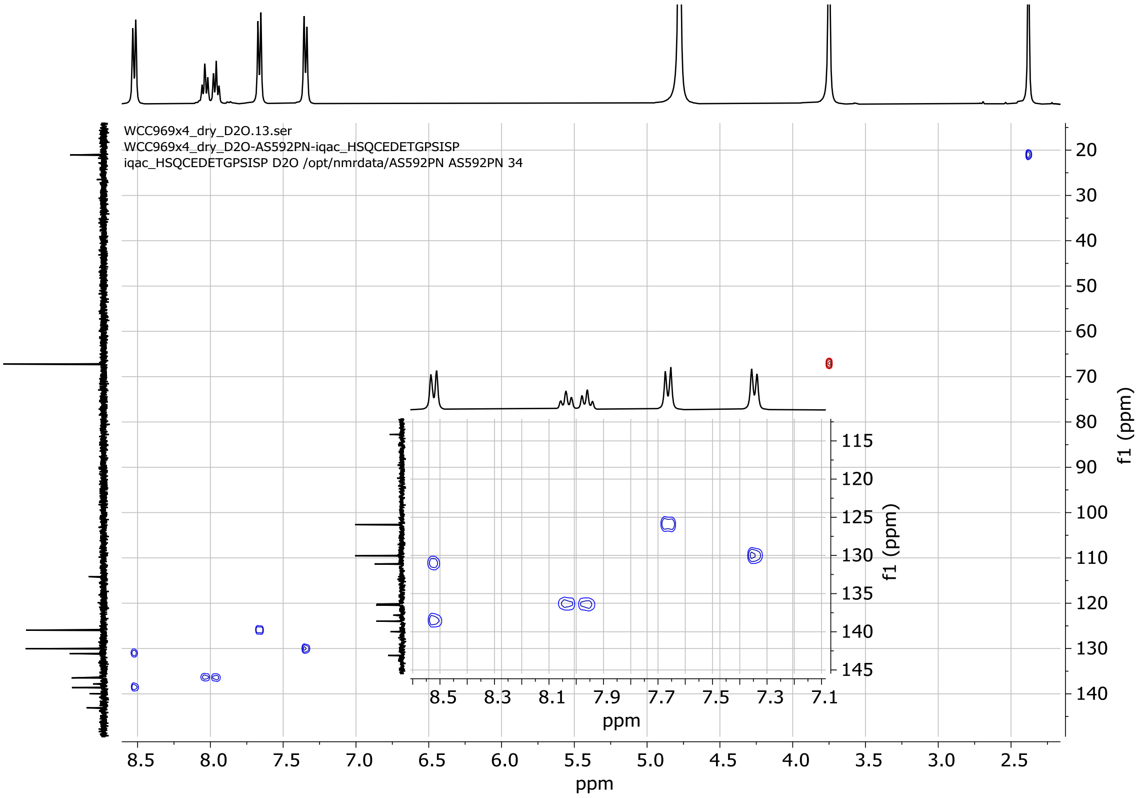

^{1}H-^{13}C HMBCGP experiment in D_2O

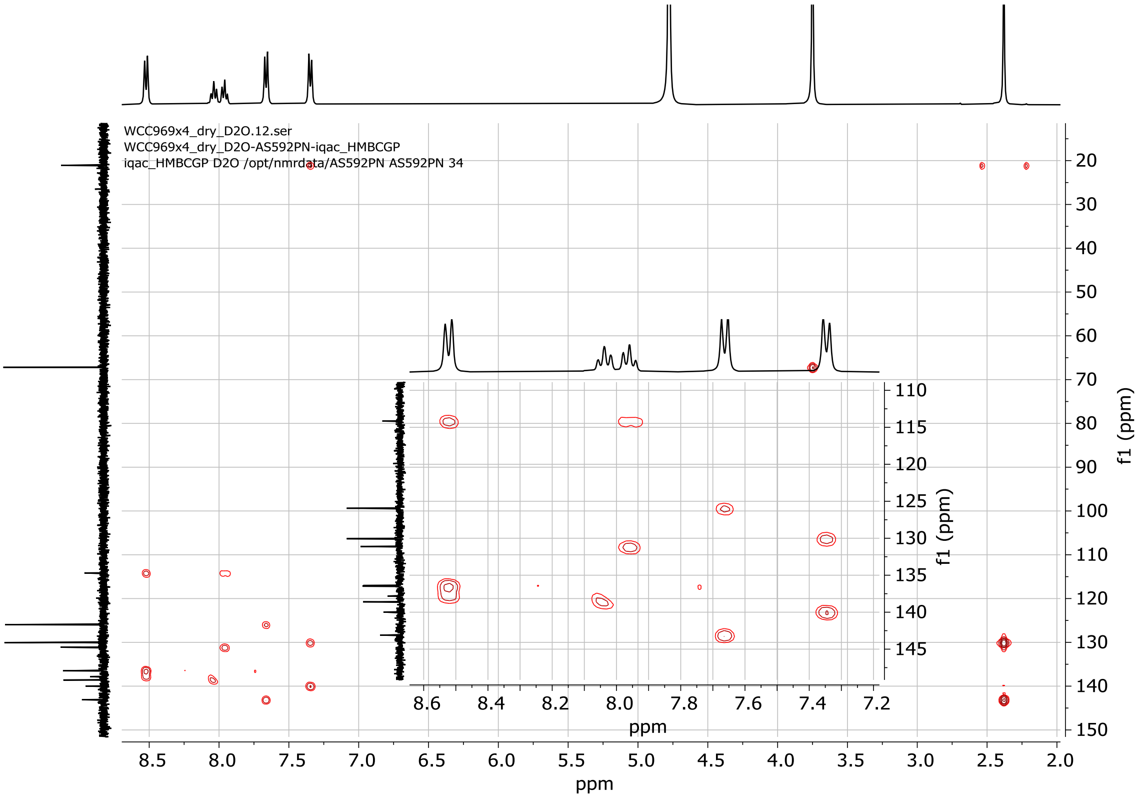

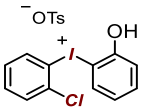

(**S1b**): ^{1}H NMR (400 MHz, DMSO-d^{6})

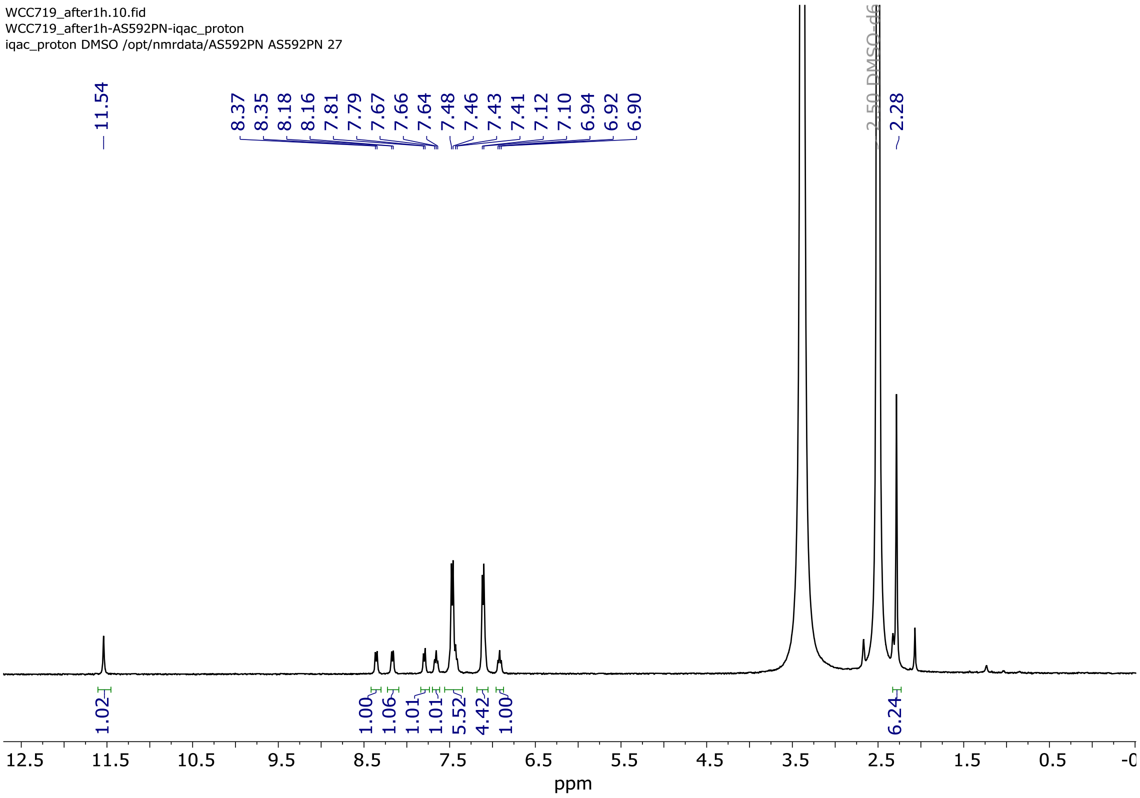

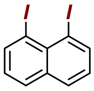

1,8-diiodonaphthalene: ^{1}H NMR (400 MHz, $CDCl_3$)

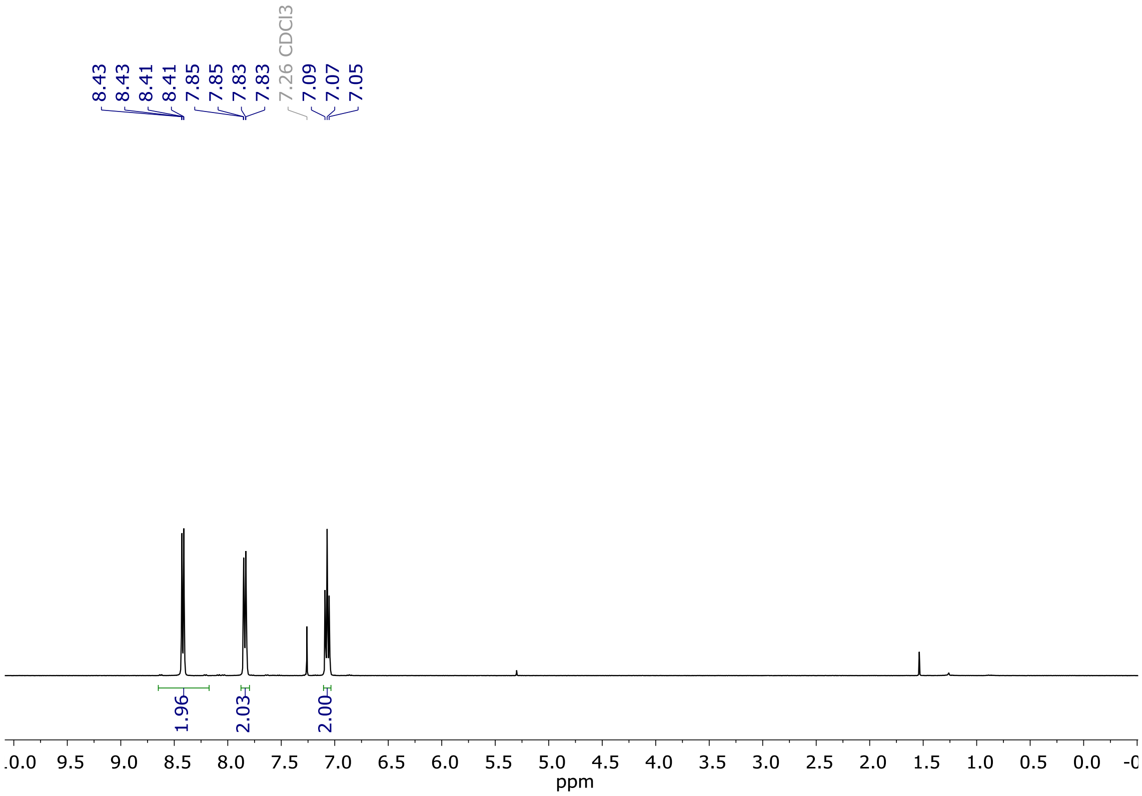

^{13}C NMR (101 MHz, $CDCl_3$)

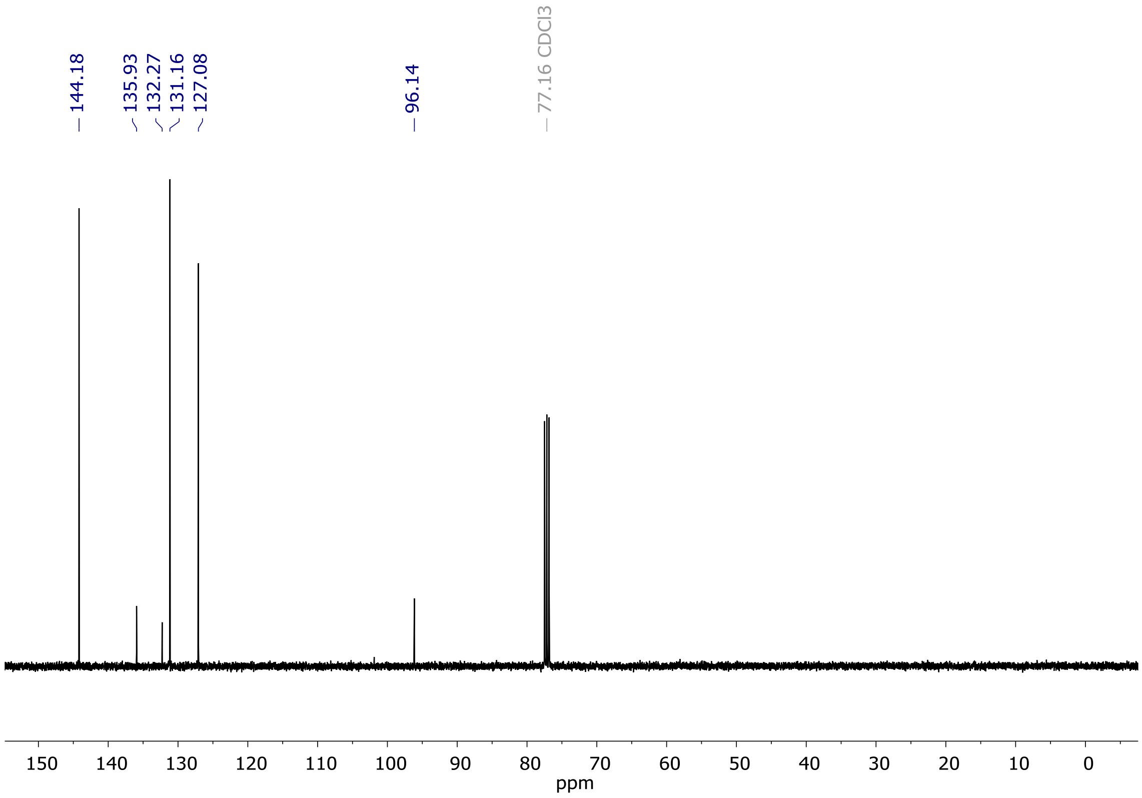

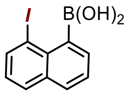

(8-iodonaphthalen-1-yl)boronic acid: ^{1}H NMR (400 MHz, Methanol-d_4)

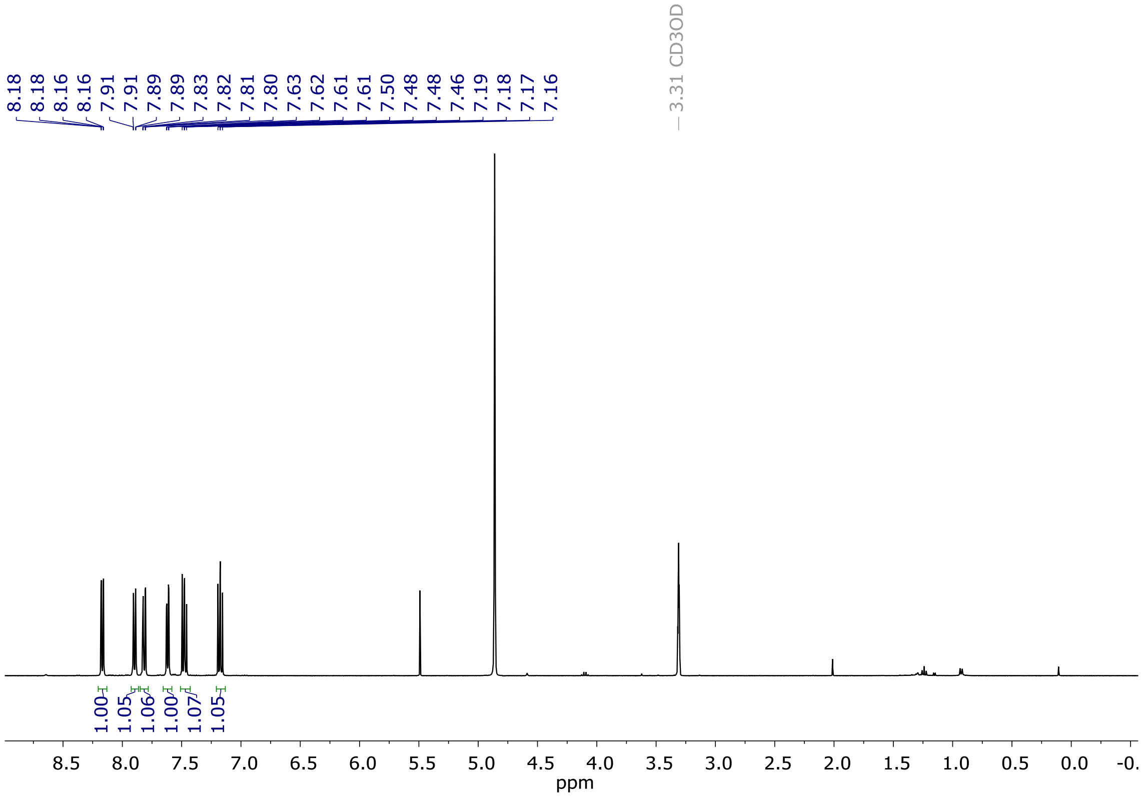

^{13}C NMR (101 MHz, Methanol-d_4)

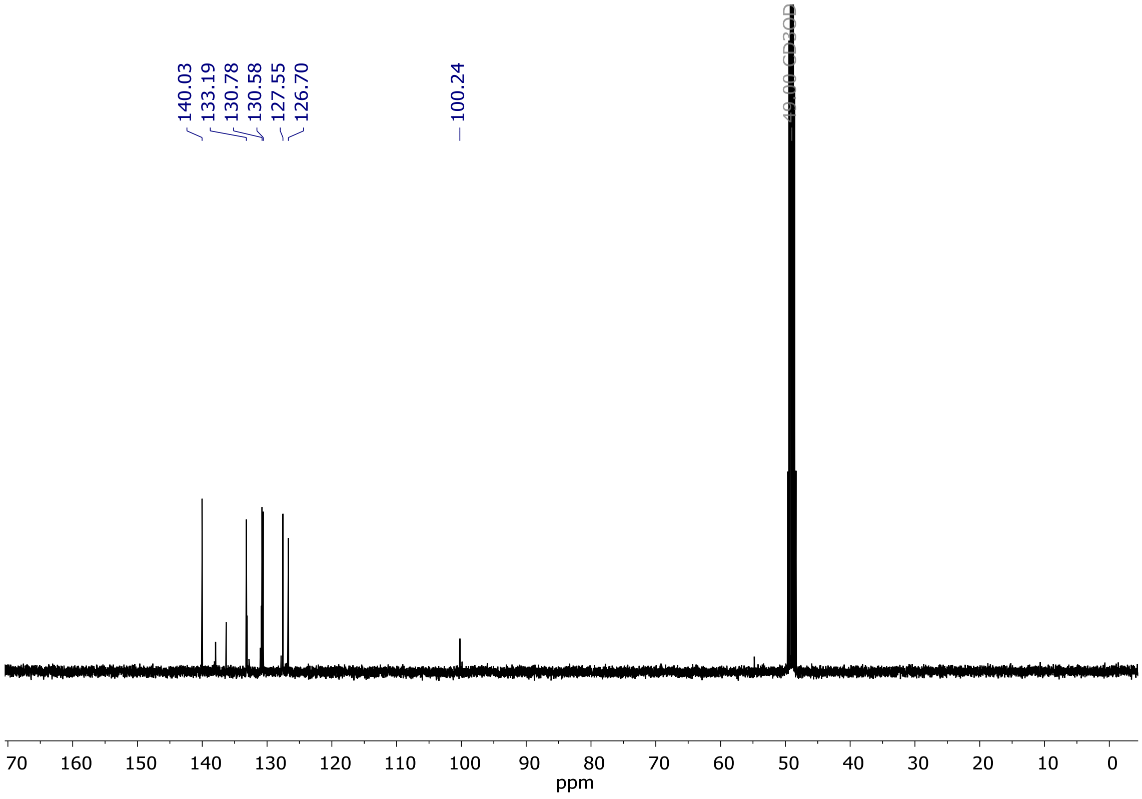

HSQCAD experiment in Methanol-d_4

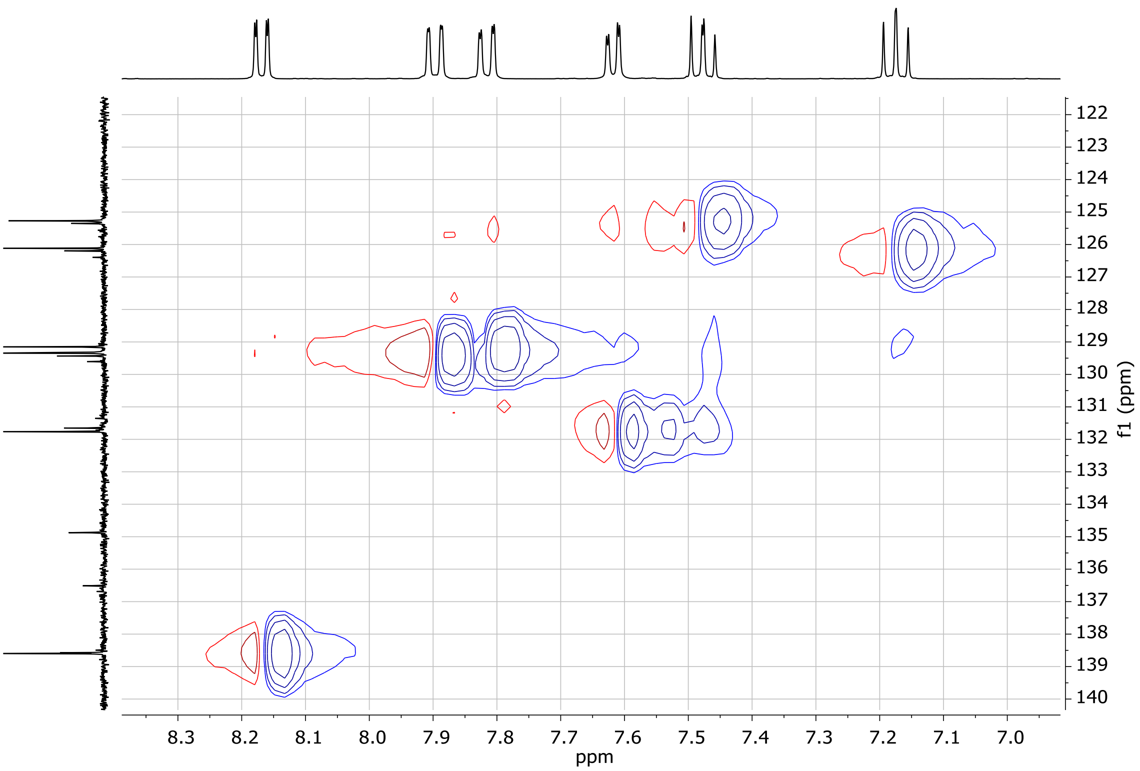

Gradient COSY ^1H-^1H experiment in Methanol-d_4

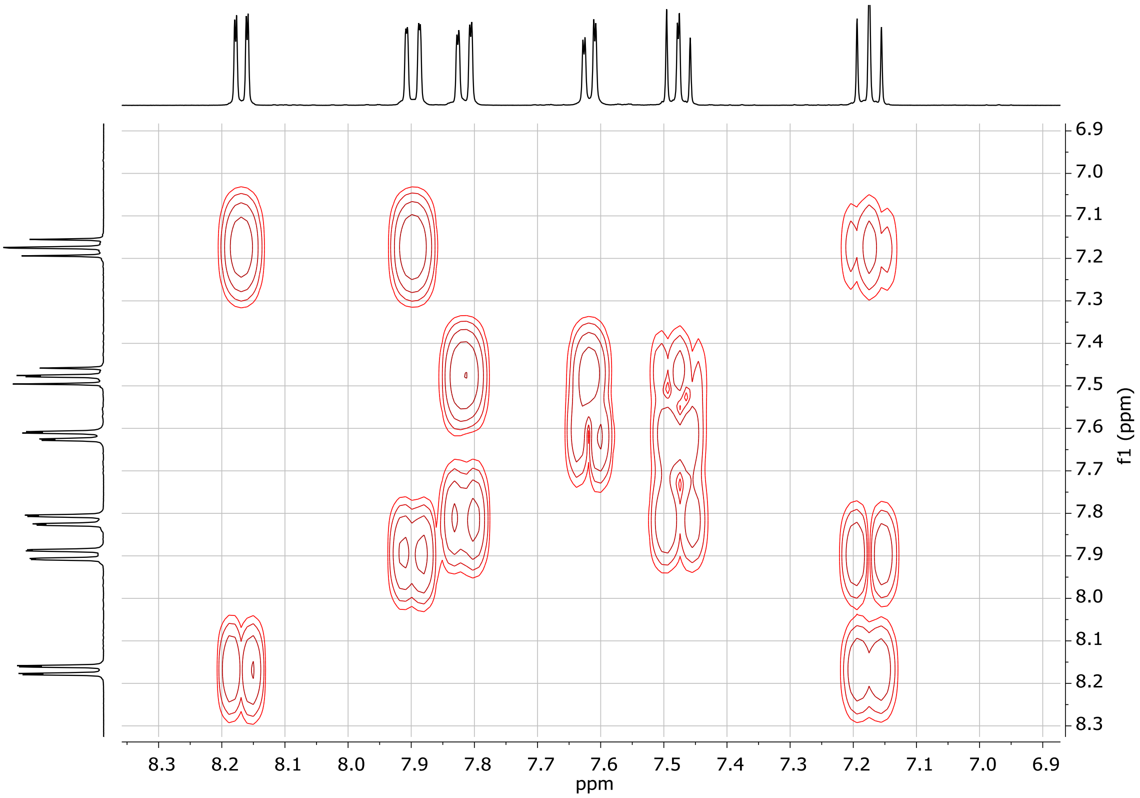

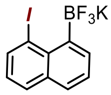

(**10**): ^{1}H NMR (400 MHz, DMSO-d_6)

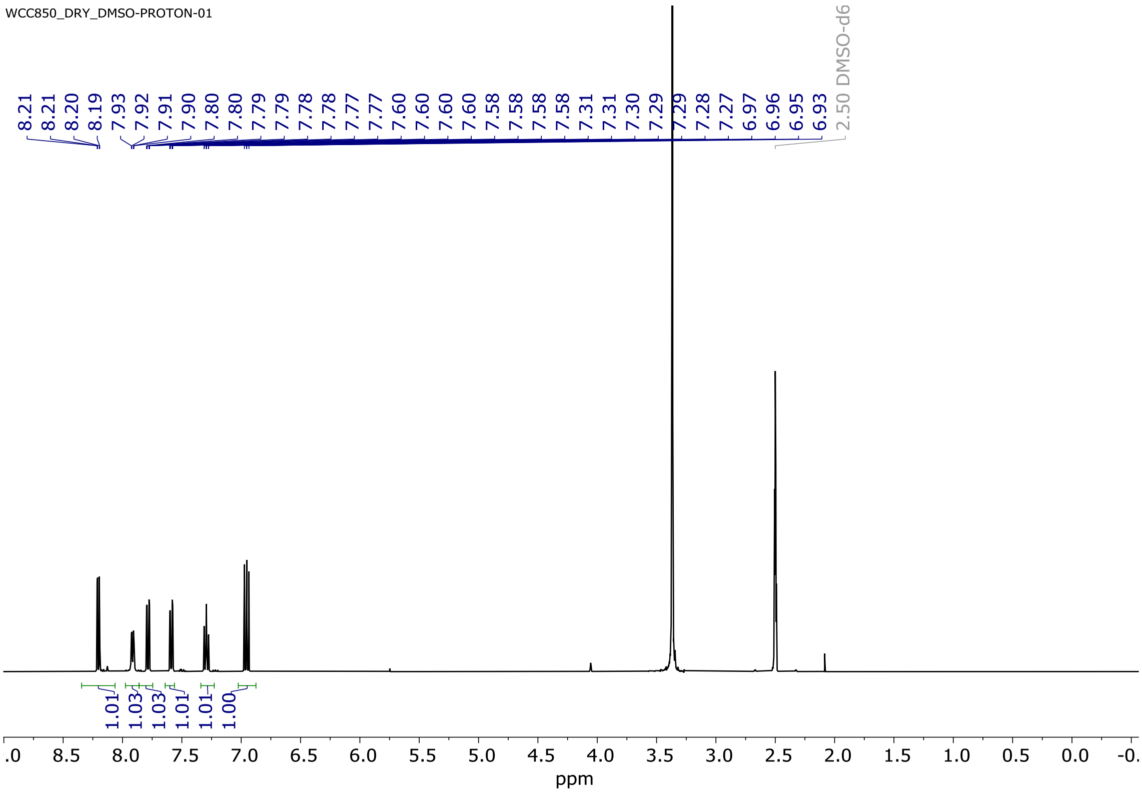

^{13}C NMR (101 MHz, DMSO-d_6)

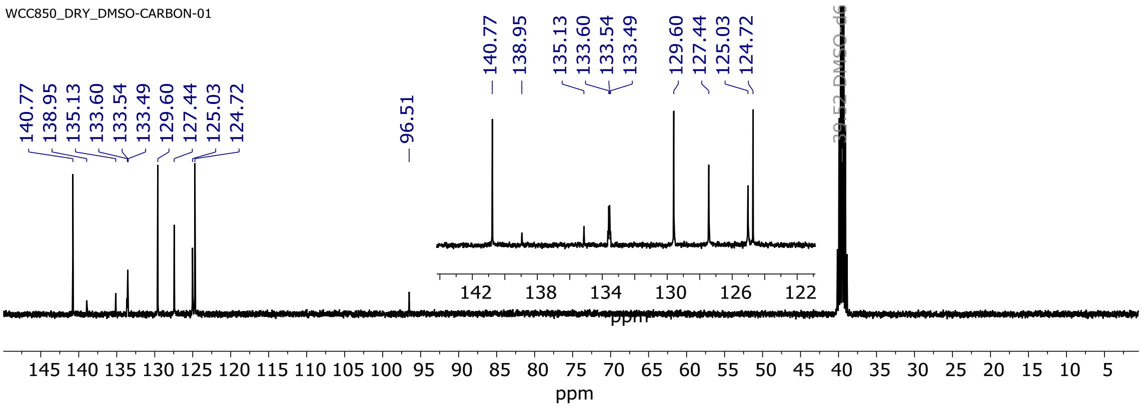

^{19}F NMR (376 MHz, DMSO-d_6)

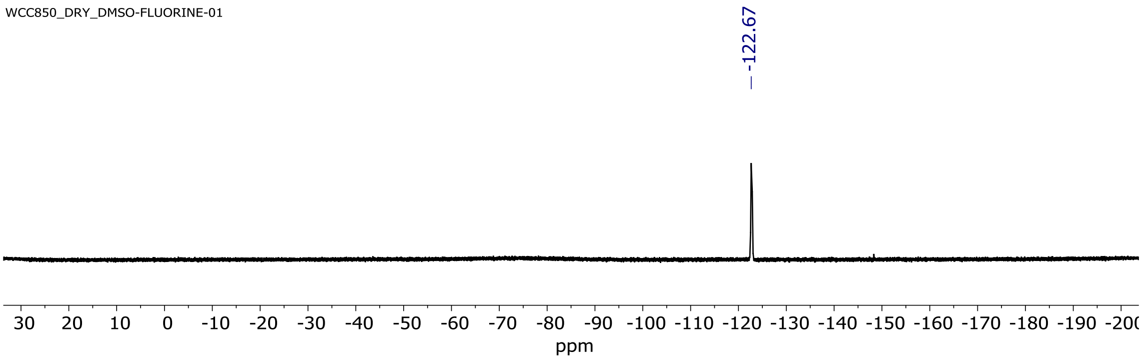

HSQCAD experiment in DMSO-d_6

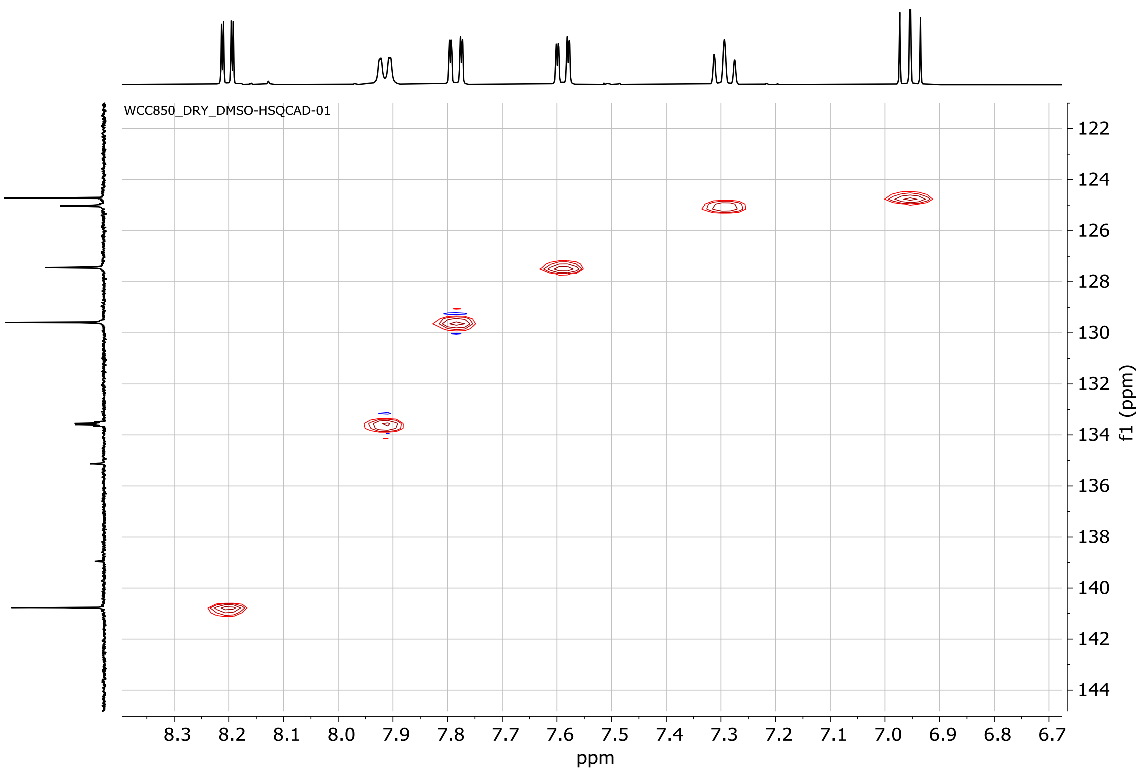

Gradient HMBCAD experiment in DMSO-d_6

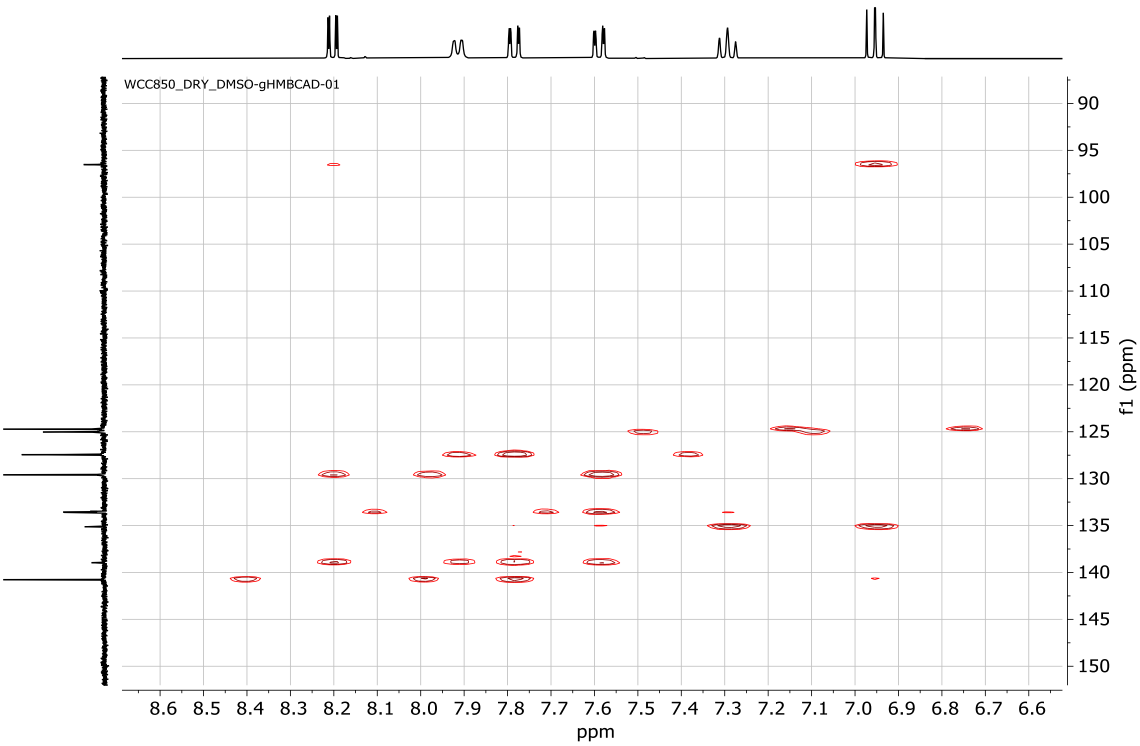

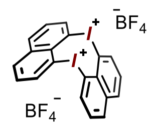

9-$(BF_4)_2$: ^1H NMR (400 MHz, MeOD)

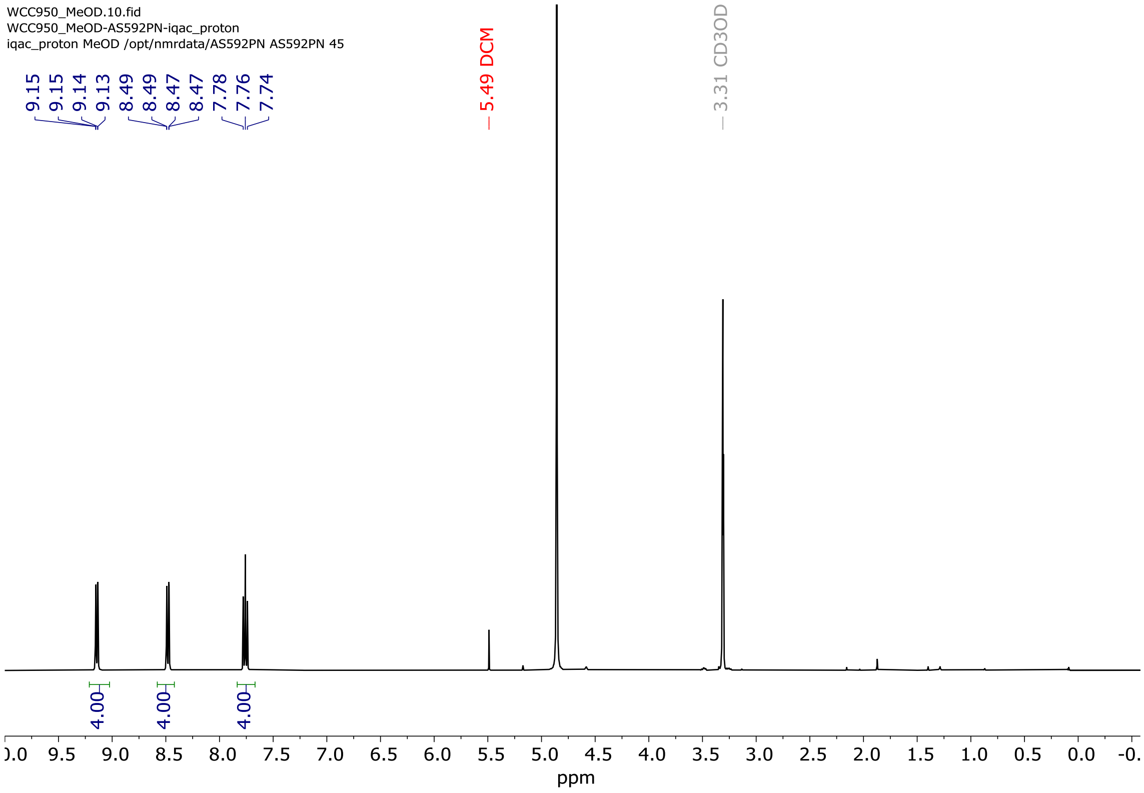

^{13}C NMR (101 MHz, MeOD)

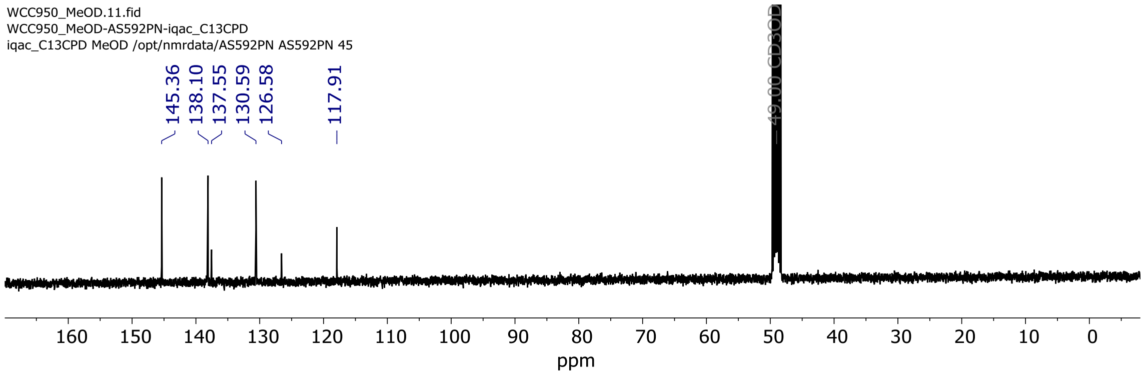

^{19}F NMR (376 MHz, MeOD)

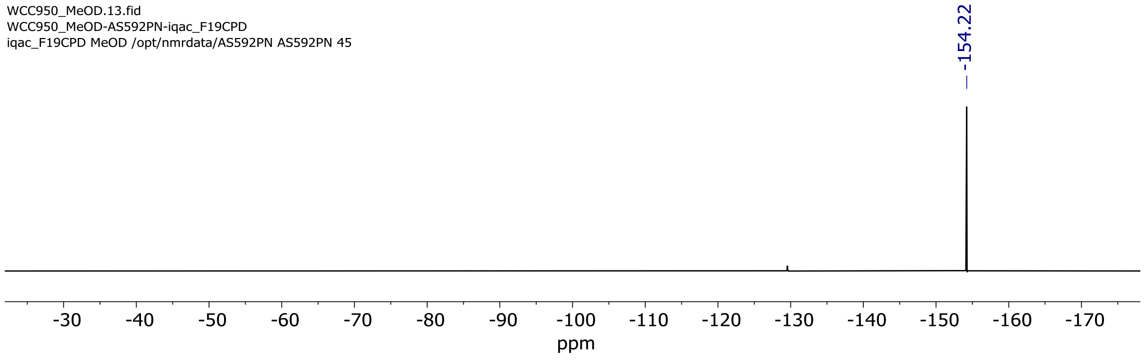

^{11}B NMR (128 MHz, MeOD)

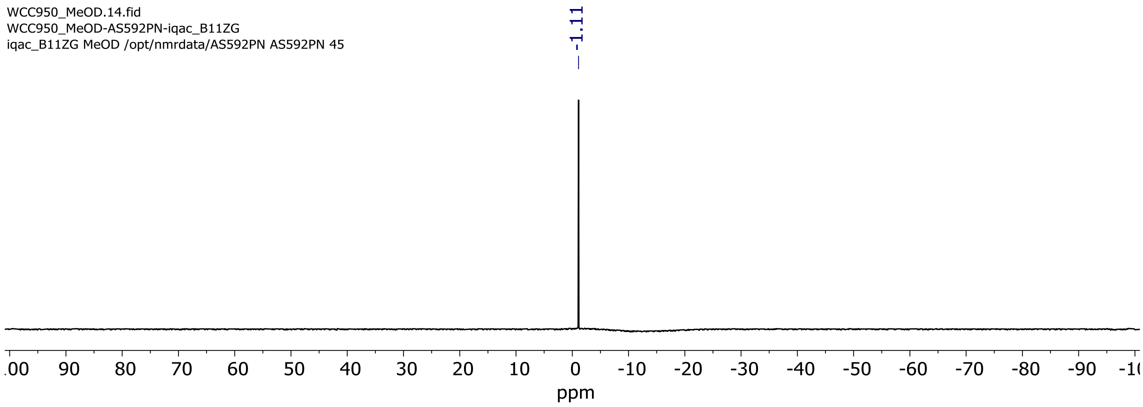

HSQCEDETGPSISP experiment in MeOD

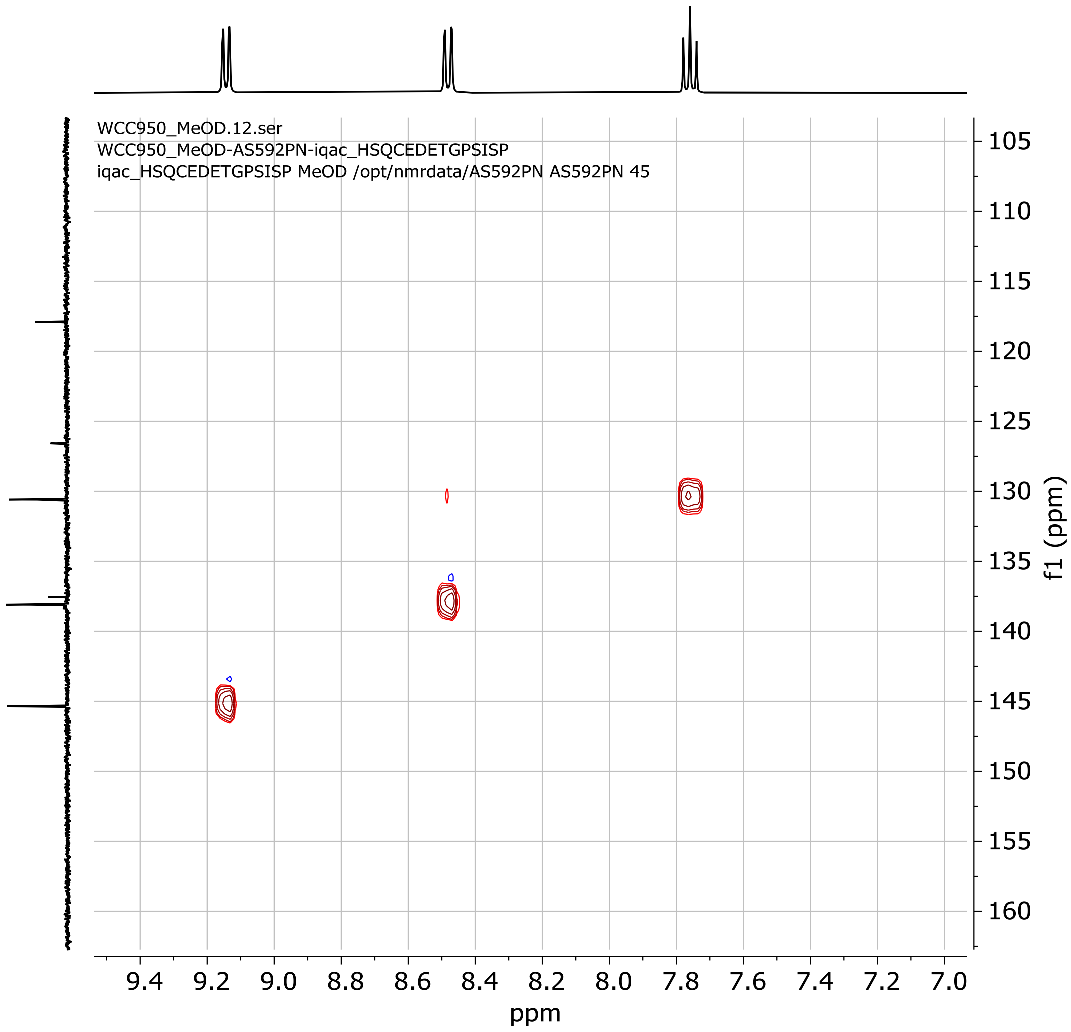

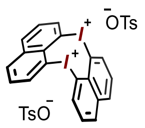

9-(OTs)$_2$: ^{1}H NMR (400 MHz, DMSO-d_6)

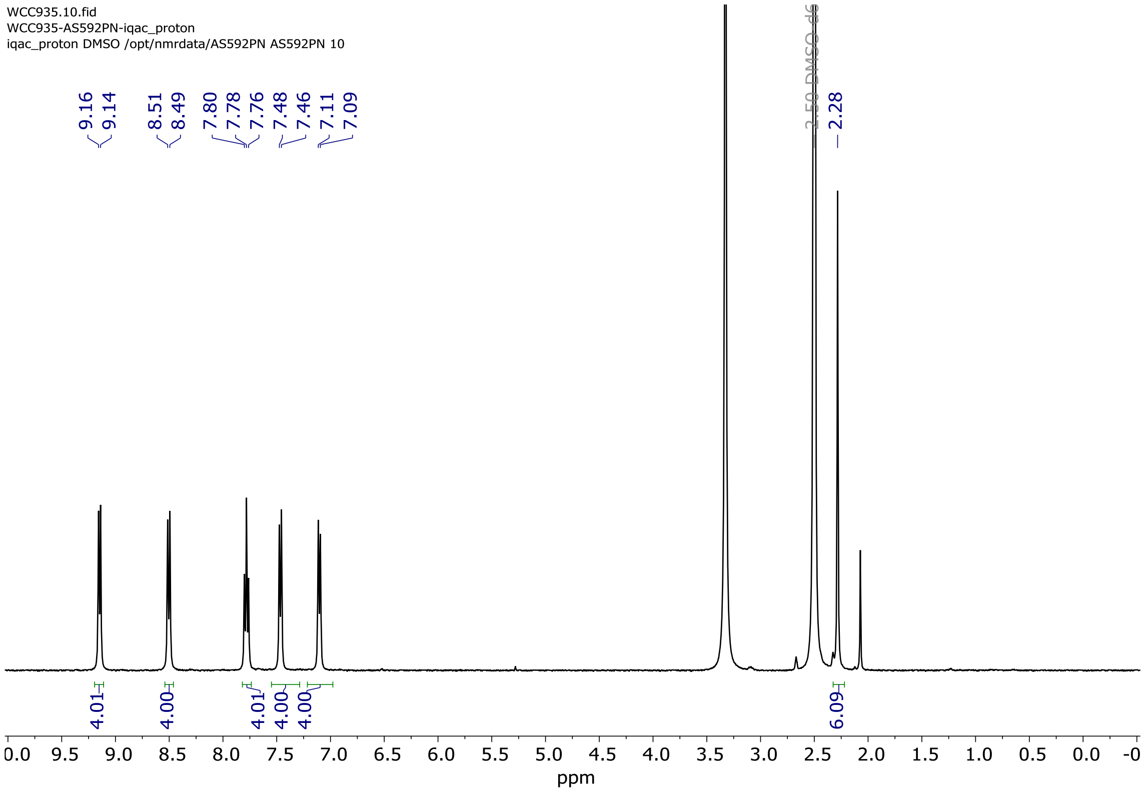

^{13}C NMR (101 MHz, DMSO-d_6)

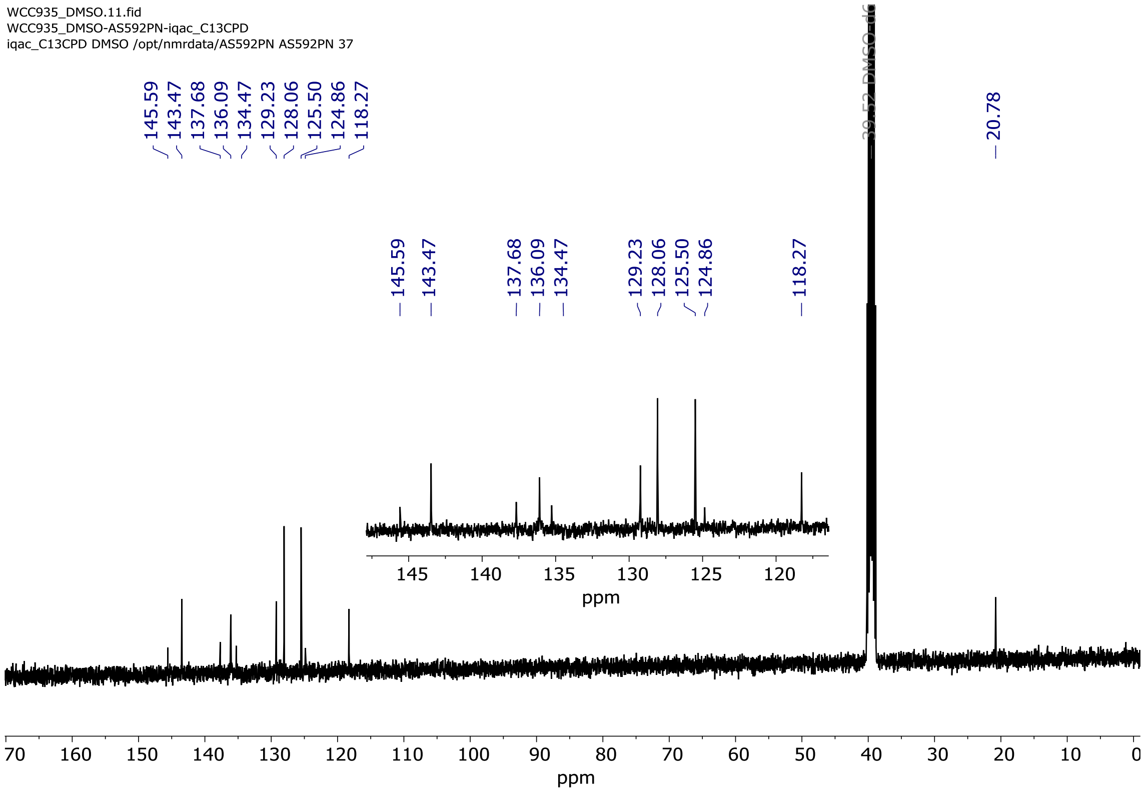

HSQCEDETGPSISP experiment in DMSO-d_6

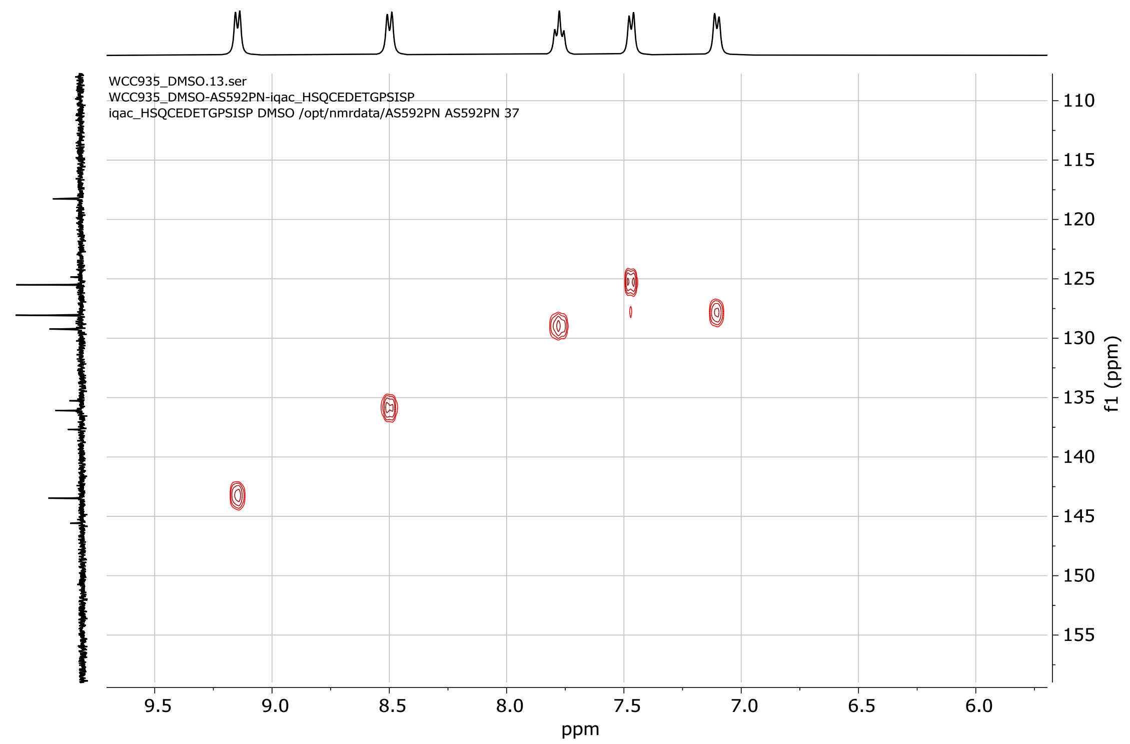

HMBCGP experiment in DMSO-d_6

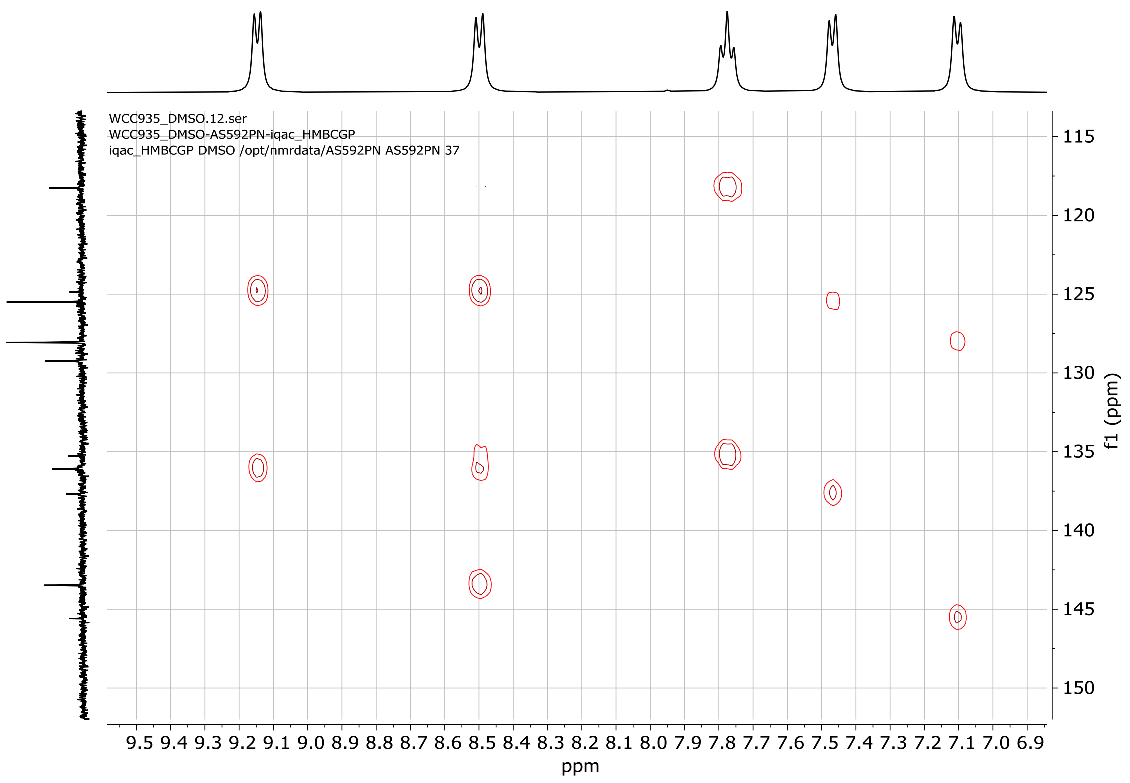

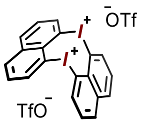

9-(OTf)$_2$: ^{1}H NMR (400 MHz, DMSO-d_6)

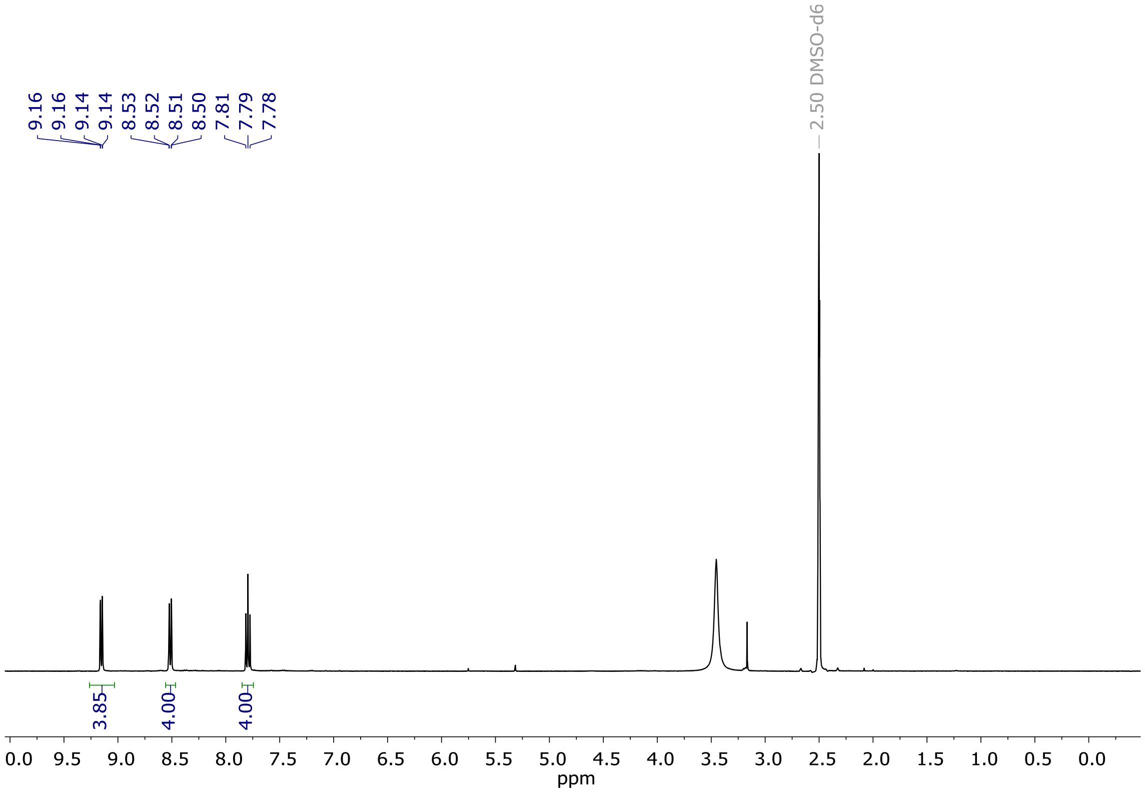

^{13}C NMR (101 MHz, DMSO-d_6)

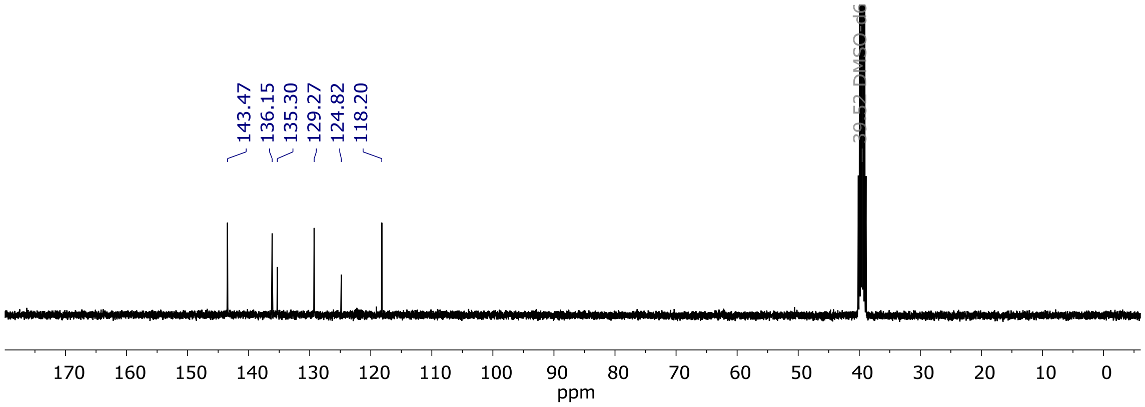

^{19}F NMR (376 MHz, DMSO-d_6)

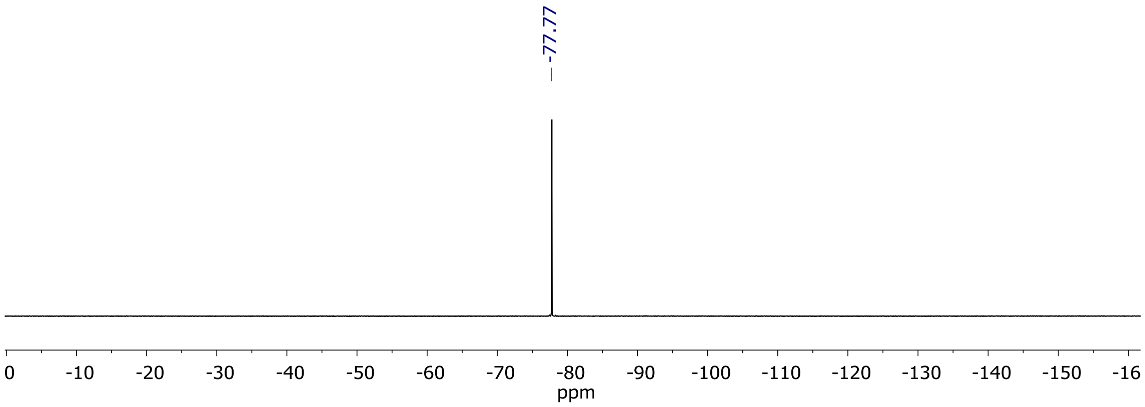

HSQCAD experiment in DMSO-d_6

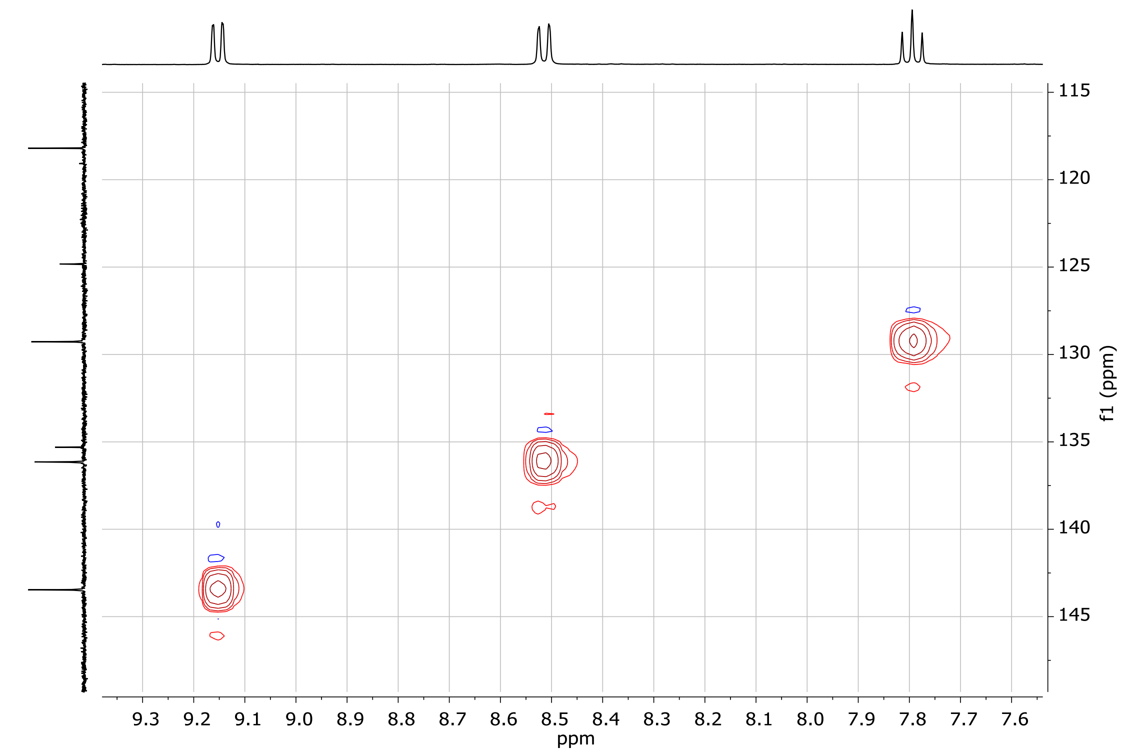

Gradient HMBCAD experiment in DMSO-d_6

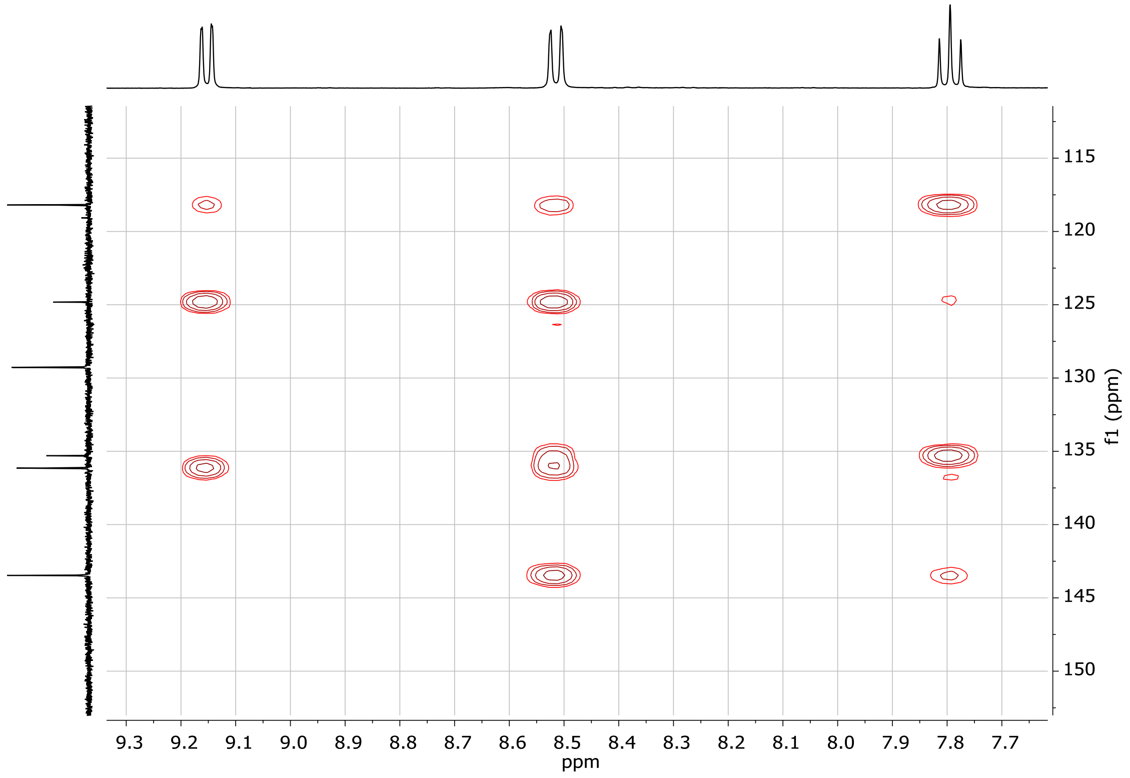

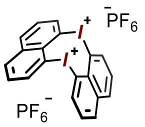

9-$(PF_6)_2$: ^{1}H NMR (400 MHz, MeOD)

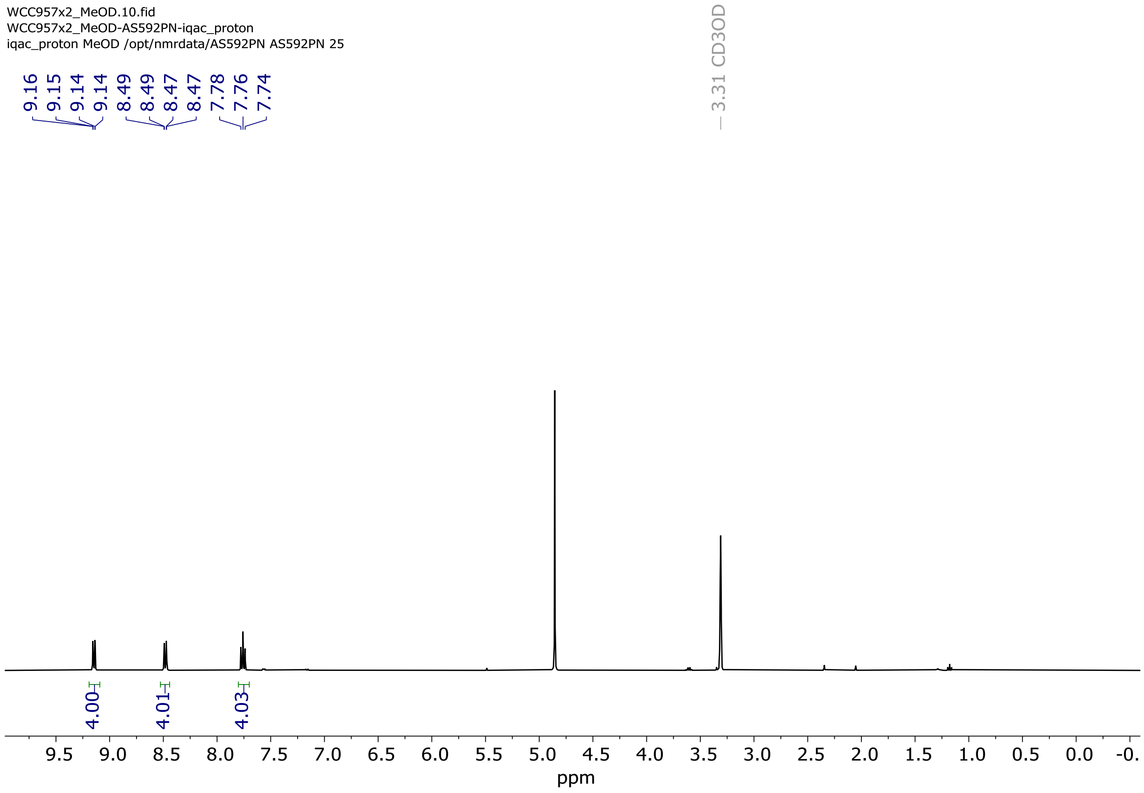

^{13}C NMR (101 MHz, MeOD)

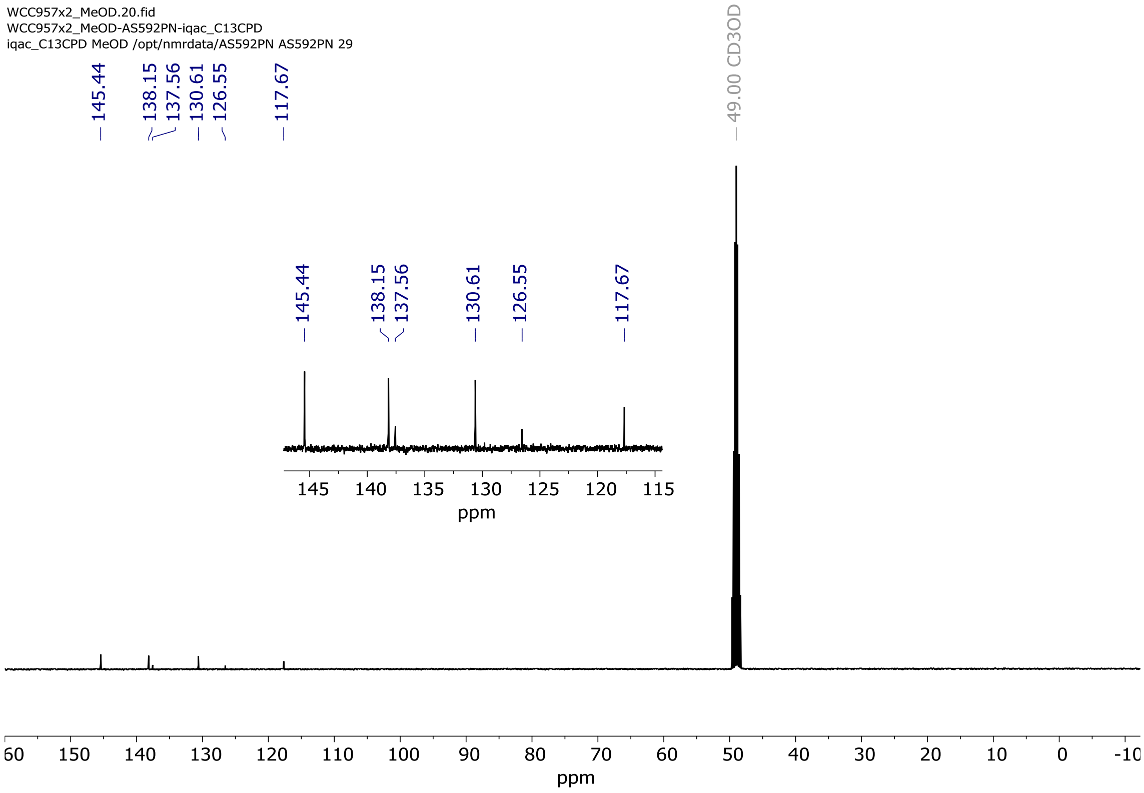

^{19}F NMR (376 MHz, MeOD)

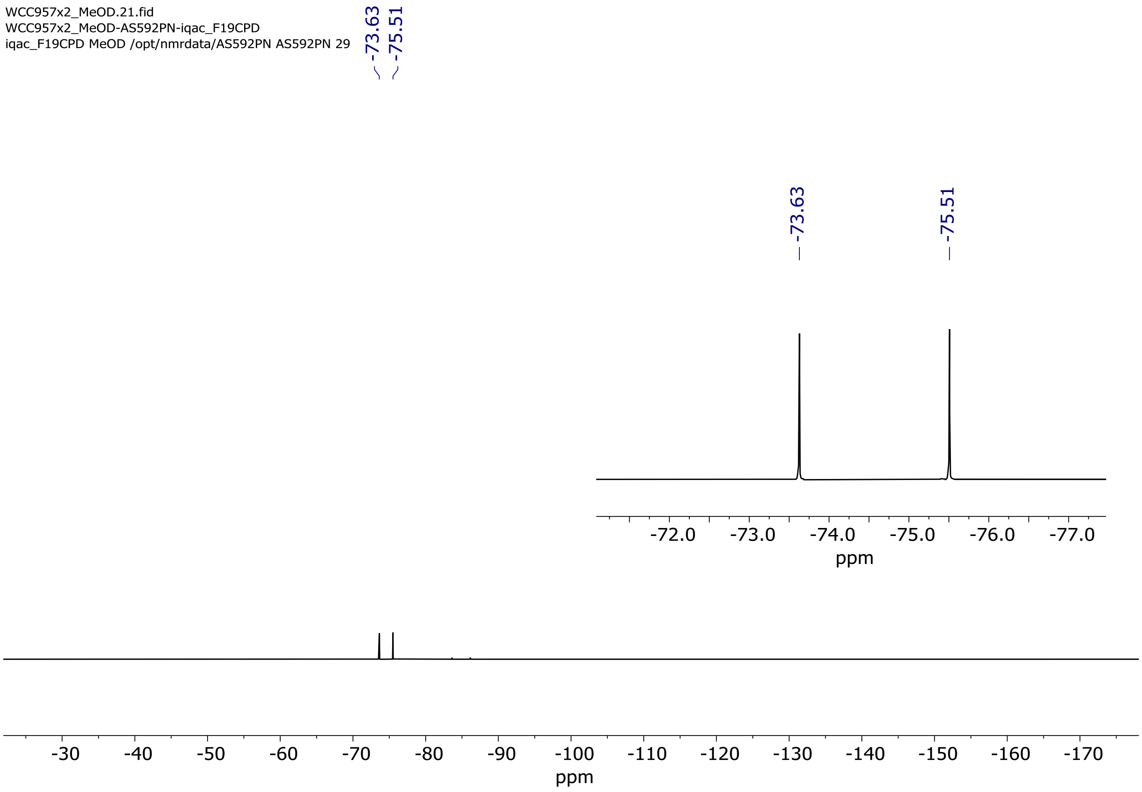

^{31}P NMR (162 MHz, MeOD)

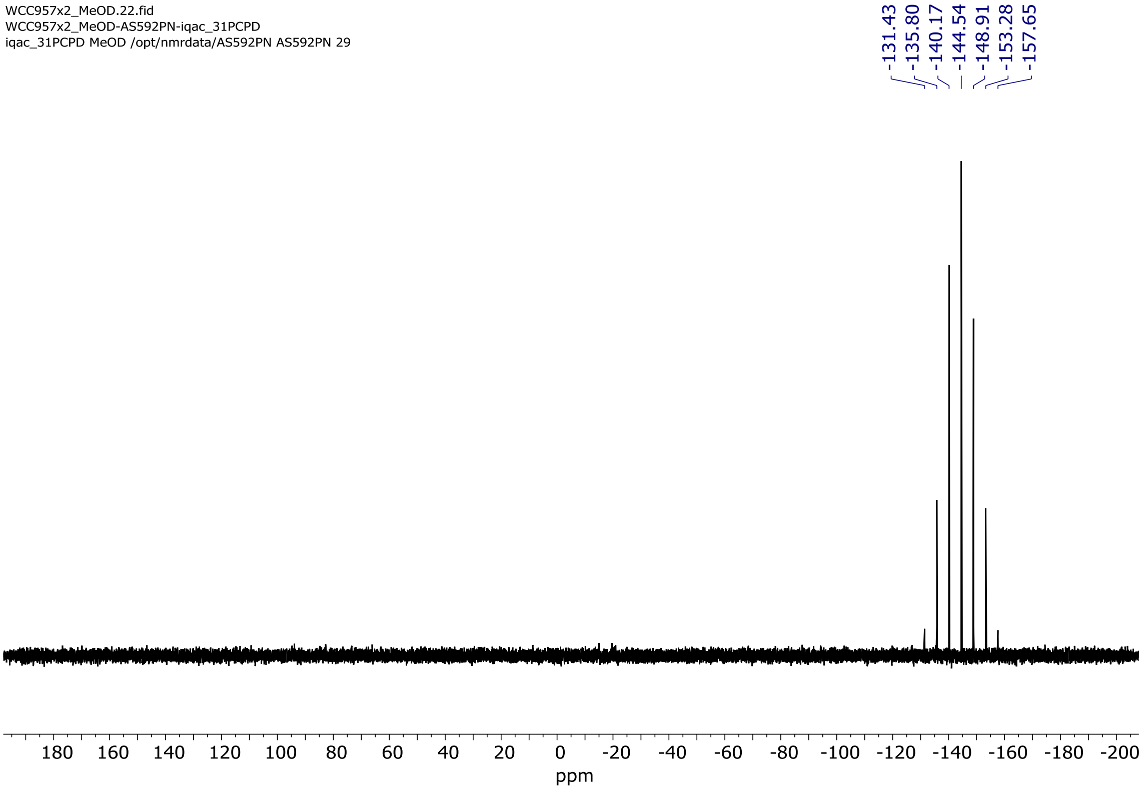

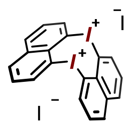

9-(I)$_2$: ^{1}H NMR (400 MHz, DMSO-d$_6$)

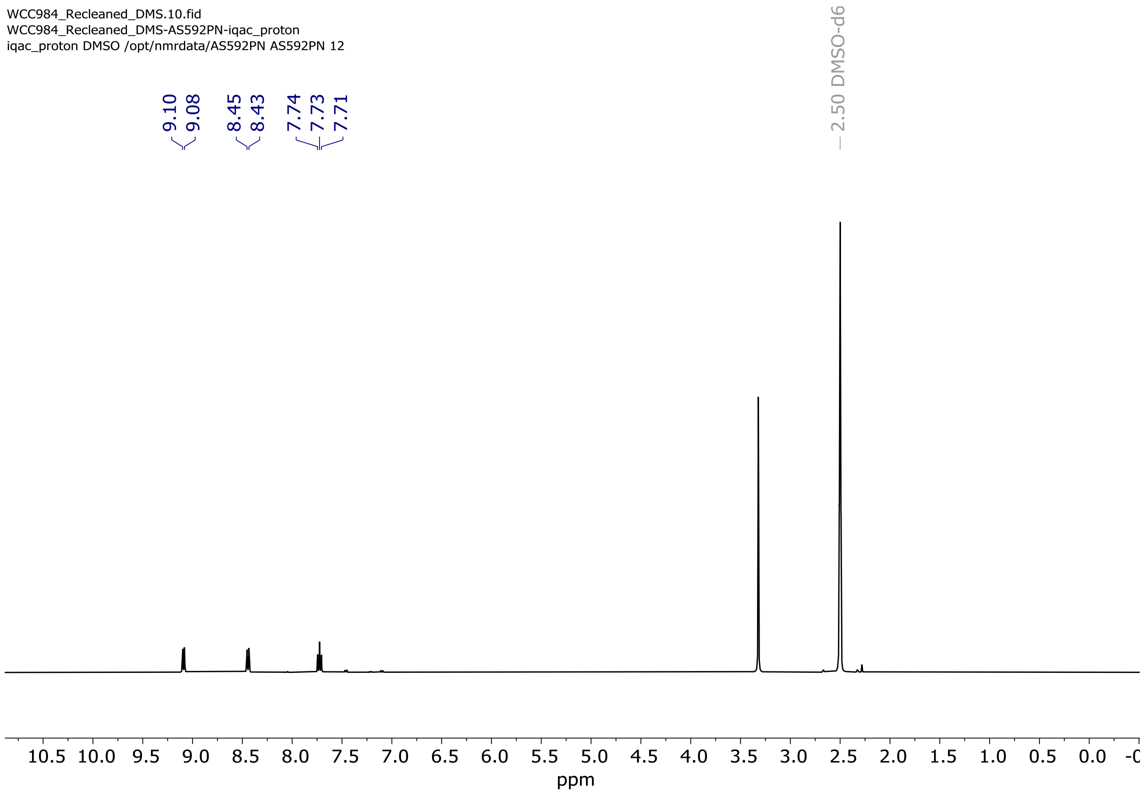

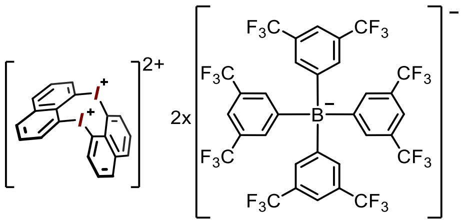

9-(BArf^{24})$_2$: ^{1}H NMR (400 MHz, CD_3OD)

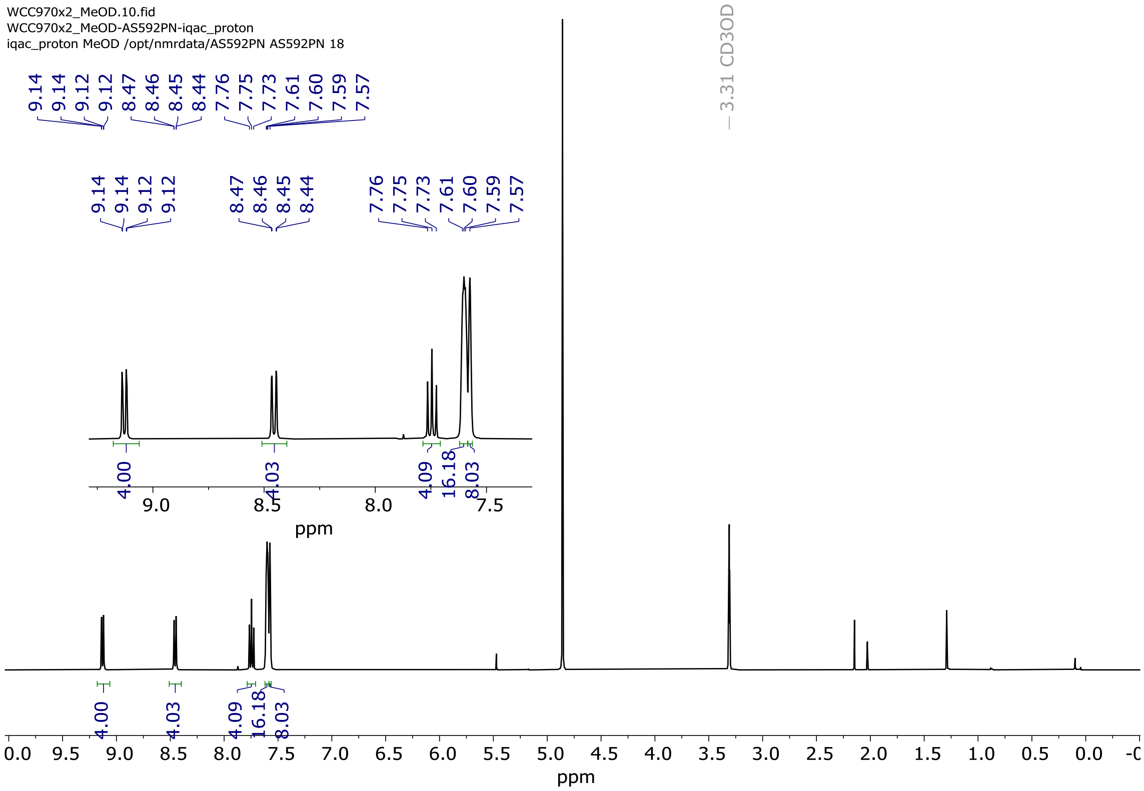

^{13}C NMR (101 MHz, DMSO-d_6)

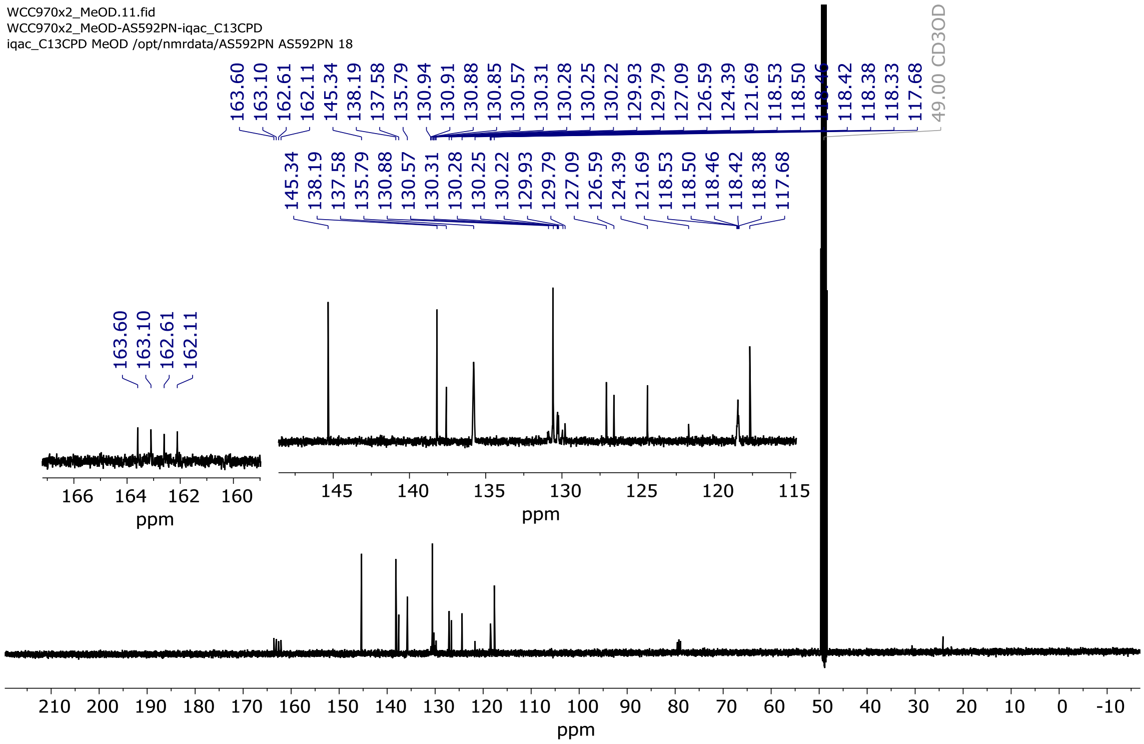

^{19}F NMR (376 MHz, DMSO-d_6)

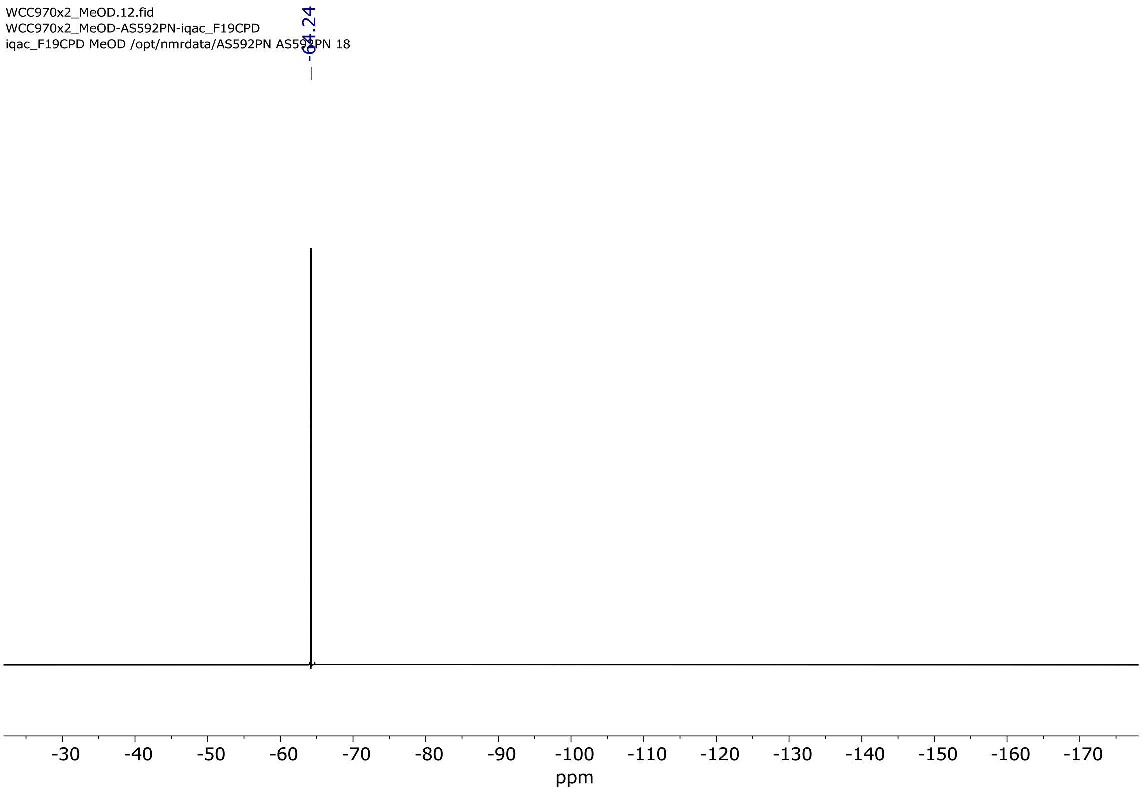

^{11}B NMR (128 MHz, MeOD)

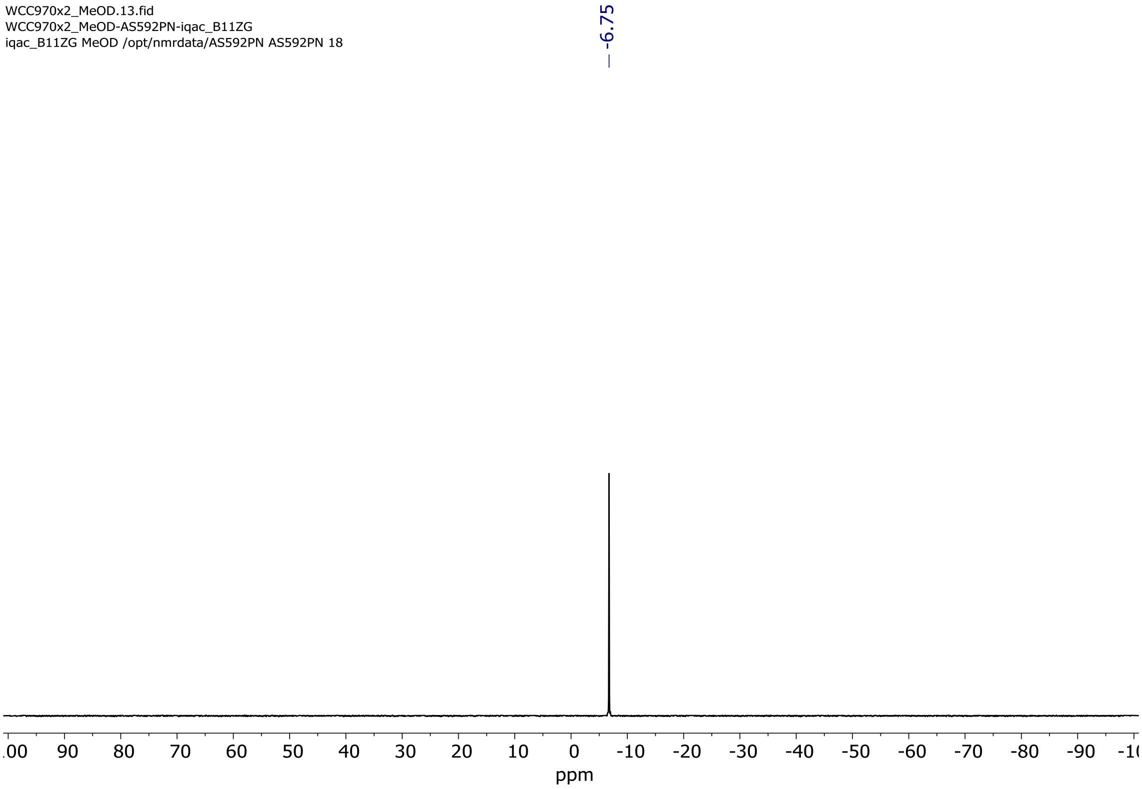

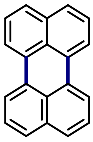

perylene: ^{1}H NMR (400 MHz, $CDCl_3$)

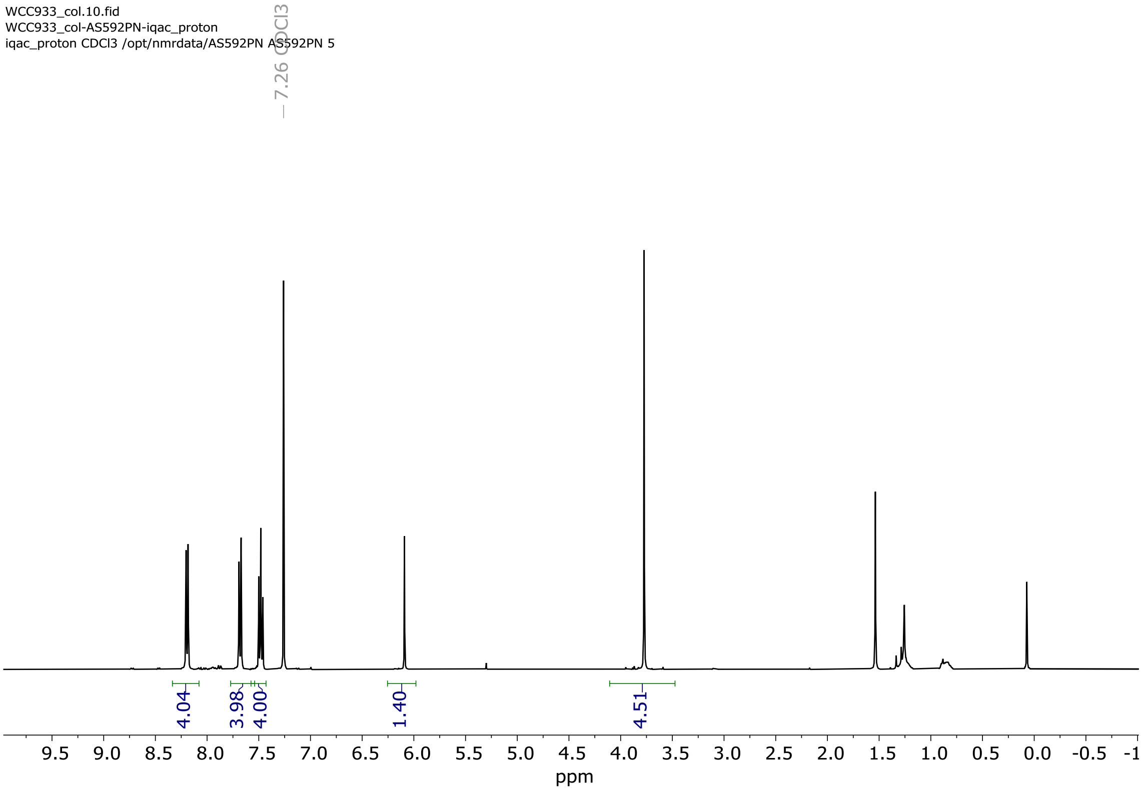

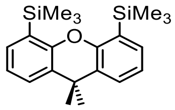

(**11**): ^{1}H NMR (400 MHz, $CDCl_3$)

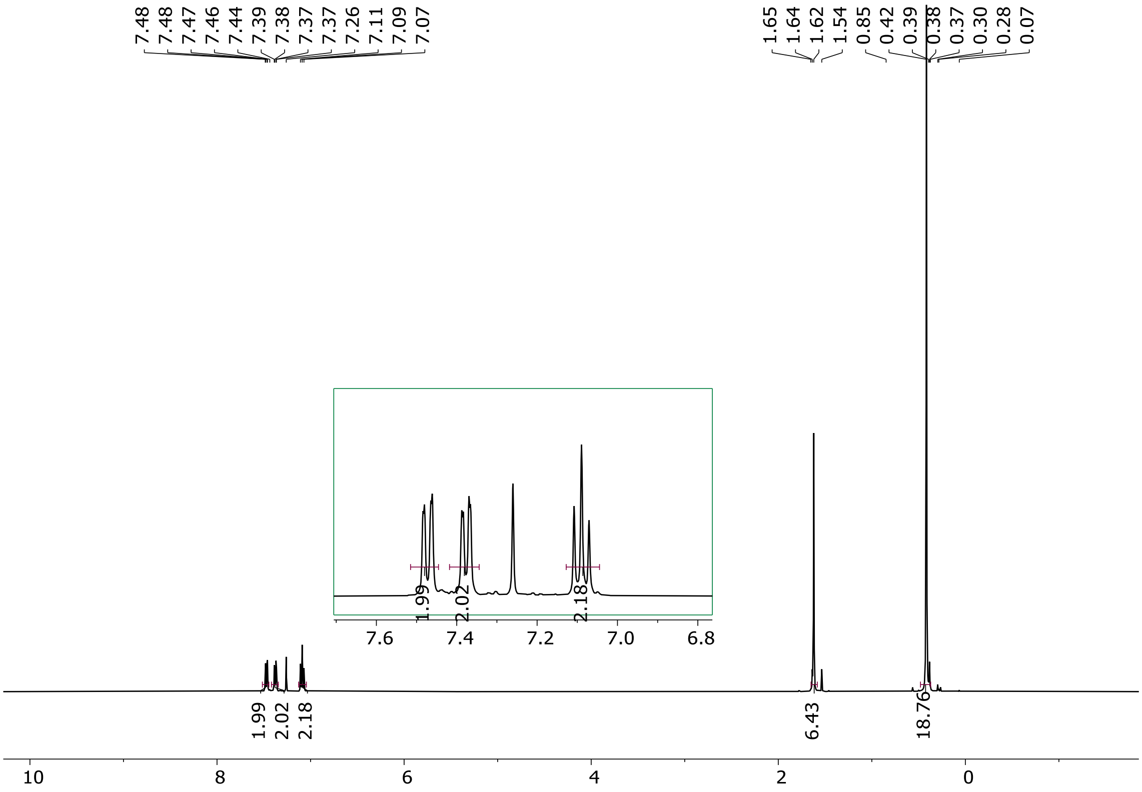

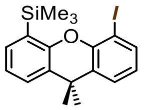

(**12**): ^{1}H NMR (400 MHz, $CDCl_3$)

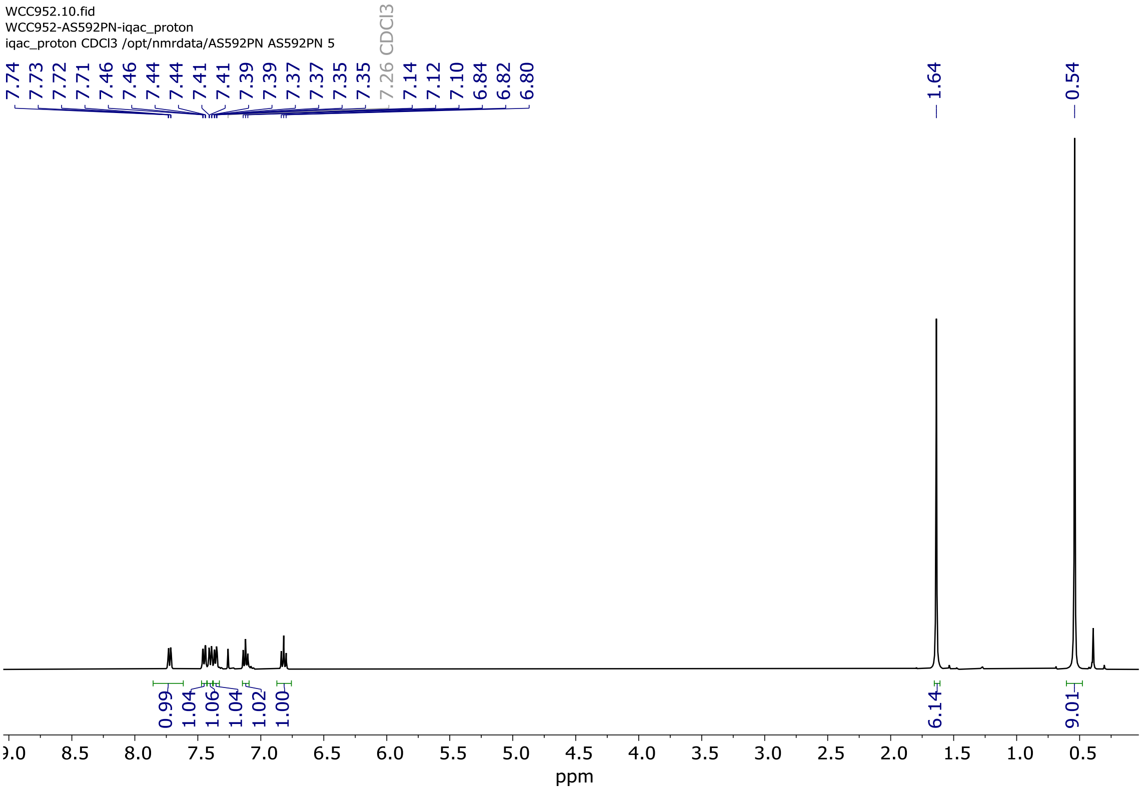

^{13}C NMR (101 MHz, $CDCl_3$)

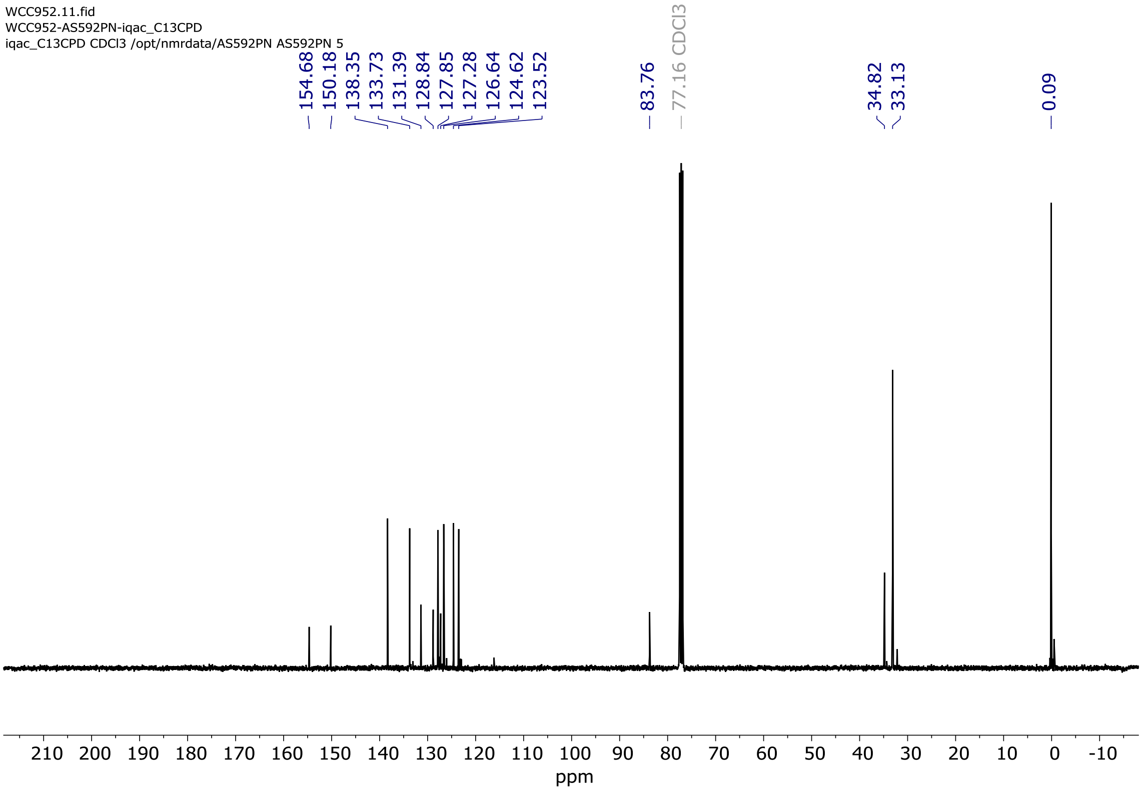

^{1}H-^{13}C HSQCEDETGPSISP experiment in $CDCl_3$

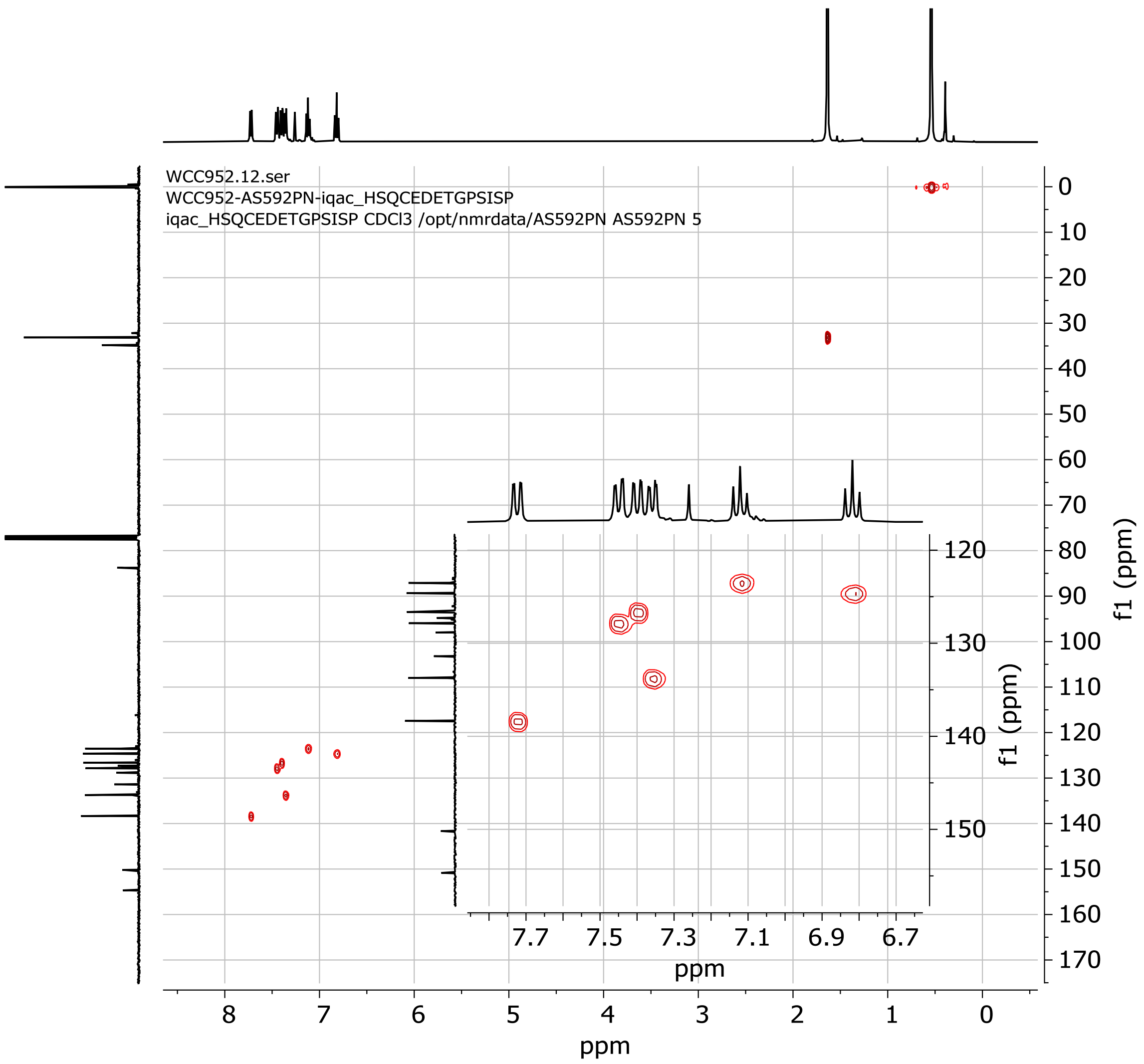

WCC952.12.ser
WCC952-AS592PN-iqac_HSQCEDETGPSISP
iqac_HSQCEDETGPSISP CDCl3 /opt/nmrdata/AS592PN AS592PN 5
f1 (ppm)
ppm

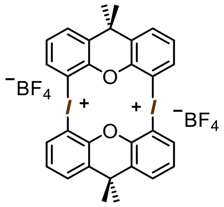

14-$(BF_4)_2$: ^{1}H NMR (400 MHz, CD_3OD)

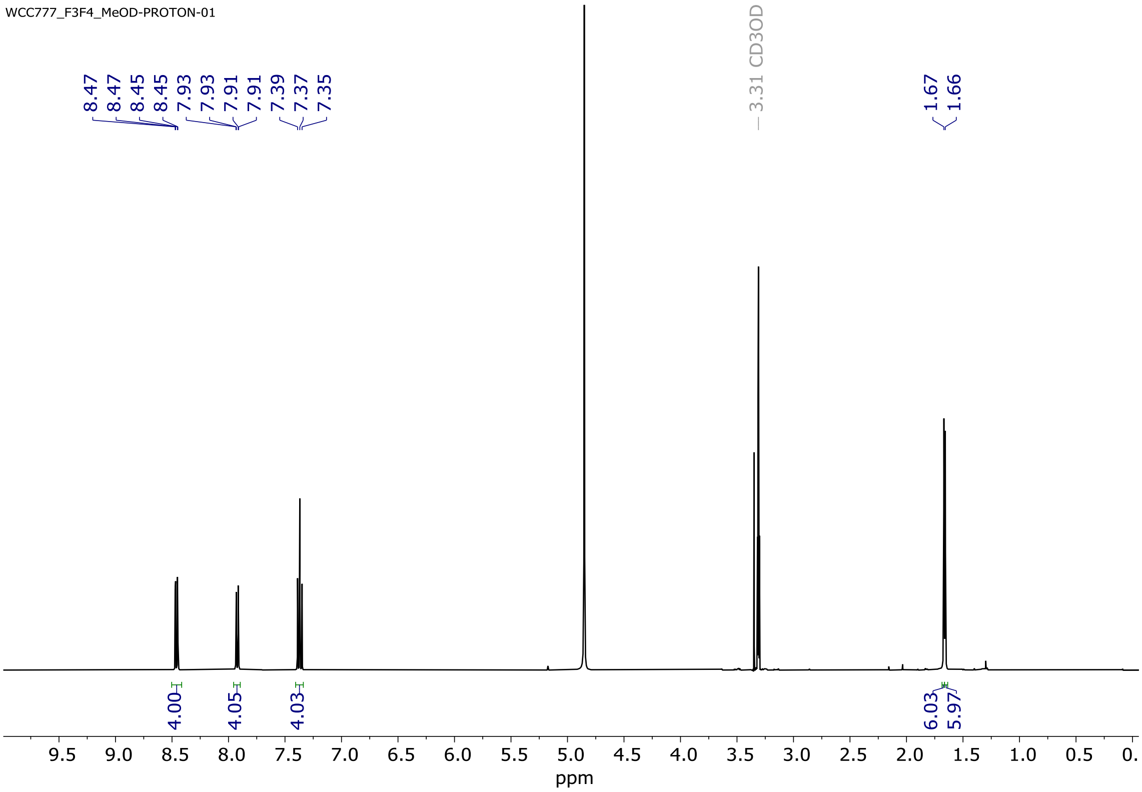

^{13}C NMR (101 MHz, CD_3OD)

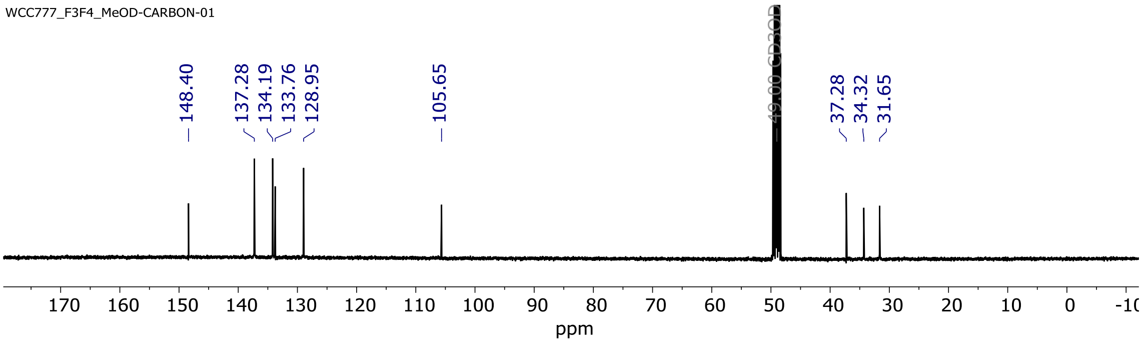

^{19}F NMR (376 MHz, CD_3OD)

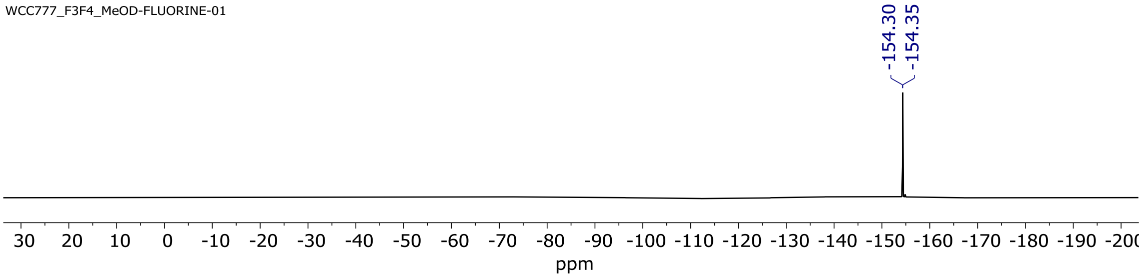

^{1}H-^{13}C HSQCAD experiment in CD_3OD

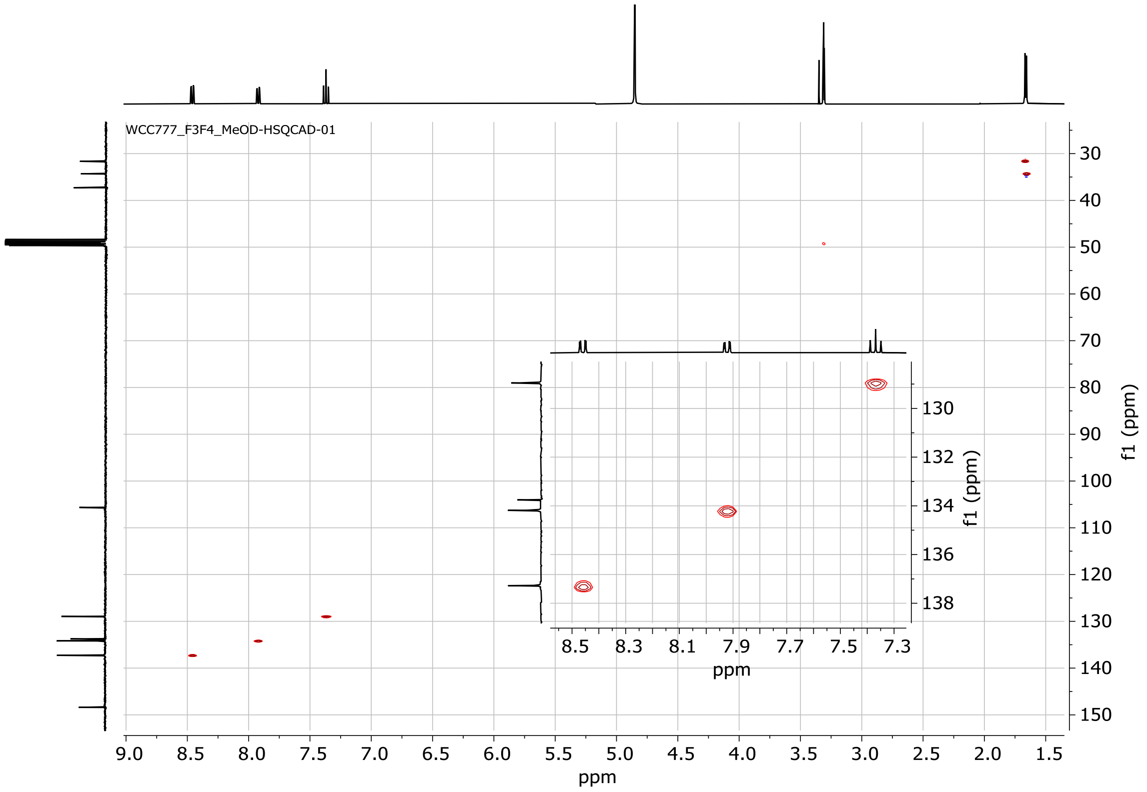

^{1}H-^{13}C gHMBCAD experiment in CD_3OD

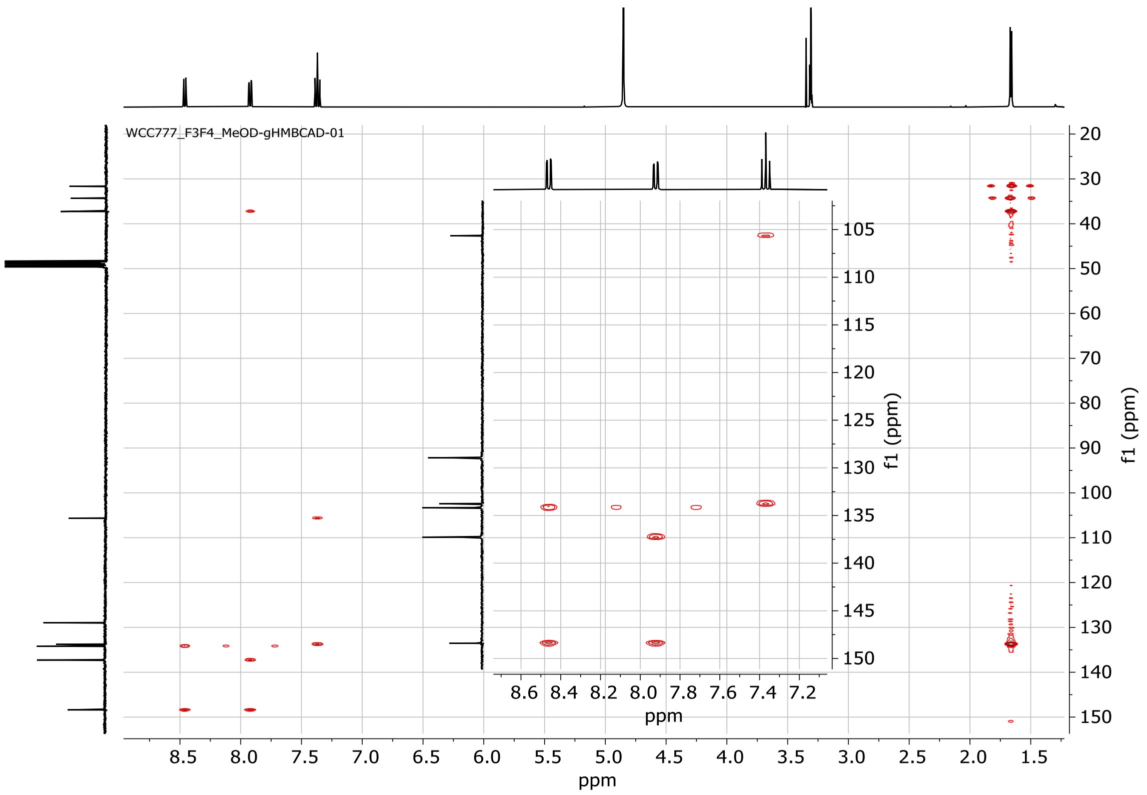

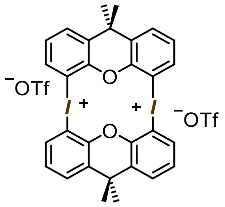

14-(OTf)$_2$: ^{1}H NMR (400 MHz, CD_3OD)

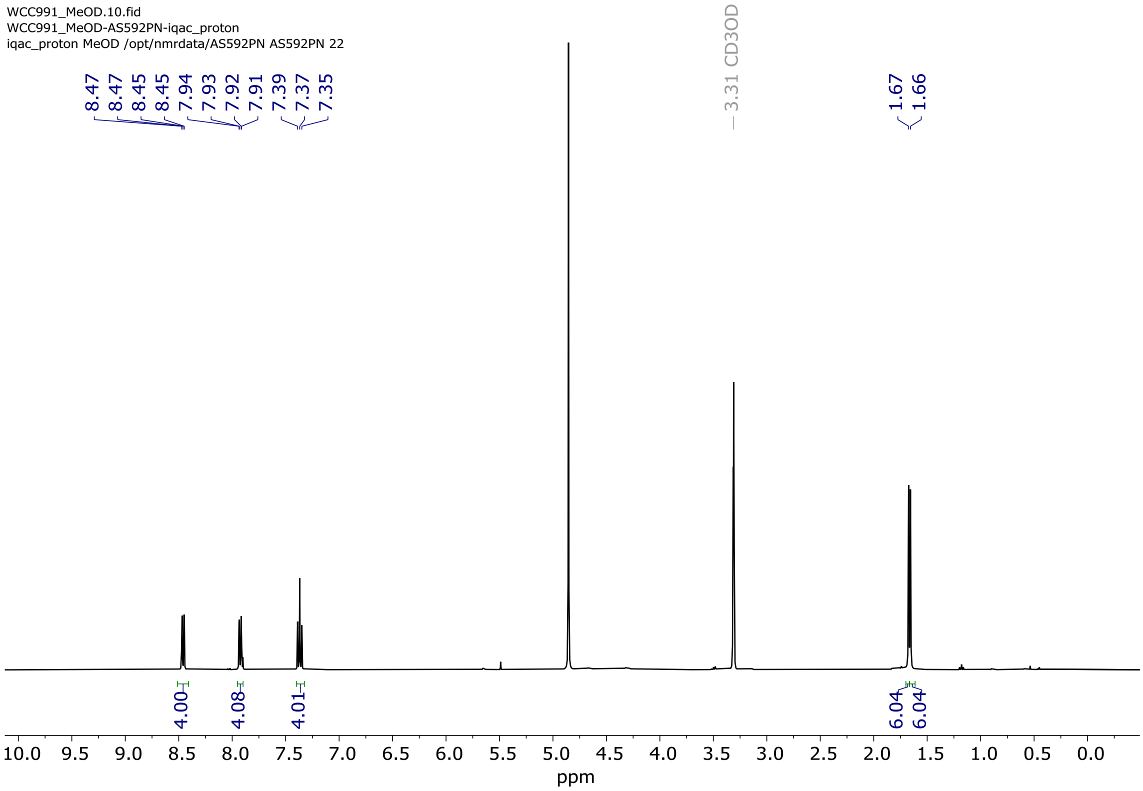

^{13}C NMR (101 MHz, CD_3OD)

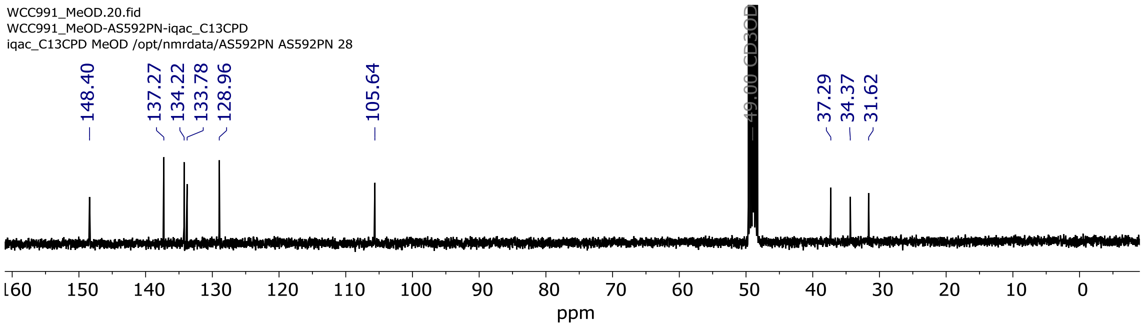

^{19}F NMR (376 MHz, CD_3OD)

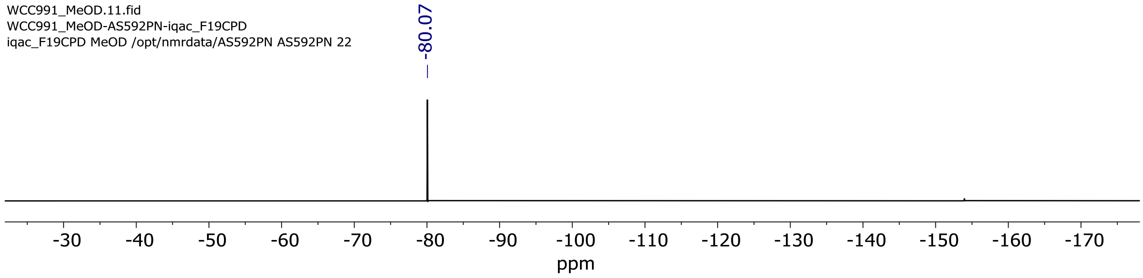

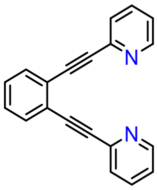

(**L2**): ^{1}H NMR (400 MHz, CD_2Cl_2)

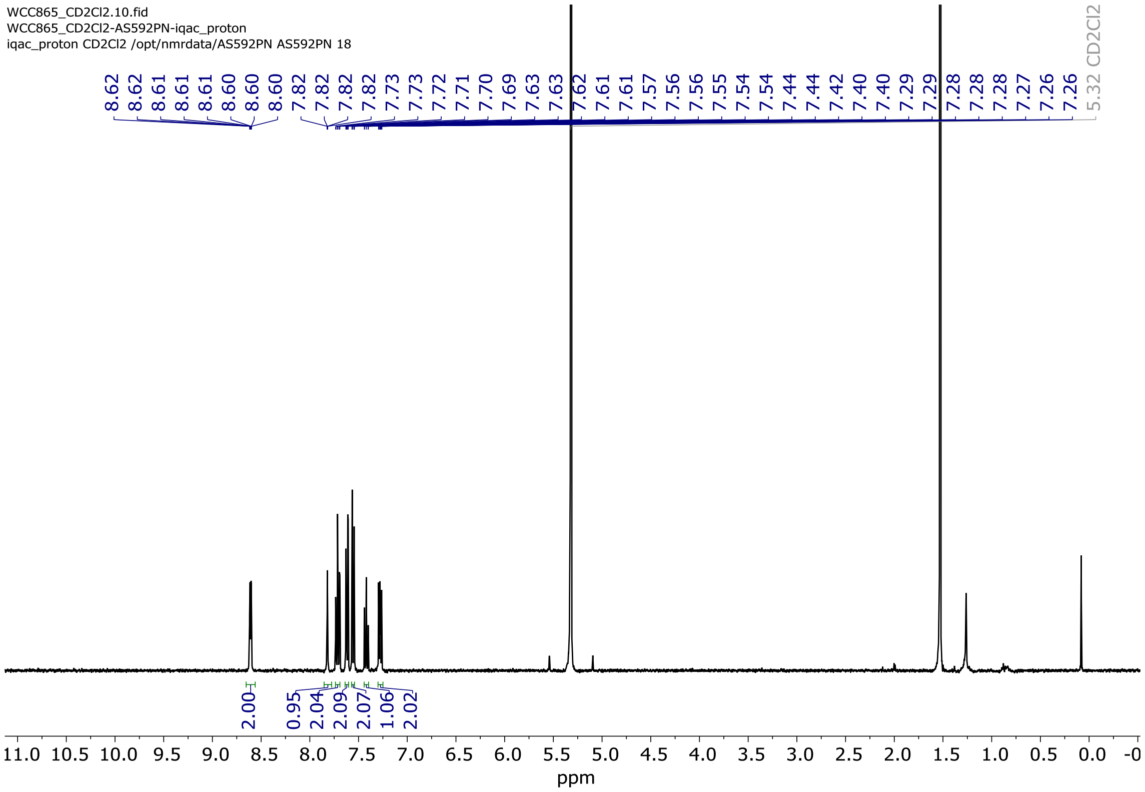

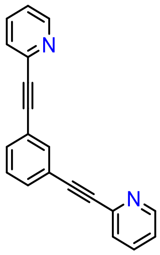

(**L1**): ^{1}H NMR (400 MHz, $CDCl_3$)

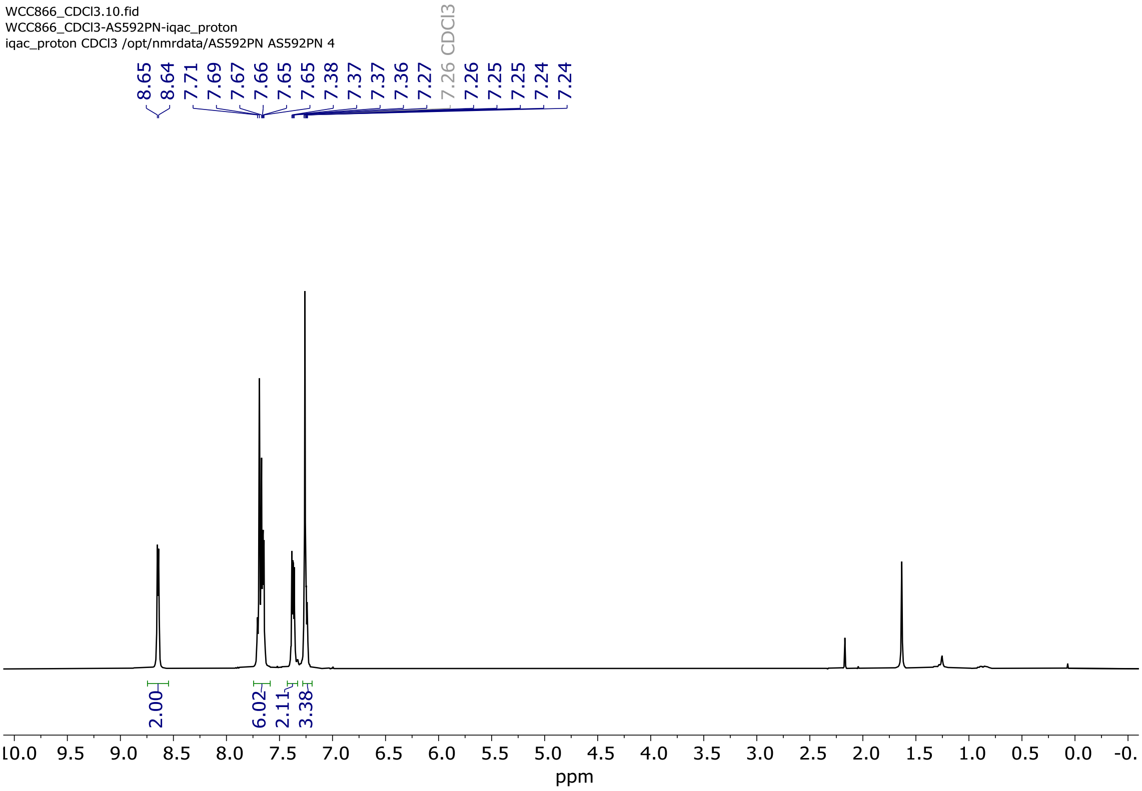

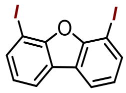

(**x**): ^{1}H NMR (400 MHz, $CDCl_3$)

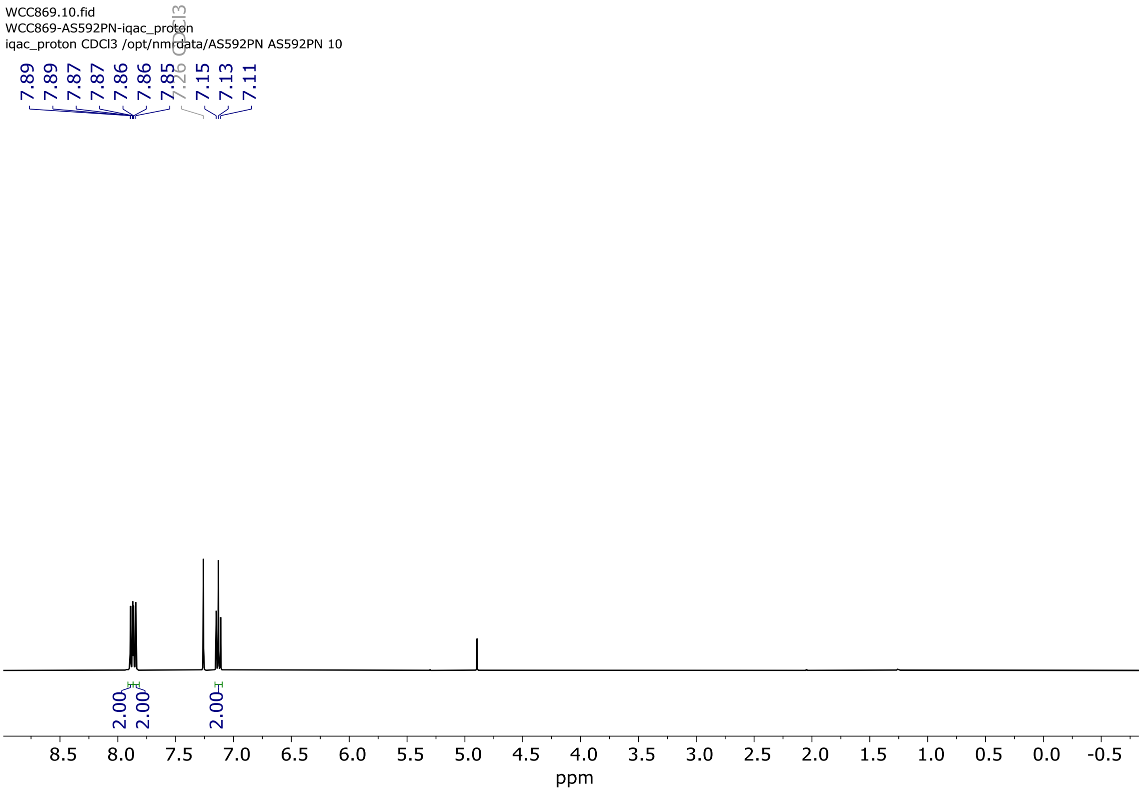

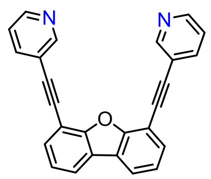

(**L3**): ^{1}H NMR (400 MHz, Acetone)

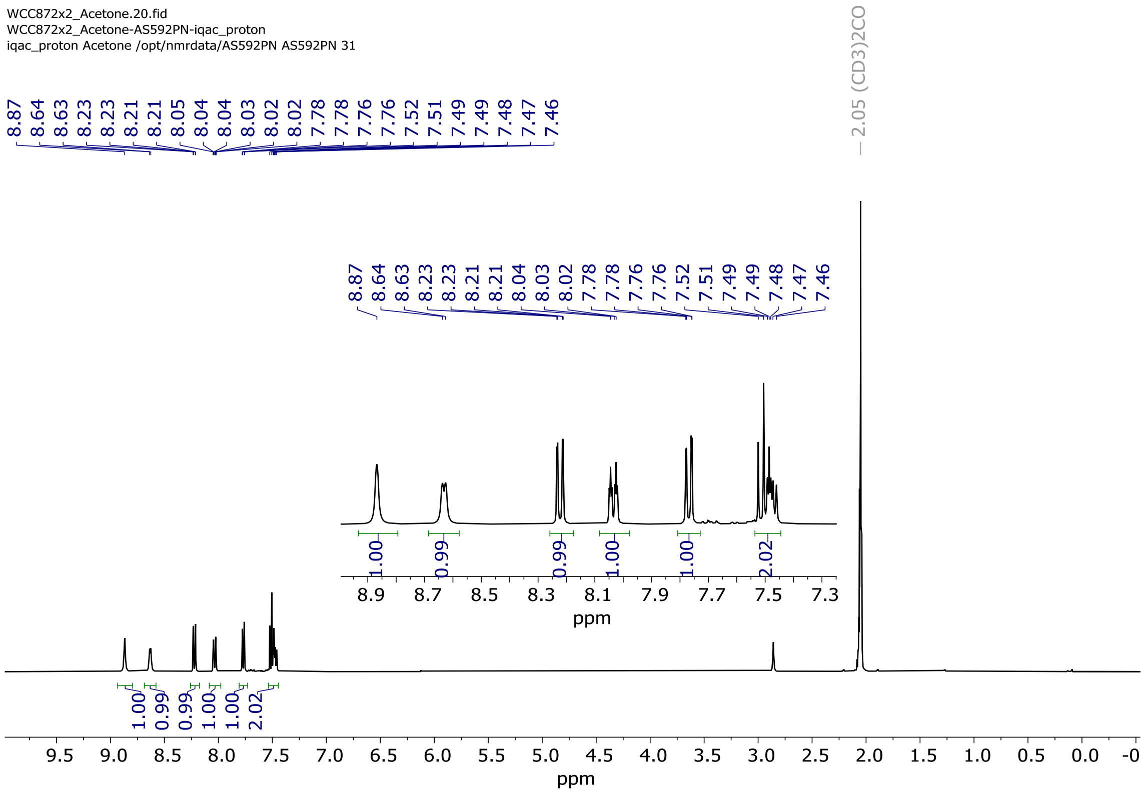

^{13}C NMR (101 MHz, Acetone)

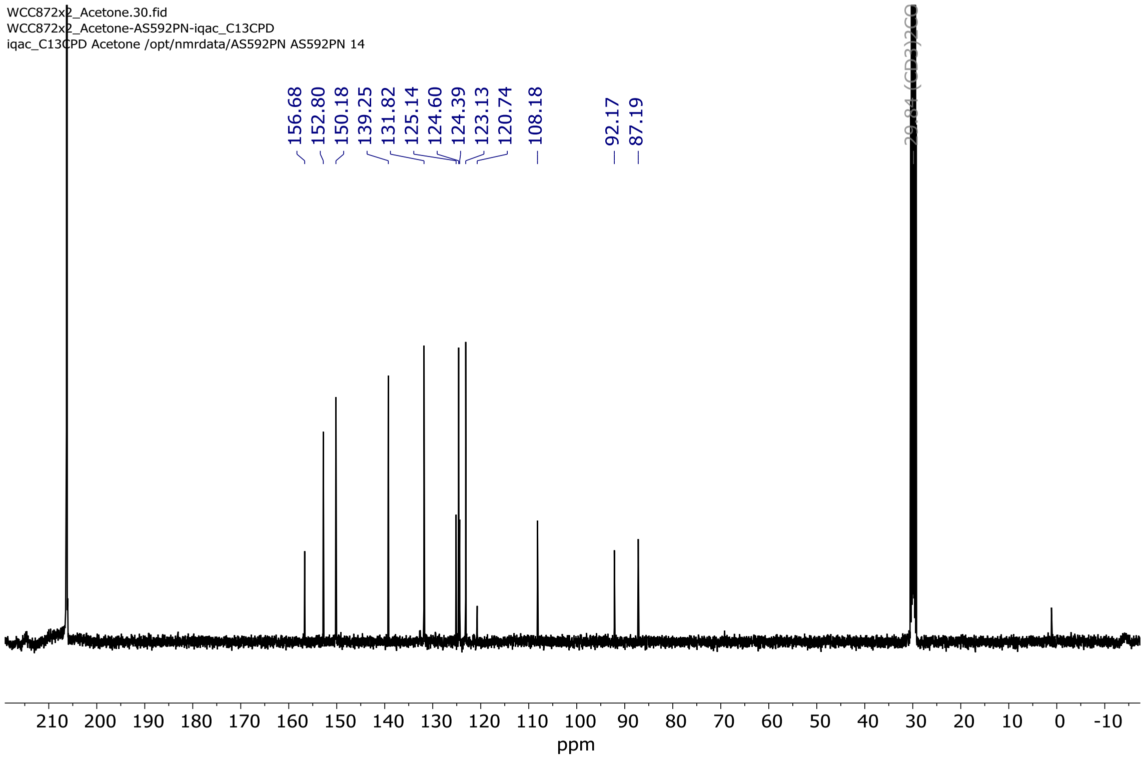

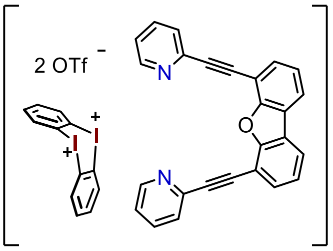

1·**L3**-(OTf)$_2$

(**1**·L3-(OTf)$_2$): ^{1}H NMR (400 MHz, DMSO)

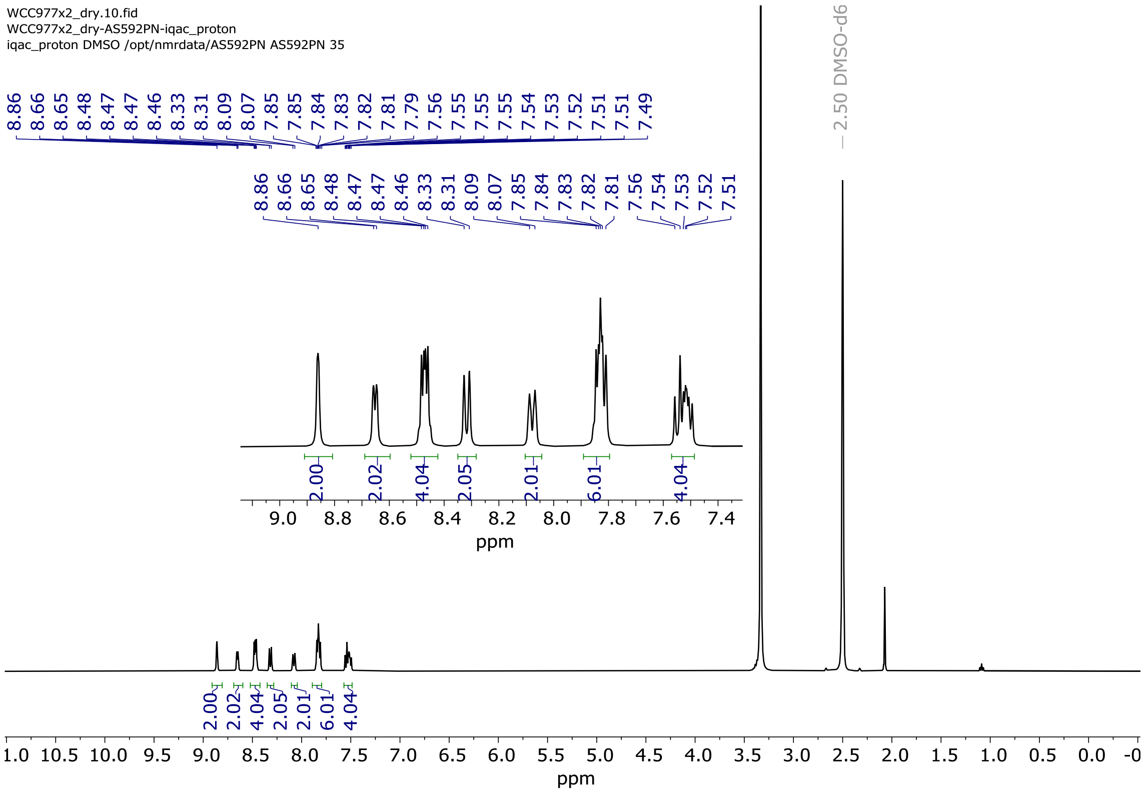

^{19}F NMR (376 MHz, DMSO-d_6)

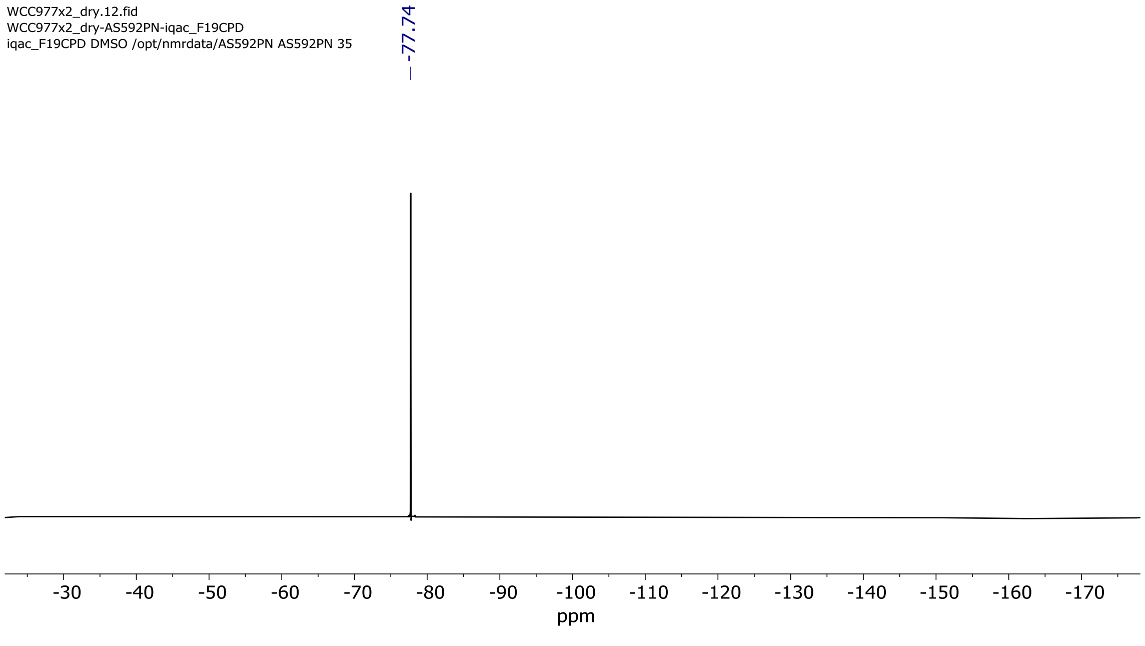

^{13}C NMR (101 MHz, DMSO)

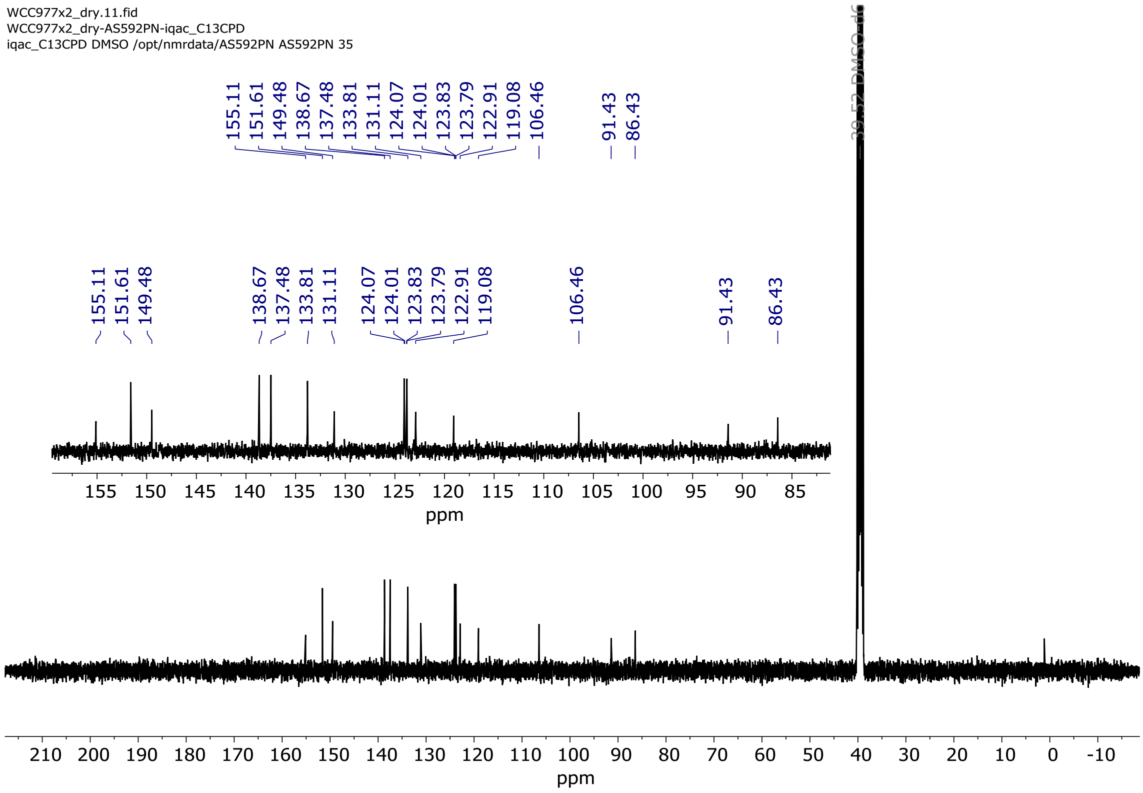

6. X-Ray diffraction structural determination details.

In all cases, a prismatic crystal was selected and used for the X-ray crystallographic analysis. The X-ray intensity data were measured on a D8 Venture system equipped with a multilayer monochromator and a Mo microfocus (λ = 0.71073 Å). The frames were integrated with the Bruker SAINT software package using a narrow-frame algorithm. Data were corrected for absorption effects using the multi-scan method (SADABS), and the structure was solved and refined using the Bruker SHELXTL software package.

Table S1. Crystal data and structure refinement for 3ANZB133_0m_a.

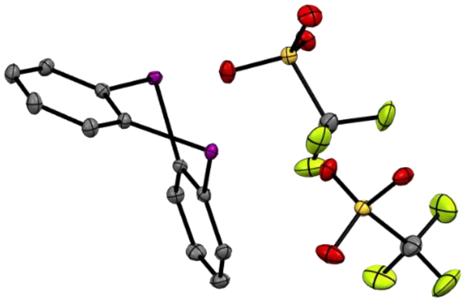

Identification code	3ANZB133_0m_a	
Empirical formula	C14 H8 F6 I2 O6 S2	
Formula weight	704.12	
Temperature	100(2) K	
Wavelength	0.71073 Å	
Crystal system	Triclinic	
Space group	P -1	
Unit cell dimensions	a = 9.4610(4) Å	α = 78.005(2)°.
	b = 10.2644(5) Å	β = 70.144(2)°.
	c = 11.6909(5) Å	γ = 70.780(2)°.
Volume	1002.40(8) $Å^3$	
Z	2	
Density (calculated)	2.333 Mg/m^3	
Absorption coefficient	3.431 mm^{-1}	
F(000)	664	
Crystal size	0.080 x 0.060 x 0.040 mm^3	
Theta range for data collection	2.662 to 30.573°.	
Index ranges	-13<=h<=13, -14<=k<=14, -16<=l<=16	
Reflections collected	28260	
Independent reflections	6034 [R(int) = 0.0205]	
Completeness to theta = 25.242°	99.2 %	
Absorption correction	Semi-empirical from equivalents	
Max. and min. transmission	0.7461 and 0.6520	
Refinement method	Full-matrix least-squares on F^2	
Data / restraints / parameters	6034 / 0 / 271	
Goodness-of-fit on F^2	1.081	
Final R indices [I>2sigma(I)]	R1 = 0.0143, wR2 = 0.0308	
R indices (all data)	R1 = 0.0164, wR2 = 0.0322	
Largest diff. peak and hole	0.468 and -0.447 e.$Å^{-3}$	

Table S2. Crystal data and structure refinement for 3ANZB115_0m_a.

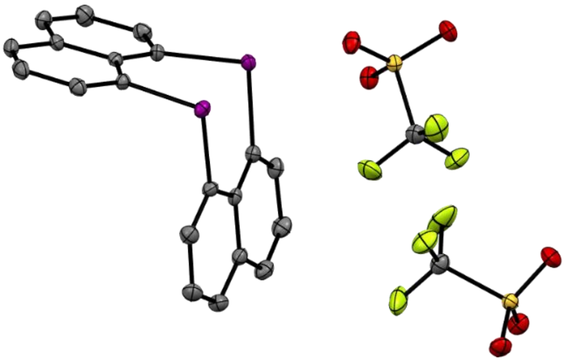

Identification code	3ANZB115_0m_a	
Empirical formula	C22 H12 F6 I2 O6 S2	
Formula weight	804.24	
Temperature	100(2) K	
Wavelength	0.71073 Å	
Crystal system	Triclinic	
Space group	P -1	
Unit cell dimensions	a = 9.9779(7) Å	α = 77.909(2)°.
	b = 11.3034(8) Å	β = 71.089(2)°.
	c = 11.9057(8) Å	γ = 74.996(2)°.
Volume	1215.35(15) $Å^3$	
Z	2	
Density (calculated)	2.198 Mg/m^3	
Absorption coefficient	2.845 mm^{-1}	
F(000)	768	
Crystal size	0.090 x 0.060 x 0.040 mm^3	
Theta range for data collection	2.428 to 30.566°.	
Index ranges	-14<=h<=14, -16<=k<=16, -16<=l<=17	
Reflections collected	46052	
Independent reflections	7423 [R(int) = 0.0346]	
Completeness to theta = 25.242°	99.8 %	
Absorption correction	Semi-empirical from equivalents	
Max. and min. transmission	0.7461 and 0.6535	
Refinement method	Full-matrix least-squares on F^2	
Data / restraints / parameters	7423 / 0 / 343	
Goodness-of-fit on F^2	1.151	
Final R indices [I>2sigma(I)]	R1 = 0.0221, wR2 = 0.0447	
R indices (all data)	R1 = 0.0295, wR2 = 0.0500	
Largest diff. peak and hole	1.031 and -0.847 e.$Å^{-3}$	

Table S3. Crystal data and structure refinement for 3anzb182b_0ma_b_sq_sa.

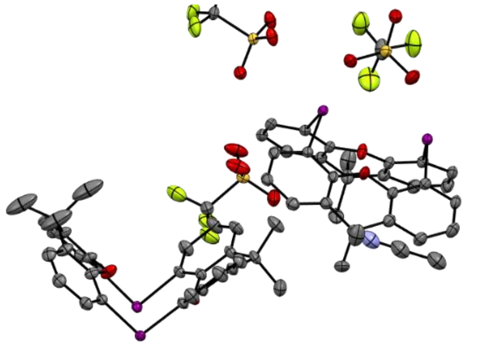

Identification code	3anzb182b_0ma_b_sq_sa	
Empirical formula	C66 H51 F12 I4 N O16 S4	
Formula weight	1977.91	
Temperature	293(2) K	
Wavelength	0.71073 Å	
Crystal system	Orthorhombic	
Space group	P n m a	
Unit cell dimensions	a = 34.6125(11) Å	α = 90°.
	b = 15.1719(4) Å	β = 90°.
	c = 14.8224(4) Å	γ = 90°.
Volume	7783.8(4) $Å^3$	
Z	4	
Density (calculated)	1.688 Mg/m^3	
Absorption coefficient	1.798 mm^{-1}	
F(000)	3864	
Crystal size	0.300 x 0.100 x 0.080 mm^3	
Theta range for data collection	1.921 to 30.562°.	
Index ranges	0<=h<=49, 0<=k<=21, 0<=l<=21	
Reflections collected	12298	
Independent reflections	12298 [R(int) = 0.0445]	
Completeness to theta = 25.242°	99.5 %	
Absorption correction	Semi-empirical from equivalents	
Max. and min. transmission	0.7461 and 0.6162	
Refinement method	Full-matrix least-squares on F^2	
Data / restraints / parameters	12298 / 0 / 479	
Goodness-of-fit on F^2	1.096	
Final R indices [I>2sigma(I)]	R1 = 0.0392, wR2 = 0.1107	
R indices (all data)	R1 = 0.0423, wR2 = 0.1145	
Largest diff. peak and hole	1.585 and -4.113 e.$Å^{-3}$	

Table S4. Crystal data and structure refinement for mo_3ANAAB44A_0m.

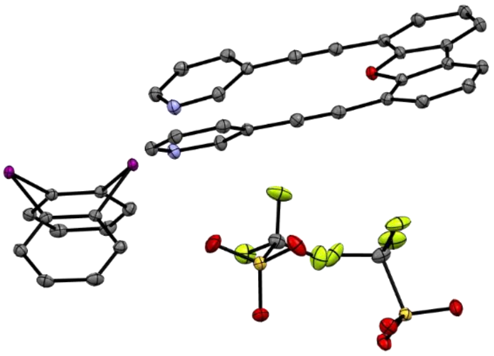

Identification code	mo_3anaab44a_0m	
Empirical formula	C40 H22 F6 I2 N2 O7 S2	
Formula weight	1074.51	
Temperature	100(2) K	
Wavelength	0.71073 Å	
Crystal system	Triclinic	
Space group	P -1	
Unit cell dimensions	a = 10.0233(3) Å	α = 75.3870(10)°.
	b = 10.7098(3) Å	β = 87.4370(10)°.
	c = 18.9012(5) Å	γ = 82.0840(10)°.
Volume	1944.55(10) $Å^3$	
Z	2	
Density (calculated)	1.835 Mg/m^3	
Absorption coefficient	1.807 mm^{-1}	
F(000)	1048	
Crystal size	0.102 x 0.067 x 0.028 mm^3	
Theta range for data collection	1.982 to 30.700°.	
Index ranges	-14<=h<=14, -15<=k<=15, -27<=l<=27	
Reflections collected	100739	
Independent reflections	11987 [R(int) = 0.0309]	
Completeness to theta = 25.242°	100.0 %	
Absorption correction	Semi-empirical from equivalents	
Max. and min. transmission	0.7461 and 0.6824	
Refinement method	Full-matrix least-squares on F^2	
Data / restraints / parameters	11987 / 0 / 532	
Goodness-of-fit on F^2	1.049	
Final R indices [I>2sigma(I)]	R1 = 0.0171, wR2 = 0.0385	
R indices (all data)	R1 = 0.0198, wR2 = 0.0397	
Largest diff. peak and hole	0.441 and -0.377 e.$Å^{-3}$	

Table S5. Crystal data and structure refinement for 3ANBZ163_0m_a_a.

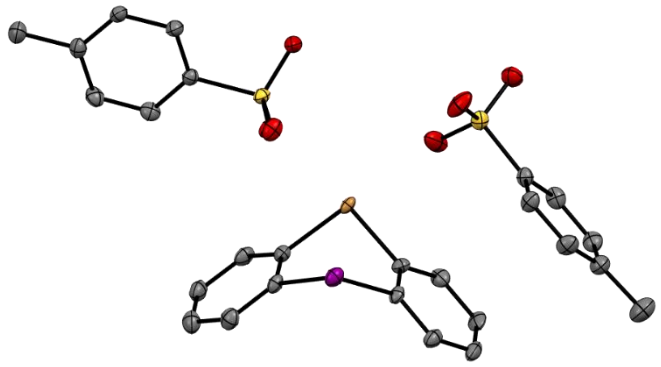

Identification code	3ANBZ163_0m_a_a	
Empirical formula	C26 H22 Br I O6 S2	
Formula weight	701.36	
Temperature	100(2) K	
Wavelength	0.71073 Å	
Crystal system	Triclinic	
Space group	P -1	
Unit cell dimensions	a = 9.9847(10) Å	α = 71.652(4)°.
	b = 10.7941(12) Å	β = 86.764(4)°.
	c = 13.5562(16) Å	γ = 66.298(4)°.
Volume	1265.6(2) $Å^3$	
Z	2	
Density (calculated)	1.840 Mg/m^3	
Absorption coefficient	3.050 mm^{-1}	
F(000)	692	
Crystal size	0.200 x 0.060 x 0.020 mm^3	
Theta range for data collection	2.175 to 30.594°.	
Index ranges	-14<=h<=14, -15<=k<=15, -19<=l<=19	
Reflections collected	48632	
Independent reflections	7783 [R(int) = 0.0482]	
Completeness to theta = 25.242°	100.0 %	
Absorption correction	Semi-empirical from equivalents	
Max. and min. transmission	0.7461 and 0.6020	
Refinement method	Full-matrix least-squares on F^2	
Data / restraints / parameters	7783 / 0 / 327	
Goodness-of-fit on F^2	1.056	
Final R indices [I>2sigma(I)]	R1 = 0.0449, wR2 = 0.1118	
R indices (all data)	R1 = 0.0618, wR2 = 0.1252	
Largest diff. peak and hole	3.068 and -2.335 e.$Å^{-3}$	

Table S6. Crystal data and structure refinement for 3ANZB179_0m_a.

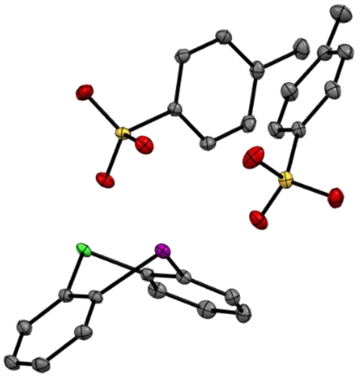

Identification code	3ANZB179_0m_a	
Empirical formula	C26 H22 Cl I O6 S2	
Formula weight	656.90	
Temperature	100(2) K	
Wavelength	0.71073 Å	
Crystal system	Triclinic	
Space group	P -1	
Unit cell dimensions	a = 9.9680(9) Å	α = 71.915(3)°.
	b = 10.6541(10) Å	β = 88.923(3)°.
	c = 13.5160(12) Å	γ = 67.040(3)°.
Volume	1247.8(2) Å^3	
Z	2	
Density (calculated)	1.748 Mg/m^3	
Absorption coefficient	1.600 mm^{-1}	
F(000)	656	
Crystal size	0.160 x 0.140 x 0.080 mm^3	
Theta range for data collection	2.199 to 30.605°.	
Index ranges	-14<=h<=14, -15<=k<=15, -19<=l<=19	
Reflections collected	45658	
Independent reflections	7637 [R(int) = 0.0286]	
Completeness to theta = 25.242°	99.8 %	
Max. and min. transmission	0.7461 and 0.6904	
Refinement method	Full-matrix least-squares on F^2	
Data / restraints / parameters	7637 / 0 / 327	
Goodness-of-fit on F^2	1.090	
Final R indices [I>2sigma(I)]	R1 = 0.0248, wR2 = 0.0595	
R indices (all data)	R1 = 0.0287, wR2 = 0.0626	
Largest diff. peak and hole	1.725 and -0.946 e.Å^{-3}	
